# Enantioselective Borylcupration/Cyclization of Alkene‐Tethered Oxime Esters

**DOI:** 10.1002/anie.202420479

**Published:** 2025-02-11

**Authors:** Jonathan Bajohr, Shangyu Li, Bijan Mirabi, Colton E. Johnson, Mark Lautens

**Affiliations:** ^1^ Department of Chemistry University of Toronto 80 St George St Toronto ON M5S 3H6 Canada

**Keywords:** Asymmetric Catalysis, Copper Borylation, 1-Pyrrolines

## Abstract

A copper‐catalyzed enantioselective synthesis of borylated 1‐pyrrolines from *γ*,*δ*‐unsaturated oxime esters is reported. Twenty‐four novel 1‐pyrroline derivatives are reported in yields ranging from 26% to 96% and enantioselectivities from 74.5:25.5 er to 99:1 er. Examples derived from *α*‐unsubstituted, non‐fluorinated oxime esters are reported. The hydroxyl group following oxidation of the Bpin moiety acts as a directing group in highly diastereoselective reductions of the pyrrolines to the corresponding prolinol derivatives. Additionally, the Bpin group can be retained in the products following a simplified, chromatography‐free workup procedure. DFT supports a reaction mechanism which proceeds through the formation of an (*R*)‐benzylic copper intermediate, followed by stereoretentive cyclization with respect to the configuration at the metalated carbon. The conclusions of the computational data are corroborated through control experiments and deuterium‐labelling studies.

## Graphical abstract

An asymmetric synthesis of boryl‐functionalized 1‐pyrrolines is reported. A range of *γ*,*δ*‐unsaturated oxime esters are compatible in the reaction and the borylated products are shown to be used in the synthesis of novel prolinol derivatives through a directed reduction of the cyclic imines. Experimental and computational investigations reveal that the reaction likely proceeds via a stereoretentive cyclization of the benzylic copper intermediates.

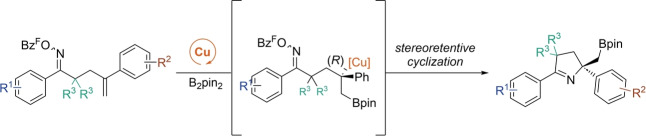

The borylcupration of π‐systems followed by electrophilic trapping of the intermediate organocopper species is a powerful tool to achieve difunctionalization of various olefins, alkynes, and allene derivatives.[^1^, ^2^, ^3^, ^4^, ^5^] These approaches to the synthesis of borylated targets are compatible with a wide range of electrophiles, and the resulting boron handles are sought after for subsequent reactivity. Moreover, when the substrates include a tethered electrophile, efficient cyclizations to enantioenriched hetero‐ and carbocycles can be accomplished.

1‐Pyrrolines are found in a variety of natural products and can be useful intermediates in the synthesis of pyrrole and pyrrolidine derivatives (Scheme 1A).[^6^, ^7^, ^8^] Relatively simple alkaloids such as myosmine and eudistomin I have been prepared by the condensation of alkylamines onto a tethered carbonyl moiety.[^9^, ^10^] An increasingly prevalent synthetic approach for the construction of 1‐pyrrolines involves the cyclization of alkene‐tethered oxime esters.^[11]^ This strategy is exemplified in the 2016 total synthesis of gelesenicine by the Ferreira group,^[12]^ with the pyrroline being constructed in the final step via an AIBN/Bu_3_SnH promoted formation and cyclization of an iminyl‐radical onto a proximal alkene.

**Scheme 1:**
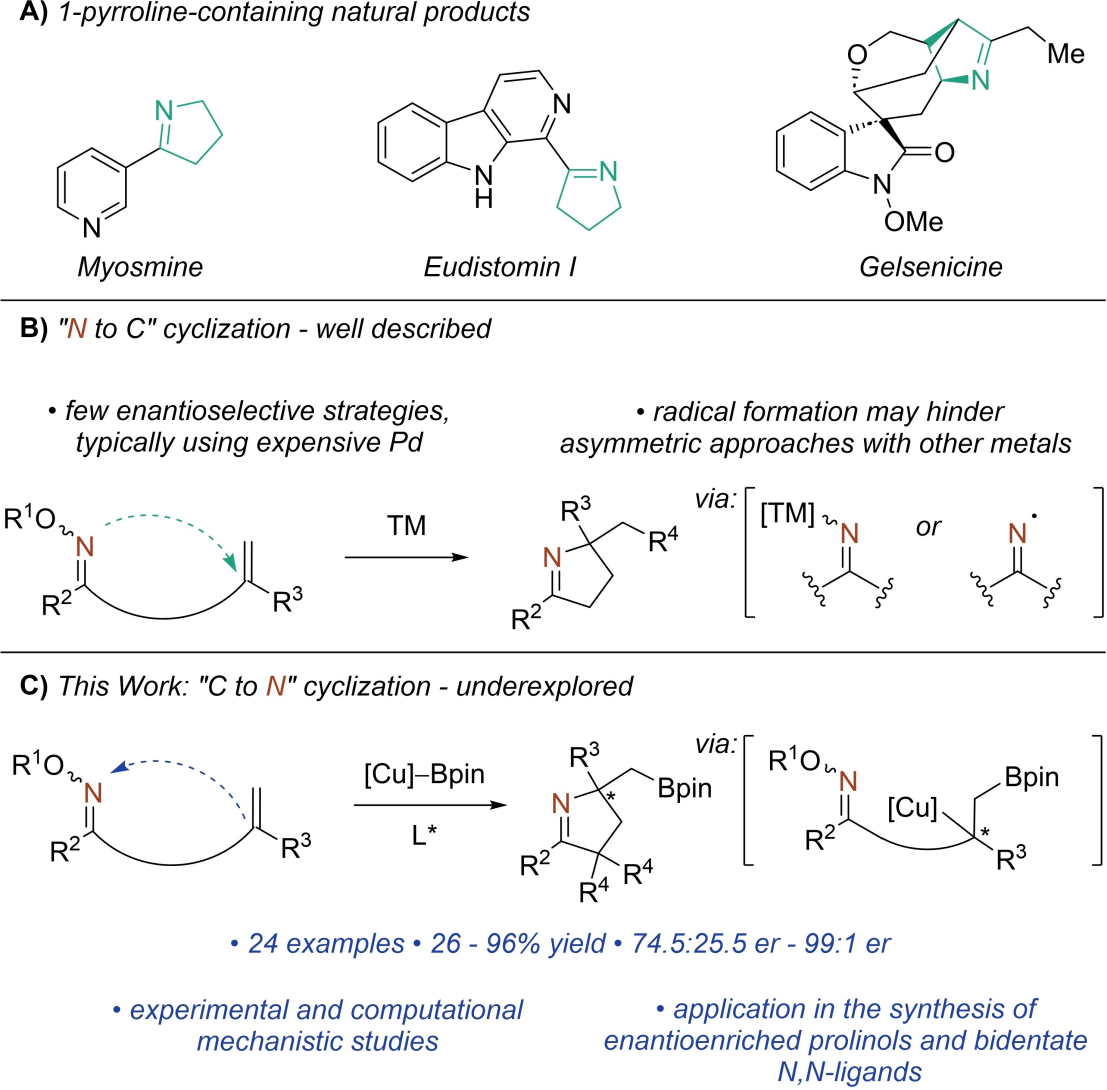
Cyclization strategies of alkene‐tethered oxime esters through traditional *“N to C”* methods, and the current *“C to N”* strategy via asymmetric borylcupration.

Transition metals also promote these cyclizations, proceeding through either an initial oxidative addition into the N−O bond followed by migratory insertion, or via formation and cyclization of iminyl radicals (Scheme 1B).^[13][14]^ The development of asymmetric cyclizations of alkene‐tethered oxime esters using palladium (asymmetric Narasaka‐Heck reactions) has trailed behind other asymmetric Heck‐type cyclizations due to the potential of competing SET pathways.[^15^, ^16^, ^17^, ^18^, ^19^] Similarly, promoting the desired cyclizations using less expensive copper or nickel catalysts frequently proceeds via radical pathways.[^20^, ^21^, ^22^, ^23^, ^24^, ^25^, ^26^, ^27^, ^28^, ^29^] We hypothesized that the limitations associated with the aforementioned “*N to C*” cyclizations of this class of substrates could be bypassed if the initiating step of the cascade excluded the N−O bond.

Continuing our interest in the development of asymmetric borylcupration/cyclization methodologies,^[30]^ we envisioned that by employing *γ*,*δ*‐unsaturated oxime esters in concert with copper borylation strategies, a highly enantioselective “*C to N*” cyclization could be realized (Scheme 1C), which would afford enantioenriched boryl‐functionalized pyrrolines. The Wu group in 2020 demonstrated that oxime esters bearing tethered terminal alkenes engaged in borylative cyclizations, however the mechanism of the cyclization step, as well as an asymmetric variant of this transformation, have yet to be disclosed.^[31]^

We commenced our efforts to address these goals using our library of pentafluorinated oxime esters **1 a–x** as model substrates in the asymmetric Cu‐borylation/cyclization reaction.[^32^, ^33^] Optimized reaction conditions comprised of [Cu(MeCN)_4_]PF_6_ (5 mol %), *S,S*‐BDPP (7.5 mol %), NaO*t*Bu (2.2 equiv), B_2_pin_2_ (1.5 equiv) in THF at room temperature effectively produced the desired enantioenriched borylated pyrrolines (Scheme 2, see the Supporting Information for details on reaction optimization). The instability of compounds **2 a–x** during silica‐gel flash column chromatography purification led to their isolation as the corresponding alcohols **3 a–x**, after oxidation. However, it is important to highlight that the synthetically useful Bpin handle could be retained in select products by following a simplified chromatography‐free purification protocol (*vide‐infra*). Under the optimized conditions, unsubstituted pyrroline **3 a** was isolated in 87 % yield and 96.5 : 3.5 er. Alkyl substituents, aryl chlorides and dioxolane rings were tolerated on the aryl ring adjacent to the oxime ester yielding pyrrolines **3 b**‐**3 e**, demonstrating excellent yields and enantioselectivities throughout. Electron‐withdrawing groups (**3 f**, **3 g**) and a methoxy group (**3 h**) at the *meta*‐ position of the styrene showed comparable results. Electron withdrawing *para‐* trifluoromethyl or fluoro‐ substituents delivered products **3i** and **3j** in excellent yields, with slightly reduced enantioselectivity (92.5:7.5 er) observed in the case of trifluoromethyl substitution. When introducing an ester in this position (**3k**), excellent yields were maintained although the enantioselectivity was further reduced to 74.5:25.5 er. Alkyl or aryl (**3 l, 3 m**) groups in this position were successfully incorporated, including **3n** (86%, 96.5:3.5 er) containing a bulky *tert‐*butyl group. Disubstituted arenes yielded products **3 o** (80 %, 95.5 : 4.5 er) and **3 p** (76 %, 98 : 2 er). The absolute stereochemistry of **3 p** was determined by single‐crystal X‐ray crystallography, and the other products were assigned by analogy. Styrenes bearing dioxolane rings or a pentadeuterated arene gave products **3 q** (78 %, 96 : 4 er) and **3 r** (85 %, 96.5 : 3.5 er). Pyrroline **3 s** bearing a 2‐pyridyl group was isolated in 55 % yield and excellent enantioselectivity (99 : 1 er). The rationale behind the design of **3 s** was that a bidentate *N,N*‐ligand could be derived from this scaffold. An initial proof‐of concept study forming a Ni‐complex with **3 s** (see the Supporting Information for more details) was successful, and studies are currently underway investigating derivatives of such ligands for asymmetric catalysis. Product **3t,** with a 2‐chloropyridyl motif was isolated in 89% yield, although exhibited a low enantiomeric ratio of 78:22. Replacing the *gem*‐dimethyl group with a cyclohexane ring yielded spirocyclic pyrroline **3 u** in excellent yield and er. Trisubstituted pyrrolines were successfully synthesized from the corresponding *α*‐unsubstituted *γ*,*δ*,*–*unsaturated oxime esters, which were unreactive in previous studies.^[31]^ Both alkyl (**3 v**) and halogen substituents (**3 w**) were incorporated into these products, albeit in lower yield and enantioselectivity; a trend also observed in related processes.^[34]^ Notably, product **3 x** was isolated in 34 % yield and 91 : 9 er from the corresponding *α*‐unsubstituted, non‐fluorinated substrate, showcasing reactivity across a broad range of oxime‐ester derivatives. Substrates that did not engage in the desired reactivity include alkyl‐substituted alkene **1y**, *para*‐methoxy styrene **1z**, as well as benzo‐fused substrate **1aa**.

**Scheme 2:**
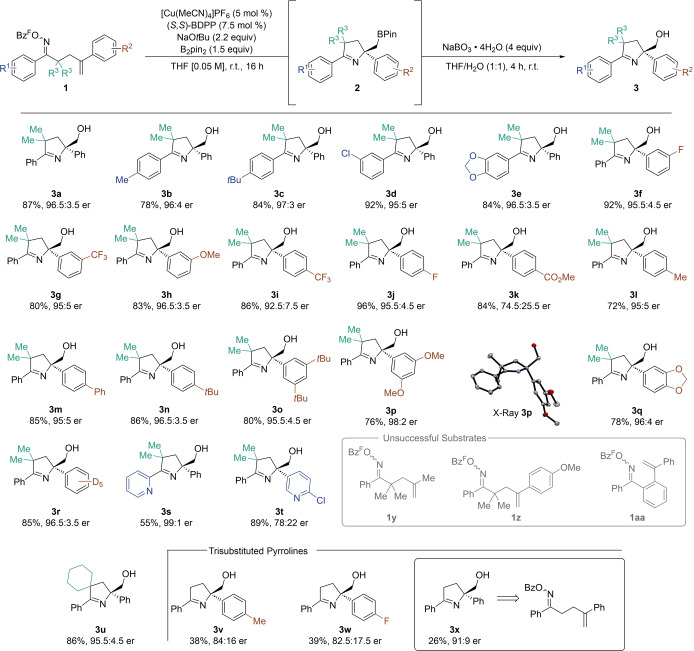
Accessible enantioenriched pyrrolines. Reactions run on 0.2 mmol scale. All yields represent isolated, purified material unless noted otherwise. Enantiomeric ratios determined via HPLC using a chiral stationary phase. Bz^F^=pentafluorobenzoyl. BDPP=2,4‐bis(diphenylphosphino)pentane.

As previously noted, retaining the Bpin group in the pyrroline products was possible using a chromatography‐free work‐up procedure. The synthesis of borylated pyrroline **2 p** was accomplished on 1 mmol scale in 88 % yield and >99 : 1 er (Scheme 3A). Pyrrolines can serve as precursors to prolinols, which can in turn be applied towards the synthesis of non‐canonical proline derivatives or used as chiral auxiliaries.[^35^, ^36^, ^37^, ^38^] The primary alcohol in the product was shown to direct the reduction of the cyclic imine, as seen with the diastereoselective formation of prolinol **4** from **3 p** (74 % yield, >20 : 1 dr) using NaBH_4_ in MeCN/AcOH at −35 °C (Scheme 3B).[^39^, ^40^]

**Scheme 3:**
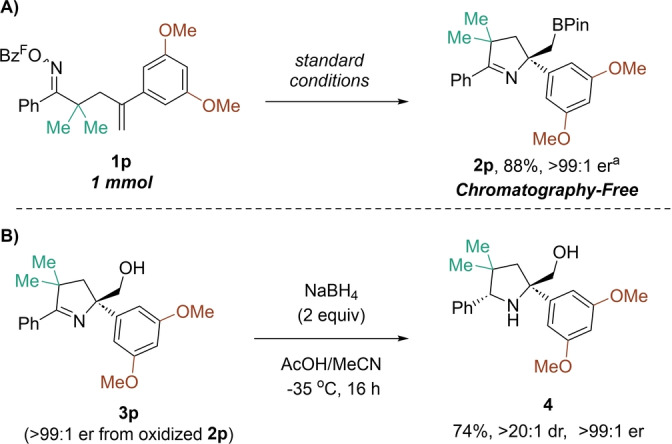
All yields represent isolated, purified material unless noted otherwise. Enantiomeric ratios determined via HPLC using a chiral stationary phase. Bz^F^=pentafluorobenzoyl. A) Scale‐up synthesis of Bpin containing product **2 p**. [a] enantiomeric ratio determined following trituration with pentanes. For standard conditions, see Scheme 2. B) Diastereoselective synthesis of enantioenriched prolinol **4**. Reaction run on 0.1 mmol scale. dr determined from ^1^H NMR analysis of crude reaction mixture.

We turned to density functional theory (DFT) to investigate the reaction mechanism of this transformation.[^41^, ^42^] Geometry optimization and vibrational analyses were performed using the *ω*B97X‐D^[43]^ functional with the 6–31 g* Pople basis set[^44^, ^45^] for main group elements. The LANL2DZ basis set and its effective core potential (ECP) was used for copper.[^46^, ^47^, ^48^, ^49^] Single‐point energies were re‐evaluated at the B3LYP−D3 level using the 6–311+g** basis set for main group elements and the SDD basis set and ECP for copper.^[46][50–57]^ The effect of solvation was evaluated with the SMD model utilizing default values for THF.^[58]^

The generally accepted mechanism for copper‐catalyzed borylation reactions involves initial migratory insertion of a Cu–Bpin species into a π‐bond, followed by electrophilic trapping. We were particularly interested in the latter step, since both stereoinvertive and stereoretentive mechanisms have been proposed.[^59^, ^60^, ^61^] Scheme 4 shows the free energy profile for the borylimination of substrate **1 a**. Binding of the *re* face of substrate **1 a** to the ligated Cu–Bpin species **I** was found to be endergonic by 6.2 kcal mol^–1^ (**II‐*re***); comparatively, binding of the *si* face was endergonic by only 2.7 kcal mol^–1^ (**II‐*si***). Our computations indicate that borylcupration via **II‐*si*‐TS** (ΔG^≠^=22.2 kcal mol^–1^) leading to the (*S*)‐benzylic copper intermediate **III‐(*S*)** is less favored than the diastereomeric transition state **II‐*re*‐TS** (ΔG^≠^=18.5 kcal mol^–1^, ΔΔG^≠^=3.7 kcal mol^–1^) leading to the (*R*)‐benzylic copper **III‐(*R*)**. These results suggest that the preferred migratory insertion under the reaction conditions leads selectively to **III‐(*R*)**. An invertive cyclization traversing through **III‐TS‐inv** was found to have an activation barrier of 10.6 kcal mol^–1^. Release of the Cu–OBz^F^ species with concomitant formation of the (*S*)‐enantiomer of the borylated product **IV‐(*S*)** was highly exergonic (ΔG=‐82.7 kcal mol^–1^). Notably, this is the minor enantiomer of the product that is observed experimentally. The stereoretentive cyclization transition state **III‐TS‐ret** leading to **IV‐(*R*)** was found to be much more favorable with an activation barrier of only 2.8 kcal mol^–1^ (ΔΔG^≠^=7.8 kcal mol^–1^). We highlight that neither transition state featured coordination to the boron atom of the Bpin group.^[30]^

**Scheme 4:**
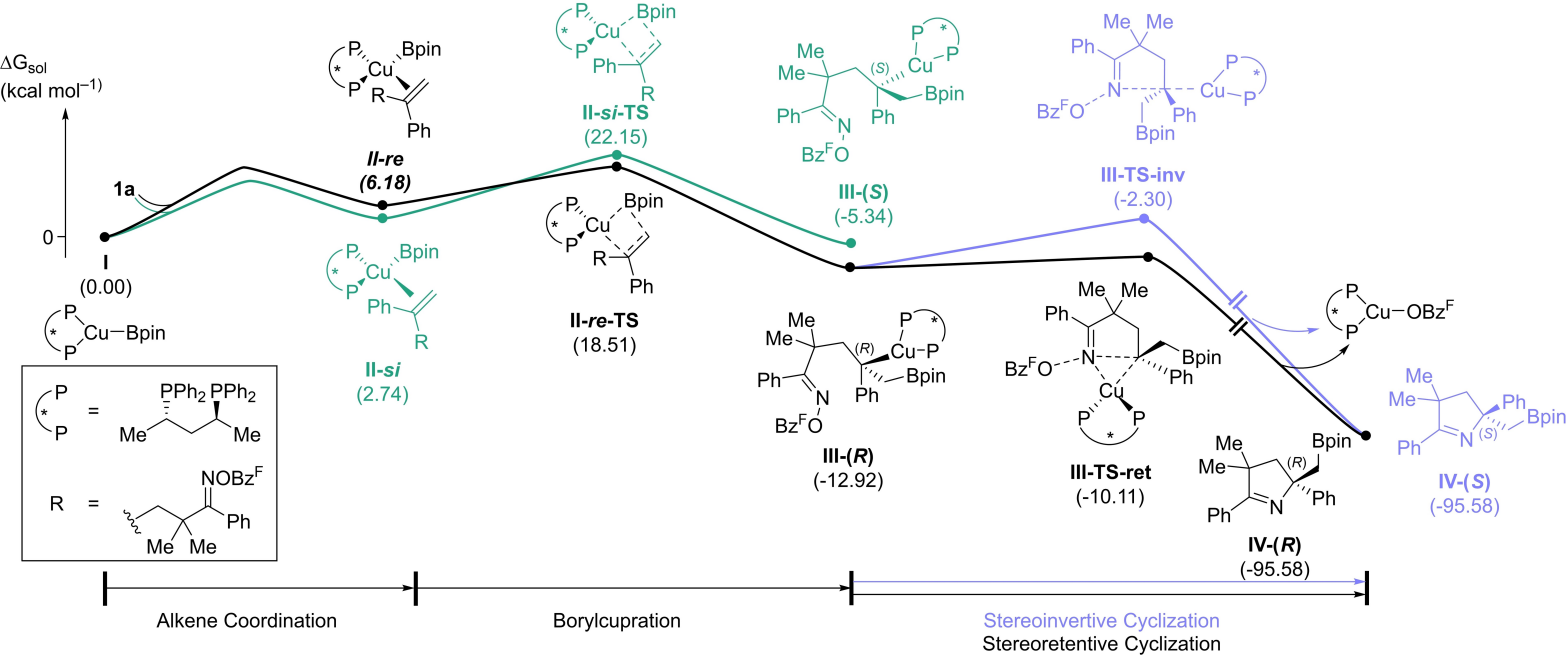
Computed solvation free energy profile of the reaction

Our group has previously proposed that borylcupration of similar substrates would occur via the stereoinvertive manifold.[^62^, ^63^, ^64^] The current computational results cast doubt on this previous stereoinduction model.^1^ The current computational results are consistent with experimental observations (*vide infra*). The stereochemical course of related borylcupration methodologies remain to be established in a similar fashion. To rationalize the preference for a retentive over invertive cyclization mechanism, we performed an analysis of the non‐covalent interactions (NCIs) present in **III‐TS‐ret** and **III‐TS‐inv** using the Multiwfn software (see the Supporting Information for NCI plots).^[65]^ The qualitative results reveal additional stabilizing interactions in **III‐TS‐ret** that could account for the significant preference to undergo stereoretentive cyclization. Typically, the invertive pathway is invoked to minimize unfavorable steric interactions as the copper catalyst is geometrically positioned further from the linker in **III‐TS‐inv** than in **III‐TS‐ret**. However, in the latter case, the close proximity of the catalyst to the linker leads to a strong NCI between one of the phenyl rings on the ligand and one of the methyl groups on the linker, as well as a greater number of NCIs between the ligand phenyl groups and the phenyl group on the benzylic carbon (Scheme 5). The combination of these interactions could explain the preference for stereoretention over stereoinversion. Additionally, the presence of the NCI between the *gem‐*dimethyl group and the ligand could account for the significant loss in enantioselectivity in products **3 v**‐**3 x**.

**Scheme 5:**
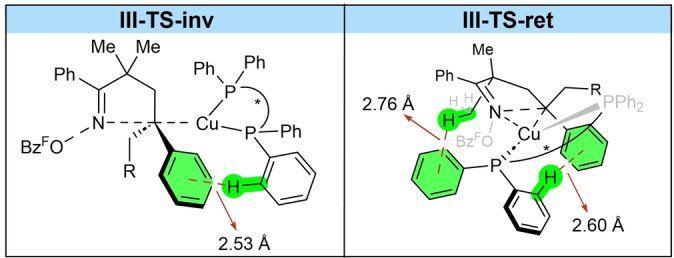
Schematic representation of Key NCIs in the competitive cyclization transition states. See the Supporting Information for full NCI plots.

To support our computational studies, control experiments and synthetic derivatizations were performed (Scheme 6). Substrate **1 b** was subjected to the optimized reaction conditions in the presence of TEMPO (Scheme 6A). While copper‐catalyzed asymmetric radical‐cross couplings with electrophilic nitrogen sources have been proposed,^[66]^ the current system was not influenced by the radical scavenger and a single‐electron pathway is unlikely to be operative. Experimental evidence for the diastereoselective formation of (*R*)‐benzylic copper species **III‐(*R*)** from initial asymmetric borylcupration was achieved by employing deuterated substrate **1 a‐D**, in a similar strategy reported by Shimizu and Kanai,^[59]^ as well as Tong.^[67]^ No deuterium scrambling was observed when subjecting **1 a‐D** to the standard reaction conditions (Scheme 6B). The resulting stereochemistry at the deuterated position of **3 a‐D** was uncovered through a reduction/cyclic carbamate ester formation sequence (Scheme 6C) to lock the conformation. Once again, the pyrroline reduction proceeded with high diastereoselectivity, delivering the hydride *syn*‐ with respect to the alcohol substituent. Upon treatment of the corresponding prolinol with triphosgene and DIPEA, cyclic carbamate **5** was isolated and the stereochemistry of the product confirmed by 2D NOE experiments. The structure of **5** is consistent with a mechanism in which the formation of the (*R*)‐benzylic copper species **III‐*(R*)** through a *syn*‐borylcupration, cyclizes in a stereoretentive fashion to **IV‐(*R*)**. This outcome is consistent with a borylcupration stereoinduction model proposed by the Procter group in 2021.^[68]^

**Scheme 6:**
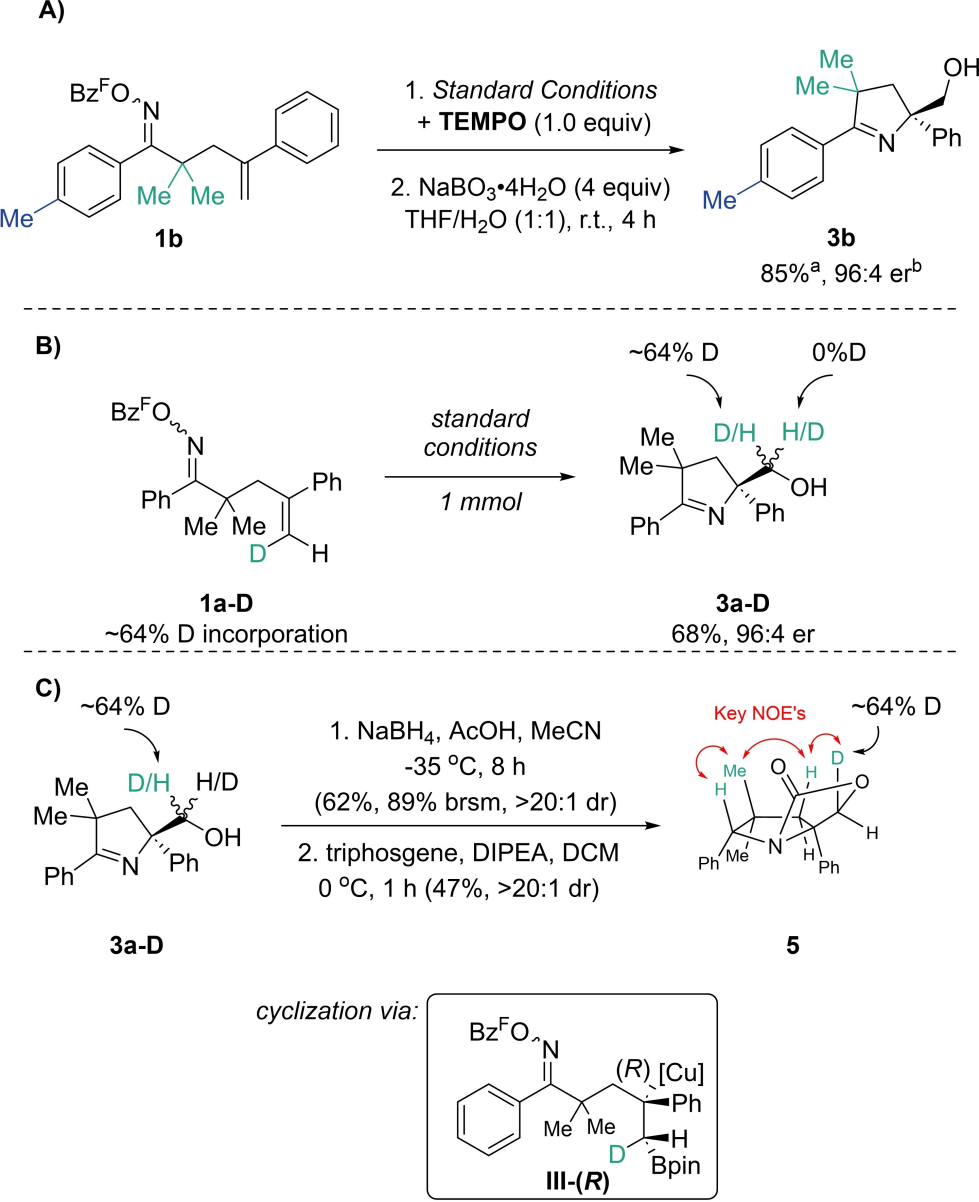
Control experiments and deuterium labelling studies. All yields represent isolated, purified material unless noted otherwise. Enantiomeric ratios determined via HPLC using a chiral stationary phase. dr determined by ^1^H NMR analysis of crude reaction mixtures. Bz^F^=pentafluorobenzoyl. [a] ^1^H NMR yield of the borylated product **2 b** [b] enantiomeric ratio of alcohol **3 b**.

In summary, we have developed an effective asymmetric synthesis of borylated 1‐pyrrolines from *γ*,*δ*‐unsubstituted oxime esters. Various substitution patterns were tolerated in the borylation reaction, including the incorporation of heteroaromatic rings. Additionally, the range of substrates known to engage in borylative cyclizations to 1‐pyrrolines has been expanded to *α*‐unsubstitued oxime esters, including non‐fluorinated variants. The Bpin handle can be readily oxidized and the resulting primary alcohol can direct the reduction of the C−N double bond of the products with high diastereoselectivity, giving rise to prolinol derivatives. DFT analysis of the present reaction suggests that the cyclization occurs with stereoretention and that the decreased distance between the catalyst and the linker in the transition state leads to stabilizing NCIs. When a stereodefined, mono‐deuterated alkene was subjected to the reaction conditions followed by a reduction/cyclic carbamate formation sequence, the stereochemical course of the borylation/cyclization cascade was experimentally uncovered. This report continues to underscore the importance and applicability of asymmetric copper borylation strategies towards the synthesis of new enantioenriched heterocycle derivatives.

## Supporting Information

The authors have cited additional references within the Supporting Information.

## Conflict of Interests

The authors declare no conflict of interest.

## Supporting information

As a service to our authors and readers, this journal provides supporting information supplied by the authors. Such materials are peer reviewed and may be re‐organized for online delivery, but are not copy‐edited or typeset. Technical support issues arising from supporting information (other than missing files) should be addressed to the authors.

Supporting Information

## Data Availability

The data that support the findings of this study are available in the supplementary material of this article.

## Footnotes

n1 Our previous model was inspired by observations from the Meek group, which suggested that invertive pathways are preferred during sterically demanding electrophilic trapping within this class of transformation, and that the BDPP ligand underwent cyclization in an invertive fashion. See references 63 & 64 for more information.
