# Supplementary material for: Enantioselective Borylcupration/Cyclization of Alkene‐Tethered Oxime Esters
Source: Angew Chem Int Ed Engl. 2025 Feb 11;64(10):e202420479. doi: 10.1002/anie.202420479 (PMC11878337; doi:10.1002/anie.202420479)
Supplement: Supplementary file 1 — Supporting Information [file ANIE-64-e202420479-s001.pdf]

Angewandte Chemie
Eine Zeitschrift der Gesellschaft Deutscher Chemiker

Supporting Information

Enantioselective Borylcupration/Cyclization of Alkene-Tethered Oxime Esters

J. Bajohr, S. Li, B. Mirabi, C. E. Johnson, M. Lautens**

Enantioselective Borylcupration/Cyclization of Alkene-Tethered Oxime Esters

Jonathan Bajohr, Shangyu Li, Bijan Mirabi*, Colton E. Johnson and Mark Lautens*

mark.lautens@utoronto.ca
bijan.mirabi@alumni.utoronto.ca

Department of Chemistry, University of Toronto, 80 St. George Street, Toronto, Ontario, M5S 3H6, Canada

Supporting Information

Contents

General Considerations 2
Optimization Tables 3
General Procedures 6
Starting Material Characterization 14
Known Starting Materials 14
New Starting Materials 15
Unsuccessful Substrates 18
Product Characterization 19
***E/Z* Oxime Ester Determination of Substrate 1a** 48
Computational Details 51
NCI Plots 51
Computed Energy Components 56
Cartesian Coordinates 57
NMR Spectra 102
3p Crystallographic Data 158
3s-Ni Crystallographic Data 170
References 183

General Considerations

All reactions were performed in flame-dried glassware under argon. Reaction progress was monitored by TLC using UV light, $KMnO_4$ or CAM stain for visualization. THF was distilled over sodium/benzophenone prior to use. MeCN was distilled over CaH prior to use. HPLC grade hexanes and isopropanol were purchased from Fisher. All other reagents were purchased from Sigma, Alfa, Fisher or Combi-Blocks and were used as received. NaO*t*Bu was purified by sublimation at elevated temperature under vacuum or purchased from Sigma and stored in a glove box. Catalytic reactions were performed in flame-dried, 2 dr vials, equipped with a PTFE-lined septa (ThermoScientific National B7995-15) and a stir bar (Fisher cat. No. 14-513-57, 12 x 4.5 mm). An oil bath was used as the heating source for reactions when needed. Reactions ran at -35 °C were run in a bath of isopropyl alcohol cooled in a NESLAB cryotroll CB-80. Flash column chromatography was performed with Silicycle 46-60 μ, silica gel.

^{1}H and ^{13}C NMR spectra were obtained at 296 K on an Agilent DD2 600 MHz spectrometer, Agilent DD2 500 MHz spectrometer equipped with a 5 mm Xses Cold Probe or a 400 MHz Varian MercuryPlus NMR Spectrometer. ^{19}F NMR were obtained on a Bruker Avance III 400. Measurements were referenced to the solvent. NMR data are referenced as chemical shift (δ ppm), multiplicity (s = singlet, d = doublet, t = triplet, q = quartet, ABq = AB quartet, m = multiplet, b = broad), coupling constant (Hz).

NMR yields were obtained by ^{1}H NMR analysis using a 5 second relaxation delay unless stated otherwise, and 1,3,5- trimethoxybenzene as an internal standard. HRMS were obtained on a JEOL AccuTOF-DART performed at the Advanced Instrumentation for Molecular Structure (AIMS) at the University of Toronto. IR spectra were acquired on a Perkin-Elmer Spectrum 100 instrument with a single- bounce diamond/ZeSe ATR accessory. Data is presented in wavenumbers (cm^{-1}). Chiral HPLC was performed on Agilent 1100 or 1200 series operated by ChemStation LC 3D software. Specific rotation values were obtained on a Rudolph Research Analytical Autopol® IV Automatic Polarimeter.

Optimization Tables

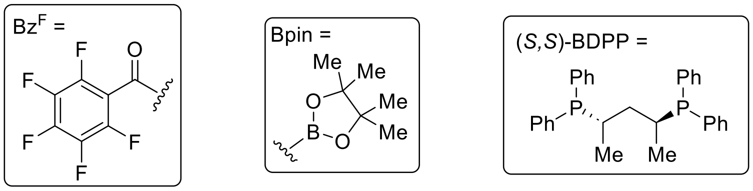

Table S1. Initial Reaction Condition Screening

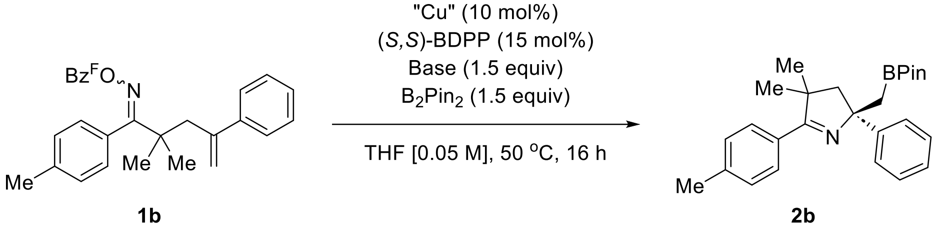

Entry	Copper Source	Base	Yield 2b (%)[a]	er of 2b[b]
1	CuCl	LiO*t*Bu	trace	N.D.
2	CuCl	NaO*t*Bu	55	94:6
3	$[Cu(MeCN)_4]PF_6$	LiO*t*Bu	66	95:5
4	$[Cu(MeCN)_4]PF_6$	NaO*t*Bu	60	95:5

Table S1: Reactions run on a 0.1 mmol scale unless noted otherwise. [a] Yield determined by ^{1}H NMR of the crude reaction mixture using 1,3,5-trimethoxybenzene as internal standard. [b] Determined by HPLC using chiral stationary phase. N.D. = Not determined

Table S2. Variations of Base, Solvent, Temperature and Reaction Time Using 5 mol % Copper[A]

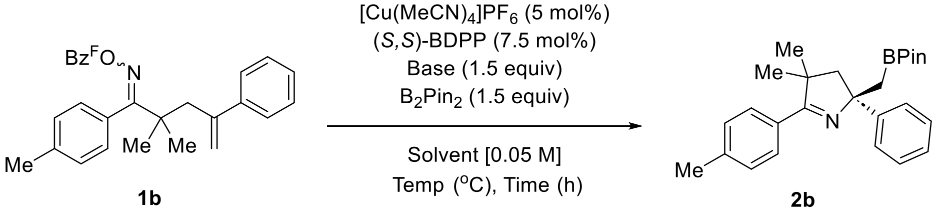

Entry	Base	Solvent	Temp (°C)	Time (h)	Yield 2b (%)[a]
1	LiO*t*Bu	THF	50	22	53
2	NaO*t*Bu	THF	50	22	60
3	NaO*t*Bu	THF	60	15	66
4	NaO*t*Bu	THF	80	15	64
5	NaO*t*Bu	1,4-dioxane	50	16	77
6	NaO*t*Bu	MeCN	50	16	36
7	NaO*t*Bu	Toluene	50	16	29
8	NaO*t*Bu	DCE	50	16	trace

Table S2: Reactions run on a 0.1 mmol scale unless noted otherwise. [a] Yield determined by ^{1}H NMR of the crude reaction mixture using 1,3,5-trimethoxybenzene as internal standard

[A] Following this point in the optimization, accurate acquisition of enantiomeric ratios (er's) were obtained from the isolated oxidized material **3b**. The borylated pyrroline **2b** decomposed slightly upon purification using silica-gel column chromatography, and results in slight overlap of impurity peaks in the HPLC trace.

Table S3. Variations of Base, Temperature and Reaction Time in 1,4-dioxane

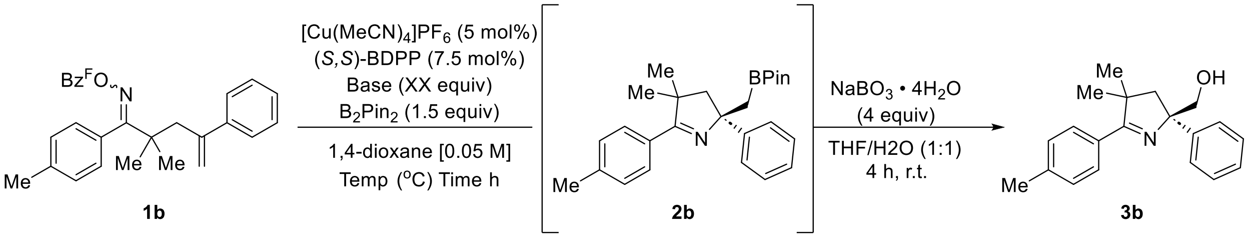

Entry	Base	Temp (°C)	Time (h)	Yield (%)[a,b]	er[c]
1	NaO*t*Bu (1.5 equiv)	50	16	81	94:6
2	NaOMe (1.5 equiv)	50	16	53	N.D.
3	KO*t*Bu (1.5 equiv)	50	16	81 (75)	93:7
4	NaO*t*Bu (1.5 equiv)	60	16	55	N.D.
5	LiO*t*Bu (1.5 equiv)	50	16	53	N.D
6	NaO*t*Bu (1.5 equiv)	65	20	59	N.D
7	NaO*t*Bu (1.5 equiv)	50	16	50	N.D
8	NaO*t*Bu (3 equiv)	50	16	65 (65)	93:7
9	NaO*t*Bu (2.2 equiv)	50	16	63	94:6

Table S3: Reactions run on a 0.1 mmol scale unless noted otherwise. [a] Yield of **2b** determined by ^{1}H NMR of the crude reaction mixture using 1,3,5-trimethoxybenzene as internal standard. [b] Isolated yield of **3b** in parentheses. [c] er of **3b** determined by HPLC using chiral stationary phase. N.D. = Not Determined

Table S4. Variation of Copper Source, Ligand, Solvent, and Temperature Using NaO*t*Bu (2.2 equiv)

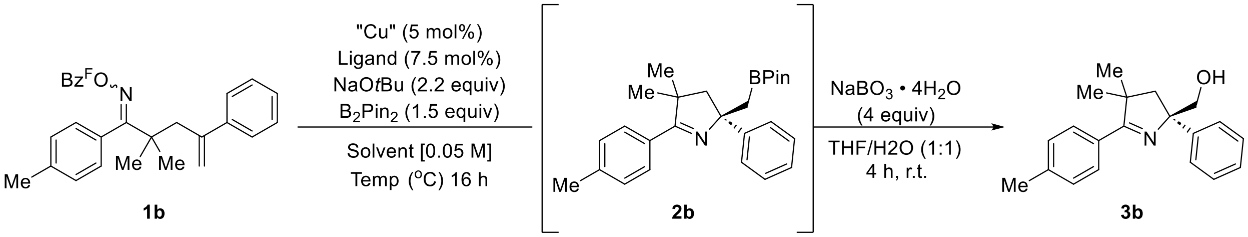

Entry	Copper Source	Ligand	Solvent	Temp (°C)	Yield (%)[a,b]	er[c]
1	$[Cu(MeCN)_4]PF_6$	(*S,S*)-BDPP	MTBE	50	87 (82)	94:6
2	$[Cu(MeCN)_4]PF_6$	(*S,S*)-BDPP	THF	50	87 (79)	94.5:5.5
3	$[Cu(MeCN)_4]PF_6$	(*S,S*)-Ph-BPE	THF	50	39	93:7
**4**	$\mathbf{[Cu(MeCN)_4]PF_6}$	**(*S,S*)-BDPP**	**THF**	**r.t.**	**80**	**96:4**
5	$[Cu(MeCN)_4]PF_6$	SL-J002-1	THF	50	trace	N.D.
6	CuCl	(*S,S*)-BDPP	THF	r.t.	62	N.D.
**7[d]**	$\mathbf{[Cu(MeCN)_4]PF_6}$	**(*S,S*)-BDPP**	**THF**	**r.t.**	**87 (78)**	**96:4**
8[e]	$[Cu(MeCN)_4]PF_6$	(*S,S*)-BDPP	THF	r.t.	95	96:4

Table S4: Reactions run on a 0.1 mmol scale unless noted otherwise. [a] Yield of **2b** determined by ^{1}H NMR of the crude reaction mixture using 1,3,5-trimethoxybenzene as internal standard. [b] Isolated yield of **3b** in parentheses. [c] er of **3b** determined by HPLC using chiral stationary phase. [d] Run on a 0.2 mmol scale. [e] Using **1b** bearing an NOBz (Bz = Benzoyl) group instead of NOBz^F. N.D. = Not Determined

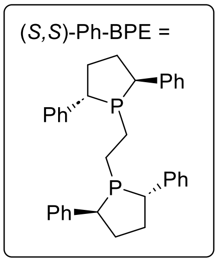

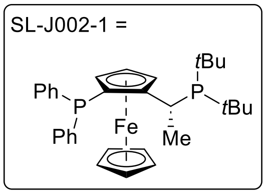

General Procedures

General Procedure 1 (GP1): Synthesis of isobutyropheneone derivative INT-S1

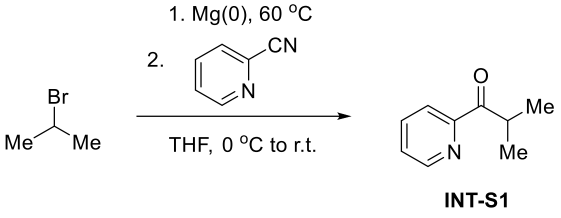

Isobutyrophenone derivative **INT-S1** was synthesized from the corresponding Grignard addition to 2-cyanopyridine. Used in the synthesis of **1s** (GP2).

In a flame-dried, round bottom flask under argon atmosphere were added magnesium turnings (700 mg, 28.8 mmol, 1.4 equiv), a small crystal of iodine and THF (24 mL). 2-bromopropane (2.25 mL, 24 mmol, 1.2 equiv) was added dropwise, and the yellow solution became clear and was stirred at 60 °C for 1 hour before cooling to room temperature. The Grignard solution was then transferred dropwise via syringe to a solution of 2-cyanopyridine (2.08 g, 20 mmol, 1.0 equiv) in 12 mL of THF at 0 °C. The solution was stirred overnight, gradually warming to room temperature. The reaction was quenched with 1M HCl, and extracted with EtOAc (3 x 50 mL). The combined organic extractions were combined, dried over $MgSO_4$, filtered and concentrated under vacuum. The crude residue was purified by silica-gel column chromatography to yield **INT-S1** as a clear oil (1.9 g, 13 mmol, 65% yield), matching previously reported spectroscopic data.[1]

General Procedure 2 (GP2): Synthesis of alkene-tethered oxime esters bearing *gem*-dimethyl groups

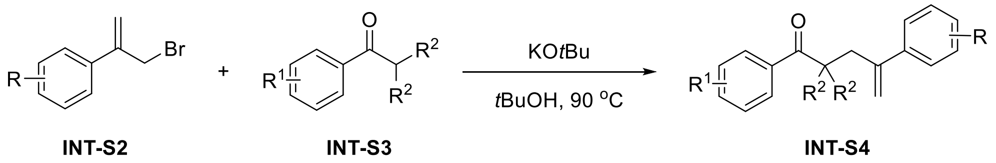

Allylic bromides **INT-S2** were synthesized according to Zhao and coworkers (2018).[2] Reaction typically performed on a 10-20 mmol scale. To a solution of **INT-S1** or isobutyrophenone (**INT-S3**, R^1 = H, R^2 = Me) in tert-butanol [0.33M] at room temperature in a flame-dried round-bottom flask was added KO*t*Bu (5 equiv) and the mixture was stirred for 10 minutes. The corresponding allylic bromide **INT-S2** (1.5 equiv) was then added dropwise, and the reaction mixture was refluxed at 90 °C. Once the starting material had been consumed (typically after 4 hours), monitored by TLC, the reaction was cooled to room temperature, diluted with H_2O and EtOAc, and the aqueous layer was extracted with EtOAc (3 x 20 mL). The combined organic layers were dried over $MgSO_4$, filtered and concentrated under vacuum to give **INT-S4** as orange oils. The crude ketones were passed through a plug of silica gel, eluting with 1:1 DCM/pentane, concentrated under vacuum and used in the next step without further purification.

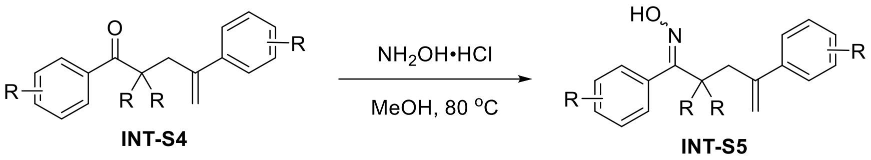

Reactions typically performed on a 5-10 mmol scale. Hydroxylamine-HCl (4 equiv) and sodium acetate (7 equiv) were added to a solution of **INT-S4** in methanol [0.5M] at room temperature. The reaction mixture was refluxed at 80 °C for 4 hours or until the starting material had been consumed, monitored by TLC. The reaction was cooled to room temperature, diluted with EtOAc and washed with brine. Concentration of the organic layer under vacuum gave crude **INT-S5** as white solids, which were triturated with pentanes prior to being used in the next step.

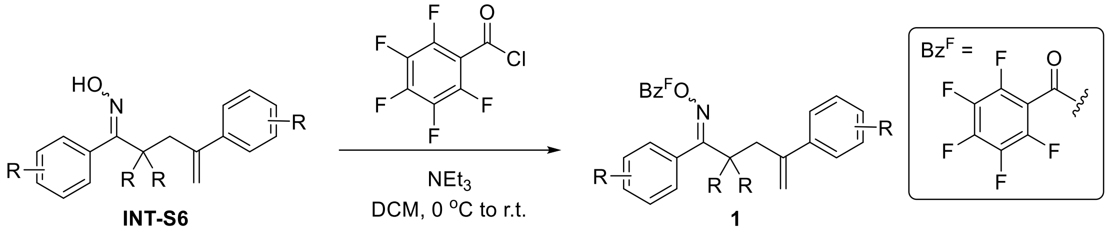

Reaction typically performed on a 5 mmol scale. In a flame-dried round-bottom flask under argon atmosphere was added **INT-S6**, anhydrous DCM [0.5M] and anhydrous triethylamine (2 equiv). The solution was cooled to 0 °C and pentafluorobenzoyl chloride (1.2 equiv) was added dropwise. The reaction was warmed to room temperature, and upon consumption of the starting material (monitored by TLC), quenched with H_2O. DCM was added and the organic layer was washed with brine, dried over $MgSO_4$, filtered and concentrated under vacuum. The crude material was purified by silica gel column chromatography (typically 0% → 50% DCM in pentanes, gradient) to afford purified substrates **1o** and **1s**.

<u>General Procedure 3 (GP3): Synthesis of α-unsubstituted alkene-tethered oxime esters</u>

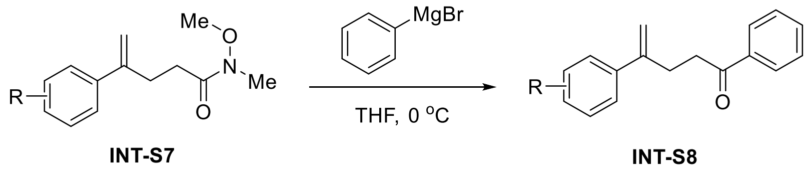

Reactions performed on a 3-7 mmol scale. Weinreb amides were prepared according to Lautens and coworkers (2019).[3] To a solution of weinreb amides **INT-S7** (1 equiv) in THF (1 mL/mmol) at 0 °C was added freshly prepared phenylmagnesium bromide (2 equiv) dropwise (prepared analogously to the Grignard reagent outlined in **GP1**). The reaction was left to stir at 0 °C until starting material was consumed, monitored by TLC. The reaction was quenched with saturated ammonium chloride solution, and extracted with EtOAc (3 x 50 mL). The organic extractions were combined, dried over $MgSO_4$, filtered and concentrated under vacuum to give crude ketones **INT-S8** as yellow oils, used directly in the next step.

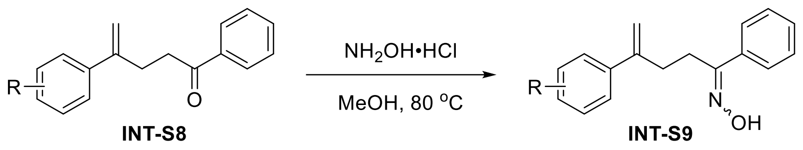

Reaction typically performed on a 2-7 mmol scale. Hydroxylamine-HCl (4 equiv) and sodium acetate (7 equiv) were added to a solution of **INT-S8** in methanol [0.5M] at room temperature. The reaction mixture was refluxed at 80 °C for 4 hours or until the starting material had been consumed, monitored by TLC. The reaction was cooled to room temperature, diluted with EtOAc and washed with brine. Concentration of the organic layer under vacuum gave crude **INT-S9** as white solids, which were triturated with pentanes prior to being used in the next step.

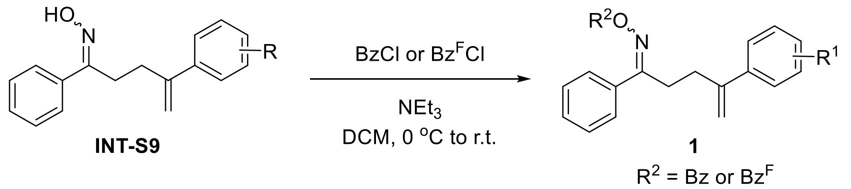

Reaction typically performed on a 2-6 mmol scale. In a flame-dried round-bottom flask under argon atmosphere was added **INT-S9**, anhydrous DCM [0.5M] and anhydrous triethylamine (2 equiv). The solution was cooled to 0 °C and pentafluorobenzoyl chloride (1.2 equiv) or benzoyl chloride (1.2 equiv, synthesis of **1x**) was added dropwise. The reaction was warmed to room temperature, and upon consumption of the starting material (monitored by TLC), quenched with H_2O. DCM was added and the organic layer was washed with brine, dried over $MgSO_4$, filtered and concentrated under vacuum. The crude material was purified by silica gel column chromatography (typically 0% → 50% DCM in pentanes, gradient) to afford purified substrates **1v**, **1w** and **1x**.

General Procedure 4 (GP4): Enantioselective Borylcupration/Cyclization Reaction

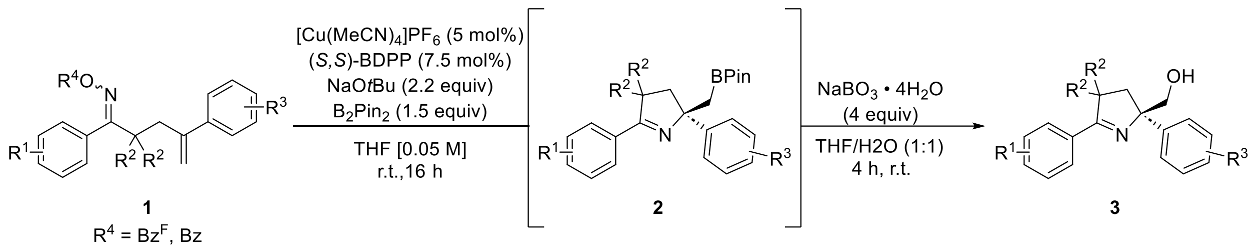

Reactions performed on a 0.2 mmol scale. Three 2-dram vials (one containing a stir bar) were flame-dried and cooled under argon. To the first vial with the stir bar, $[Cu(MeCN)_4]PF_6$ (3.7 mg, 0.01 mmol, 5 mol %), Na*Ot*Bu (42 mg, 0.44 mmol, 2.2 equiv) and (*S,S*)-BDPP (6.6 mg, 0.015 mmol, 7.5 mol %) were added, and the vial was sealed with a septum cap. 1 mL of THF was added to the first vial and the copper, ligand and base were stirred for 5 minutes. To the second vial, B_2pin_2 (114 mg, 0.45 mmol) was added and dissolved in 1.5 mL of THF. After 5 minutes, 1 mL of the B_2pin_2 solution was added to the first vial (0.3 mmol B_2pin_2, 1.5 equiv) and stirred for an additional 5 minutes. To the third vial, alkene-tethered oxime esters **1** (0.22 mmol) were added and dissolved in 2.2 mL of THF. 5 minutes following the addition of B_2pin_2, 2 mL of the solution of **1** was added to the reaction mixture (0.2 mmol, 1 equiv) and left to stir at room temperature for 16

hours. Upon completion, the reactions were diluted with EtOAc (1 mL) and passed through a short plug of silica-gel, eluting with EtOAc.

The filtered reactions were concentrated under vacuum and the crude borylated pyrrolines **2** were re-dissolved in THF (4 mL) and H_2O (4 mL). $NaBO_3 \cdot 4H_2O$ (123 mg, 0.8 mmol, 4 equiv) was then added and the reactions were left to stir for 4 hours at room temperature. Upon completion, the reactions were diluted with EtOAc and washed with H_2O (3 x 5 mL) and brine (1 x 5 mL). The organic layer was dried over $MgSO_4$ and passed through a short pad of celite, and concentrated under vacuum. The crude material was purified by flash column chromatography (typically 0% →35% EtOAc in Pentane, gradient) to give purified pyrrolines **3**.

Racemic mixtures of the products were synthesized in an identical fashion on a 0.1 mmol scale, using 1,3-bis(diphenylphosphino)propane (DPPP) as a ligand.

General Procedure 5 (GP5): Scale-up (1 mmol) Synthesis of Borylated Pyrroline 2p

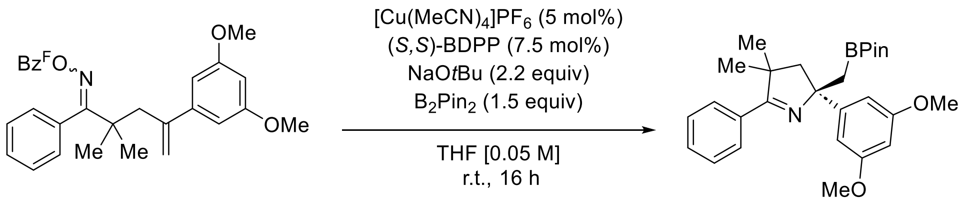

1p **2p**

Reaction performed on a 1.0 mmol scale. A 50 mL round-bottom flask containing a stir bar and two 2-dram vials were flame-dried and cooled under argon. To the round bottom flask, $[Cu(MeCN)_4]PF_6$ (18.6 mg, 0.05 mmol, 5 mol %), NaO*t*Bu (211 mg, 2.2 mmol, 2.2 equiv) and (*S,S*)-BDPP (33 mg, 0.075 mmol, 7.5 mol %) were added, and the flask was sealed with a rubber septum. 5 mL of THF was added to the flask and the copper, ligand and base were stirred for 5 minutes. To the second vial, B_2pin_2 (381 mg, 1.5 mmol, 1.5 equiv) was added and dissolved in 5 mL of THF. After 5 minutes, the B_2pin_2 solution was added to the flask and stirred for an additional 5 minutes. To the third vial, alkene-tethered oxime ester **1p** (533 mg, 1 mmol, 1 equiv) was added and dissolved in 10 mL of THF. 5 minutes following the addition of B_2pin_2, the solution of **1p** was added to the reaction mixture and left to stir at room temperature for 16 hours. Upon completion, the reactions were diluted with EtOAc (5 mL) and passed through a short plug of silica-gel, eluting with EtOAc.

The filtered reaction was washed with H_2O (4 x 30 mL) and brine (1 x 30 mL), and the organic layer was dried over $MgSO_4$, filtered and concentrated to give borylated pyrroline **2o**. The product was further purified by trituration with pentanes, giving pure **2o** as a tan solid (396 mg, 88% yield, >99:1 er).
Racemic mixtures of the products were synthesized in an identical fashion on a 1 mmol scale, using 1,3-bis(diphenylphosphino)propane (DPPP) as a ligand.

General Procedure 6 (GP6): Diastereoselective Reduction of Pyrroline 3p To Prolinol 4

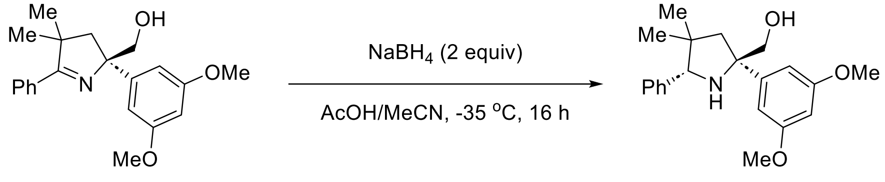

3p **4**

Two 2-dram vials (one containing a stir bar) were flame-dried and cooled under argon. $NaBH_4$ (4.5 mg, 0.12 mmol, 2 equiv) was added to the first vial containing a stir bar, followed by MeCN (0.5 mL). The mixture was cooled to 0 °C in an ice bath, and AcOH (0.2 mL) was added dropwise. The reaction was left to stir at 0 °C for 10 minutes or until no more gas evolution was observed. In the second vial, **3p** (20 mg, 0.059 mmol, 1 equiv) was added and dissolved in 0.5 mL of MeCN (sonication or gentle heating helps fully dissolve the material). The reaction vial was then moved to a cooling bath at -35 °C and stirred for 5 minutes before the addition of the solution of **3p** dropwise. The reaction was left to stir at -35 °C for 8 hours. Upon completion, saturated sodium bicarbonate solution (1 mL) was added at -35 °C and the reaction was stirred at room temperature for 10 minutes. The reaction was diluted with DCM, washed with saturated sodium bicarbonate solution (3 x 5 mL), dried over $MgSO_4$, filtered and concentrated under vacuum. The crude material was purified by column chromatography (0% → 40% EtOAc/Pentanes) to give prolinol derivative **4** as a clear oil (15 mg, 74% yield, >20:1 dr, >99:1 er).

General Procedure 7 (GP7): TEMPO Control Experiment

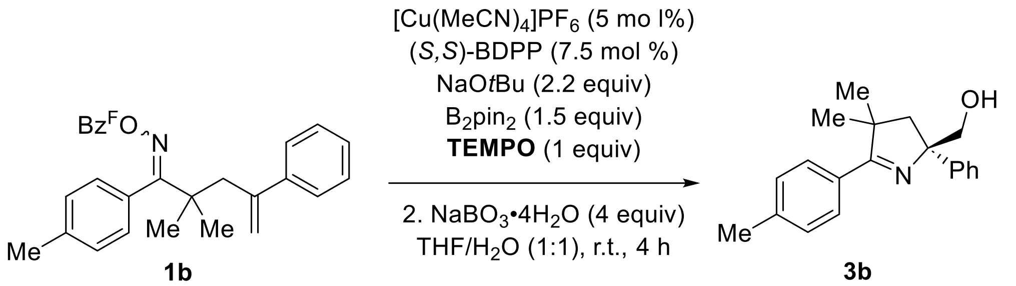

Reaction performed on a 0.1 mmol scale. Three 2-dram vials (one containing a stir bar) were flame-dried and cooled under argon. To the first vial with the stir bar, $[Cu(MeCN)_4]PF_6$ (1.9 mg, 0.005 mmol, 5 mol %), NaO*t*Bu (21 mg, 0.22 mmol, 2.2 equiv) and (*S,S*)-BDPP (3.3 mg, 0.075 mmol, 7.5 mol %) were added, and the vial was sealed with a septum cap. 0.5 mL of THF was added to the first vial and the copper, ligand and base were stirred for 5 minutes. To the second vial, B_2pin_2 (57 mg, 0.225 mmol) was added and dissolved in 0.75 mL of THF. After 5 minutes, 0.5 mL of the B_2pin_2 solution was added to the first vial (0.15 mmol B_2pin_2, 1.5 equiv) and stirred for an additional 5 minutes. To the third vial, alkene-tethered oxime ester **1b** (0.1 mmol) and TEMPO (15.6 mg, 0.1 mmol, 1 equiv) were added and dissolved in 1 mL of THF. 5 minutes following the addition of B_2pin_2, the solution of **1b** and TMEPO was added to the reaction mixture and left to stir at room temperature for 16 hours. Upon completion, the reaction was diluted with EtOAc (1 mL) and passed through a short plug of silica-gel, eluting with EtOAc.

The filtered reaction was concentrated under vacuum and the crude borylated pyrroline **2b** was re-dissolved in THF (2 mL) and H_2O (2 mL). $NaBO_3$•$4H_2O$ (62 mg, 0.4 mmol, 4 equiv) was then added and the reactions were left to stir for 4 hours at room temperature. Upon completion, the reactions were diluted with EtOAc and washed with H_2O (3 x 5 mL) and brine (1 x 5 mL). The organic layer was dried over $MgSO_4$ and passed through a short pad of celite, and concentrated under vacuum. The crude material was purified by flash column chromatography (typically 0% →35% EtOAc in Pentane, gradient) to give purified pyrroline **3b**.

General Procedure 8 (GP8): Synthesis of Cyclic Carbamate 5

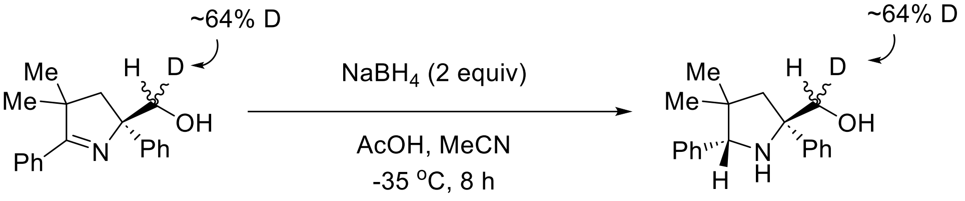

A 2-dram and a 9-dram vial (the 9-dram containing a stir bar) were flame-dried and cooled under argon. $NaBH_4$ (50 mg, 1.3 mmol, 2 equiv) was added to the 9-dram vial containing a stir bar, followed by MeCN (5.5 mL). The mixture was cooled to 0 °C in an ice bath, and AcOH (2.2 mL) was added dropwise. The reaction was left to stir at 0 °C for 10 minutes or until no more gas evolution was observed. In the 2-dram vial, **3a-D** (183 mg, 0.65 mmol, 1 equiv) was added and dissolved in 5.5 mL of MeCN). The 9-dram vial was then moved to a cooling bath at -35 °C and stirred for 5 minutes before the addition of the solution of **3a-D** dropwise. The reaction was left to stir at -35 °C for 8 hours. Upon completion, saturated sodium bicarbonate solution (5 mL) was added at -35 °C and the reaction was stirred at room temperature for 10 minutes or until no more gas evolution was observed. The reaction was diluted with DCM, washed with saturated sodium bicarbonate solution (3 x 5 mL), dried over $MgSO_4$, filtered and concentrated under vacuum. The crude material was purified by column chromatography (0% → 40% EtOAc/Pentanes) to give prolinol derivative **INT-S10** as a beige sap (114 mg, 62% yield (89% yield brsm), >20:1 dr), used in the next step.

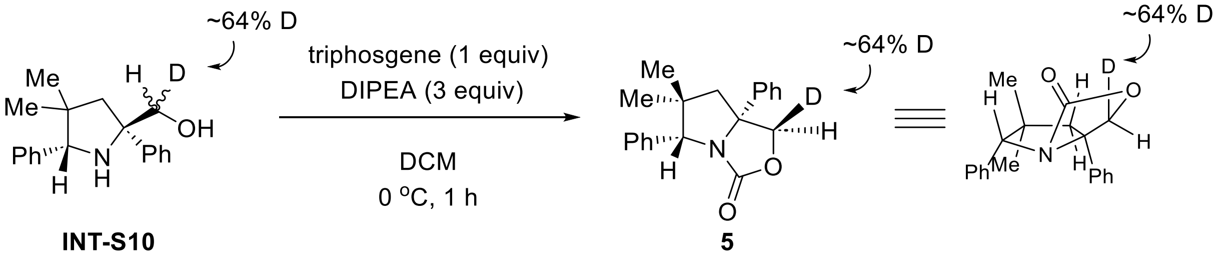

A 2-dram vial containing a stir bar was flame dried and cooled under argon. **INT-S10** (105 mg, 0.37 mmol, 1 equiv) was added to the vial and dissolved in anhydrous DCM (2 mL). The reaction vial was placed in an ice bath and stirred for 5 minutes. DIPEA (0.2 mL, 1.1 mmol, 3 equiv) was added to the reaction followed by triphosgene (109 mg, 0.37 mmol, 1 equiv) in a single portion. The reaction was left to stir at 0 °C for 10 minutes, and then warmed to room temperature for an additional 50 minutes. The reaction was diluted with DCM, washed with saturated sodium

bicarbonate solution (3 x 5 mL), dried over $MgSO_4$, filtered and concentrated under vacuum. The crude residue was triturated with pentanes, giving **5** as a white solid (54 mg, 47% yield, >20:1 dr).

General Procedure 9 (GP9): Synthesis of Dinuclear Ni^{II} Complex from 3s

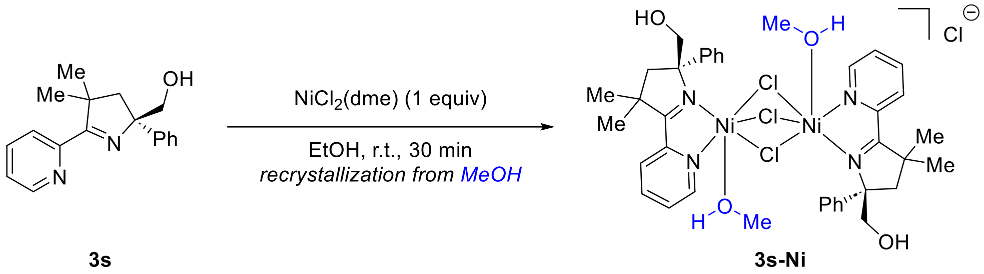

A 2-dram vial containing a stir-bar was flame dried and cooled under argon was added $NiCl_2$(dme) (7.9 mg, 0.036 mmol, 1 equiv). **3r** (10 mg, 0.036 mmol, 1 equiv) was dissolved in anhydrous EtOH (0.5 mL) and added to the reaction vial. The reaction was left to stir at room temperature for 30 minutes, followed by the removal of EtOH under vacuum. The resulting green powder was recrystallized from the slow diffusion of pentanes into a solution of MeOH, yielding green crystals of complex **3s-Ni**.

General Procedure 10 (GP10): Synthesis of Substrates 1k and 1t

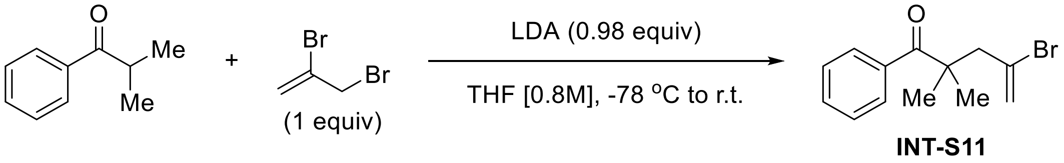

Preparation of LDA: To an ice cooled solution of iPr_2NH (1.98mL, 14.70mmol) in THF (18.8mL) was added *n*-BuLi (5.88mL, 14.70mmol, 2.5M in hexane) and allowed to stir for 30 minutes then used directly.

To a -78 °C solution of LDA (14.70 mmol) in THF (18.8 mL) was added isobutyrophenone (2.25mL, 15mmol) dropwise and allowed to stir for 2h at -78 °C. To the solution was added 2,3-dibromopropene (1.47mL, 15mmol, 80% tech. grade) dropwise at -78 °C and was allowed to stir for 2 hours. The solution was then warmed to room temperature and allowed to stir for 1 hour. The reaction was then quenched with NH_4Cl and extracted with Et_2O, then the combined organic phases were washed with brine, dried over $MgSO_4$, and concentrated under vacuum. The resulting oil was passed through a plug of silica eluting with 20% CH_2Cl_2/Pentane to yield **INT-S11** as a yellow oil contaminated with isobutyrophenone (1.285g, 62:38 **INT-S11:SM**, 31%**)** which was used directly in the next step without further characterization.

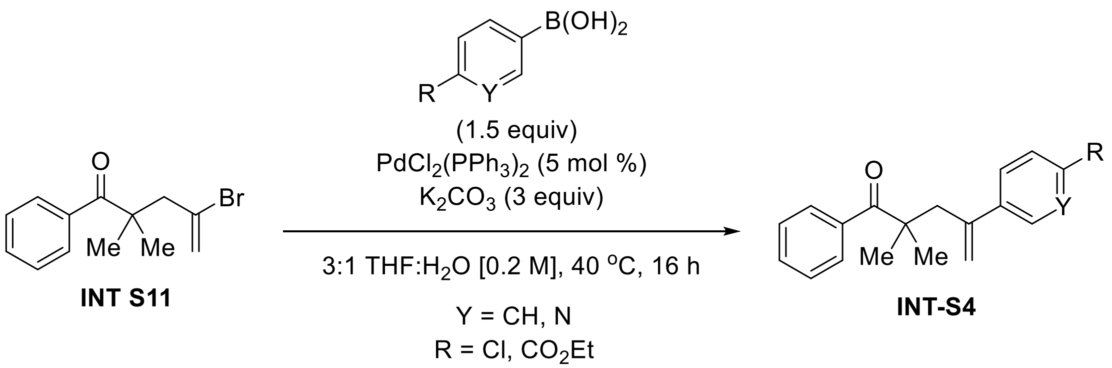

Reactions performed on a 0.85 to 1.3 mmol scale. To a stirred solution of $PdCl_2(PPh_3)_2$ (5 mol %), boronic acid (1.5 equiv) and K_2CO_3 (3 equiv) in 3:1 THF/H_2O [0.2 M] was added **INT-S11** (1 equiv) and heated to 40 °C for 16 hours. The reaction was then cooled to room temperature, diluted with EtOAc, washed with H_2O and brine, before being dried over $MgSO_4$ and concentrated under vacuum. The resulting oils were purified via column chromatography (15% EtOAc/Pentane) to yield ketones **INT-S4** yellow oils, used in the synthesis of substrates **1k** and **1t** as per **GP2**.

Starting Material Characterization

Known Starting Materials

The following Substrates were synthesized according to previous literature reports:

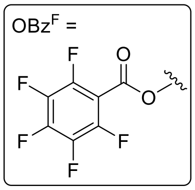

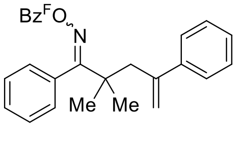
1a

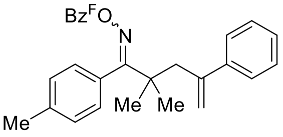
1b

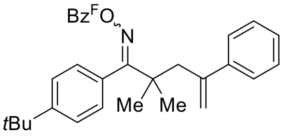
1c

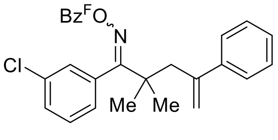
1d

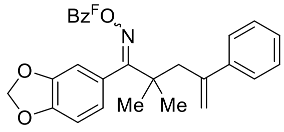
1e

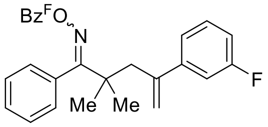
1f

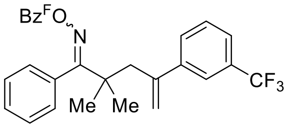
1g

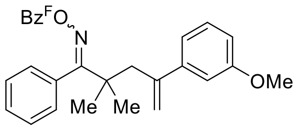
1h

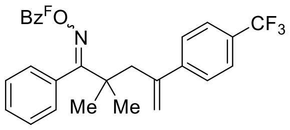
1i

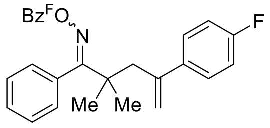
1j

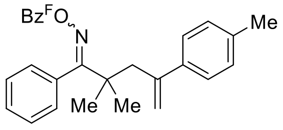
1l

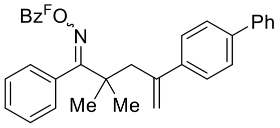
1m

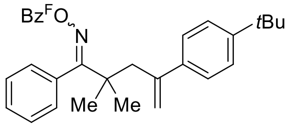
1n

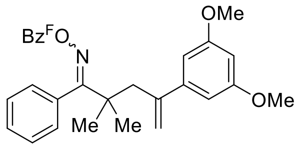
1p

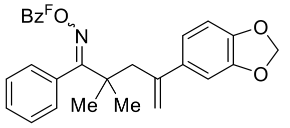
1q

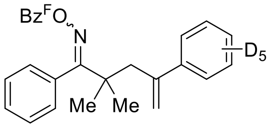
1r

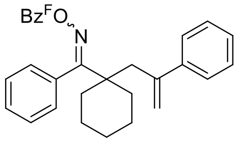
1u

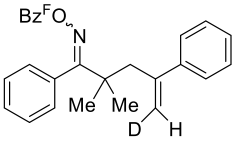
1a-D
64% D

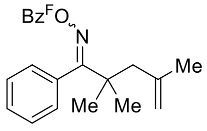
1y

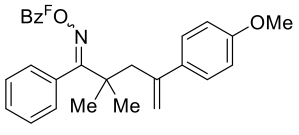
1z

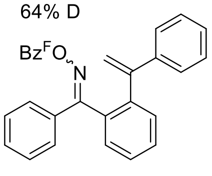
1aa

For the synthesis and characterization of **1a-D**, **1d** and **1p** see Lautens and coworkers (2023).[4]
For the synthesis and characterization of **1a**, **1b**, **1i**, **1j**, **1l**, **1**r, and **1z** see Liang and coworkers (2021).[5]
For the synthesis and characterization of **1c**, **1e**, **1f**, **1g**, **1h**, **1m**, **1n**, **1q**, **1u** and **1aa** see Lautens and coworkers (2023).[6]
For the synthesis and characterization of **1y**, see Wang and Lin (2018)[35]

New Starting Materials

4-(3,5-di-tert-butylphenyl)-2,2-dimethyl-1-phenylpent-4-en-1-one-O-perfluorobenzoyl oxime (1o):

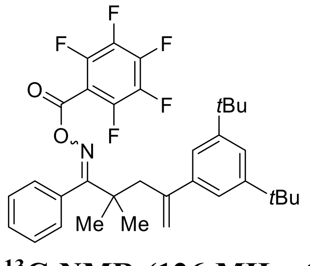

Synthesized according to GP2 on a 2.0 mmol scale from the corresponding oxime. Purified by silica gel column chromatography (0% → 50% DCM/Pentane) to give **1o** as a white solid (521mg, 45% yield).

^{1}H NMR (300 MHz, $CDCl_3$): δ 7.37 – 7.32 (m, 3H), 7.30 (t, *J* = 1.8 Hz, 1H), 7.15 (d, *J* = 1.8 Hz, 2H), 7.04 – 6.97 (m, 2H), 5.37 (d, *J* = 1.7 Hz, 1H), 5.21 (d, *J* = 1.6 Hz, 1H), 2.89 (s, 2H), 1.29 (s, 18H), 1.15 (s, 6H).
^{13}C NMR (126 MHz, $CDCl_3$): δ 177.2, 150.5, 146.4, 142.3, 132.5, 128.6, 128.0, 126.5, 121.3, 120.9, 117.5, 44.8, 42.5, 34.8, 31.5, 26.3. Signals corresponding to the perfluorinated aromatic ring were not resolvable due to weak intensity.
^{19}F NMR (282 MHz, $CDCl_3$): δ -137.5, -148.5, -160.4.
IR (ATR, cm^{-1}): 2965, 2871, 1771, 1654, 1592, 1521, 1506, 1466, 1428, 1362.
HRMS: (DART) m/z: $[M+H]^+$ Calculated for $C_{34}H_{37}NO_2F_5$ 586.2739; Found 586.2739
MP: 88-89 °C

2,2-dimethyl-4-phenyl-1-(pyridin-2-yl)pent-4-en-1-one O-perfluorobenzoyl oxime (1s):

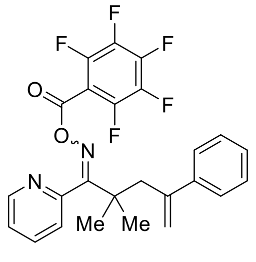

Synthesized according to **GP1** and **GP2** on a 5.3 mmol scale from the corresponding oxime. Purified by silica gel column chromatography (0% → 50% DCM/Pentane) to give **1s** as a white solid (960 mg, 38% yield).

^{1}H NMR (400 MHz, $CDCl_3$): δ 8.66 (ddd, *J* = 4.9, 1.8, 1.0 Hz, 1H), 7.69 (td, *J* = 7.8, 1.8 Hz, 1H), 7.41 – 7.36 (m, 2H), 7.32 – 7.20 (m, 4H), 6.98 (dt, *J* = 7.8, 1.1 Hz, 1H), 5.37 (d, *J* = 1.7 Hz, 1H), 5.23 (s, 1H), 3.05 (s, 2H), 1.14 (s, 6H).
^{13}C NMR (101 MHz, $CDCl_3$): δ 174.0, 152.0, 149.2, 145.5, 143.1, 135.9, 128.2, 127.2, 126.7, 123.5, 123.0, 118.3, 44.5, 42.3, 26.2. Signals corresponding to the perfluorinated aromatic ring were not resolvable due to weak intensity.
^{19}F NMR (376 MHz, $CDCl_3$): -137.3, -148.1, -160.3.
IR (ATR, cm^{-1}): 2979, 1755, 1654, 1588, 1570, 1521, 1505, 1468, 1453, 1426.
HRMS: (DART) m/z: $[M+H]^+$ Calculated for $C_{25}H_{20}N_2O_2F_5$ 475.1440; Found 475.1433
MP: 106-107 °C

1-phenyl-4-(p-tolyl)pent-4-en-1-one O-perfluorobenzoyl oxime (1v):

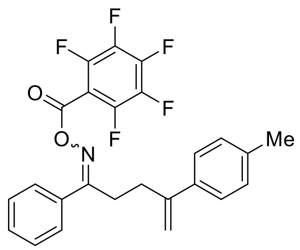

Synthesized according to **GP3** on a 6.6 mmol scale from the corresponding oxime. Purified by silica gel column chromatography (0% → 50% DCM/Pentane) to give **1v** as an off-white solid (2.1 g, 69% yield).

^{1}H NMR (400 MHz, CDCl$_3$): δ 7.75 – 7.69 (m, 2H), 7.53 – 7.40 (m, 3H), 7.23 – 7.19 (m, 2H), 7.02 (d, *J* = 7.6 Hz, 2H), 5.27 (s, 1H), 4.96 (s, 1H), 3.06 (t, *J* = 7.0 Hz, 2H), 2.74 (t, 2H), 2.29 (s, 3H).
^{13}C NMR (101 MHz, CDCl$_3$): δ 168.1, 145.8, 137.5, 136.9, 132.9, 131.1, 128.9, 128.8, 127.6, 125.8, 113.1, 32.2, 27.8, 20.9. Signals corresponding to the perfluorinated aromatic ring were not resolvable due to weak intensity.
^{19}F NMR (376 MHz, CDCl$_3$): δ -136.9, -148.2, -160.1.
IR (ATR, cm^{-1}): 1757, 1653, 1522, 1495, 1444, 1418, 1355, 1324, 1190, 1096.
HRMS: (DART) m/z: [M+H]$^+$ Calculated for $C_{25}H_{19}NO_2F_5$ 460.1331; Found 460.1336
MP: 97-99 °C

4-(4-fluorophenyl)-1-phenylpent-4-en-1-one O-perfluorobenzoyl oxime (1w):

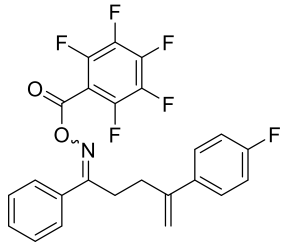

Synthesized according to **GP3** on a 4.1 mmol scale from the corresponding oxime. Purified by silica gel column chromatography (0% → 50% DCM/Pentane) to give **1w** as an off-white solid (930 mg, 49% yield).

^{1}H NMR (400 MHz, CDCl$_3$): δ 7.72 – 7.66 (m, 2H), 7.53 – 7.40 (m, 3H), 7.30 – 7.23 (m, 2H), 6.90 (t, *J* = 8.7 Hz, 2H), 5.24 (s, 1H), 4.99 (s, 1H), 3.06 (t, *J* = 7.7 Hz, 2H), 2.74 (t, *J* = 7.7 Hz, 2H).
^{13}C NMR (101 MHz, CDCl$_3$): δ 167.9, 162.3 (d, *J* = 247.0 Hz), 156.3, 145.0, 136.0 (d, *J* = 3.4 Hz), 132.8, 131.2, 128.2 (d, *J* = 133.6 Hz), 127.6 (d, *J* = 8.0 Hz), 115.0 (d, *J* = 21.3 Hz), 113.9 (d, *J* = 1.3 Hz), 32.3, 27.5. Signals corresponding to the perfluorinated aromatic ring were not resolvable due to weak intensity.
^{19}F NMR (376 MHz, CDCl$_3$): -114.7, -137.1, -147.5, -159.9.
IR (ATR, cm^{-1}): 1756, 1648, 1600, 1566, 1527, 1508, 1490, 1441, 1406, 1320.
HRMS: (DART) m/z: [M+H]$^+$ Calculated for $C_{24}H_{16}NO_2F_6$ 464.1080; Found 464.1077
MP: 122-124 °C

1,4-diphenylpent-4-en-1-one O-benzoyl oxime (1x):

Note: Substrate **1x** was isolated and used as a ~10:1 mixture of *Z/E* oxime ester isomers. NMR data is representative of the major isomer.

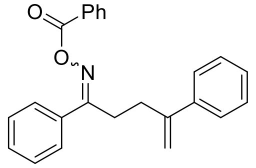

Synthesized according to **GP3** using benzoyl chloride on a 1.9 mmol scale from the corresponding oxime. Purified by silica gel column chromatography (0% → 50% DCM/Pentane) to give **1x** as a yellow solid (254 mg, 38% yield).

^{1}H NMR (600 MHz, CDCl$_3$): δ 8.02 – 7.96 (m, 2H), 7.80 – 7.76 (m, 2H), 7.64 – 7.58 (m, 1H), 7.49 – 7.42 (m, 5H), 7.41 – 7.37 (m, 2H), 7.30 – 7.25 (m, 3H), 5.34 (d, *J* = 1.1 Hz, 1H), 5.11 (d, *J* = 1.2 Hz, 1H), 3.18 – 3.10 (m, 2H), 2.89 – 2.83 (m, 2H).

^{13}C NMR (126 MHz, CDCl$_3$): δ 166.7, 163.8, 146.7, 140.0, 133.8, 133.3, 130.7, 129.7, 129.1, 128.7, 128.6, 128.5, 127.8, 113.7, 32.5, 27.8.
IR (ATR, cm^{-1}): 1744, 1616, 1600, 1495, 1442, 1346, 1313, 1255, 1175, 1080.
HRMS: (DART) m/z: [M+H]$^+$ Calculated for $C_{24}H_{22}NO_2$ 356.1645; Found 356.1648
MP: 85-87 °C

Methyl 4-(4,4-dimethyl-5-(((perfluorobenzoyl)oxy)imino)-5-phenylpent-1-en-2-yl)benzoate (1k)

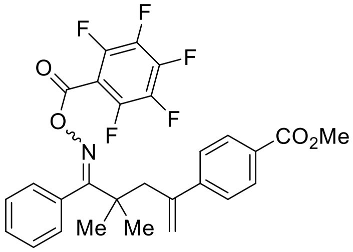

Synthesized according to **GP10** and **GP2** on a 0.82 mmol scale from the corresponding oxime. Purified by silica gel column chromatography (0% → 50% DCM/Pentane) to give **1k** as an off-white solid (339 mg, 76% yield).

^{1}H NMR (400 MHz, CDCl$_3$): δ 7.99 – 7.94 (m, 2H), 7.46 – 7.41 (m, 2H), 7.40 – 7.33 (m, 3H), 7.02 – 6.95 (m, 2H), 5.47 (d, *J* = 1.4 Hz, 1H), 5.31 (d, *J* = 1.3 Hz, 1H), 3.90 (s, 3H), 2.96 (d, *J* = 0.9 Hz, 2H), 1.12 (s, 6H).
^{13}C NMR (101 MHz, CDCl$_3$): δ 176.4, 166.9, 147.7, 144.8, 132.3, 129.7, 129.0, 128.7, 128.1, 126.6, 126.3, 120.0, 52.1, 44.3, 42.4, 26.3.
^{19}F NMR (470 MHz, CDCl$_3$): δ -137.5, -148.3, -160.3
IR (ATR, cm^{-1}): 2974, 1760, 1720, 1651, 1607, 1522, 1497, 1436, 1276, 1189.
HRMS: (DART) m/z: [M+H]$^+$ Calculated for $C_{28}H_{23}NO_4F_5$ 532.1542; Found 532.1538
MP: 86-89 °C

4-(6-chloropyridin-3-yl)-2,2-dimethyl-1-phenylpent-4-en-1-one O-perfluorobenzoyl oxime (1t)

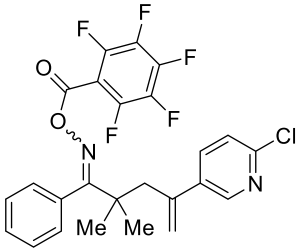

Synthesized according to **GP10** and **GP2** on a 0.85 mmol scale from the corresponding oxime. Purified by silica gel column chromatography (0% → 90% DCM/Pentane) to give **1t** as an off-white solid (338 mg, 78% yield).

^{1}H NMR (500 MHz, CDCl$_3$): δ 8.39 (dd, *J* = 2.6, 0.7 Hz, 1H), 7.67 (dd, *J* = 8.3, 2.6 Hz, 1H), 7.39 – 7.34 (m, 3H), 7.28 – 7.24 (m, 2H), 7.00 – 6.94 (m, 2H), 5.45 (d, *J* = 1.1 Hz, 1H), 5.36 (d, *J* = 1.0 Hz, 1H), 2.94 (d, *J* = 1.0 Hz, 2H), 1.14 (s, 6H).
^{13}C NMR (126 MHz, CDCl$_3$): δ 175.9, 150.2, 147.5, 141.3, 137.5, 136.9, 132.1, 128.8, 128.1, 126.2, 123.7, 120.7, 44.1, 42.3, 26.5.
^{19}F NMR (470 MHz, CDCl$_3$): δ -137.5, -148.1, -160.2
IR (ATR, cm^{-1}): 3057, 2973, 1756, 1651, 1579, 1522, 1496, 1459, 1324, 1188.
HRMS: (DART) m/z: [M+H]$^+$ Calculated for $C_{25}H_{19}N_2O_2F_5Cl$ 509.1050; Found 509.1052
MP: 82-85 °C

Unsuccessful Substrates

The following substrates did not engage in the asymmetric borylation/cyclization reaction:

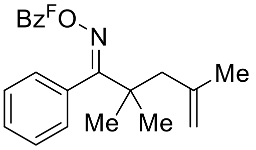

1y

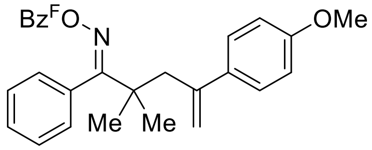

1z

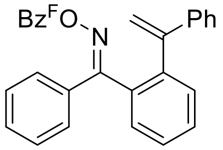

1aa

Product Characterization

(R)-(4,4-dimethyl-2,5-diphenyl-3,4-dihydro-2H-pyrrol-2-yl)methanol (3a):

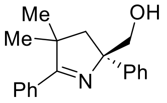

Synthesized according to **GP4** on a 0.2 mmol scale. Purified by silica gel column chromatography (0% → 40% EtOAc/Pentane) to give **3a** as a white solid (48.6 mg, 87% yield, 96.5:3.5 er).

^{1}H NMR (500 MHz, CDCl$_3$): δ 7.77 – 7.73 (m, 2H), 7.47 – 7.39 (m, 5H), 7.35 – 7.30 (m, 2H), 7.25 – 7.20 (m, 1H), 3.95 (d, *J* = 11.3 Hz, 1H), 3.65 – 3.58 (m, 1H), 3.03 (s, 1H), 2.34 (ABq, *J* = 12.8 Hz, 2H), 1.36 (s, 3H), 1.07 (s, 3H).
^{13}C NMR (126 MHz, CDCl$_3$): δ 181.3, 146.1, 134.6, 129.7, 128.3, 128.3, 128.2, 126.6, 126.0, 78.7, 70.9, 51.7, 49.5, 27.6, 27.6.
IR (ATR, cm^{-1}): 3150 (br), 2958, 2934, 2865, 2843, 1599, 1570, 1490, 1444, 1363.
HRMS: (DART) m/z: [M+H]$^+$ Calculated for $C_{19}H_{22}NO$ 280.1696; Found 280.1694
MP: 56-57 °C
$[\alpha]^D_{20}$: (*c* = 0.48, CH_2Cl_2) +145.8
HPLC: IA, 1 mL/min, 5% IPA/Hexane

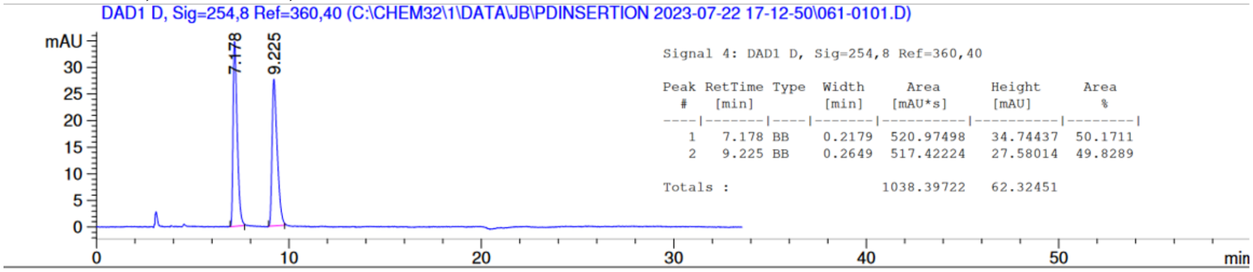

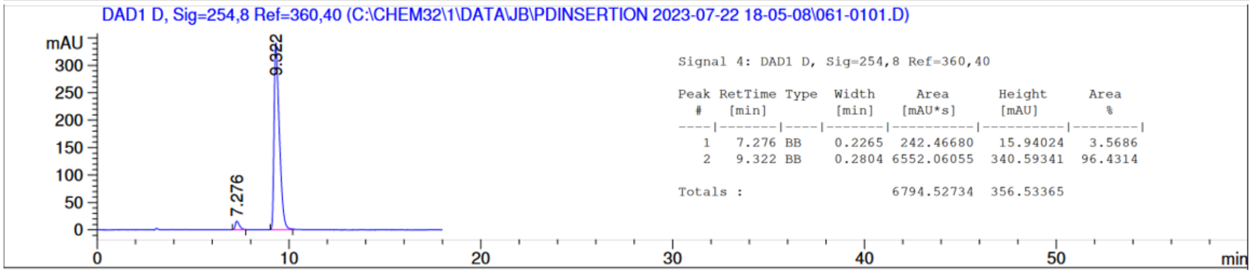

(R)-(4,4-dimethyl-2-phenyl-5-(p-tolyl)-3,4-dihydro-2H-pyrrol-2-yl)methanol (3b):

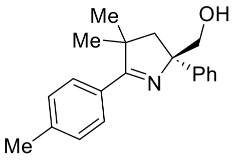

Synthesized according to **GP4** on a 0.2 mmol scale. Purified by silica gel column chromatography (0% → 40% EtOAc/Pentane) to give **3b** as a white solid (45.7 mg, 78% yield, 96:4 er).

^{1}H NMR (500 MHz, CDCl$_3$): δ 7.72 – 7.68 (m, 2H), 7.44 – 7.40 (m, 2H), 7.35 – 7.29 (m, 2H), 7.25 – 7.20 (m, 3H), 3.94 (d, *J* = 11.2 Hz, 1H), 3.63 (d, *J* = 11.3 Hz, 1H), 2.67 (s (br), 1H), 2.45 (d, *J* = 12.8 Hz, 1H), 2.40 (s, 3H), 2.20 (d, *J* = 12.7 Hz, 1H), 1.39 (s, 3H), 1.09 (s, 3H).
^{13}C NMR (126 MHz, CDCl$_3$): δ 181.0, 146.2, 139.9, 131.6, 128.9, 128.3, 128.3, 126.6, 126.0, 78.4, 71.0, 51.6, 49.7, 27.8 – 27.5 (m), 21.4.
IR (ATR, cm^{-1}): 3175 (br), 3064, 3030, 2956, 2928, 2857, 1600, 1564, 1488, 1446.

HRMS: (DART) m/z: $[M+H]^+$ Calculated for $C_{20}H_{24}NO$ 294.1852; Found 294.1859
MP: 77-79 °C
$[\alpha]_{20}^{D}$**:** (c = 0.26, CH_2Cl_2) +176.9
HPLC: IA, 1 mL/min, 5% IPA/Hexane

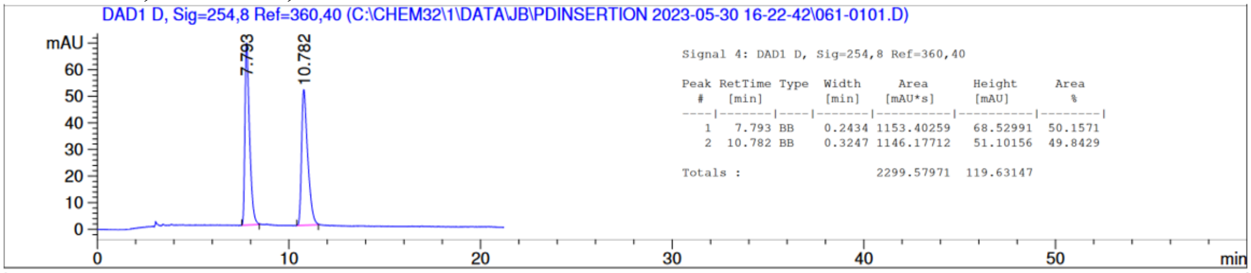

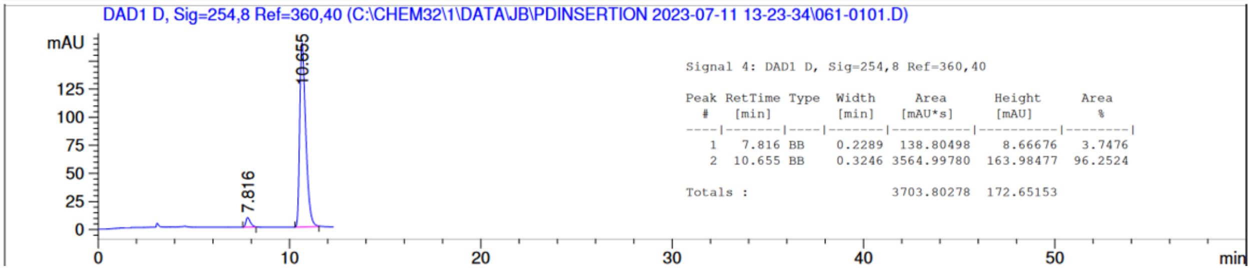

(R)-(5-(4-(tert-butyl)phenyl)-4,4-dimethyl-2-phenyl-3,4-dihydro-2H-pyrrol-2-yl)methanol (3c):

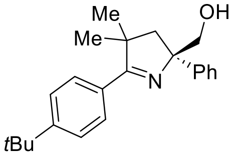

Synthesized according to **GP4** on a 0.2 mmol scale. Purified by silica gel column chromatography (0% → 40% EtOAc/Pentane) to give **3c** as a white solid (56.2 mg, 84% yield, 97:3 er).

^{1}H NMR (500 MHz, $CDCl_3$): δ 7.77 – 7.73 (m, 2H), 7.46 – 7.41 (m, 4H), 7.32 (m, 2H), 7.24 – 7.20 (m, 1H), 3.94 (d, J = 11.3 Hz, 1H), 3.63 (d, J = 11.3 Hz, 1H), 2.73 (s, 1H), 2.32i (ABq, J = 12.7 Hz, 2H), 1.39 (s, 3H), 1.36 (s, 9H), 1.09 (s, 3H).
^{13}C NMR (126 MHz, $CDCl_3$): δ 180.8, 153.0, 146.2, 131.5, 128.2, 128.2, 126.6, 126.0, 125.2, 78.4, 71.0, 51.6, 49.8, 34.8, 31.2, 27.8, 27.7.
IR (ATR, cm^{-1}): 3204 (br), 2960, 2866, 1606, 1558, 1445, 1363, 1317, 1268, 1180.
HRMS: (DART) m/z: $[M+H]^+$ Calculated for $C_{23}H_{30}NO$ 336.2322; Found 336.2318
MP: 51-53 °C
$[\alpha]_{20}^{D}$**:** (c = 0.26, CH_2Cl_2) +107.7
HPLC: IA, 1 mL/min, 5% IPA/Hexane

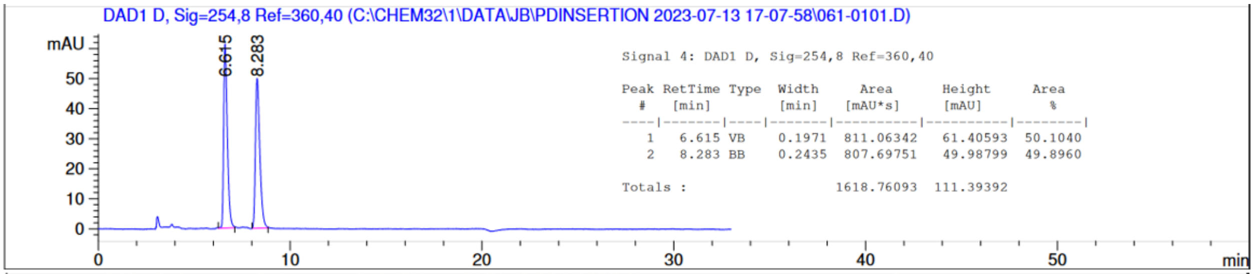

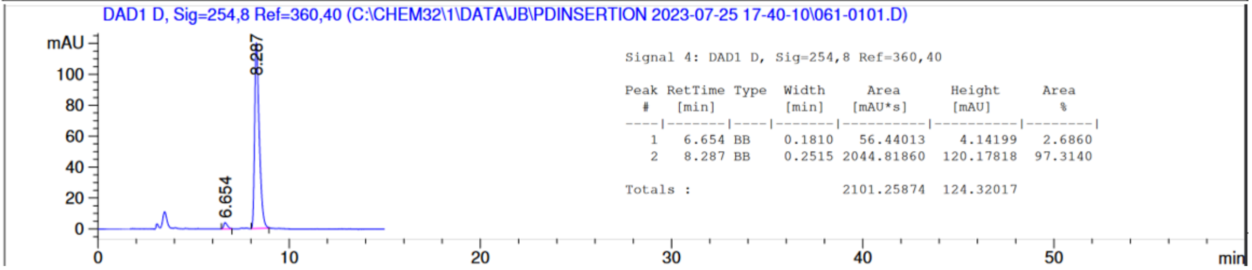

(R)-(5-(3-chlorophenyl)-4,4-dimethyl-2-phenyl-3,4-dihydro-2H-pyrrol-2-yl)methanol (3d):

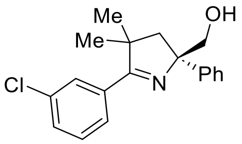

Synthesized according to **GP4** on a 0.2 mmol scale. Purified by silica gel column chromatography (0% → 40% EtOAc/Pentane) to give **3d** as a white solid (57.7 mg, 92% yield, 95:5 er).

^{1}H NMR (500 MHz, CDCl$_3$): δ 7.78 (t, *J* = 1.8 Hz, 1H), 7.67 – 7.62 (m, 1H), 7.44 – 7.39 (m, 3H), 7.38 – 7.36 (m, 1H), 7.36 – 7.31 (m, 2H), 7.26 – 7.22 (m, 1H), 3.97 – 3.90 (m, 1H), 3.64 (dd, *J* = 11.2, 6.9 Hz, 1H), 2.52 – 2.44 (m, 2H), 2.23 (d, *J* = 12.8 Hz, 1H), 1.39 (s, 3H), 1.09 (s, 3H).
^{13}C NMR (126 MHz, CDCl$_3$): δ 179.9, 145.7, 136.3, 134.3, 129.8, 129.6, 128.4, 126.8, 126.4, 126.0, 78.8, 71.0, 51.7, 49.5, 27.5.
IR (ATR, cm^{-1}): 3190 (br), 2957, 2934, 2852, 1611, 1563, 1490, 1465, 1446, 1362.
HRMS: (DART) m/z: $[M+H]^+$ Calculated for $C_{19}H_{21}NOCl$ 314.1306; Found 314.1303. Relative intensity of peaks 314.13 : 316.13 = 3:1.
MP: 93-95 °C
$[\alpha]^{D}_{20}$**:** (*c* = 0.28, CH_2Cl_2) +150.0
HPLC: IA, 1 mL/min, 5% IPA/Hexane

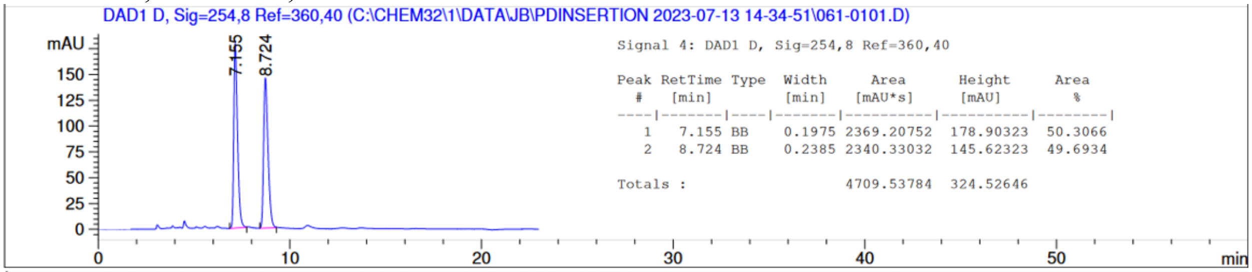

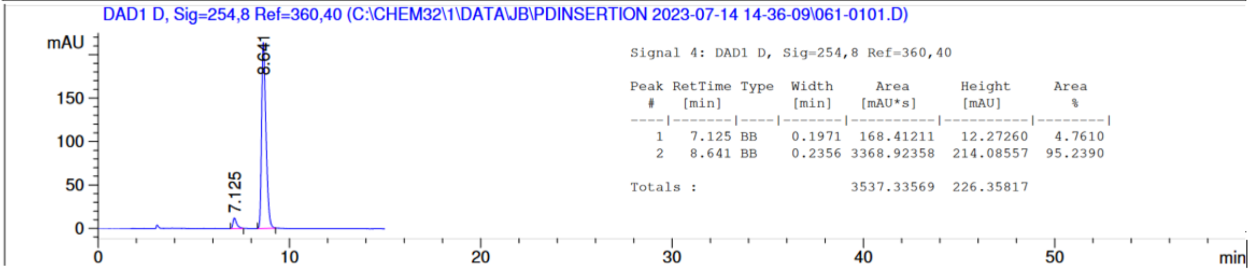

(R)-(5-(benzo[d][1,3]dioxol-5-yl)-4,4-dimethyl-2-phenyl-3,4-dihydro-2H-pyrrol-2-yl)methanol (3e):

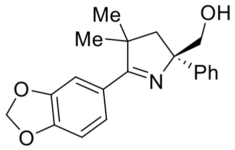

Synthesized according to **GP4** on a 0.2 mmol scale. Purified by silica gel column chromatography (0% → 40% EtOAc/Pentane) to give **3e** as a light yellow, sticky glass (54.2 mg, 84% yield, 96.5:3.5 er).

^{1}H NMR (500 MHz, $CDCl_3$): δ 7.42 – 7.38 (m, 3H), 7.35 – 7.30 (m, 3H), 7.24 – 7.20 (m, 1H), 6.84 (d, J = 8.1 Hz, 1H), 6.03 – 5.99 (m, 2H), 3.92 (d, J = 11.2 Hz, 1H), 3.63 (d, J = 11.2 Hz, 1H), 2.53 – 2.43 (m, 2H), 2.21 (d, J = 12.7 Hz, 1H), 1.42 (s, 3H), 1.10 (s, 3H).
^{13}C NMR (126 MHz, $CDCl_3$): δ 179.9, 149.0, 147.6, 146.1, 128.4, 128.3, 126.6, 126.0, 122.7, 108.9, 107.9, 101.3, 78.1, 71.1, 51.4, 50.0, 27.9 (d, J = 1.2 Hz), 27.8.
IR (ATR, cm^{-1}): 3206 (br), 2961, 2868, 1735, 1592, 1503, 1489, 1443, 1344, 1302.
HRMS: (DART) m/z: $[M+H]^+$ Calculated for $C_{20}H_{22}NO_3$ 324.1594; Found 324.1601
MP: 35-37 °C
$[\alpha]_{20}^{D}$**:** (c = 0.32, CH_2Cl_2) +175.0
HPLC: IA, 1 mL/min, 5% IPA/Hexane

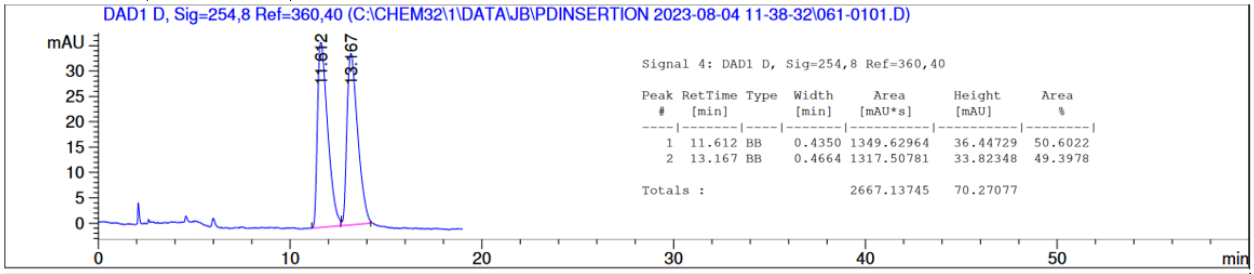

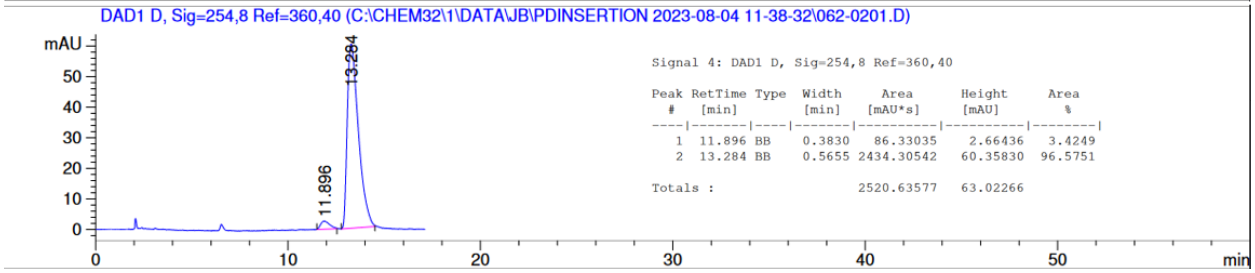

(R)-(2-(3-fluorophenyl)-4,4-dimethyl-5-phenyl-3,4-dihydro-2H-pyrrol-2-yl)methanol (3f):

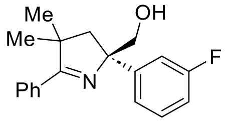

Synthesized according to **GP4** on a 0.2 mmol scale. Purified by silica gel column chromatography (0% → 40% EtOAc/Pentane) to give **3f** as a white solid (54.7 mg, 92% yield, 95.5:4.5 er).

^{1}H NMR (500 MHz, $CDCl_3$): δ 7.76 – 7.72 (m, 2H), 7.48 – 7.40 (m, 3H), 7.29 (m, 1H), 7.20 – 7.13 (m, 2H), 6.92 (m, 1H), 3.91 (d, J = 11.4 Hz, 1H), 3.60 (d, J = 11.4 Hz, 1H), 3.14 (s, 1H), 2.30 (ABq, J = 12.8 Hz, 2H), 1.35 (s, 3H), 1.08 (s, 3H).
^{13}C NMR (126 MHz, $CDCl_3$): δ 181.7, 163.8, 161.8, 148.8 (d, J = 6.7 Hz), 134.3, 129.9, 129.8 (d, J = 8.3 Hz), 128.3 (d, J = 5.4 Hz), 121.6 (d, J = 2.8 Hz), 113.5 (d, J = 21.1 Hz), 113.2 (d, J = 22.4 Hz), 78.4 (d, J = 1.8 Hz), 70.7, 51.7, 49.6, 27.6, 27.5.
^{19}F NMR (376 MHz, $CDCl_3$): δ -112.8.
IR (ATR, cm^{-1}): 3184 (br), 2963, 2936, 2867, 1730, 1612, 1583, 1483, 1442, 1362.

HRMS: (DART) m/z: $[M+H]^+$ Calculated for $C_{19}H_{21}NOF$ 298.1602; Found 298.1600
MP: 47-50 °C
$[\alpha]^D_{20}$**:** (c = 0.325, CH_2Cl_2) +129.2
HPLC: IA, 1 mL/min, 5% IPA/Hexane

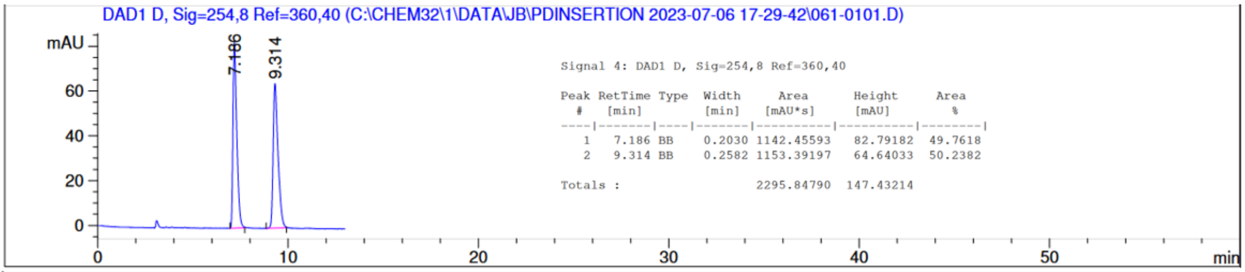

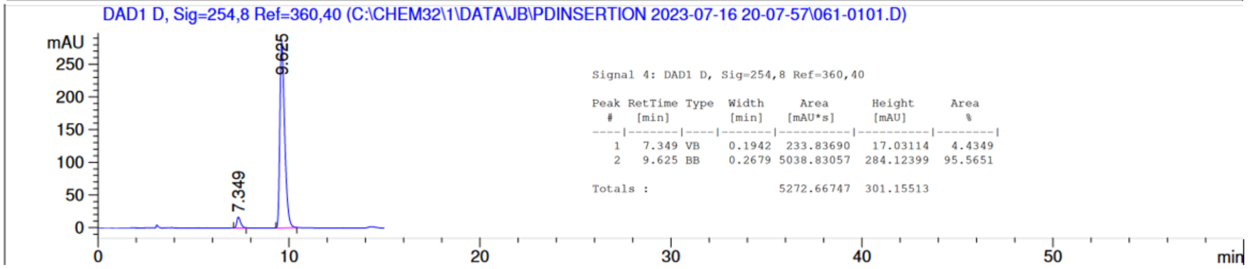

(R)-(4,4-dimethyl-5-phenyl-2-(3-(trifluoromethyl)phenyl)-3,4-dihydro-2H-pyrrol-2-yl)methanol (3g):

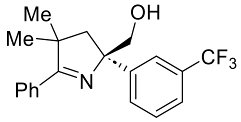

Synthesized according to **GP4** on a 0.2 mmol scale. Purified by silica gel column chromatography (0% → 40% EtOAc/Pentane) to give **3g** as a white solid (55.6 mg, 80% yield, 95:5 er).

^{1}H NMR (500 MHz, $CDCl_3$): δ 7.77 – 7.74 (m, 2H), 7.69 (m, 1H), 7.66 – 7.61 (m, 1H), 7.50 (m, 1H), 7.48 – 7.41 (m, 4H), 3.91 (d, J = 11.3 Hz, 1H), 3.63 (d, J = 11.4 Hz, 1H), 2.85 (s, 1H), 2.34 (ABq, J = 12.8 Hz, 2H), 1.39 (s, 3H), 1.09 (s, 3H).
^{13}C NMR (126 MHz, $CDCl_3$): δ 181.9, 147.2, 134.2, 130.6 (q, J = 31.9 Hz), 130.0, 129.5 (d, J = 1.4 Hz), 128.7, 128.3, 128.3, 124.2 (q, J = 272.4 Hz), 123.6 (q, J = 3.8 Hz), 122.8 (q, J = 3.8 Hz), 78.5, 71.0, 51.9, 49.6, 27.7, 27.6.
^{19}F NMR (376 MHz, $CDCl_3$): δ -62.4.
IR (ATR, cm^{-1}): 3137 (br), 2968, 2959, 2938, 2848, 1738, 1605, 1572, 1432, 1366.
HRMS: (DART) m/z: $[M+H]^+$ Calculated for $C_{20}H_{21}NOF_3$ 348.1569; Found 348.1575
MP: 54-55 °C
$[\alpha]^D_{20}$**:** (c = 0.325, CH_2Cl_2) +116.9
HPLC: IA, 1 mL/min, 5% IPA/Hexane

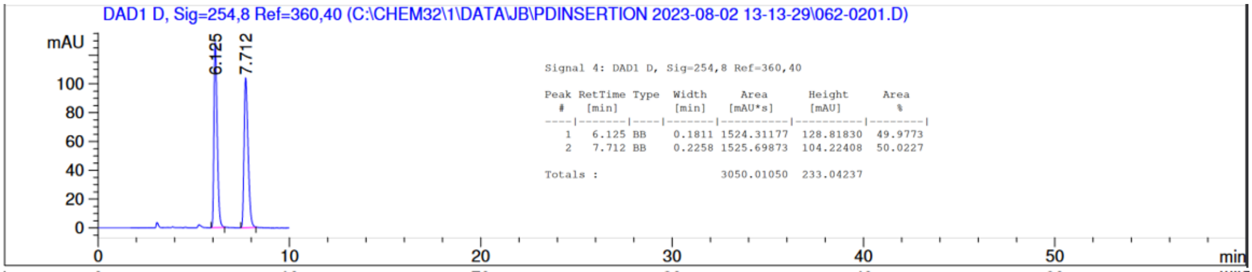

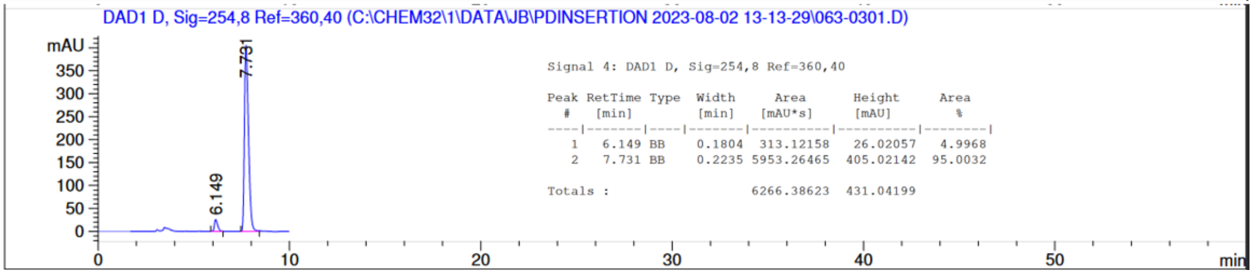

(R)-(2-(3-methoxyphenyl)-4,4-dimethyl-5-phenyl-3,4-dihydro-2H-pyrrol-2-yl)methanol (3h):

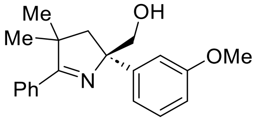

Synthesized according to **GP4** on a 0.2 mmol scale. Purified by silica gel column chromatography (0% → 40% EtOAc/Pentane) to give **3h** as a white solid (51.4 mg, 83% yield, 96.5:3.5 er).

^{1}H NMR (500 MHz, $CDCl_3$): δ 7.76 – 7.73 (m, 2H), 7.46 – 7.40 (m, 3H), 7.27 – 7.22 (m, 1H), 7.04 – 6.98 (m, 2H), 6.78 (ddd, J = 8.2, 2.6, 0.9 Hz, 1H), 3.98 – 3.90 (m, 1H), 3.80 (s, 3H), 3.65 – 3.59 (m, 1H), 2.77 (s, 1H), 2.33 (ABq, J = 12.7 Hz, 2H), 1.37 (s, 3H), 1.09 (s, 3H).
^{13}C NMR (126 MHz, $CDCl_3$): δ 181.4, 159.5, 147.8, 134.5, 129.7, 129.3, 128.3, 128.2, 118.4, 112.2, 111.7, 78.6, 71.0, 55.2, 51.7, 49.5, 27.6.
IR (ATR, cm^{-1}): 3149 (br), 2962, 2861, 2833, 1605, 1578, 1484, 1428, 1309, 1290.
HRMS: (DART) m/z: $[M+H]^+$ Calculated for $C_{20}H_{24}NO_2$ 310.1802; Found 310.1795
MP: 65-66 °C
$[\alpha]_{20}^{D}$: (c = 0.485, CH_2Cl_2) +127.8
HPLC: IA, 1 mL/min, 5% IPA/Hexane

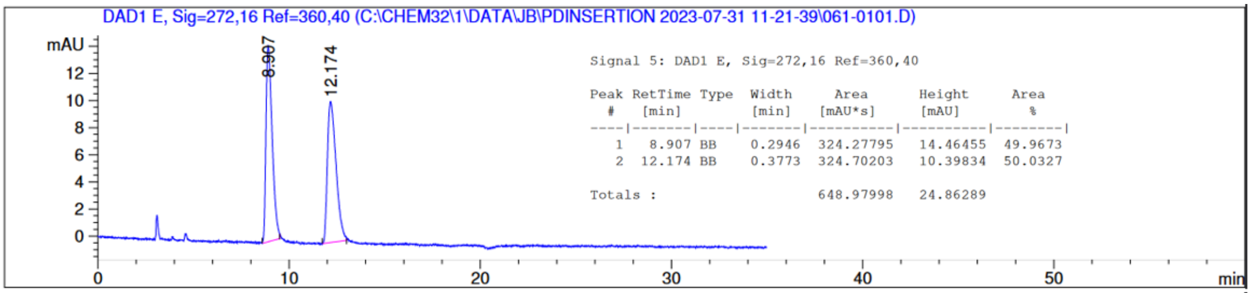

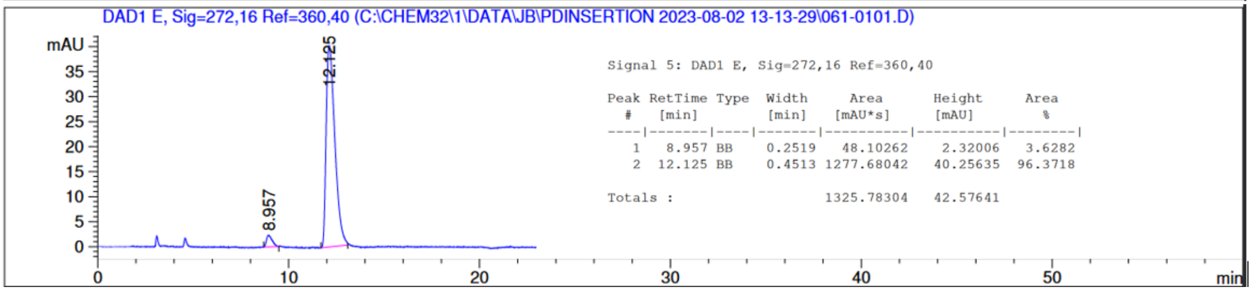

(R)-(4,4-dimethyl-5-phenyl-2-(4-(trifluoromethyl)phenyl)-3,4-dihydro-2H-pyrrol-2-yl)methanol (3i):

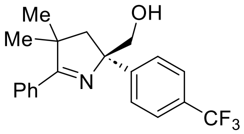

Synthesized according to **GP4** on a 0.2 mmol scale. Purified by silica gel column chromatography (0% → 40% EtOAc/Pentane) to give **3i** as a white solid (59.7 mg, 86% yield, 92.5:7.5 er).

^{1}H NMR (500 MHz, $CDCl_3$): δ 7.77 – 7.72 (m, 2H), 7.60 – 7.56 (m, 2H), 7.56 – 7.52 (m, 2H), 7.50 – 7.41 (m, 3H), 3.91 (d, *J* = 11.4 Hz, 1H), 3.62 (d, *J* = 11.4 Hz, 1H), 3.09 (s, 1H), 2.32 (ABq, *J* = 12.8 Hz, 2H), 1.37 (s, 3H), 1.07 (s, 3H).
^{13}C NMR (126 MHz, $CDCl_3$): δ 181.9, 150.2 – 150.2 (m), 134.2, 130.0, 128.9 (q, *J* = 32.4 Hz), 128.3, 128.3, 126.4, 125.2 (q, *J* = 3.7 Hz), 124.8 (q, *J* = 272.0 Hz), 78.6, 70.8, 51.8, 49.6, 27.7, 27.5.
^{19}F NMR (376 MHz, $CDCl_3$): δ -62.4.
IR (ATR, cm^{-1}): 3199 (br), 2931, 2855, 1617, 1573, 1468, 1444, 1411, 1364, 1324.
HRMS: (DART) m/z: $[M+H]^+$ Calculated for $C_{20}H_{21}NOF_3$ 348.1570; Found 348.1573
MP: 64-65 °C
$[\alpha]_{20}^{D}$: (*c* = 0.29, CH_2Cl_2) +110.3
HPLC: IA, 1 mL/min, 5% IPA/Hexane

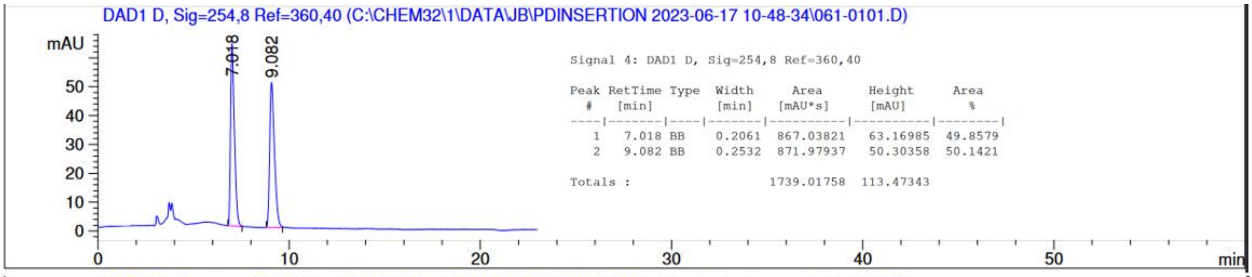

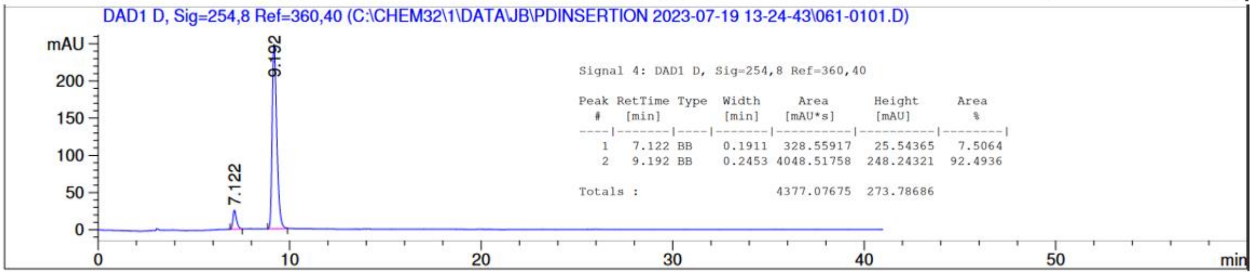

(R)-(2-(4-fluorophenyl)-4,4-dimethyl-5-phenyl-3,4-dihydro-2H-pyrrol-2-yl)methanol (3j):

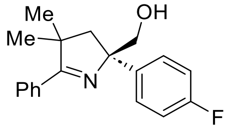

Synthesized according to **GP4** on a 0.2 mmol scale. Purified by silica gel column chromatography (0% → 40% EtOAc/Pentane) to give **3j** as a white solid (57.0 mg, 96% yield, 95.5:4.5 er).

^{1}H NMR (500 MHz, $CDCl_3$): δ 7.74 – 7.69 (m, 2H), 7.46 – 7.35 (m, 5H), 7.04 – 6.96 (m, 2H), 3.89 (d, J = 11.4 Hz, 1H), 3.56 (d, J = 11.4 Hz, 1H), 3.44 (s, 1H), 2.29 (ABq, J = 12.7 Hz, 2H), 1.33 (s, 3H), 1.05 (s, 3H).
^{13}C NMR (126 MHz, $CDCl_3$): δ 181.5, 161.6 (d, J = 244.9 Hz), 141.8 (d, J = 3.2 Hz), 134.3, 129.8, 128.3, 128.2, 127.6 (d, J = 7.8 Hz), 115.0 (d, J = 21.1 Hz), 78.3, 70.8, 51.7, 49.5, 27.5.
^{19}F NMR (376 MHz, $CDCl_3$): δ -116.6.
IR (ATR, cm^{-1}): 3215 (br), 3051, 2966, 2926, 2869, 2848, 1594, 1570, 1507, 1444.
HRMS: (DART) m/z: $[M+H]^+$ Calculated for $C_{19}H_{21}NOF$ 298.1602; Found 298.1608
MP: 58-60 °C
$[\alpha]_{20}^{D}$: (c = 0.39, CH_2Cl_2) +133.3
HPLC: IA, 1 mL/min, 5% IPA/Hexane

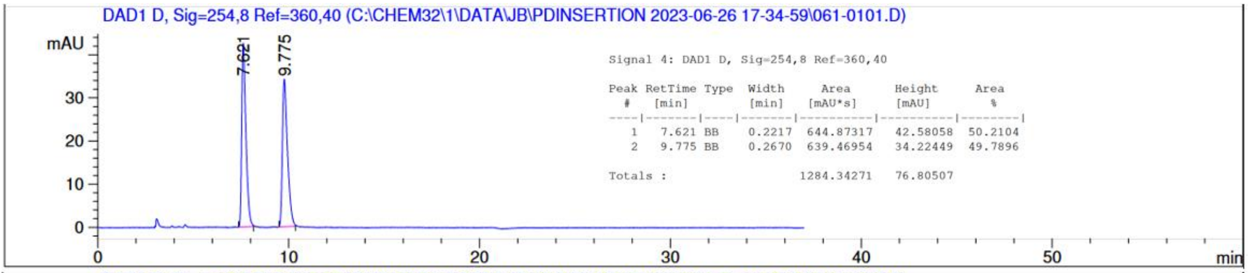

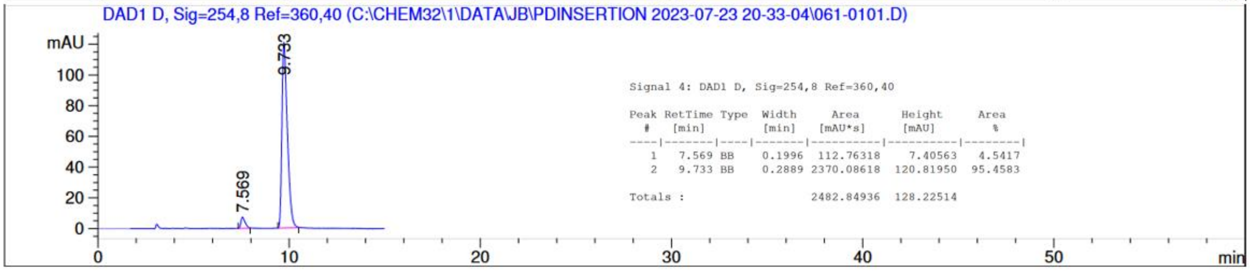

methyl (R)-4-(2-(hydroxymethyl)-4,4-dimethyl-5-phenyl-3,4-dihydro-2H-pyrrol-2-yl)benzoate (3k)

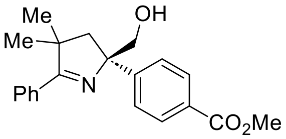

Synthesized according to **GP4** on a 0.2 mmol scale. Purified by silica gel column chromatography (0% → 40% EtOAc/Pentane) to give **3k** as a white solid (57 mg, 84% yield, 74.5:25.5 er).

^{1}H NMR (500 MHz, Methanol-d$_4$): δ 8.03 – 7.97 (m, 2H), 7.74 – 7.69 (m, 2H), 7.60 – 7.54 (m, 2H), 7.50 – 7.42 (m, 3H), 3.90 (s, 3H), 3.79 (ABq, *J* = 11.2 Hz, 2H), 2.41 (ABq, *J* = 13.0 Hz, 2H), 1.39 (s, 3H), 1.10 (s, 3H).

^{13}C NMR (101 MHz, CDCl$_3$): δ 181.8, 167.0, 151.4, 134.3, 130.0, 129.7, 128.6, 128.3, 128.3, 126.1, 78.7, 70.9, 52.1, 51.8, 49.7, 27.6, 27.6, 24.9.

IR (ATR, cm^{-1}): 3211 (br), 2953, 2866, 1717, 1608, 1570, 1435, 1275, 1181, 1110.

HRMS: (DART) m/z: $[M+H]^+$ Calculated for $C_{21}H_{24}NO_3$ 338.1751; Found 338.1755

MP: 84-87 °C

$[\alpha]^{D}_{20}$: (c = 0.31, CHCl$_3$) +61.52

HPLC: IA, 1 mL/min, 5% IPA/Hexane

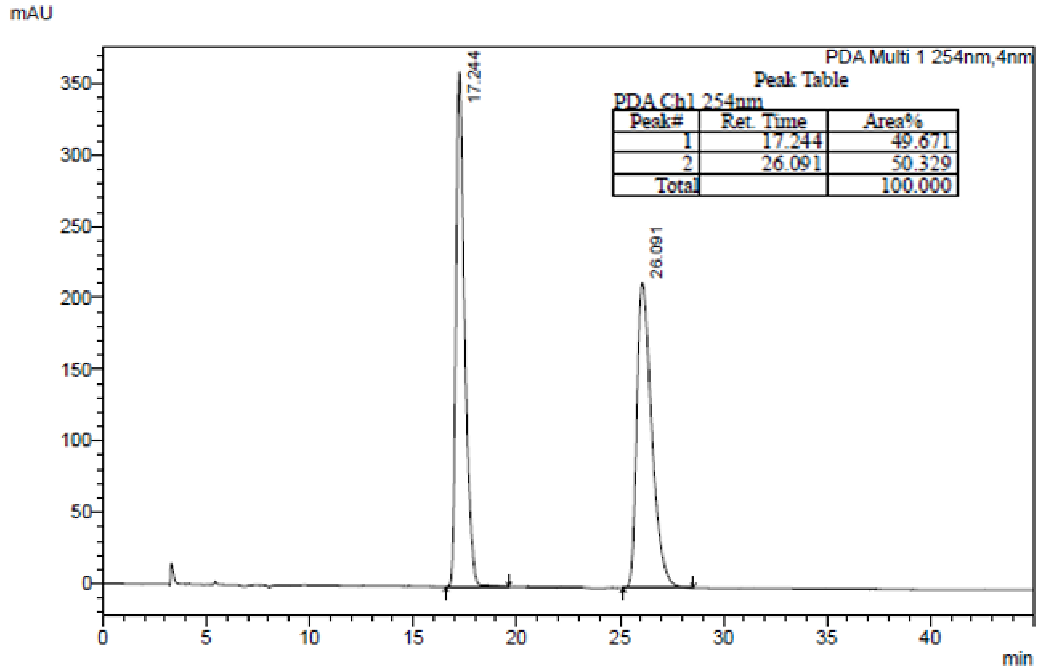
mAU
PDA Multi 1 254nm,4nm
Peak Table
PDA Ch1 254nm
Peak# | Ret. Time | Area%
1 | 17.244 | 49.671
2 | 26.091 | 50.329
Total | | 100.000
17.244
26.091
350
300
250
200
150
100
50
0
0
5
10
15
20
25
30
35
40
min

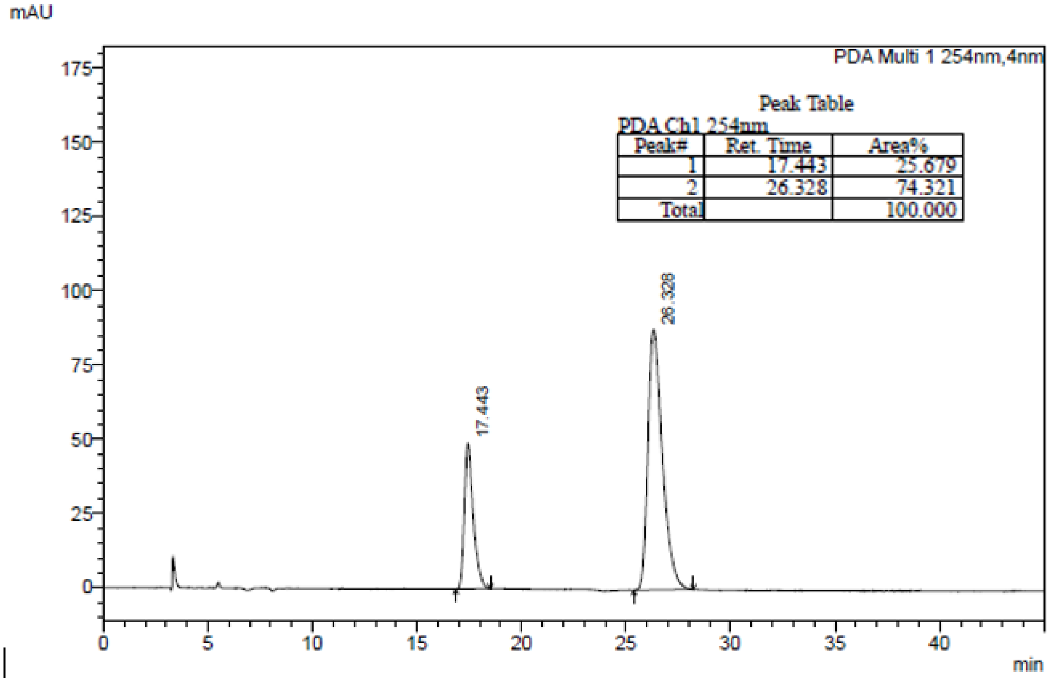
mAU
PDA Multi 1 254nm,4nm
Peak Table
PDA Ch1 254nm
Peak# | Ret. Time | Area%
1 | 17.443 | 25.679
2 | 26.328 | 74.321
Total | | 100.000
17.443
26.328
175
150
125
100
75
50
25
0
0
5
10
15
20
25
30
35
40
min

(R)-(4,4-dimethyl-5-phenyl-2-(p-tolyl)-3,4-dihydro-2H-pyrrol-2-yl)methanol (3l)

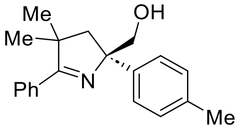

Synthesized according to **GP4** on a 0.2 mmol scale. Purified by silica gel column chromatography (0% → 40% EtOAc/Pentane) to give **3l** as a white solid (42.3 mg, 72% yield, 95:5 er).

^{1}H NMR (500 MHz, $CDCl_3$): δ 7.77 – 7.73 (m, 2H), 7.45 – 7.39 (m, 3H), 7.34 – 7.30 (m, 2H), 7.16 – 7.11 (m, 2H), 3.93 (d, J = 11.2 Hz, 1H), 3.62 (d, J = 11.3 Hz, 1H), 2.52 (s, 1H), 2.44 (d, J = 12.7 Hz, 1H), 2.33 (s, 3H), 2.20 (d, J = 12.7 Hz, 1H), 1.38 (s, 3H), 1.09 (s, 3H).
^{13}C NMR (126 MHz, $CDCl_3$): δ 181.2, 143.0, 136.2, 134.7, 129.7, 129.0, 128.2, 128.2, 125.9, 78.4, 71.1, 51.7, 49.5, 27.7 – 27.5 (m), 21.0.
IR (ATR, cm^{-1}): 3121 (br), 2960, 2929, 2865, 2843, 1738, 1598, 1569, 1510, 1445.
HRMS: (DART) m/z: $[M+H]^+$ Calculated for $C_{20}H_{24}NO$ 294.1852; Found 294.1855
MP: 73-75 °C
$[\alpha]_{20}^{D}$: (c = 0.34, CH_2Cl_2) +135.3
HPLC: IA, 1 mL/min, 5% IPA/Hexane

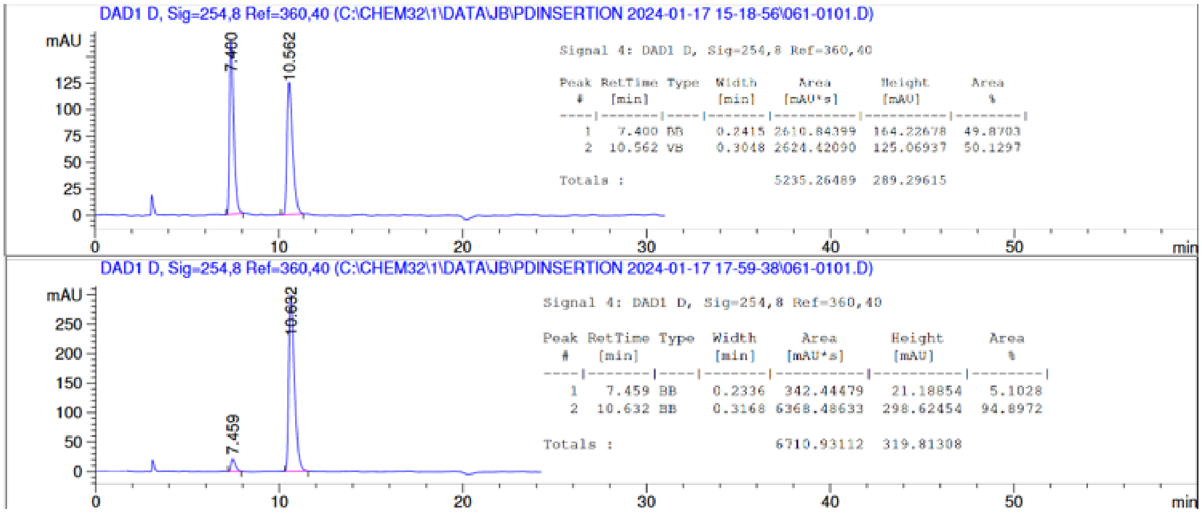

(R)-(2-([1,1'-biphenyl]-4-yl)-4,4-dimethyl-5-phenyl-3,4-dihydro-2H-pyrrol-2-yl)methanol (3m):

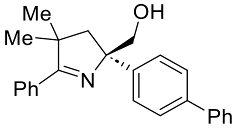

Synthesized according to **GP4** on a 0.2 mmol scale. Purified by silica gel column chromatography (0% → 40% EtOAc/Pentane) to give **3m** as a white solid (60.7 mg, 85% yield, 95:5 er).

^{1}H NMR (500 MHz, $CDCl_3$): δ 7.81 – 7.77 (m, 2H), 7.61 – 7.55 (m, 4H), 7.53 – 7.49 (m, 2H), 7.47 – 7.40 (m, 5H), 7.35 – 7.31 (m, 1H), 4.00 (d, J = 11.3 Hz, 1H), 3.68 (d, J = 11.3 Hz, 1H), 2.83 (s, 1H), 2.38 (ABq, J = 12.7 Hz, 2H), 1.40 (s, 3H), 1.13 (s, 3H).
^{13}C NMR (126 MHz, $CDCl_3$): δ 181.4, 145.1, 140.9, 139.5, 134.6, 129.8, 128.7, 128.3, 128.3, 127.1, 127.0, 127.0, 126.5, 78.5, 71.0, 51.8, 49.6, 27.7.
IR (ATR, cm^{-1}): 3198, 3057, 3027, 2960, 2929, 2865, 1739, 1600, 1571, 1485.
HRMS: (DART) m/z: $[M+H]^+$ Calculated for $C_{25}H_{26}NO$ 356.2009; Found 356.2006
MP: 56-58 °C
$[\alpha]_{20}^{D}$: (c = 0.265, CH_2Cl_2) +113.2

HPLC: IA, 1 mL/min, 5% IPA/Hexane

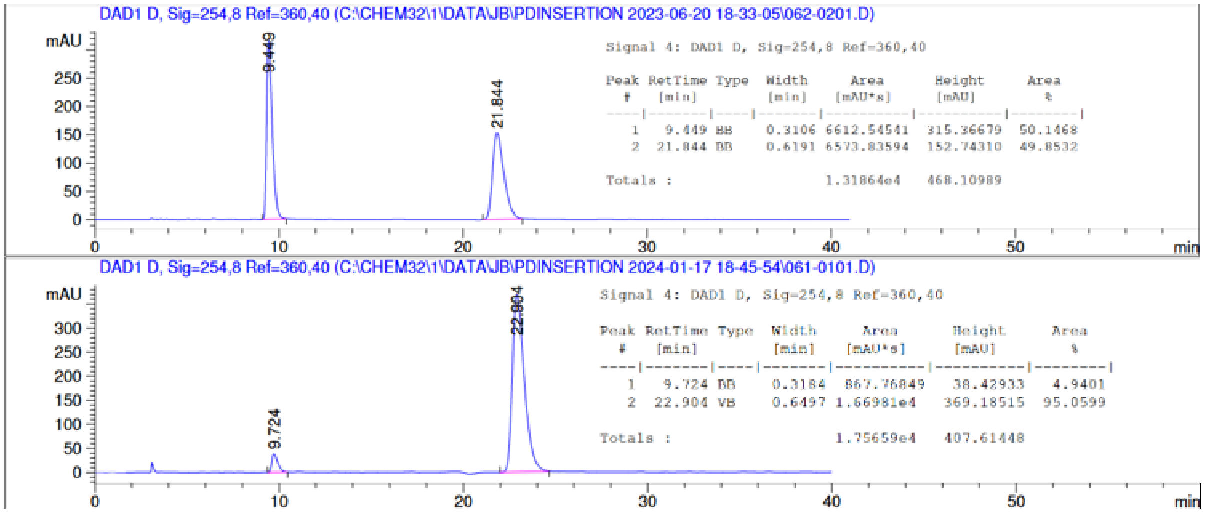

(R)-(2-(4-(tert-butyl)phenyl)-4,4-dimethyl-5-phenyl-3,4-dihydro-2H-pyrrol-2-yl)methanol (3n):

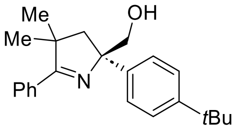

Synthesized according to **GP4** on a 0.2 mmol scale. Purified by silica gel column chromatography (0% → 40% EtOAc/Pentane) to give **3n** as a white solid (57.7 mg, 86% yield, 96.5:3.5 er).

^{1}H NMR (500 MHz, $CDCl_3$): δ 7.77 – 7.72 (m, 2H), 7.47 – 7.39 (m, 3H), 7.38 – 7.31 (m, 4H), 3.94 (d, J = 11.3 Hz, 1H), 3.62 (d, J = 11.3 Hz, 1H), 2.76 (s, 1H), 2.33 (ABq, J = 12.7 Hz, 2H), 1.38 (s, 3H), 1.31 (s, 9H), 1.09 (s, 3H).
^{13}C NMR (126 MHz, $CDCl_3$): δ 181.1, 149.3, 142.8, 134.7, 129.6, 128.3, 128.2, 125.6, 125.1, 78.5, 71.0, 51.7, 49.4, 34.4, 31.4, 27.7, 27.6.
IR (ATR, cm^{-1}): 3208 (br), 2960, 2902, 2866, 1610, 1573, 1509, 1444, 1393, 1362.
HRMS: (DART) m/z: $[M+H]^+$ Calculated for $C_{23}H_{30}NO$ 336.2322; Found 336.2315
MP: 47-50 °C
$[\alpha]^{D}_{20}$: (c = 0.26, CH_2Cl_2) +115.4
HPLC: IA, 1 mL/min, 5% IPA/Hexane

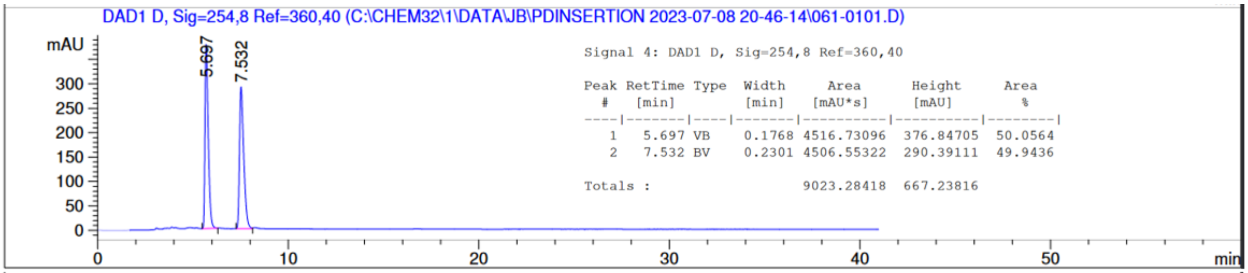

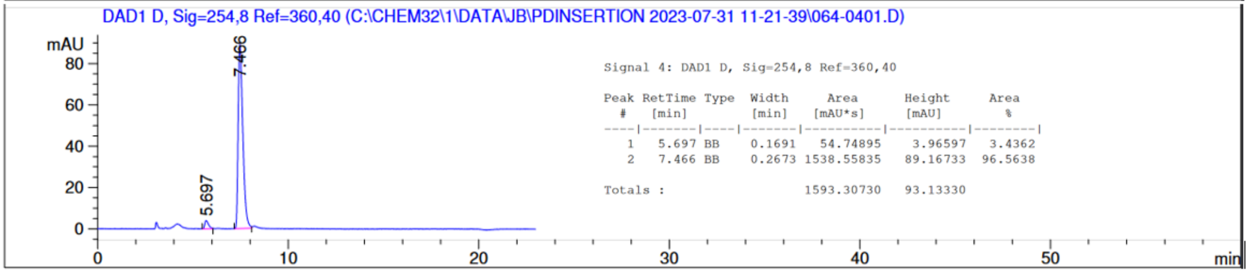

(R)-(2-(3,5-di-tert-butylphenyl)-4,4-dimethyl-5-phenyl-3,4-dihydro-2H-pyrrol-2-yl)methanol (3o):

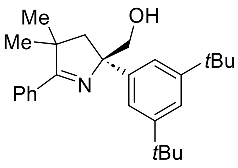

Synthesized according to **GP4** on a 0.2 mmol scale. Purified by silica gel column chromatography (0% → 40% EtOAc/Pentane) to give **3o** as a white solid (62.3 mg, 80% yield, 95.5:4.5 er).

^{1}H NMR (500 MHz, $CDCl_3$): δ 7.72 – 7.67 (m, 2H), 7.46 – 7.39 (m, 3H), 7.29 (s, 3H), 3.97 (dd, J = 11.5, 3.9 Hz, 1H), 3.65 (dd, J = 11.3, 7.9 Hz, 1H), 2.86 (s, 1H), 2.36 (ABq, J = 12.7 Hz, 2H), 1.38 (s, 3H), 1.33 (s, 18H), 1.05 (s, 3H).
^{13}C NMR (126 MHz, $CDCl_3$): δ 181.3, 150.4, 145.2, 134.9, 129.5, 128.3, 128.2, 120.4, 120.2, 79.3, 70.9, 51.8, 49.5, 35.0, 31.6, 27.8, 27.5.
IR (ATR, cm^{-1}): 3149 (br), 2962, 2931, 2861, 1739, 1594, 1571, 1448, 1361, 1308.
HRMS: (DART) m/z: $[M+H]^+$ Calculated for $C_{27}H_{38}NO$ 392.2950; Found 392.1951
MP: 150-153 °C
$[\alpha]^D_{20}$: (c = 0.405, CH_2Cl_2) +103.7
HPLC: IA, 1 mL/min, 5% IPA/Hexane

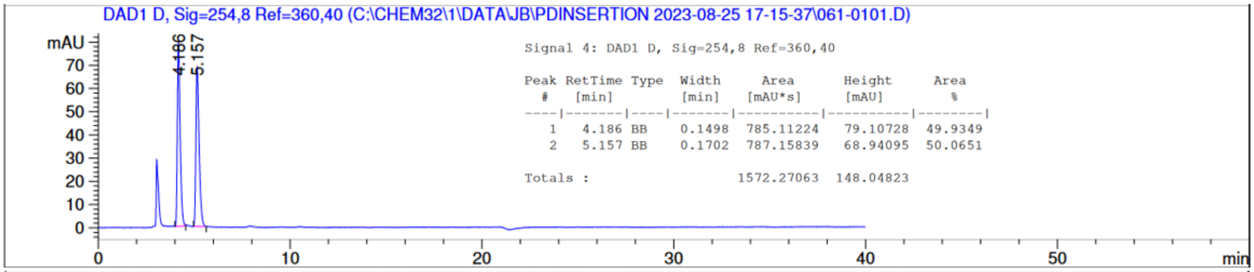

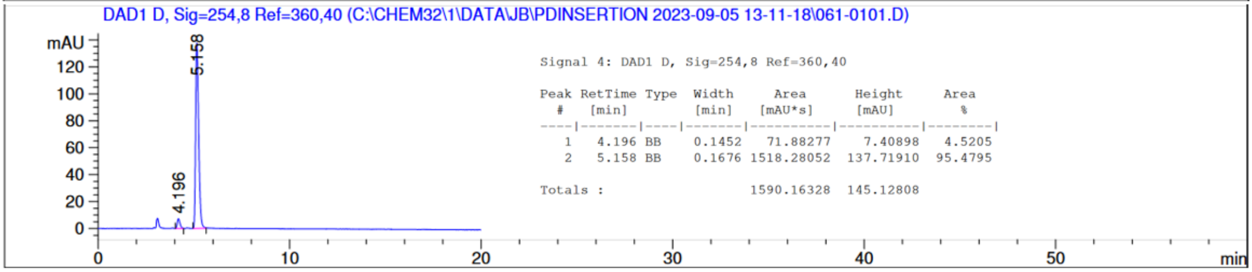

(R)-(2-(3,5-dimethoxyphenyl)-4,4-dimethyl-5-phenyl-3,4-dihydro-2H-pyrrol-2-yl)methanol (3p):

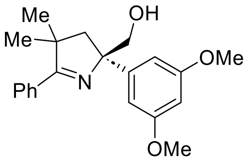

Synthesized according to **GP4** on a 0.2 mmol scale. Purified by silica gel column chromatography (0% → 40% EtOAc/Pentane) to give **3p** as a white solid (51.6 mg, 76% yield, 98:2 er).

^{1}H NMR (500 MHz, $CDCl_3$): δ 7.73 – 7.69 (m, 2H), 7.46 – 7.37 (m, 3H), 6.61 (d, *J* = 2.3 Hz, 2H), 6.34 (t, *J* = 2.3 Hz, 1H), 3.94 (d, *J* = 11.3 Hz, 1H), 3.79 (s, 6H), 3.60 (d, *J* = 11.3 Hz, 1H), 3.02 (s, 1H), 2.31 (ABq, *J* = 12.7 Hz, 2H), 1.35 (s, 3H), 1.09 (s, 3H).
^{13}C NMR (126 MHz, $CDCl_3$): δ 181.5, 160.6, 148.7, 134.5, 129.7, 128.3, 128.2, 104.4, 98.3, 78.8, 70.9, 55.3, 51.6, 49.4, 27.6, 27.5.
IR (ATR, cm^{-1}): 3176 (br), 2957, 2932, 2846, 1738, 1606, 1586, 1458, 1427, 1363.
HRMS: (DART) m/z: $[M+H]^+$ Calculated for $C_{21}H_{26}NO_3$ 340.1907; Found 340.1902
MP: 145-146 °C
$[\alpha]^D_{20}$: (c = 0.25, CH_2Cl_2) +128.0
HPLC: IA, 1 mL/min, 5% IPA/Hexane

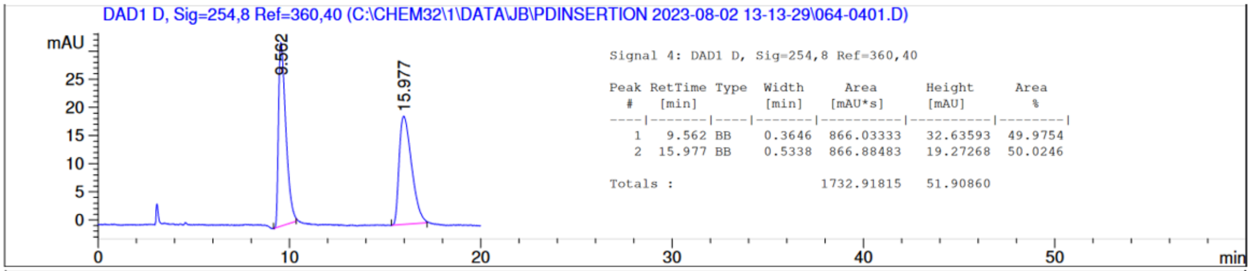

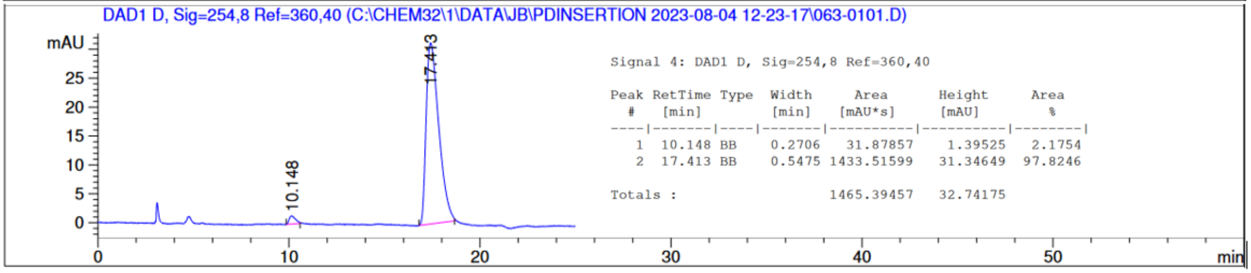

(R)-(2-(benzo[d][1,3]dioxol-5-yl)-4,4-dimethyl-5-phenyl-3,4-dihydro-2H-pyrrol-2-yl)methanol (3q)

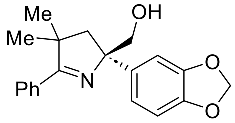

Synthesized according to **GP4** on a 0.2 mmol scale. Purified by silica gel column chromatography (0% → 40% EtOAc/Pentane) to give **3q** as a white solid (50.5 mg, 78% yield, 96:4 er).

^{1}H NMR (500 MHz, CDCl$_3$): δ 7.79 – 7.74 (m, 2H), 7.46 – 7.38 (m, 3H), 6.95 (d, J = 1.8 Hz, 1H), 6.88 (dd, J = 8.1, 1.8 Hz, 1H), 6.77 (d, J = 8.1 Hz, 1H), 5.95 – 5.92 (m, 2H), 3.89 (d, J = 11.1 Hz, 1H), 3.59 (d, J = 11.2 Hz, 1H), 2.47 (s, 1H), 2.28 (ABq, J = 12.7 Hz, 2H), 1.38 (s, 3H), 1.12 (s, 3H).
^{13}C NMR (126 MHz, CDCl$_3$): δ 181.2, 147.6, 146.1, 140.1, 134.5, 129.8, 128.2, 128.2, 119.1, 108.0, 106.9, 100.9, 78.3, 71.2, 51.7, 49.7, 27.6.
IR (ATR, cm^{-1}): 3428, 3171, 2964, 2927, 1738, 1616, 1599, 1570, 1484, 1431.
HRMS: (DART) m/z: [M+H]$^+$ Calculated for $C_{20}H_{22}NO_3$ 324.1594; Found 324.1602
MP: 75-77 °C
$[\alpha]_{20}^{D}$: (c = 0.44, CH_2Cl_2) +122.7
HPLC: IA, 1 mL/min, 5% IPA/Hexane

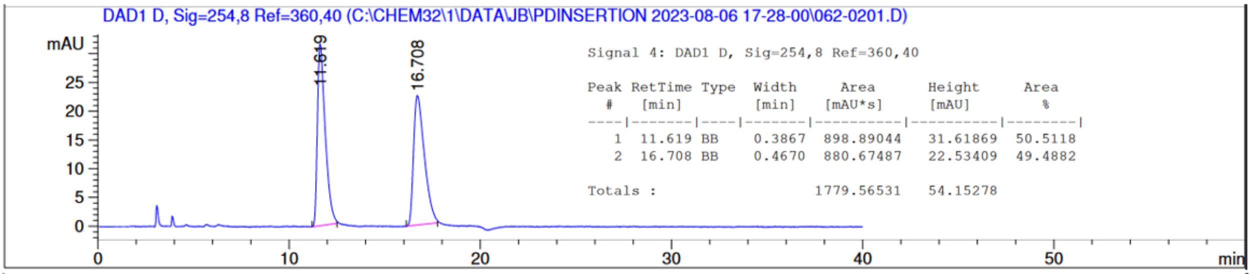

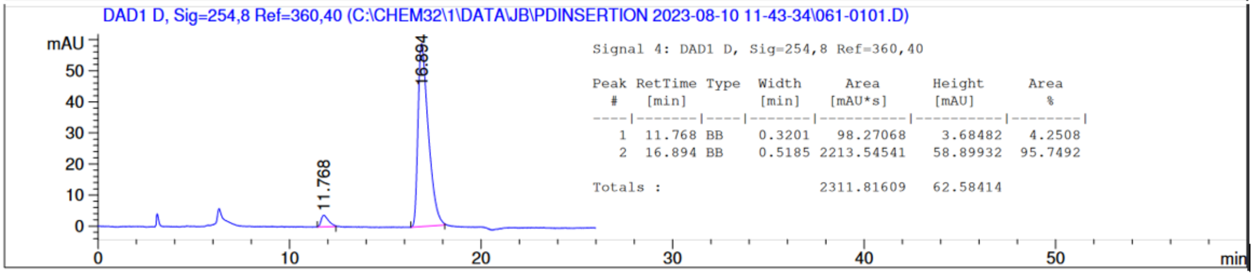

(R)-(4,4-dimethyl-5-phenyl-2-(phenyl-d5)-3,4-dihydro-2H-pyrrol-2-yl)methanol (3r):

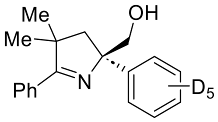

Synthesized according to **GP4** on a 0.2 mmol scale. Purified by silica gel column chromatography (0% → 40% EtOAc/Pentane) to give **3r** as a white solid (48.6 mg, 85% yield, 96.5:3.5 er).

^{1}H NMR (500 MHz, $CDCl_3$): δ 7.78 – 7.74 (m, 2H), 7.45 – 7.40 (m, 3H), 3.97 – 3.92 (m, 1H), 3.66 – 3.61 (m, 1H), 2.76 (s, 1H), 2.34 (ABq, J = 12.7 Hz, 2H), 1.38 (s, 3H), 1.08 (s, 3H).
^{13}C NMR (126 MHz, $CDCl_3$): δ 181.3, 145.9, 134.6, 129.7, 128.3 (d, J = 6.6 Hz), 78.6, 71.0, 51.7, 49.5, 27.6, 27.6.
IR (ATR, cm^{-1}): 3144 (br), 2958, 2934, 2866, 2842, 2705, 2272, 1599, 1570, 1445.
HRMS: (DART) m/z: $[M+H]^+$ Calculated for $C_{19}H_{17}NOD_5$ 285.2010; Found 285.2000
MP: 58-60 °C
$[\alpha]_{20}^{D}$: (c = 0.475, CH_2Cl_2) +122.1
HPLC: IA, 1 mL/min, 5% IPA/Hexane

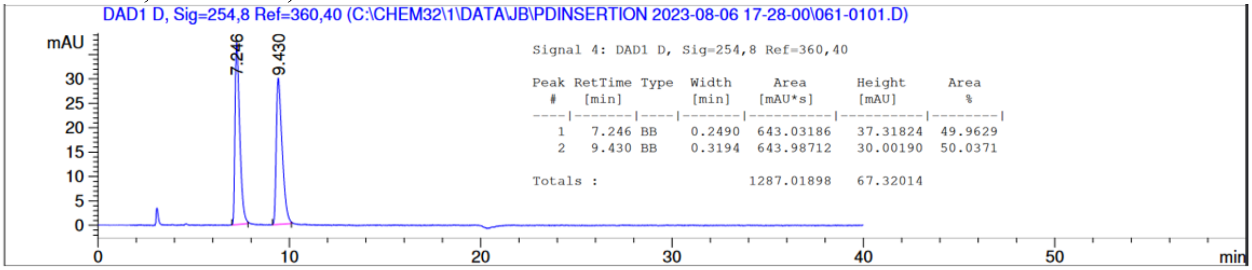

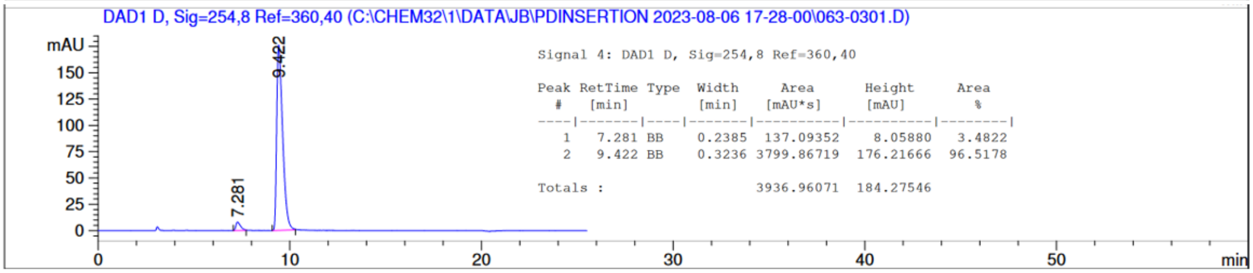

(R)-(4,4-dimethyl-2-phenyl-5-(pyridin-2-yl)-3,4-dihydro-2H-pyrrol-2-yl)methanol (3s):

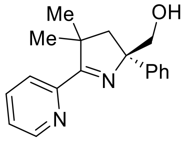

Synthesized according to **GP4** on a 0.2 mmol scale. Purified by silica gel column chromatography (0% → 40% EtOAc/Pentane) to give **3s** as a yellow oil (30.7 mg, 55% yield, 99:1 er).

^{1}H NMR (500 MHz, CDCl$_3$): δ 8.64 – 8.58 (m, 1H), 8.21 (dt, *J* = 7.9, 1.1 Hz, 1H), 7.74 (td, *J* = 7.7, 1.8 Hz, 1H), 7.44 – 7.40 (m, 2H), 7.35 – 7.29 (m, 3H), 7.25 – 7.21 (m, 1H), 4.01 – 3.93 (m, 1H), 3.73 – 3.66 (m, 1H), 2.43 (d, *J* = 12.8 Hz, 1H), 2.28 – 2.19 (m, 2H), 1.58 (s, 3H), 1.29 (s, 3H).
^{13}C NMR (126 MHz, CDCl$_3$): δ 180.0, 153.5, 148.5, 146.0, 136.1, 128.3, 126.6, 126.0, 124.3, 123.7, 79.2, 71.3, 51.8, 49.9, 27.6, 27.6 – 27.5 (m).
IR (ATR, cm^{-1}): 3337 (br), 3057, 2957, 2929, 2865, 1739, 1611, 1585, 1566, 1446.
HRMS: (DART) m/z: $[M+H]^+$ Calculated for $C_{18}H_{21}N_2O$ 281.1648; Found 281.1658
$[\alpha]^{D}_{20}$: (*c* = 0.63, CH_2Cl_2) +139.7
HPLC: IC, 0.5 mL/min, 10% IPA/Hexane

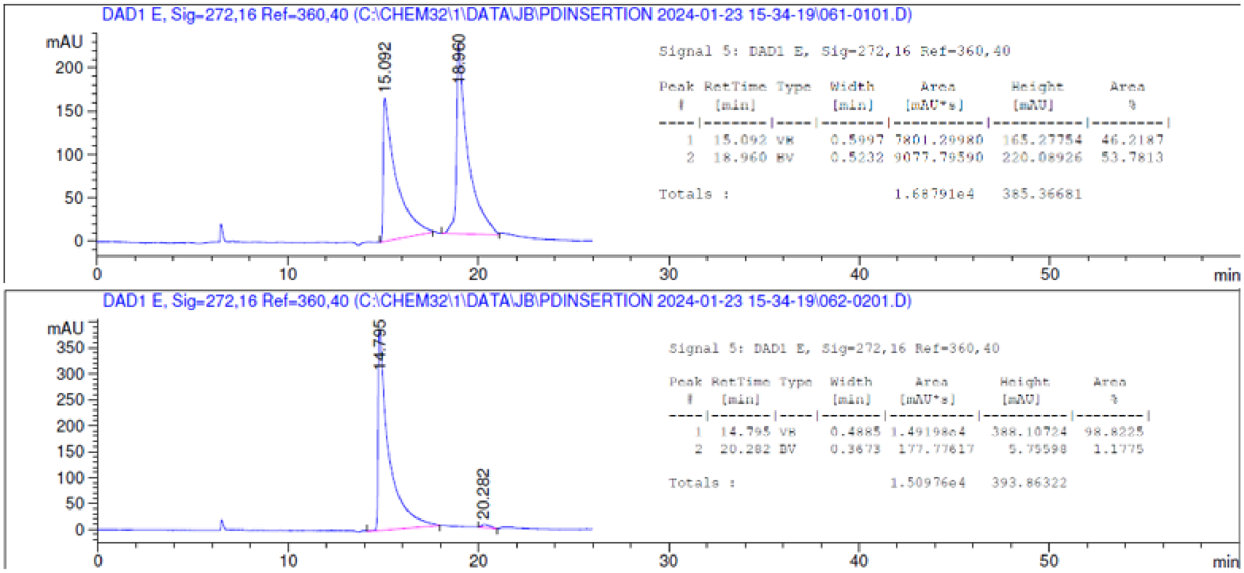

(R)-(2-(6-chloropyridin-3-yl)-4,4-dimethyl-5-phenyl-3,4-dihydro-2H-pyrrol-2-yl)methanol (3t)

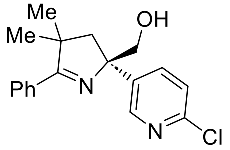

Synthesized according to **GP4** on a 0.2 mmol scale. Purified by silica gel column chromatography (0% → 40% EtOAc/Pentane) to give **3t** as a white solid (56 mg, 89% yield, 78:22 er).

^{1}H NMR (500 MHz, CDCl$_3$): δ 8.47 (m, 1H), 7.81 – 7.72 (m, 3H), 7.50 – 7.40 (m, 3H), 7.29 (m, 1H), 3.92 – 3.86 (m, 1H), 3.69 – 3.63 (m, 1H), 2.47 (m, 1H), 2.18 (m, 1H), 2.14 (t, *J* = 6.9 Hz, 1H), 1.46 (m, 3H), 1.15 (m, 3H).
^{13}C NMR (126 MHz, CDCl$_3$): δ 182.0, 149.9, 147.8, 140.5, 136.9 (d, *J* = 0.9 Hz), 134.0, 130.1, 128.4, 128.2, 123.8, 71.1, 52.1, 49.4, 27.8, 27.6.

IR (ATR, cm^{-1}): 3315 (br) 2958, 2926, 2867, 1610, 1560, 1453, 1365, 1103, 632
HRMS: (DART) m/z: $[M+H]^+$ Calculated for $C_{18}H_{20}N_2OCl$ 315.1259; Found 315.1252
MP: 118-120 °C
$[\boldsymbol{\alpha}]_{\mathbf{20}}^{\boldsymbol{D}}$**:** (*c* = 0.255, $CHCl_3$) +71.35
HPLC: IC, 1 mL/min, 5% IPA/Hexane

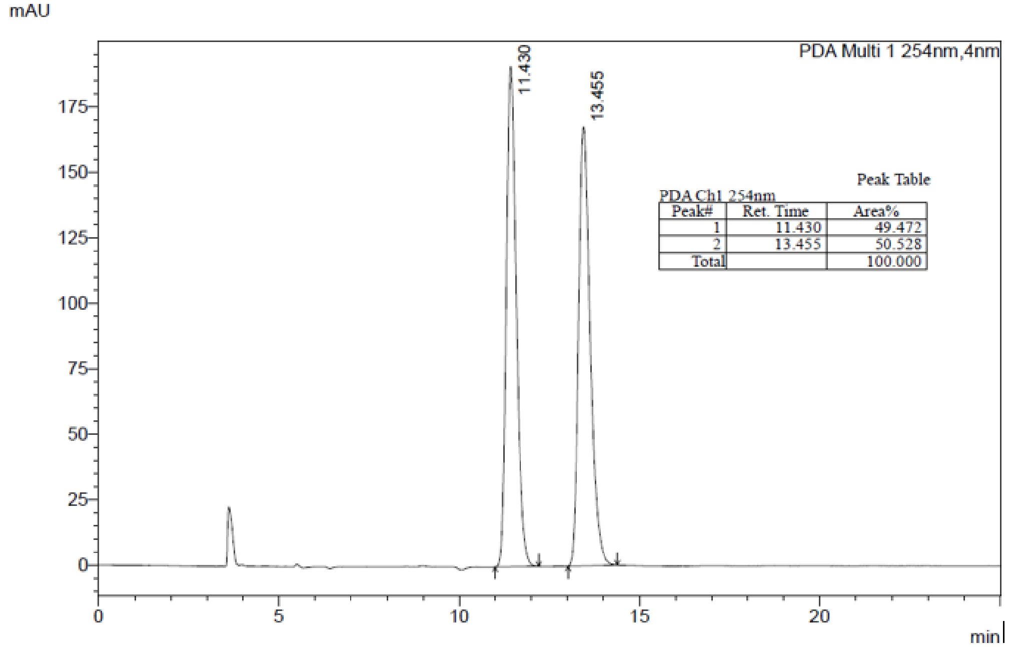

Peak Table
PDA Ch1 254nm

Peak#	Ret. Time	Area%
1	11.430	49.472
2	13.455	50.528
Total		100.000

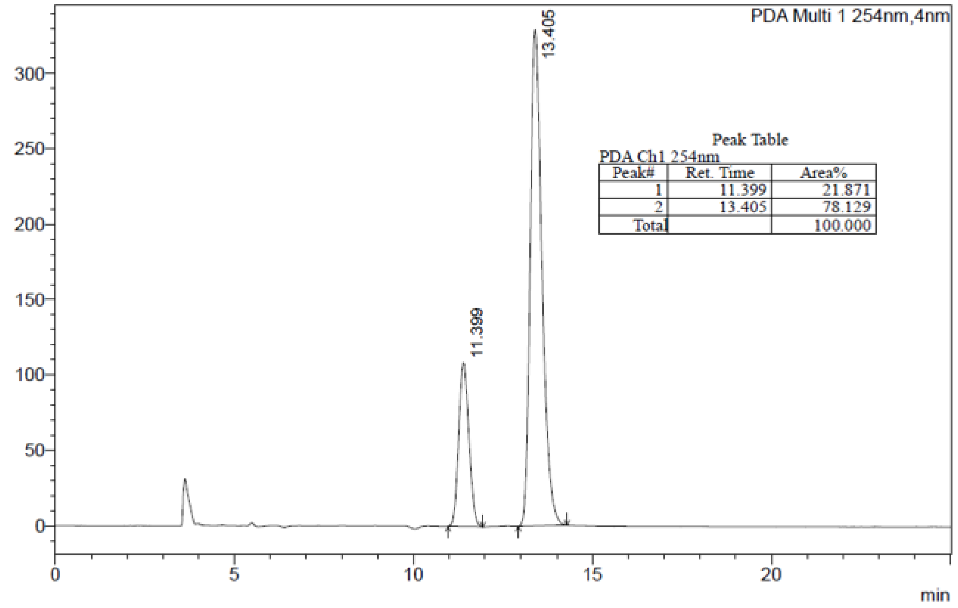

Peak Table
PDA Ch1 254nm

Peak#	Ret. Time	Area%
1	11.399	21.871
2	13.405	78.129
Total		100.000

(R)-(1,3-diphenyl-2-azaspiro[4.5]dec-1-en-3-yl)methanol (3u):

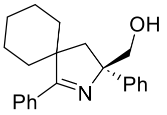

Synthesized according to **GP4** on a 0.2 mmol scale. Purified by silica gel column chromatography (0% → 40% EtOAc/Pentane) to give **3u** as a clear oil (55.0 mg, 86% yield, 95.5:4.5 er).

^{1}H NMR (500 MHz, $CDCl_3$): δ 7.63 – 7.56 (m, 2H), 7.47 – 7.40 (m, 5H), 7.36 – 7.31 (m, 2H), 7.23 (ddt, J = 7.9, 6.7, 1.3 Hz, 1H), 3.94 (d, J = 11.3 Hz, 1H), 3.62 (d, J = 11.3 Hz, 1H), 2.68 (s, 1H), 2.39 – 2.32 (m, 2H), 1.76 – 1.59 (m, 4H), 1.54 – 1.44 (m, 2H), 1.40 – 1.21 (m, 2H), 1.16 – 1.03 (m, 2H).
^{13}C NMR (126 MHz, $CDCl_3$): δ 182.4, 146.4, 135.6, 129.2, 128.3, 128.3, 128.1, 126.6, 125.9, 79.5, 71.1, 57.6, 43.2, 35.0, 33.5, 25.4, 23.2, 22.9.
IR (ATR, cm^{-1}): 3236 (br), 3056, 3023, 2925, 2853, 1738, 1626, 1600, 1572, 1492.
HRMS: (DART) m/z: $[M+H]^+$ Calculated for $C_{22}H_{26}NO$ 320.2009; Found 320.2011
$[\alpha]^{D}_{20}$**:** (c = 0.195, CH_2Cl_2) +82.1
HPLC: IA, 1 mL/min, 5% IPA/Hexane

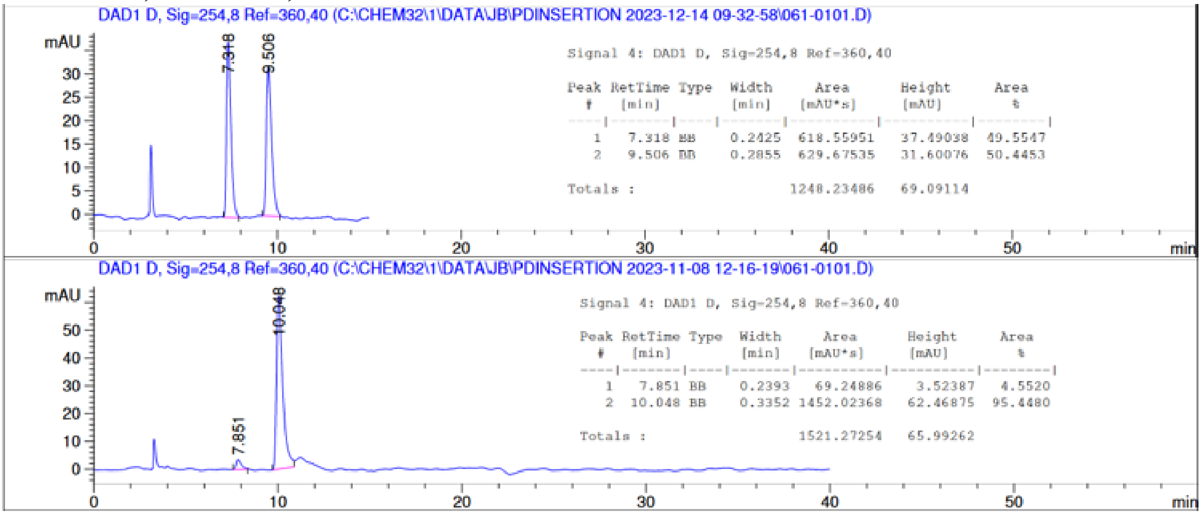

(R)-(5-phenyl-2-(p-tolyl)-3,4-dihydro-2H-pyrrol-2-yl)methanol (3v)

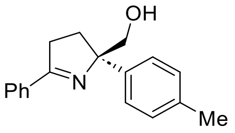

Synthesized according to **GP4** on a 0.2 mmol scale. Purified by silica gel column chromatography (0% → 40% EtOAc/Pentane) to give **3v** as a yellow solid (20.0 mg, 38% yield, 84:16 er).

^{1}H NMR (500 MHz, $CDCl_3$): δ 7.88 – 7.83 (m, 2H), 7.47 – 7.43 (m, 1H), 7.43 – 7.38 (m, 2H), 7.37 – 7.33 (m, 2H), 7.16 – 7.11 (m, 2H), 3.87 (ABq, J = 11.4 Hz, 2H), 3.12 – 3.03 (m, 1H), 2.94 – 2.85 (m, 1H), 2.61 – 2.53 (m, 1H), 2.34 – 2.32 (s, 3H), 2.24 – 2.15 (m, 1H).
^{13}C NMR (126 MHz, $CDCl_3$): δ 174.2, 142.3, 136.4, 134.1, 130.7, 129.0, 128.4, 128.0, 126.0, 83.1, 70.8, 35.9, 32.0, 21.0.
IR (ATR, cm^{-1}): 3235 (br), 3057, 3024, 2969, 2935, 2919, 2862, 1739, 1683, 1620.
HRMS: (DART) m/z: $[M+H]^+$ Calculated for $C_{18}H_{20}NO$ 266.1539; Found 266.1535
MP: 50-55 °C
$[\alpha]^{D}_{20}$**:** (c = 0.1, CH_2Cl_2) +100.0

HPLC: IA, 1 mL/min, 3% IPA/Hexane

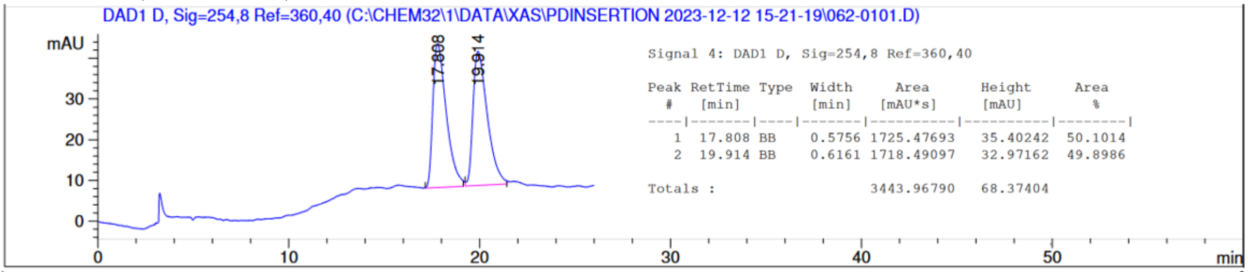

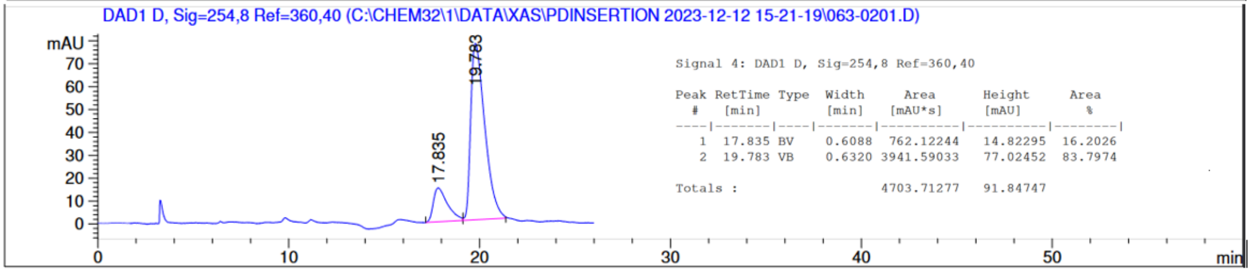

(R)-(2-(4-fluorophenyl)-5-phenyl-3,4-dihydro-2H-pyrrol-2-yl)methanol (3w)

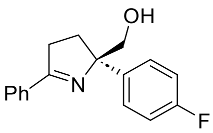

Synthesized according to **GP4** on a 0.2 mmol scale. Purified by silica gel column chromatography (0% → 40% EtOAc/Pentane) to give **3w** as an off-white solid (21.2 mg, 39% yield, 82.5:17.5 er).

^{1}H NMR (500 MHz, $CDCl_3$): δ 7.88 – 7.83 (m, 2H), 7.47 – 7.40 (m, 5H), 7.05 – 6.99 (m, 2H), 3.84 (ABq, J = 11.4 Hz, 2H), 3.13 – 3.05 (m, 1H), 2.96 – 2.87 (m, 1H), 2.62 – 2.54 (m, 1H), 2.20 – 2.12 (m, 1H).
^{13}C NMR (126 MHz, $CDCl_3$): δ 174.4, 161.7 (d, J = 245.0 Hz), 141.1 (d, J = 3.1 Hz), 133.9, 130.9, 128.4, 128.0, 127.7 (d, J = 7.8 Hz), 115.0 (d, J = 21.2 Hz), 82.9, 70.9, 36.0, 32.1.
^{19}F NMR (376 MHz, $CDCl_3$): δ -116.4.
IR (ATR, cm^{-1}): 3221 (br), 3065, 2970, 2922, 2862, 1739, 1687, 1622, 1599, 1577.
HRMS: (DART) m/z: $[M+H]^+$ Calculated for $C_{17}H_{17}NOF$ 270.1289; Found 270.1287
MP: 59-62 °C
$[\alpha]_{20}^{D}$: (c = 0.515, CH_2Cl_2) +97.1
HPLC: IA, 1 mL/min, 3% IPA/Hexane

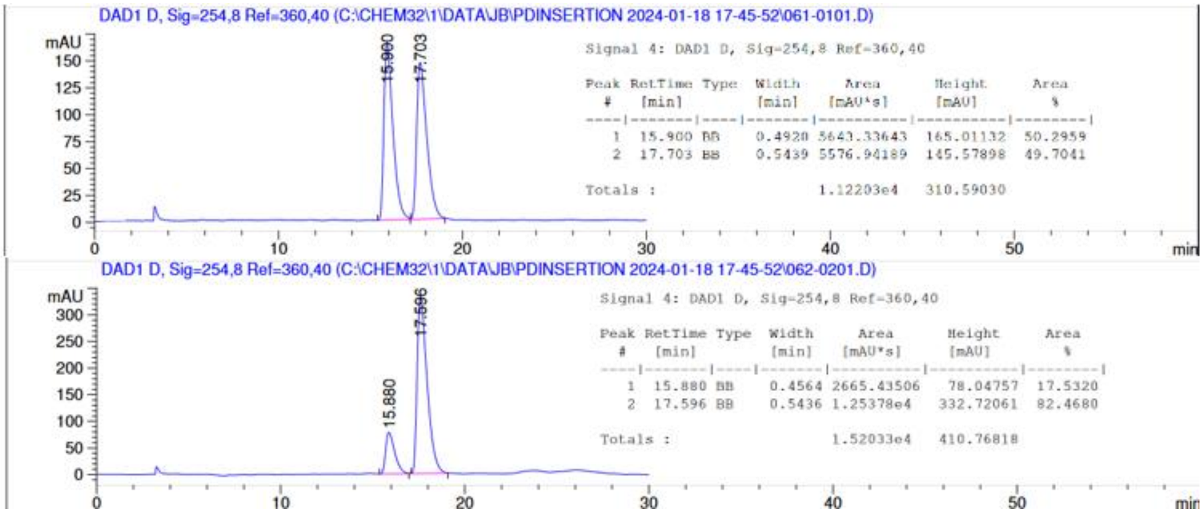

(R)-(2,5-diphenyl-3,4-dihydro-2H-pyrrol-2-yl)methanol (3x):

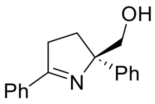

Synthesized according to **GP4** on a 0.2 mmol scale. Purified by silica gel column chromatography (0% → 40% EtOAc/Pentane) to give **3x** as a white solid (17.9 mg, 26% yield, 91:9 er).

^{1}H NMR (500 MHz, CDCl$_3$): δ 7.85 – 7.80 (m, 2H), 7.49 – 7.43 (m, 3H), 7.40 (ddt, *J* = 8.3, 6.7, 1.3 Hz, 2H), 7.35 – 7.31 (m, 2H), 7.26 – 7.21 (m, 1H), 4.04 (d, *J* = 11.5 Hz, 1H), 3.76 (d, *J* = 11.5 Hz, 1H), 3.10 – 3.01 (m, 1H), 2.91 – 2.82 (m, 1H), 2.66 – 2.59 (m, 1H), 2.25 – 2.17 (m, 1H).
^{13}C NMR (126 MHz, CDCl$_3$): δ 174.6, 145.3, 133.9, 130.8, 128.4, 128.3, 128.1, 126.8, 126.0, 83.4, 70.7, 35.9, 31.8.
IR (ATR, cm^{-1}): 3236 (br), 3058, 3025, 2970, 2945, 2921, 2861, 1738, 1621, 1575.
HRMS: (DART) m/z: [M+H]$^+$ Calculated for $C_{17}H_{18}NO$ 252.1383; Found 252.1375
MP: 94-95 °C
$[\alpha]_{20}^{D}$: (*c* = 0.455, CH_2Cl_2) +140.7
HPLC: IA, 1 mL/min, 3% IPA/Hexane

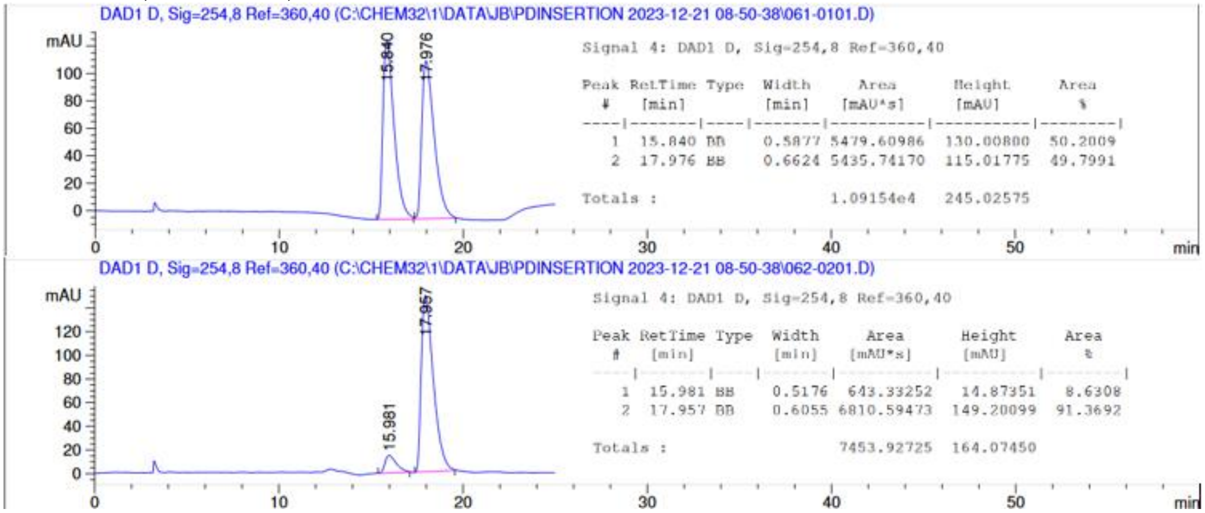

(R)-2-(3,5-dimethoxyphenyl)-4,4-dimethyl-5-phenyl-2-((4,4,5,5-tetramethyl-1,3,2-dioxaborolan-2-yl)methyl)-3,4-dihydro-2H-pyrrole (2p):

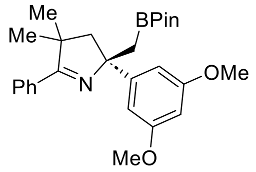

Synthesized according to **GP5** on a 1 mmol scale. Purified by trituration with pentanes to give **2p** as a tan solid (396 mg, 88% yield, >99:1 er).

^{1}H NMR (500 MHz, CDCl$_3$): δ 7.77 – 7.73 (m, 2H), 7.41 – 7.35 (m, 3H), 6.68 (d, J = 2.3 Hz, 2H), 6.28 (t, J = 2.3 Hz, 1H), 3.77 (s, 6H), 2.43 (ABq, J = 12.8 Hz, 2H), 1.66 (ABq, J = 15.3 Hz, 2H), 1.37 (s, 3H), 1.21 (s, 6H), 1.19 (s, 6H), 1.09 (s, 3H).
^{13}C NMR (126 MHz, CDCl$_3$): δ 177.6, 160.3, 153.8, 135.4, 129.1, 128.2, 128.0, 104.0, 97.8, 82.9, 75.1, 55.3, 54.2, 51.3, 27.2, 27.2, 25.0, 24.9.
IR (ATR, cm^{-1}): 2971, 1739, 1607, 1583, 1455, 1360, 1315, 1256, 1198, 1156
HRMS: (DART) m/z: [M+H]$^+$ Calculated for $C_{27}H_{37}BNO_4$ 450.2810; Found 450.2819
MP: 131-135 °C
$[\alpha]^{D}_{20}$: (c = 0.255, CH_2Cl_2) +47.1
HPLC: IB, 0.5 mL/min, 1% IPA/Hexane

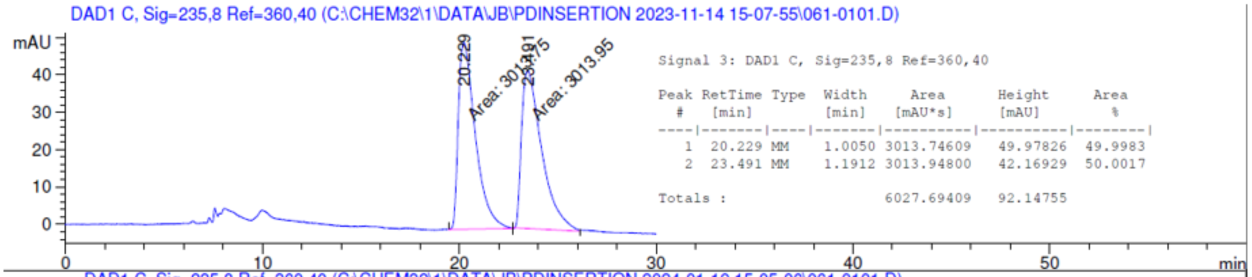

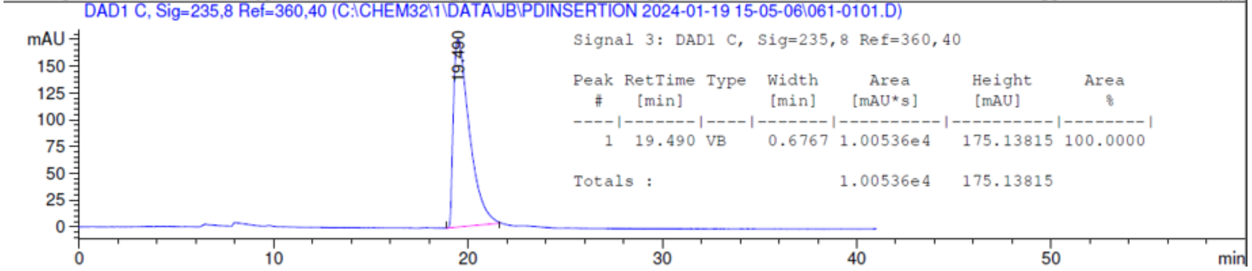

((2R,5S)-2-(3,5-dimethoxyphenyl)-4,4-dimethyl-5-phenylpyrrolidin-2-yl)methanol (4):

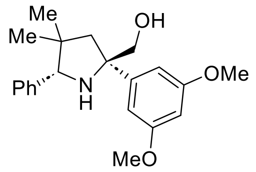

Synthesized according to **GP6** on a 0.059 mmol scale. Purified by silica gel column chromatography (0% → 35% EtOAc/Pentane) to give **4** as a clear oil (15 mg, 74% yield, >20:1 dr, >99:1er). Stereochemistry determined by 2D NMR experiments (see p. SXX)

^{1}H NMR (500 MHz, CDCl$_3$): δ 7.53 – 7.48 (m, 2H), 7.36 – 7.31 (m, 2H), 7.30 – 7.24 (m, 1H), 6.87 (d, J = 2.2 Hz, 2H), 6.37 (t, J = 2.3 Hz, 1H), 4.14 (s, 1H), 3.85 (s, 6H), 3.69 (ABq, J = 10.9 Hz, 2H), 2.30 – 2.09 (s (br), 1H), 1.99 (ABq, J = 12.7 Hz, 2H), 1.32 – 1.23 (m, 1H), 1.03 (s, 3H), 0.53 (s, 3H).
^{13}C NMR (101 MHz, CDCl$_3$): δ 160.8, 149.9, 140.6, 127.8, 127.7, 127.0, 105.1, 97.8, 70.6, 70.0, 64.7, 55.3, 51.6, 41.8, 26.6, 23.5
IR (ATR, cm^{-1}): 3357 (br), 3055, 2959, 2867, 1594, 1455, 1423, 1265, 1204, 1152
HRMS: (DART) m/z: [M+H]$^+$ Calculated for $C_{21}H_{28}NO_3$ 342.2064; Found 342.2061

$[\alpha]_{20}^{D}$**:** (c = 0.750, CH_2Cl_2) -13.9
HPLC: IA, 1 mL/min, 5% IPA/Hexane

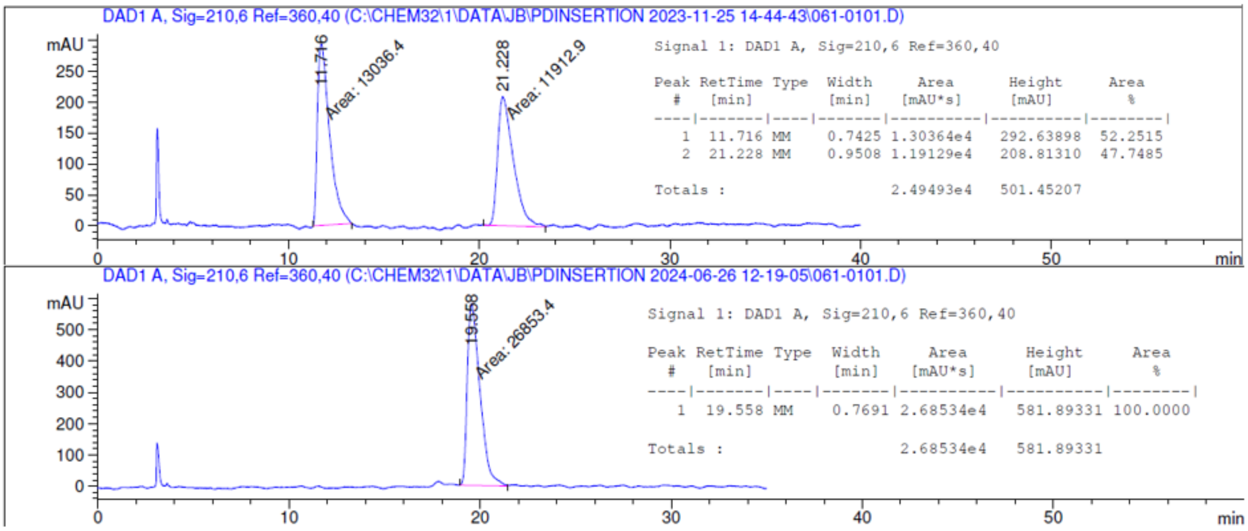

(R)-(4,4-dimethyl-2,5-diphenyl-3,4-dihydro-2H-pyrrol-2-yl)methan-d-ol (3a-D)

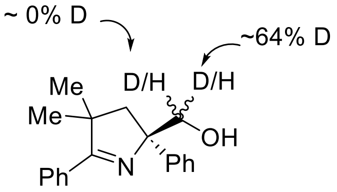

Synthesized according to **GP4** on a 1.0 mmol scale. Purified by silica gel column chromatography (0% → 40% EtOAc/Pentane) to give **3a-D** as a white solid (191 mg, 68% yield, 96:4 er).

^{1}H NMR (500 MHz, $CDCl_3$): δ 7.77 – 7.74 (m, 2H), 7.46 – 7.40 (m, 5H), 7.35 – 7.30 (m, 2H), 7.25 – 7.20 (m, 1H), 3.97 – 3.91 (m, 1H), 3.65 – 3.60 (m, 1H, ~ 64% D)), 2.91 (s (b), 1H), 2.33 (ABq, J = 12.7 Hz, 2H), 1.37 (s, 3H), 1.07 (s, 3H).
^{13}C NMR (101 MHz, $CDCl_3$): δ 181.4, 146.0, 134.6, 129.7, 128.3, 128.2, 126.6, 126.0, 126.0, 78.7, 78.6, 70.9, 70.7 – 70.3 (m), 51.7, 49.5, 49.5, 27.6, 27.6.
IR (ATR, cm^{-1}): 3152 (br), 2957, 2934, 2865, 2843, 1599, 1570, 1487, 1440, 1357.
HRMS: (DART) m/z: $[M+H]^+$ Calculated for $C_{19}H_{21}NOD$ 281.1759; Found 281.1764
$[\alpha]_{20}^{D}$**:** (c = 0.40, CH_2Cl_2) +143.2
HPLC: IA, 1 mL/min, 5% IPA/Hexane

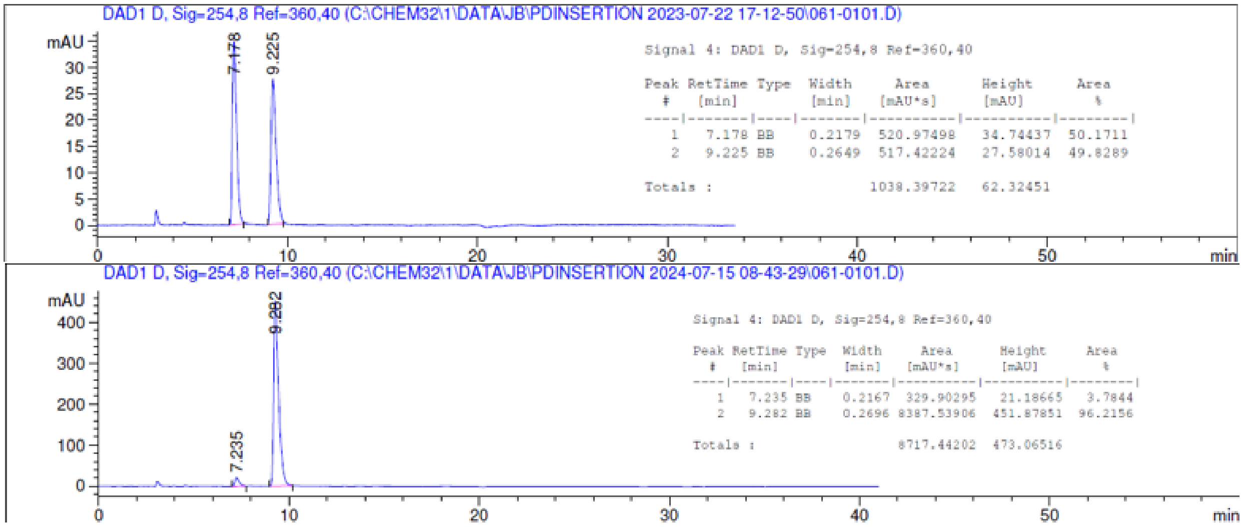

(R)-((2R,5S)-4,4-dimethyl-2,5-diphenylpyrrolidin-2-yl)methan-d-ol (INT-S10)

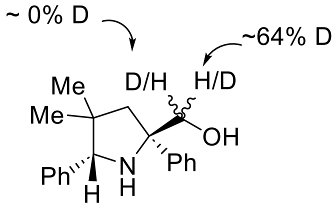

Synthesized according to **GP8** on a 0.65 mmol scale. Purified by silica gel column chromatography (0% → 40% EtOAc/Pentane) to give **INT-S10** as a beige sap (114 mg, 62% yield (89% yield brsm), >20:1 dr). Stereochemistry assigned in analogy to the crystallography data obtained from **3p** and **3s-Ni**, in concert with 2D NMR experiments.

^{1}H NMR (600 MHz, $CDCl_3$): δ 7.68 – 7.65 (m, 2H), 7.54 – 7.51 (m, 2H), 7.42 – 7.37 (m, 2H), 7.37 – 7.32 (m, 2H), 7.30 – 7.23 (m, 2H), 4.16 (s, 1H), 3.74 – 3.65 (m, 2H, (~64% D)), 2.91 (s (br), 1H), 2.08 (dd, *J* = 12.8, 1.7 Hz, 1H), 1.96 (dd, *J* = 12.9, 2.1 Hz, 1H), 1.04 (s, 3H), 0.50 (s, 3H).
^{13}C NMR (101 MHz, $CDCl_3$): δ 147.1, 140.7, 128.3, 127.9, 127.7, 127.0, 126.6, 126.4, 70.6, 70.4 – 70.2 (m), 70.0, 64.5, 64.4, 51.7, 42.0, 26.7, 23.5.
HRMS: (DART) m/z: $[M+H]^+$ Calculated for $C_{19}H_{23}NOD$ 283.1915; Found 283.19235

(1R,5S,7aR)-6,6-dimethyl-5,7a-diphenyltetrahydro-1H,3H-pyrrolo[1,2-c]oxazol-3-one-1-d (5)

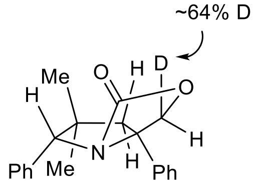

Synthesized according to **GP8** on a 0.37 mmol scale. Purified by trituration with pentanes to give **5** as a white solid (54 mg, 47% yield, >20:1 dr). Stereochemistry assigned in analogy to the crystallography data obtained from **3p** and **3s-Ni**, in concert with 2D NMR experiments.

^{1}H NMR (600 MHz, $CDCl_3$): δ 7.58 – 7.55 (m, 2H), 7.48 – 7.43 (m, 2H), 7.36 – 7.33 (m, 1H), 7.29 – 7.21 (m, 5H), 4.69 (s, 1H), 4.50 – 4.46 (m, 1H (~64% D)), 4.23 – 4.19 (m, 1H), 2.76 – 2.72 (m, 1H), 2.33 – 2.29 (m, 1H), 1.30 (s, 3H), 0.33 (s, 3H).
^{13}C NMR (101 MHz, $CDCl_3$): δ 162.6, 145.5, 138.2, 129.1, 128.2, 127.7, 127.3, 127.1, 124.7, 80.6, 80.5 – 79.9 (m), 77.4, 77.1, 76.7, 74.4, 70.2, 70.1, 51.5, 47.0, 29.0, 24.5.
IR (ATR, cm^{-1}): 3059, 3029, 2968, 2870, 1756, 1494, 1449, 1348, 1271, 1057
HRMS: (DART) m/z: $[M+H]^+$ Calculated for $C_{20}H_{21}NO_2D$ 309.1708; Found 309.1702
MP: 214-216 °C
$[\alpha]^{D}_{20}$**:** (c = 0.31, CH_2Cl_2) -154.72

Nickel Complex (3s-Ni):

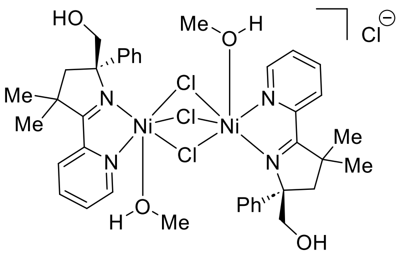

Synthesized according to **GP9** on a 0.036 mmol (10 mg) scale. **3s-Ni** appeared as green crystals from a slow diffusion of pentanes into methanol.

3s-Ni X-Ray Structure:

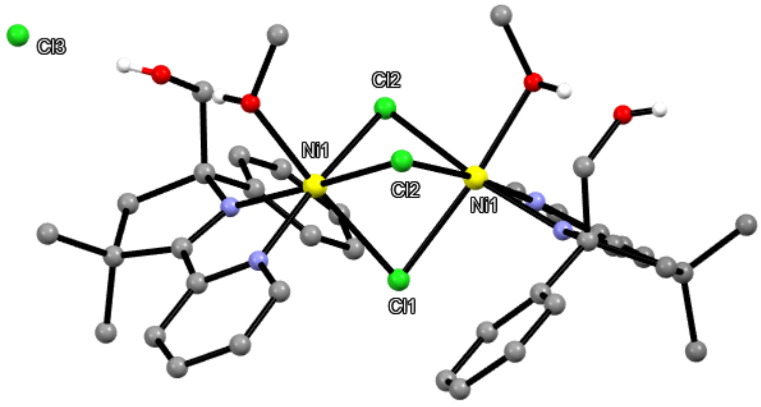

HRMS: (+ESI, Mobile phase 1:1 (v:v) MeOH:0.1% Aq. formic acid)

Ion Species "A"

Target Ion Species

Ion Species	m/z	Ionic Formula
(M+H)+	617.2412	C36 H39 N4 Ni O2

MFG Calculator Results

Target m/z	Ionic Formula	Calc m/z	+/- (mDa)	+/- (ppm)	MFG Score
617.2412	C35 H43 Ni O6	617.2408	0.4	0.6	76.93
617.2412	C36 H39 N4 Ni O2	617.2421	-0.9	-1.5	74.95
617.2412	C32 H35 N10 Ni	617.2394	1.8	2.9	73.30
617.2412	C23 H47 N2 Ni O13	617.2426	-1.4	-2.3	71.67
617.2412	C31 H39 N6 Ni O4	617.2381	3.1	5.0	65.29
617.2412	C24 H43 N6 Ni O9	617.2440	-2.8	-4.5	65.24
617.2412	C25 H39 N10 Ni O5	617.2453	-4.1	-6.6	56.69
617.2412	C30 H43 N2 Ni O8	617.2367	4.5	7.3	55.46

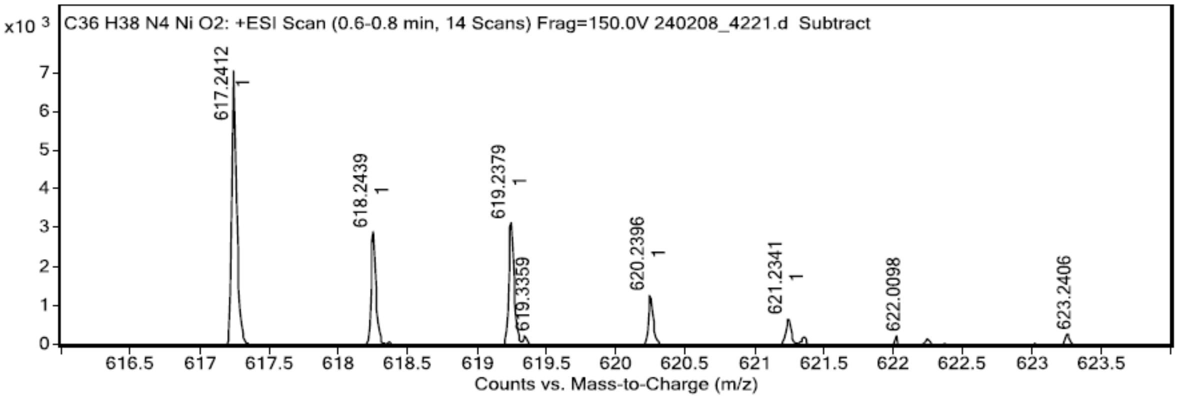

Predicted Isotope Match Table

Isotope	m/z	Calc m/z	Diff (mDa)	Abund (%)	Calc Abund (%)	+/-
1	617.2412	617.2421	-0.9	100.0	100.0	0.0
2	618.2439	618.2453	-1.4	41.2	40.9	-0.3
3	619.2379	619.2395	-1.6	44.7	47.1	2.4

Ion Species "B"

Target Ion Species

Ion Species	*m/z*	*Ionic Formula*
(M+H)+	383.0896	C19 H21 N2 Ni O3

MFG Calculator Results

Target m/z	*Ionic Formula*	*Calc m/z*	*+/- (mDa)*	*+/- (ppm)*	*MFG Score*
383.0896	C19 H21 N2 Ni O3	383.0900	-0.4	-1.0	96.72
383.0896	C15 H17 N8 Ni O	383.0873	2.3	6.0	85.94
383.0896	C14 H21 N4 Ni O5	383.0860	3.6	9.4	72.20
383.0896	C8 H21 N8 Ni O6	383.0932	-3.6	-9.4	63.69

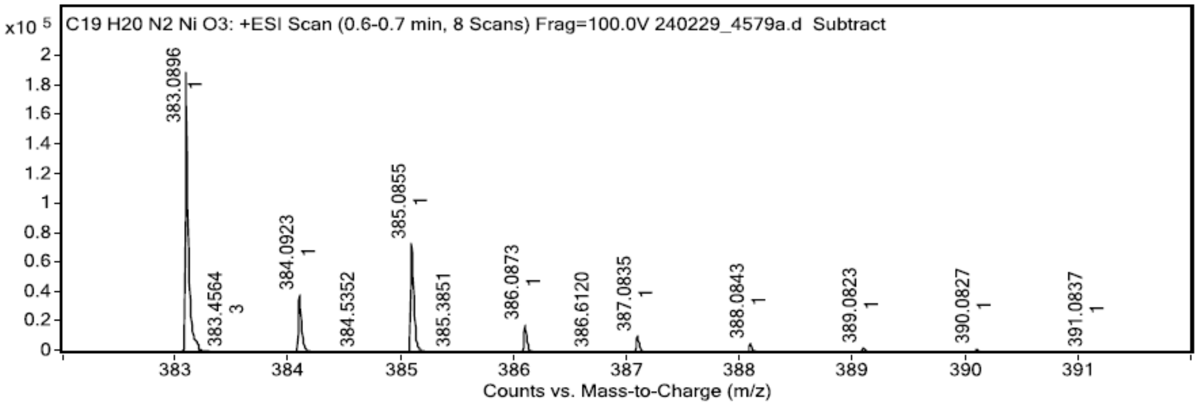

Predicted Isotope Match Table

Isotope	*m/z*	*Calc m/z*	Diff (mDa)	Abund (%)	Calc Abund (%)	+/-
1	383.0896	383.0900	-0.4	100.0	100.0	0.0
2	384.0923	384.0932	-0.9	19.9	21.6	1.7
3	385.0855	385.0862	-0.7	38.2	41.4	3.2
4	386.0873	386.0884	-1.1	9.5	10.3	0.8
5	387.0835	387.0847	-1.2	5.5	6.8	1.3
6	388.0843	388.0870	-2.7	3.0	1.3	-1.7
7	389.0823	389.0834	-1.1	1.5	1.5	0.0
8	390.0827	390.0861	-3.4	1.3	0.3	-1.0

Ion Species "C"

Target Ion Species

Ion Species	m/z	Ionic Formula
(M+H)+	373.0609	C18 H20 Cl N2 Ni O

MFG Calculator Results

Target m/z	Ionic Formula	Calc m/z	+/- (mDa)	+/- (ppm)	MFG Score
373.0609	C18 H20 Cl N2 Ni O	373.0612	-0.3	-0.8	97.93
373.0609	C13 H20 Cl N4 Ni O3	373.0572	3.7	9.9	67.15
373.0609	C7 H20 Cl N8 Ni O4	373.0644	-3.5	-9.4	65.94

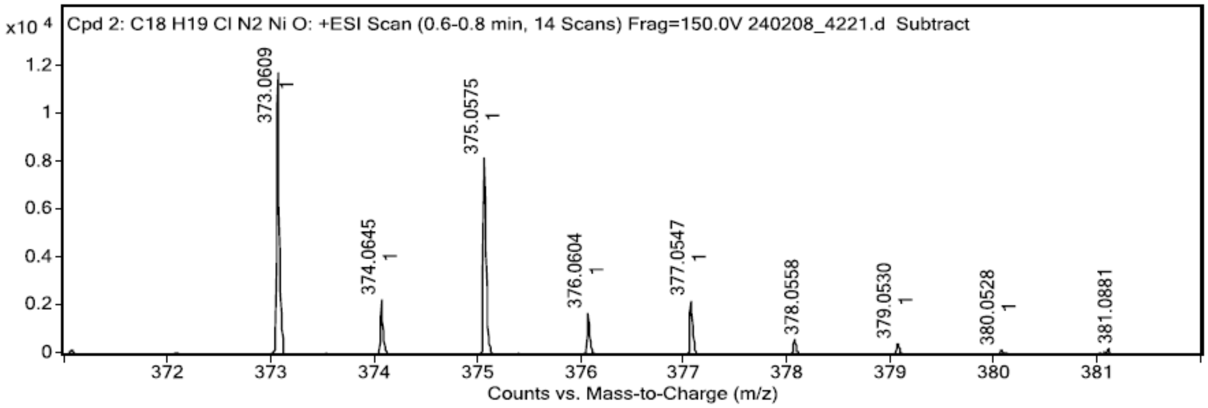

Predicted Isotope Match Table

Isotope	m/z	Calc m/z	Diff (mDa)	Abund (%)	Calc Abund (%)	+/-
1	373.0609	373.0612	-0.3	100.0	100.0	0.0
2	374.0645	374.0644	0.1	19.5	20.5	1.0
3	375.0575	375.0577	-0.2	70.0	72.7	2.7
4	376.0604	376.0603	0.1	14.9	16.3	1.4
5	377.0547	377.0547	0.0	19.1	19.6	0.5

Possible Ion species:

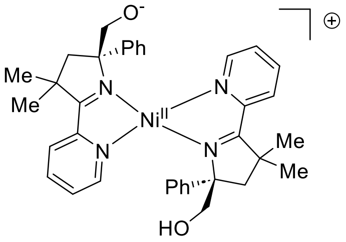

Ion Species "A"
$C_{36}H_{39}N_4NiO_2$

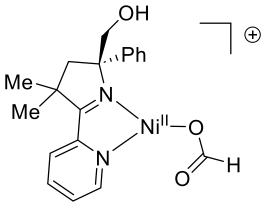

Ion Species "B"
$C_{19}H_{21}N_2NiO_3$

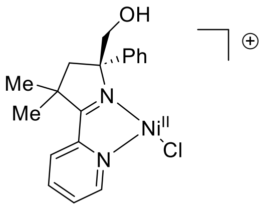

Ion Species "C"
$C_{18}H_{20}ClN_2NiO$

E/Z Oxime Ester Determination of Substrate 1a

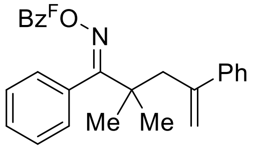

1a

^{1}H NMR

$CDCl_3$, 600 MHz

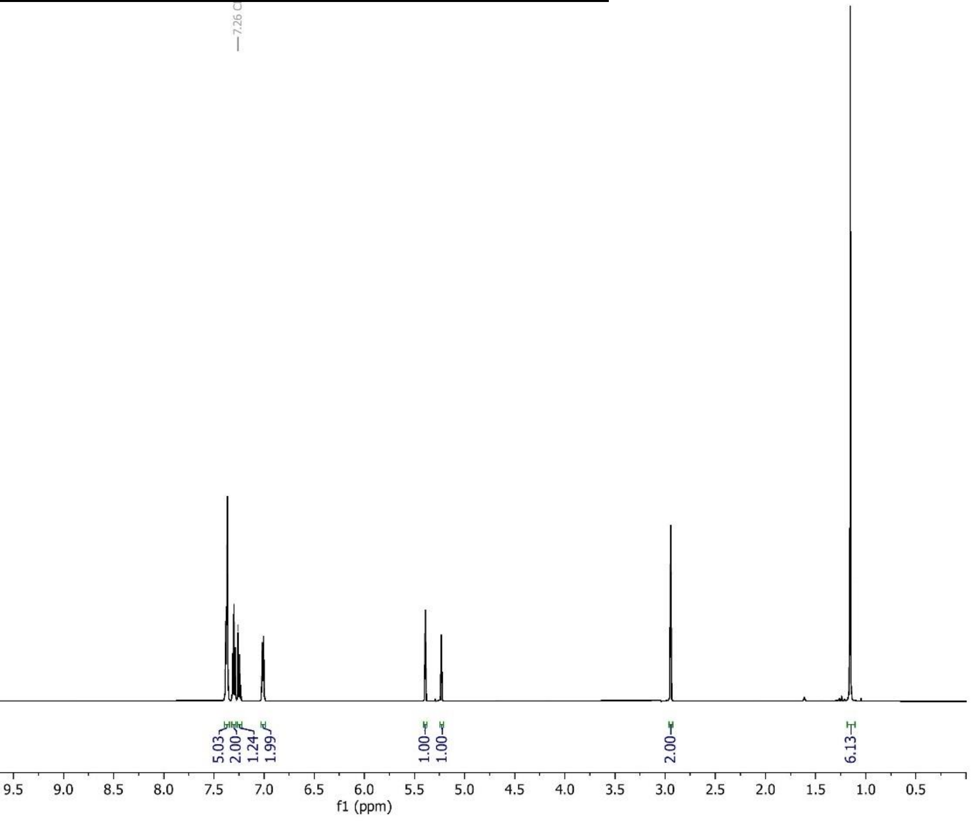

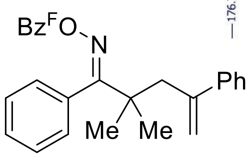

1a

^{13}C NMR

$CDCl_3$, 151 MHz

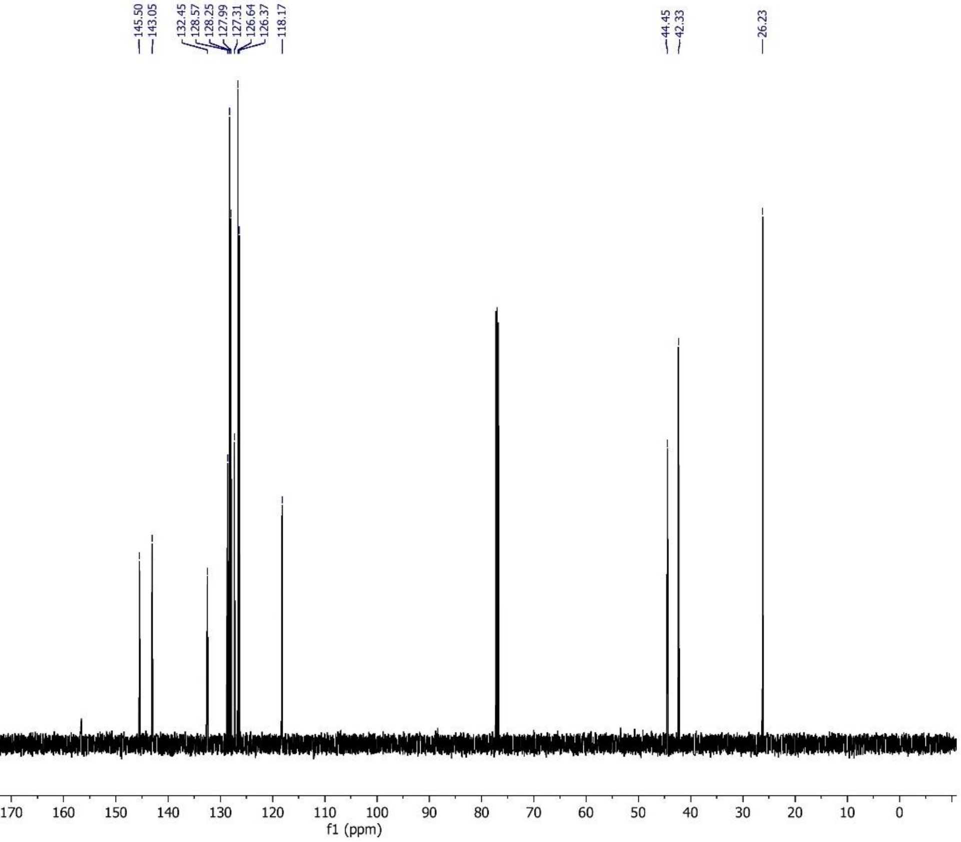

1a, **HMBC**

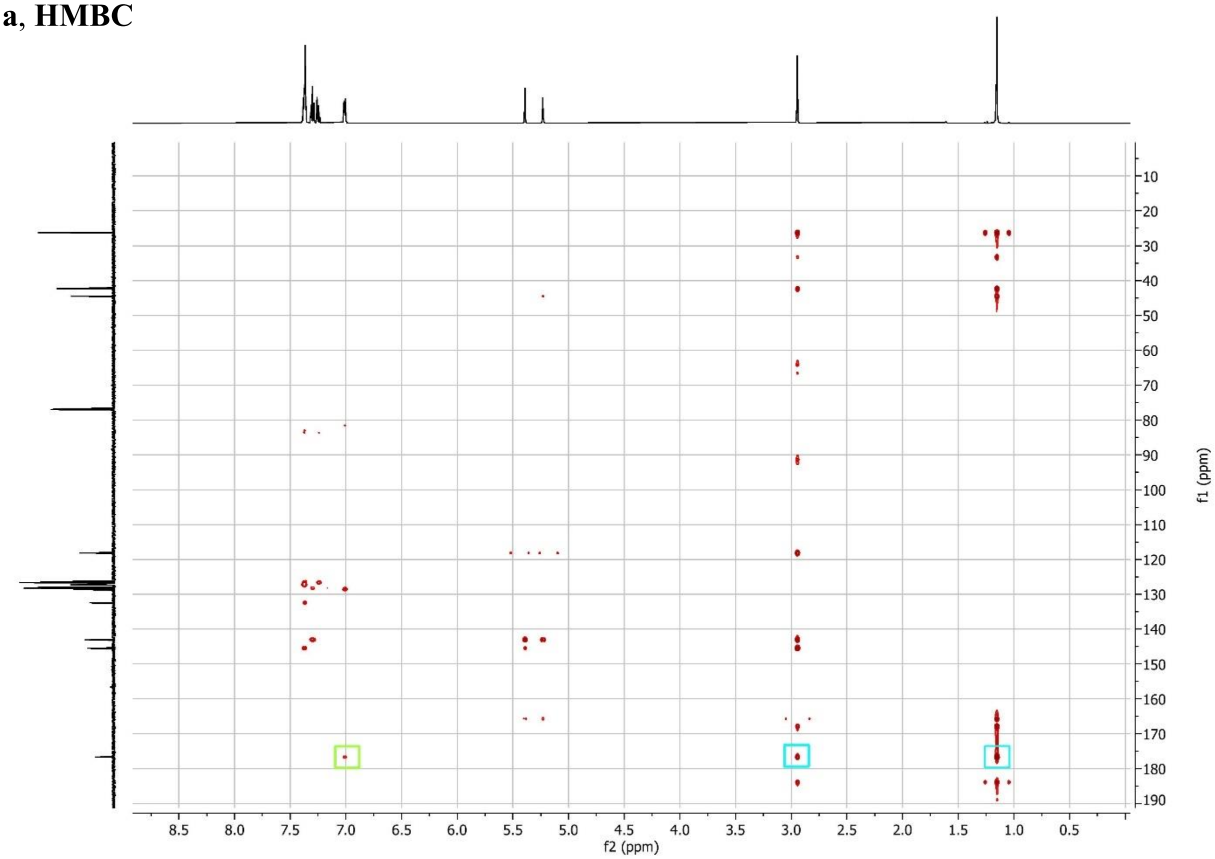

Key HMBC Signals:

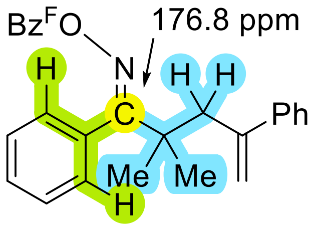

1a, HOESY

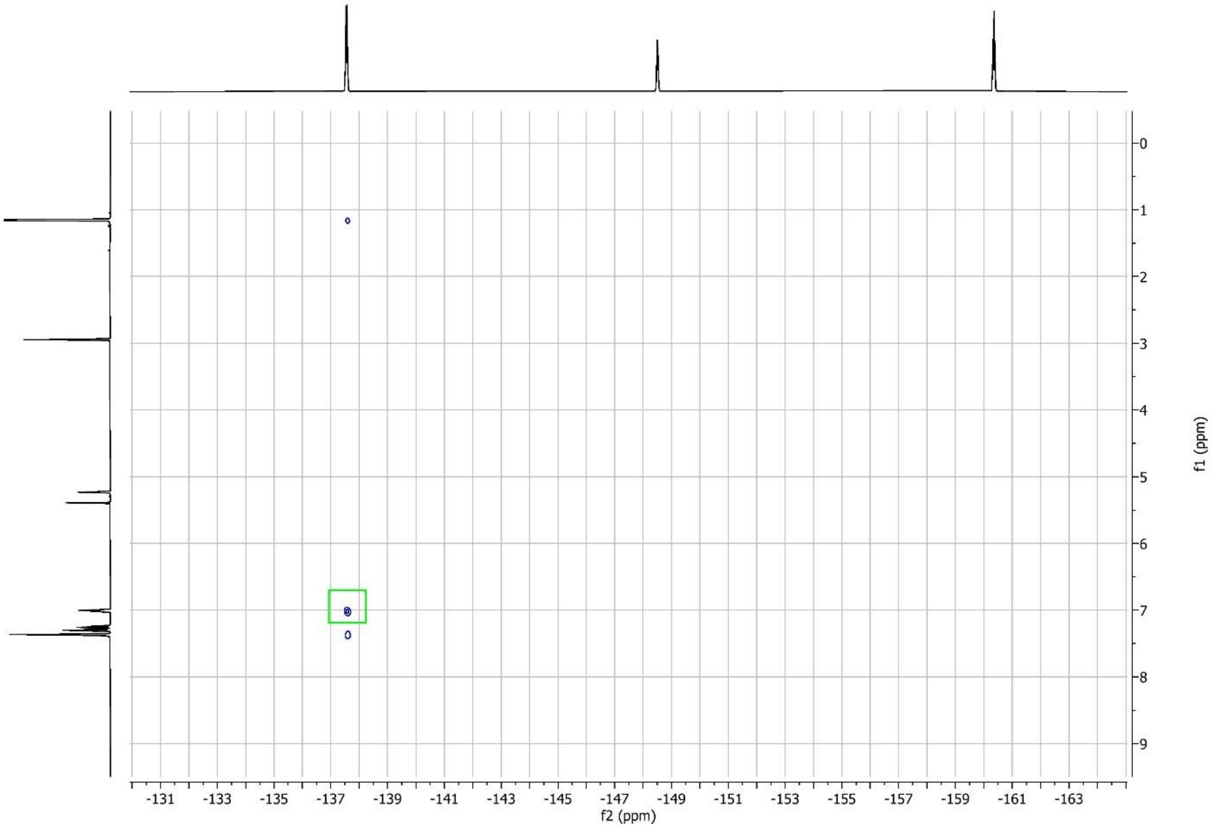

Key HOESY Signals:

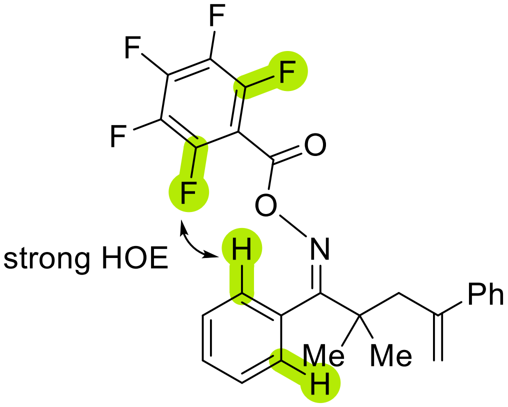

Computational Details

All geometry optimizations were performed at the wB97XD[7] level of theory without any constraints at 298.15 K and 1 atm, using an ultrafine integration grid. The wB97XD functional contains a version of Grimme's D2 dispersion model.[8] The Berny algorithm was used for geometry optimizations.[9] The 6-31g* Pople basis set[10,11] was used for main group elements. The LANL2DZ basis set and its effective core potential (ECP) were used for copper.[12,13,14,15] All frequency calculations were performed at the same level of theory for all intermediates and transition states to confirm minima or first-order saddle points (no imaginary frequencies or exactly one imaginary frequency, respectively). Single-point energies were re-evaluated using the B3LYP[16] functional with the addition of Grimme's D3 dispersion with the original damping function.[17] The 6-311+g**[18,19,20,21,22,23,24] basis set was used for main group elements and the SDD basis set and its associated ECP was used for copper.[25,26,27,28] The effect of solvation was evaluated using the SMD model utilizing default parameters for THF.[29] Solution phase-corrected energies were obtained by adding the respective gas phase thermodynamic contributions to the single-point energies. Gibbs free energies are reported relative to **I** ([(*S*,*S*)-bdpp]CuBpin complex) and are reported in kcal mol^{-1}. The Gibbs free energies were converted to standard state by addition of 1.89 kcal mol^{-1} to all species (based on RT ln (c/c_0) at 298.15 K).[30] Grimme's quasi-harmonic approximation was used with a frequency cutoff value of 50 cm^{-1} to correct for the effect of small frequency vibrational modes.[31] All calculations were performed using the Gaussian16 package.[32] The images of computed structures were created with Chemcraft (hydrogen atoms were omitted for clarity).[33]

Non-covalent interactions were assessed using the Multiwfn software.[34]

The basis sets used for the optimization and single-point energy calculations were selected based on a previous report utilizing DFT to study copper catalysis.[36] Although the results of our calculations using the B3LYP functional correlate well with the experimental observations, we recognize that there are limitations in its accuracy due to numerous errors, such as basis set superposition error (BSSE).[37, 38]

NCI Plots

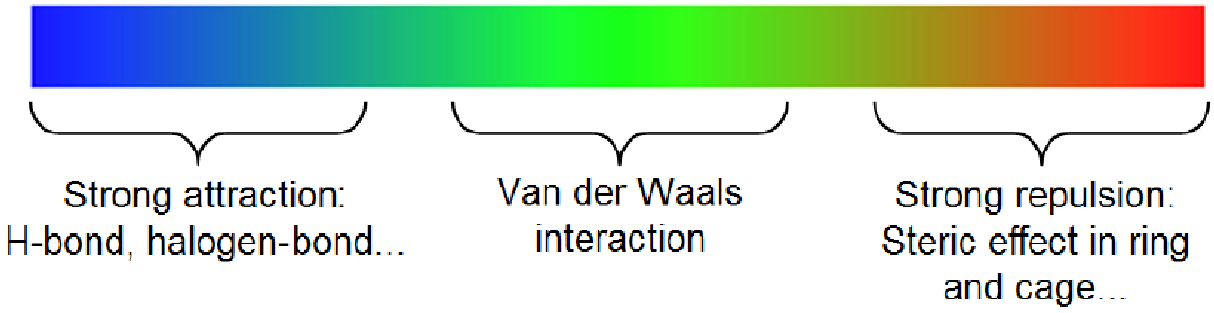

III-TS-inv

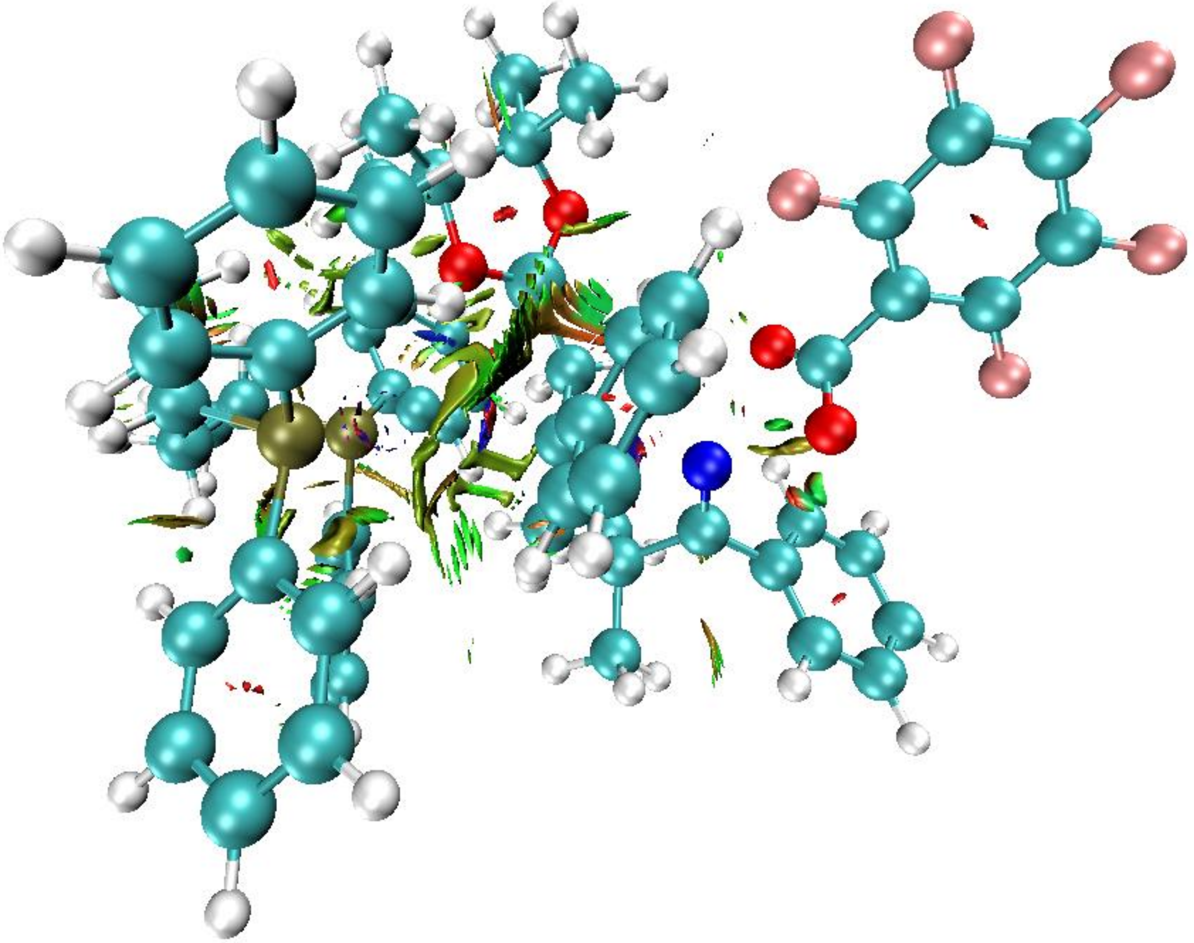

III-TS-ret

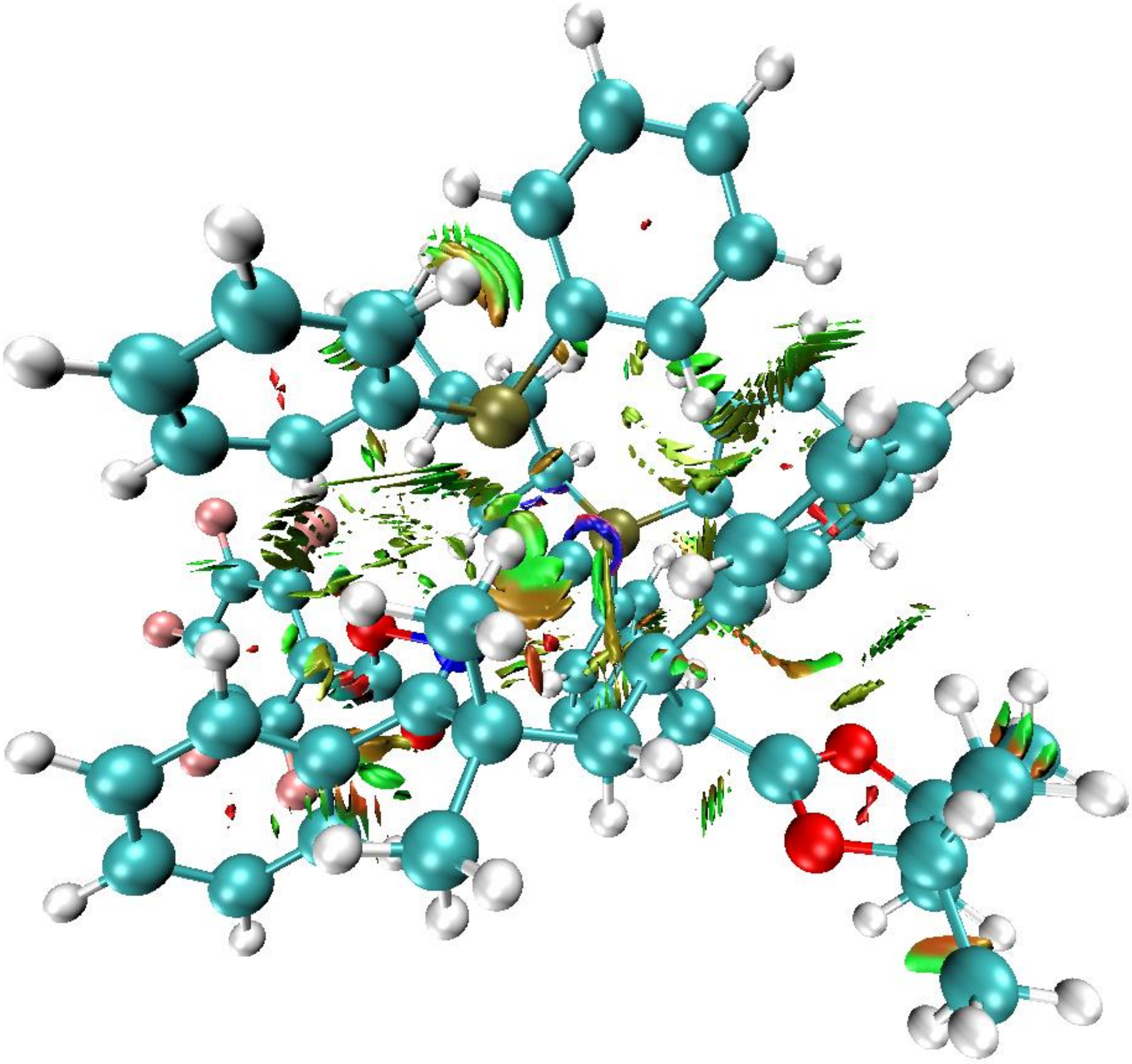

III-TS-ret – Substrate CH_3 to Ligand Ph C–H π-Interaction

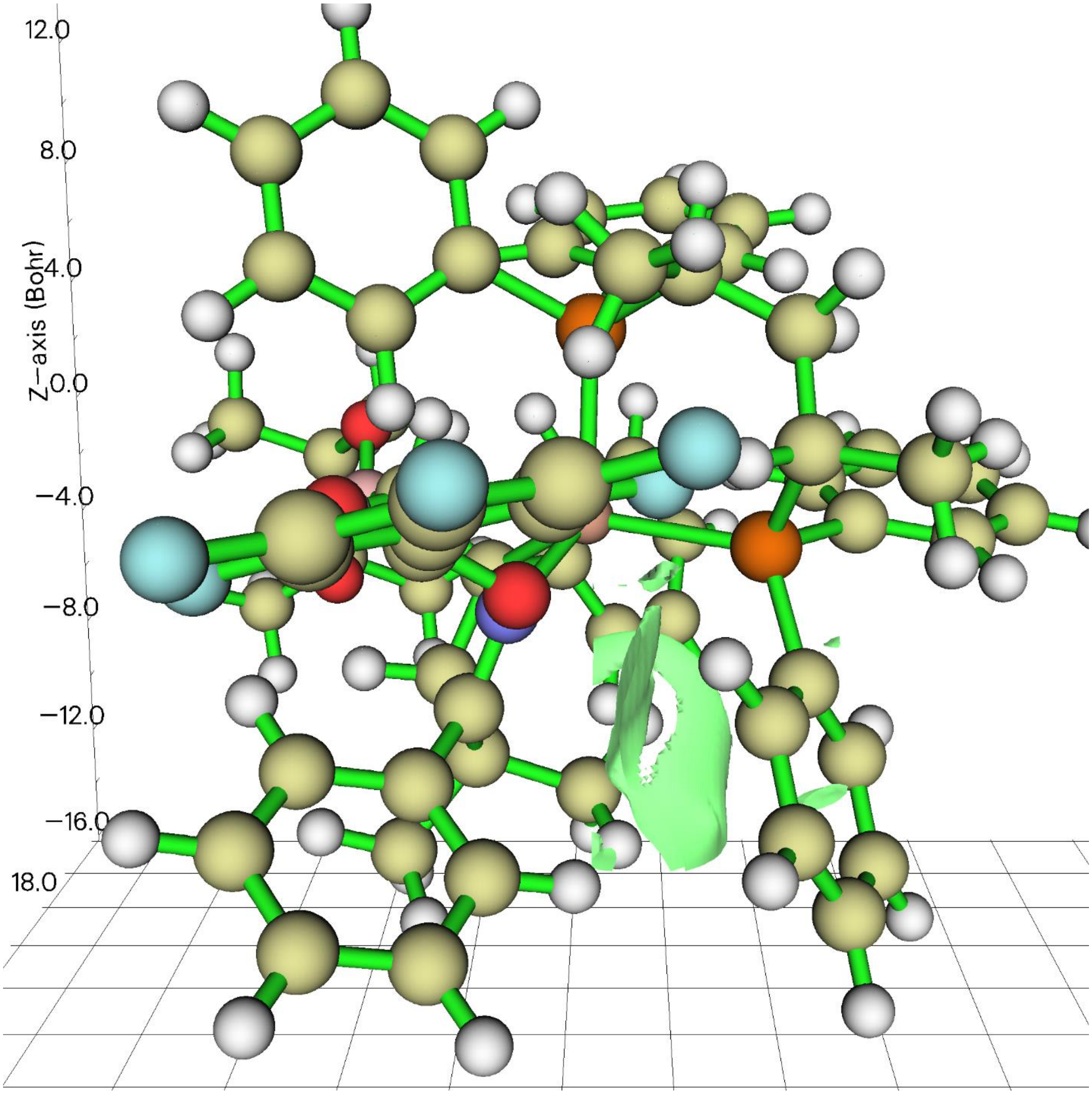

III-TS-ret – Ligand C–H to Substrate Ph C–H π-Interaction

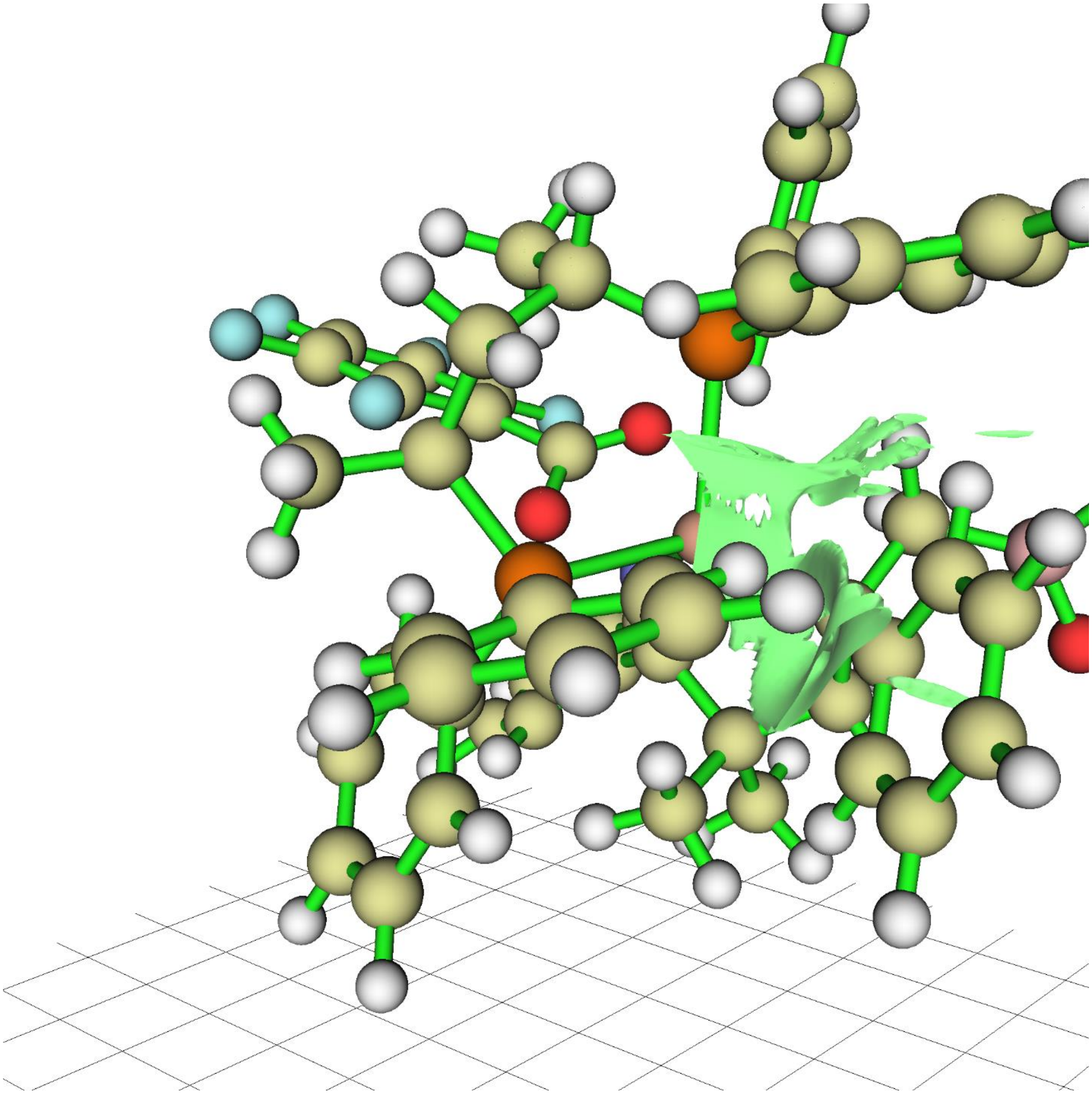

Computed Energy Components

	E(SCF)	ZPE	TS	G(sol)
	au<br>6-311+g**/SDD	au<br>6-31g*/LANL2DZ	au<br>6-31g*/LANL2DZ	au<br>6-311+g**/SDD
**I**	-2415.11259	0.701336	0.108964	-2414.520222
**1a**	-1707.66403	0.409667	0.085973	-1707.340340
**II-*si***	-4122.803907	1.115186	0.167479	-4121.8562
**II-*re***	-4122.798754	1.113645	0.165603	-4121.850712
**II-*si*-TS**	-4122.772378	1.112788	0.165681	-4121.825271
**II-*re*-TS**	-4122.778172	1.11064	0.163538	-4121.83107
**III-(*S*)**	-4122.816909	1.1149	0.167066	-4121.869075
**III-(*R*)**	-4122.826879	1.114046	0.168326	-4121.881159
**III-TS-inv**	-4122.800104	1.112165	0.168951	-4121.85689
**III-TS-ret**	-4122.823724	1.113627	0.166584	-4121.876681
**IV-(*S*)**	-1202.517623	0.532014	0.078326	-1202.063935
**IV-(*R*)**	-1202.518161	0.531683	0.078289	-1202.064767
**[Cu]–OBz^F**	-2920.416327	0.582914	0.114704	-2919.948117

Cartesian Coordinates

I

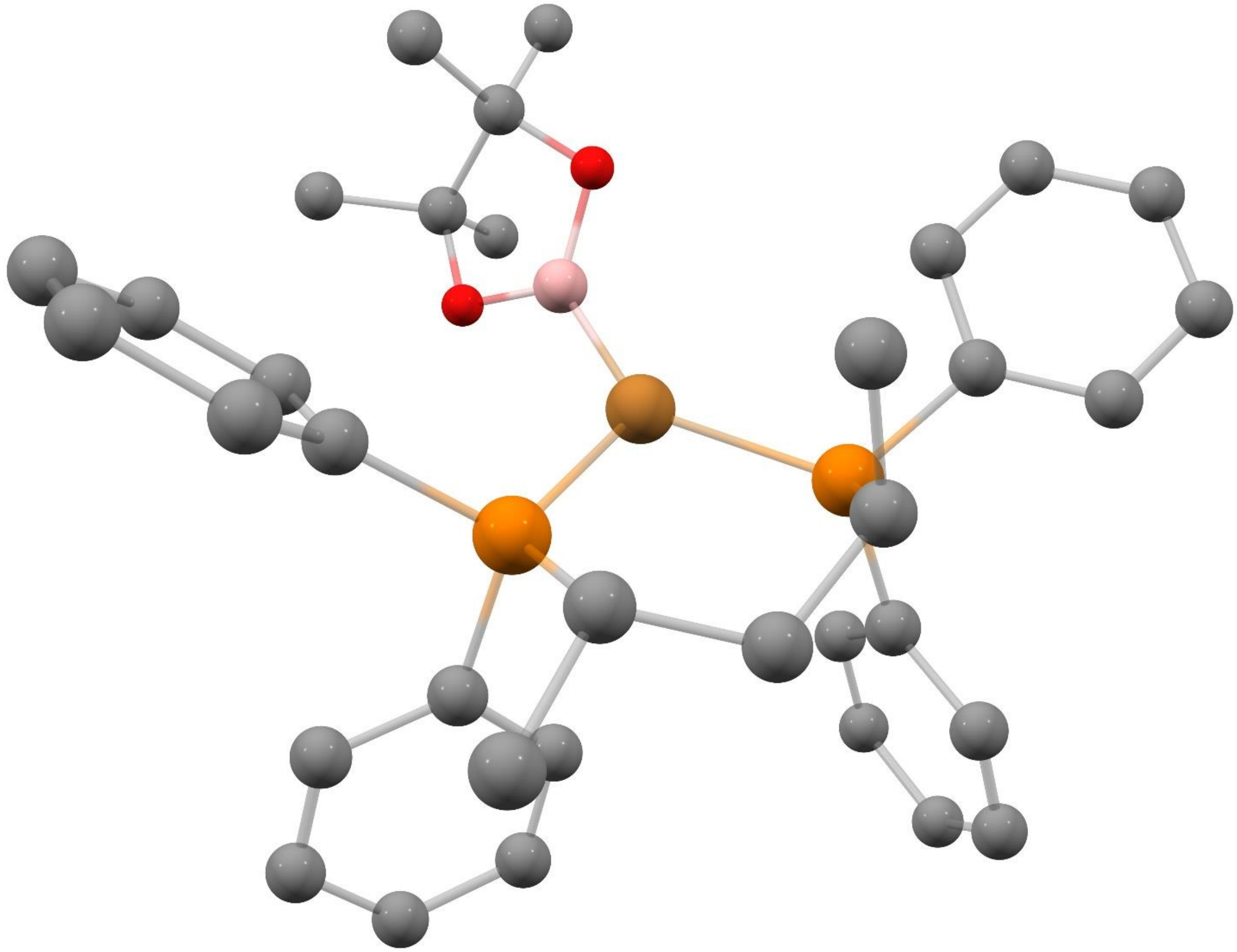

E$_{SCF}$ (wB97X-D/6-31g*-LANL2DZ): -2412.78707530 Hartree
E$_{SCF}$ (wB97X-D/6-311+g-SDD/SMD(THF)):** -2415.11259371 Hartree
Entropy (wB97X-D/6-31g*-LANL2DZ): 251.794 cal mol^{-1} K^{-1}
Thermal Correction to the Energy (wB97X-D/6-31g*-LANL2DZ): 0.742577 Hartree particle^{-1}
Thermal Correction to the Enthalpy (wB97X-D/6-31g*-LANL2DZ): 0.743521 Hartree particle^{-1}
Thermal Correction to the Gibbs Free Energy (wB97X-D/6-31g*-LANL2DZ): 0.623885 Hartree particle^{-1}
Nuclear Repulsion Energy: 5805.2578855429 Hartree

C -1.580221000000 0.457175000000 -3.113830000000

H	-1.688576000000	-0.099031000000	-4.052563000000
H	-1.921178000000	1.481886000000	-3.286289000000
H	-0.512876000000	0.502764000000	-2.866334000000
C	-2.395014000000	-0.212948000000	-2.000408000000
H	-3.456024000000	-0.087579000000	-2.254741000000
C	-2.136088000000	-1.736547000000	-1.893801000000
H	-2.551239000000	-2.125972000000	-0.954333000000
H	-2.726569000000	-2.210400000000	-2.690756000000
C	-0.695794000000	-2.270366000000	-2.058570000000
H	-0.268328000000	-1.830417000000	-2.968223000000
C	-0.750077000000	-3.795080000000	-2.221161000000
H	0.244692000000	-4.248155000000	-2.256417000000
H	-1.283206000000	-4.252815000000	-1.381353000000
H	-1.279103000000	-4.060276000000	-3.144453000000
P	-2.092813000000	0.694235000000	-0.398550000000
P	0.429644000000	-1.688352000000	-0.678855000000
C	-2.690785000000	2.382089000000	-0.777144000000
C	-1.746617000000	3.413515000000	-0.793284000000
C	-4.027038000000	2.664089000000	-1.081012000000
C	-2.137067000000	4.710274000000	-1.122312000000
C	-4.413577000000	3.959609000000	-1.404750000000
C	-3.466617000000	4.983298000000	-1.428507000000
H	-0.709819000000	3.199213000000	-0.538972000000
H	-4.771556000000	1.872019000000	-1.051157000000
H	-1.398766000000	5.506404000000	-1.132778000000
H	-5.453207000000	4.173560000000	-1.635307000000
H	-3.769948000000	5.995369000000	-1.680740000000
C	-3.331673000000	0.020897000000	0.771198000000
C	-3.035965000000	0.153786000000	2.134345000000
C	-4.511406000000	-0.631661000000	0.398939000000
C	-3.903002000000	-0.346069000000	3.100959000000
C	-5.375415000000	-1.138930000000	1.365842000000
C	-5.073533000000	-0.996311000000	2.717482000000
H	-2.110419000000	0.640963000000	2.433488000000
H	-4.762116000000	-0.759208000000	-0.650363000000
H	-3.658994000000	-0.234432000000	4.153105000000
H	-6.285019000000	-1.648413000000	1.061702000000
H	-5.747523000000	-1.394524000000	3.470141000000
C	0.187810000000	-2.973716000000	0.602852000000
C	-0.879270000000	-2.803143000000	1.491758000000
C	0.988904000000	-4.114097000000	0.714723000000
C	-1.157618000000	-3.765088000000	2.457942000000
C	0.718153000000	-5.068966000000	1.690159000000
C	-0.358004000000	-4.899994000000	2.559038000000
H	-1.493909000000	-1.909235000000	1.436795000000
H	1.829275000000	-4.252765000000	0.040482000000
H	-1.992680000000	-3.616346000000	3.136260000000
H	1.349630000000	-5.948953000000	1.771345000000

H	-0.566246000000	-5.648130000000	3.318348000000
C	2.137296000000	-1.962179000000	-1.279959000000
C	2.478307000000	-2.537309000000	-2.507516000000
C	3.161416000000	-1.491281000000	-0.444043000000
C	3.814274000000	-2.654757000000	-2.887650000000
C	4.493030000000	-1.625344000000	-0.820065000000
C	4.823959000000	-2.206421000000	-2.042853000000
H	1.710269000000	-2.893435000000	-3.186638000000
H	2.916301000000	-0.986484000000	0.487501000000
H	4.062146000000	-3.098965000000	-3.847338000000
H	5.273566000000	-1.259497000000	-0.159194000000
H	5.864471000000	-2.299767000000	-2.339791000000
Cu	0.148127000000	0.501319000000	0.199212000000
C	3.367261000000	2.979176000000	3.092508000000
H	3.350821000000	2.264611000000	3.921131000000
H	4.094107000000	3.765535000000	3.324891000000
H	2.372682000000	3.428915000000	3.014624000000
C	3.724728000000	2.232109000000	1.803798000000
C	3.494285000000	3.086183000000	0.518292000000
C	3.712290000000	4.584246000000	0.693783000000
H	3.035544000000	5.000725000000	1.443486000000
H	4.744976000000	4.795735000000	0.996156000000
H	3.523331000000	5.097780000000	-0.254481000000
C	4.297644000000	2.580351000000	-0.685820000000
H	3.932789000000	3.082537000000	-1.587086000000
H	5.368274000000	2.789507000000	-0.581087000000
H	4.158658000000	1.502771000000	-0.822649000000
C	5.115647000000	1.621242000000	1.928032000000
H	5.334699000000	0.960132000000	1.085757000000
H	5.884740000000	2.401747000000	1.968594000000
H	5.178813000000	1.030939000000	2.847887000000
O	2.109826000000	2.839262000000	0.233856000000
O	2.765837000000	1.177599000000	1.642255000000
B	1.744551000000	1.604664000000	0.782522000000

II-*si*

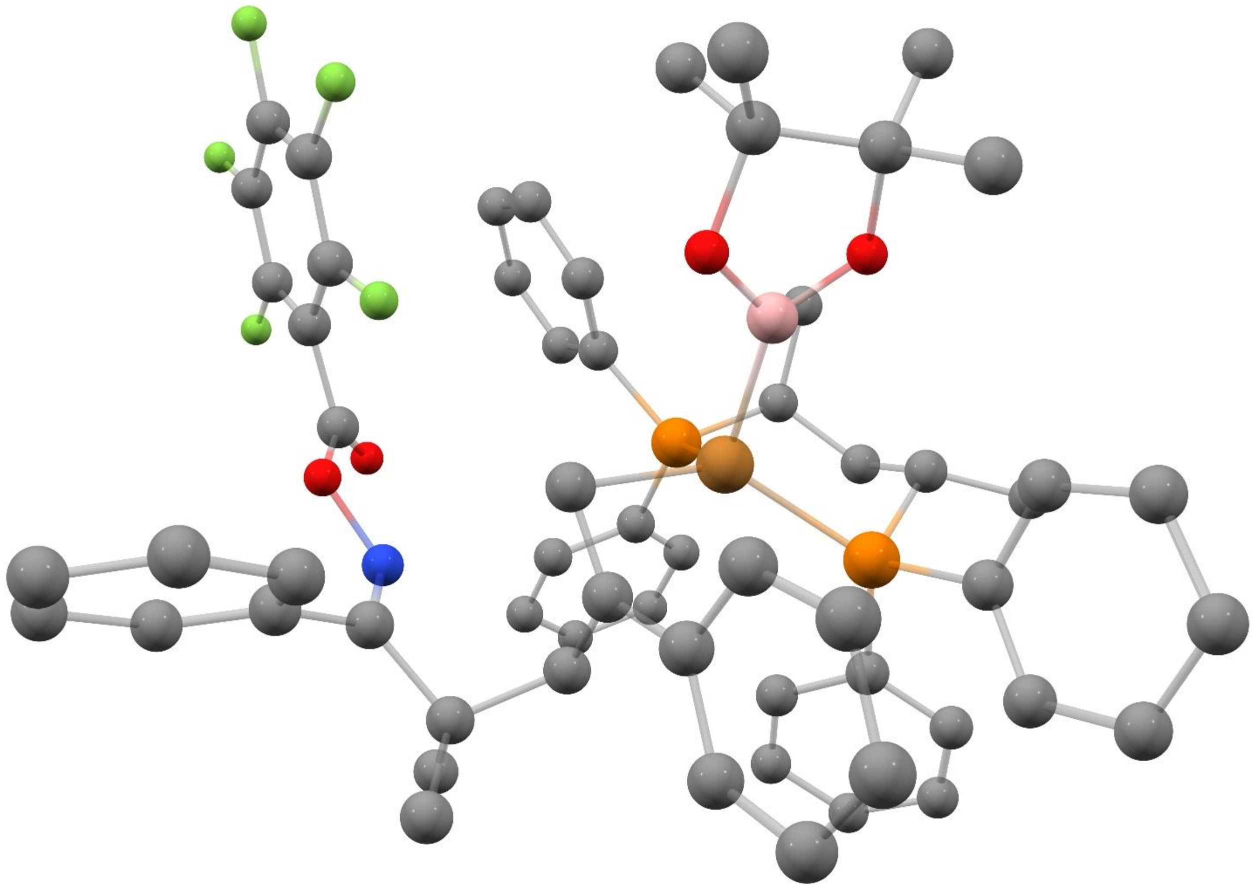

E$_{SCF}$ (wB97X-D/6-31g*-LANL2DZ): -4119.40322730 Hartree
E$_{SCF}$ (wB97X-D/6-311+g-SDD/SMD(THF)):** -4122.80390671 Hartree
Entropy (wB97X-D/6-31g*-LANL2DZ): 382.054 cal mol^{-1} K^{-1}
Thermal Correction to the Energy (wB97X-D/6-31g*-LANL2DZ): 1.186819 Hartree particle^{-1}
Thermal Correction to the Enthalpy (wB97X-D/6-31g*-LANL2DZ): 1.187763 Hartree particle^{-1}
Thermal Correction to the Gibbs Free Energy (wB97X-D/6-31g*-LANL2DZ): 1.006237 Hartree particle^{-1}
Nuclear Repulsion Energy: 15421.9820204113 Hartree

C	2.024723000000	-0.460115000000	2.639707000000
C	2.247302000000	0.897627000000	2.934888000000
C	2.930295000000	-1.389648000000	3.170513000000
C	3.305191000000	1.293647000000	3.741917000000
C	3.981294000000	-0.992850000000	3.991348000000
C	4.172869000000	0.351463000000	4.286773000000
H	1.607234000000	1.661006000000	2.506983000000
H	2.818581000000	-2.445615000000	2.955643000000
H	3.460201000000	2.351392000000	3.933525000000

H	4.656366000000	-1.743182000000	4.393536000000
H	5.001021000000	0.665310000000	4.914975000000
C	0.875846000000	-0.877007000000	1.783727000000
C	-0.105848000000	0.013485000000	1.456489000000
H	-0.174422000000	1.009219000000	1.876095000000
C	0.708940000000	-2.333476000000	1.377151000000
H	0.297117000000	-2.361286000000	0.365876000000
H	1.676594000000	-2.841622000000	1.321627000000
C	-0.238278000000	-3.200858000000	2.266231000000
C	-1.636671000000	-2.556047000000	2.265310000000
H	-0.971046000000	-0.306424000000	0.887478000000
C	-2.296313000000	-2.072472000000	3.510604000000
C	-3.583322000000	-1.252893000000	5.849458000000
C	-3.430488000000	-2.750046000000	3.966655000000
C	-1.809564000000	-0.981524000000	4.233319000000
C	-2.457589000000	-0.571869000000	5.394370000000
C	-4.067705000000	-2.345172000000	5.134280000000
H	-3.812565000000	-3.594206000000	3.399910000000
H	-0.931783000000	-0.449198000000	3.883041000000
H	-2.076604000000	0.282401000000	5.945614000000
H	-4.945591000000	-2.880943000000	5.482672000000
H	-4.082325000000	-0.933307000000	6.759448000000
C	-0.339187000000	-4.595188000000	1.619177000000
H	0.649522000000	-5.067645000000	1.602387000000
H	-0.707584000000	-4.527922000000	0.592955000000
H	-1.015718000000	-5.241842000000	2.189362000000
C	0.292025000000	-3.367212000000	3.695790000000
H	0.486694000000	-2.412757000000	4.190369000000
H	1.231226000000	-3.929421000000	3.670623000000
H	-0.417452000000	-3.928806000000	4.312792000000
N	-2.180234000000	-2.490005000000	1.109967000000
O	-3.362234000000	-1.724438000000	1.143326000000
C	-3.950156000000	-1.632650000000	-0.070707000000
O	-3.797196000000	-2.391843000000	-0.986562000000
C	-4.827471000000	-0.424100000000	-0.105846000000
C	-6.398556000000	1.879059000000	-0.391858000000
C	-4.450013000000	0.783828000000	0.485167000000
C	-6.008356000000	-0.444978000000	-0.850243000000
C	-6.797369000000	0.688528000000	-0.986683000000
C	-5.223198000000	1.927444000000	0.343579000000
F	-3.307770000000	0.907985000000	1.148029000000
F	-4.812111000000	3.082398000000	0.862595000000
F	-7.134360000000	2.972114000000	-0.540065000000
F	-7.926660000000	0.644382000000	-1.687595000000
F	-6.427100000000	-1.562237000000	-1.427113000000
C	0.942344000000	1.880338000000	-3.770483000000

H	1.597359000000	2.312145000000	-4.537623000000
H	-0.093016000000	2.047420000000	-4.080151000000
H	1.110190000000	2.424529000000	-2.836967000000
C	1.242924000000	0.382474000000	-3.634617000000
H	0.897551000000	-0.101921000000	-4.558389000000
C	2.763901000000	0.111079000000	-3.543414000000
P	0.256953000000	-0.397813000000	-2.244419000000
H	2.962076000000	-0.966471000000	-3.485878000000
H	3.172854000000	0.429428000000	-4.512647000000
C	3.606636000000	0.825324000000	-2.459716000000
H	3.187015000000	1.820316000000	-2.271684000000
C	5.046517000000	0.979067000000	-2.958877000000
P	3.449835000000	-0.032017000000	-0.794855000000
H	5.702028000000	1.406123000000	-2.194079000000
H	5.460466000000	0.012879000000	-3.268618000000
H	5.068413000000	1.643722000000	-3.830772000000
Cu	1.260828000000	0.457128000000	-0.259406000000
B	1.068985000000	2.503432000000	-0.071879000000
H	-2.013471000000	4.269019000000	0.242039000000
C	-1.157466000000	4.699153000000	-0.288579000000
H	-1.365236000000	5.756718000000	-0.484016000000
H	-1.056363000000	4.180682000000	-1.247655000000
C	0.097408000000	4.512299000000	0.571004000000
C	1.421930000000	4.797946000000	-0.205868000000
C	-0.057093000000	5.271044000000	1.883008000000
O	0.207956000000	3.109844000000	0.852391000000
C	1.307606000000	5.843194000000	-1.308474000000
C	2.595043000000	5.141932000000	0.717114000000
O	1.708072000000	3.520087000000	-0.799863000000
H	0.578526000000	5.546510000000	-2.065950000000
H	1.010595000000	6.814610000000	-0.896039000000
H	2.276557000000	5.965009000000	-1.803065000000
H	3.515477000000	5.166988000000	0.124280000000
H	2.467365000000	6.120908000000	1.191142000000
H	2.716284000000	4.382197000000	1.496232000000
H	0.747158000000	5.026586000000	2.581109000000
H	-0.057452000000	6.353917000000	1.711799000000
H	-1.007286000000	5.000149000000	2.354064000000
C	4.124985000000	-1.706254000000	-1.130347000000
C	3.197344000000	-2.749446000000	-1.206740000000
C	5.472428000000	-1.987985000000	-1.396085000000
C	3.592185000000	-4.039667000000	-1.552406000000
C	5.872154000000	-3.278427000000	-1.727310000000
C	4.932506000000	-4.305791000000	-1.810530000000
H	2.151078000000	-2.544478000000	-1.007936000000
H	6.213980000000	-1.197121000000	-1.334403000000

H	2.845939000000	-4.825279000000	-1.625205000000
H	6.920179000000	-3.482666000000	-1.927026000000
H	5.248718000000	-5.310322000000	-2.076800000000
C	4.697429000000	0.801742000000	0.252887000000
C	4.668121000000	2.201717000000	0.320656000000
C	5.599072000000	0.107503000000	1.061998000000
C	5.549356000000	2.886005000000	1.148871000000
C	6.480919000000	0.797043000000	1.890916000000
C	6.465395000000	2.186208000000	1.932092000000
H	3.937110000000	2.756146000000	-0.261160000000
H	5.609973000000	-0.977478000000	1.062245000000
H	5.514591000000	3.971363000000	1.187413000000
H	7.173171000000	0.239712000000	2.515508000000
H	7.154018000000	2.722144000000	2.579078000000
C	-1.441682000000	0.238535000000	-2.550484000000
C	-1.850463000000	1.373532000000	-1.841071000000
C	-2.305502000000	-0.312578000000	-3.502751000000
C	-3.070943000000	1.980811000000	-2.123512000000
C	-3.542418000000	0.273276000000	-3.756036000000
C	-3.920590000000	1.431515000000	-3.080265000000
H	-1.202377000000	1.781518000000	-1.069941000000
H	-2.016025000000	-1.208882000000	-4.043173000000
H	-3.357082000000	2.879517000000	-1.584010000000
H	-4.207823000000	-0.171062000000	-4.490232000000
H	-4.876475000000	1.900764000000	-3.296996000000
C	0.096541000000	-2.158309000000	-2.755261000000
C	-0.772299000000	-2.957584000000	-2.000217000000
C	0.819670000000	-2.759679000000	-3.790170000000
C	-0.917081000000	-4.312391000000	-2.273764000000
C	0.690729000000	-4.122835000000	-4.051109000000
C	-0.176497000000	-4.904483000000	-3.294817000000
H	-1.364202000000	-2.527155000000	-1.198835000000
H	1.496284000000	-2.179274000000	-4.407345000000
H	-1.621030000000	-4.896769000000	-1.688640000000
H	1.268924000000	-4.569694000000	-4.854781000000
H	-0.282498000000	-5.964677000000	-3.505863000000

II-*re*

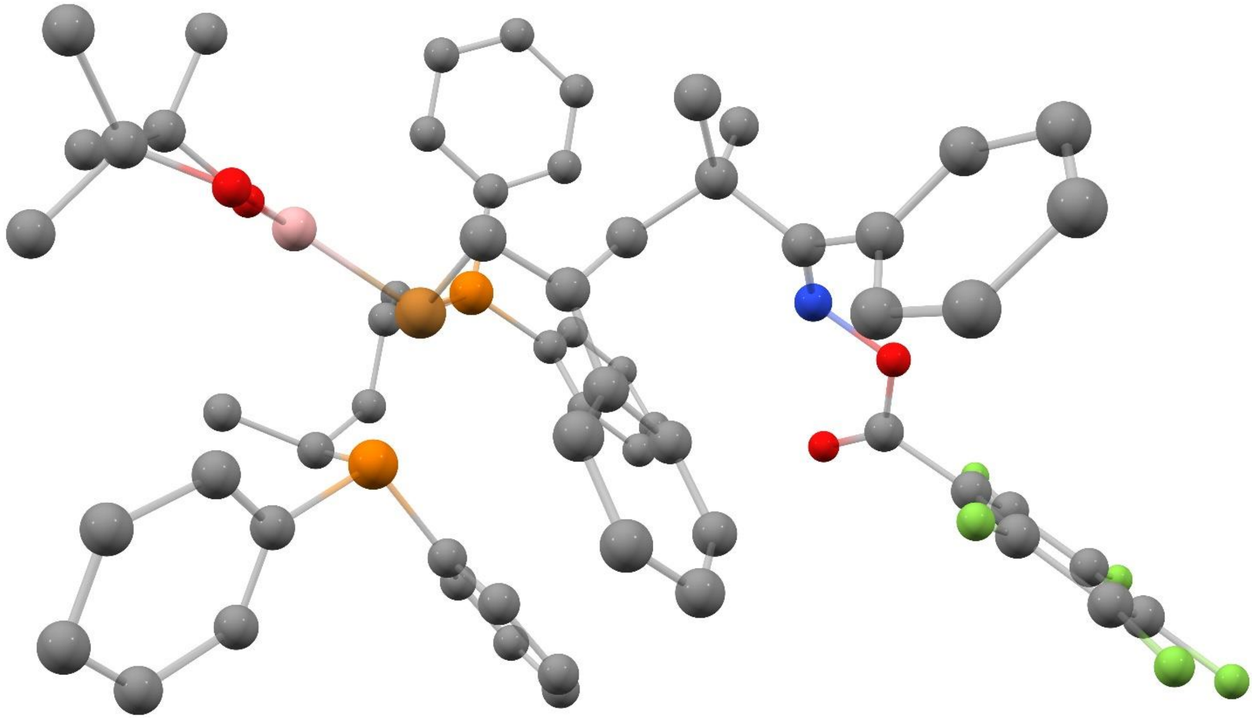

E

E_{SCF} (wB97X-D/6-31g*-LANL2DZ): -4119.38870901 Hartree
E_{SCF} (wB97X-D/6-311+g-SDD/SMD(THF)):** -4122.79875371 Hartree
Entropy (wB97X-D/6-31g*-LANL2DZ): 377.754 cal mol^{-1} K^{-1}
Thermal Correction to the Energy (wB97X-D/6-31g*-LANL2DZ): 1.184168 Hartree particle^{-1}
Thermal Correction to the Enthalpy (wB97X-D/6-31g*-LANL2DZ): 1.185112 Hartree particle^{-1}
Thermal Correction to the Gibbs Free Energy (wB97X-D/6-31g*-LANL2DZ): 1.005629 Hartree particle^{-1}
Nuclear Repulsion Energy: 14873.2583318048 Hartree

C	0.611123000000	0.266654000000	-1.466729000000
C	1.597037000000	0.661484000000	-0.550102000000
C	0.571043000000	0.944004000000	-2.697430000000
C	2.516974000000	1.657202000000	-0.859230000000
C	1.478806000000	1.953279000000	-3.000794000000
C	2.470875000000	2.307527000000	-2.088370000000
H	1.678412000000	0.171531000000	0.412059000000
H	-0.157151000000	0.657956000000	-3.448615000000
H	3.264811000000	1.928624000000	-0.120088000000
H	1.423756000000	2.447086000000	-3.966769000000
H	3.196754000000	3.077016000000	-2.334287000000
C	-0.300831000000	-0.881126000000	-1.190932000000
C	-1.426282000000	-1.100260000000	-1.940323000000

H	-1.937603000000	-2.054743000000	-1.898289000000
C	0.144081000000	-1.987844000000	-0.245640000000
H	0.548949000000	-1.586149000000	0.688041000000
H	-0.748591000000	-2.558533000000	0.029481000000
C	1.159486000000	-3.034726000000	-0.804109000000
C	2.572158000000	-2.435247000000	-0.905860000000
H	-1.748284000000	-0.444292000000	-2.742111000000
C	3.350566000000	-2.491793000000	-2.176192000000
C	4.792522000000	-2.631178000000	-4.559751000000
C	3.990934000000	-3.677630000000	-2.540798000000
C	3.438440000000	-1.373709000000	-3.005230000000
C	4.156365000000	-1.448043000000	-4.194439000000
C	4.711638000000	-3.745816000000	-3.728772000000
H	3.929713000000	-4.545789000000	-1.890028000000
H	2.956659000000	-0.446853000000	-2.716476000000
H	4.221276000000	-0.572275000000	-4.832967000000
H	5.210411000000	-4.670210000000	-4.004207000000
H	5.353268000000	-2.684487000000	-5.488200000000
C	1.216921000000	-4.198433000000	0.206467000000
H	0.235237000000	-4.678825000000	0.280983000000
H	1.504844000000	-3.836067000000	1.197650000000
H	1.946018000000	-4.954886000000	-0.107020000000
C	0.684925000000	-3.572880000000	-2.158675000000
H	0.682075000000	-2.799266000000	-2.931425000000
H	-0.337096000000	-3.953020000000	-2.056006000000
H	1.317544000000	-4.395421000000	-2.505946000000
N	3.013641000000	-1.923547000000	0.178053000000
O	4.321872000000	-1.414362000000	-0.008418000000
C	4.641690000000	-0.459680000000	0.879479000000
O	3.956962000000	-0.035247000000	1.769838000000
C	6.028224000000	0.035525000000	0.584325000000
C	8.591563000000	1.045228000000	0.106138000000
C	6.387249000000	0.504624000000	-0.676978000000
C	6.976310000000	0.090011000000	1.603103000000
C	8.254139000000	0.581934000000	1.372377000000
C	7.657228000000	1.010972000000	-0.921530000000
F	5.508260000000	0.514647000000	-1.672550000000
F	7.979577000000	1.469567000000	-2.126975000000
F	9.807245000000	1.523779000000	-0.120720000000
F	9.154167000000	0.609935000000	2.349958000000
F	6.683726000000	-0.360021000000	2.817457000000
C	-5.247965000000	2.232391000000	0.977480000000
H	-5.996458000000	2.341983000000	1.772475000000
H	-5.453183000000	2.990679000000	0.216246000000
H	-5.378061000000	1.246838000000	0.524920000000
C	-3.847693000000	2.414834000000	1.575158000000

H	-3.783126000000	3.456587000000	1.918636000000
C	-3.643227000000	1.534668000000	2.830943000000
P	-2.503101000000	2.235266000000	0.278274000000
H	-2.665237000000	1.733618000000	3.286869000000
H	-4.383074000000	1.891881000000	3.561276000000
C	-3.831655000000	0.002886000000	2.731188000000
H	-4.631105000000	-0.220157000000	2.015673000000
C	-4.219247000000	-0.554585000000	4.103838000000
P	-2.324042000000	-0.798976000000	1.965186000000
H	-4.276963000000	-1.647363000000	4.098972000000
H	-3.497585000000	-0.250425000000	4.870231000000
H	-5.200969000000	-0.166210000000	4.400468000000
Cu	-2.386946000000	-0.072239000000	-0.260963000000
B	-4.234118000000	-0.629203000000	-1.002663000000
H	-6.535147000000	-3.255905000000	0.000874000000
C	-6.378619000000	-2.945660000000	-1.037651000000
H	-7.160716000000	-3.404668000000	-1.651662000000
H	-5.404524000000	-3.325694000000	-1.361401000000
C	-6.420792000000	-1.416306000000	-1.110610000000
C	-5.985587000000	-0.853790000000	-2.501162000000
C	-7.765509000000	-0.909933000000	-0.603643000000
O	-5.383661000000	-0.920829000000	-0.248348000000
C	-6.298412000000	-1.760110000000	-3.685276000000
C	-6.518013000000	0.557088000000	-2.770419000000
O	-4.560945000000	-0.745306000000	-2.355992000000
H	-5.797018000000	-2.726210000000	-3.591957000000
H	-7.377988000000	-1.930478000000	-3.772359000000
H	-5.952569000000	-1.289043000000	-4.610771000000
H	-6.024842000000	0.954338000000	-3.663023000000
H	-7.599538000000	0.560129000000	-2.943363000000
H	-6.290068000000	1.231470000000	-1.938719000000
H	-7.770006000000	0.177902000000	-0.502499000000
H	-8.574864000000	-1.202875000000	-1.282557000000
H	-7.974306000000	-1.341170000000	0.380756000000
C	-0.957505000000	-0.403067000000	3.118929000000
C	0.010811000000	0.486604000000	2.644665000000
C	-0.833637000000	-0.900684000000	4.423552000000
C	1.080769000000	0.878349000000	3.444398000000
C	0.234979000000	-0.512020000000	5.224543000000
C	1.192839000000	0.376810000000	4.736272000000
H	-0.077365000000	0.872638000000	1.633979000000
H	-1.562015000000	-1.609630000000	4.806491000000
H	1.827070000000	1.557931000000	3.046806000000
H	0.325197000000	-0.908792000000	6.231767000000
H	2.031392000000	0.668166000000	5.361796000000
C	-2.600572000000	-2.599640000000	2.144753000000

C	-3.809461000000	-3.134566000000	1.677841000000
C	-1.596243000000	-3.475165000000	2.569806000000
C	-4.013950000000	-4.509788000000	1.666046000000
C	-1.802563000000	-4.853092000000	2.549753000000
C	-3.012535000000	-5.374928000000	2.103248000000
H	-4.579742000000	-2.471884000000	1.293240000000
H	-0.641049000000	-3.085095000000	2.908109000000
H	-4.957150000000	-4.907453000000	1.301447000000
H	-1.009953000000	-5.517453000000	2.882415000000
H	-3.172553000000	-6.449092000000	2.088618000000
C	-3.129578000000	3.296506000000	-1.084430000000
C	-3.573350000000	2.686385000000	-2.260769000000
C	-3.203032000000	4.689535000000	-0.967929000000
C	-4.087637000000	3.457970000000	-3.301109000000
C	-3.717432000000	5.457286000000	-2.006714000000
C	-4.161163000000	4.841559000000	-3.176363000000
H	-3.532607000000	1.603948000000	-2.352382000000
H	-2.845519000000	5.175817000000	-0.063959000000
H	-4.430713000000	2.972579000000	-4.210355000000
H	-3.769507000000	6.537614000000	-1.906081000000
H	-4.560717000000	5.442192000000	-3.988552000000
C	-1.148321000000	3.298886000000	0.932878000000
C	-0.074549000000	3.554563000000	0.071210000000
C	-1.097585000000	3.822169000000	2.229059000000
C	1.007934000000	4.319375000000	0.489182000000
C	-0.004983000000	4.574872000000	2.653551000000
C	1.050593000000	4.828549000000	1.784655000000
H	-0.083541000000	3.153393000000	-0.939110000000
H	-1.905773000000	3.650795000000	2.930762000000
H	1.825401000000	4.504113000000	-0.200816000000
H	0.015404000000	4.963774000000	3.667458000000
H	1.901419000000	5.417856000000	2.114014000000

II-*si*-TS

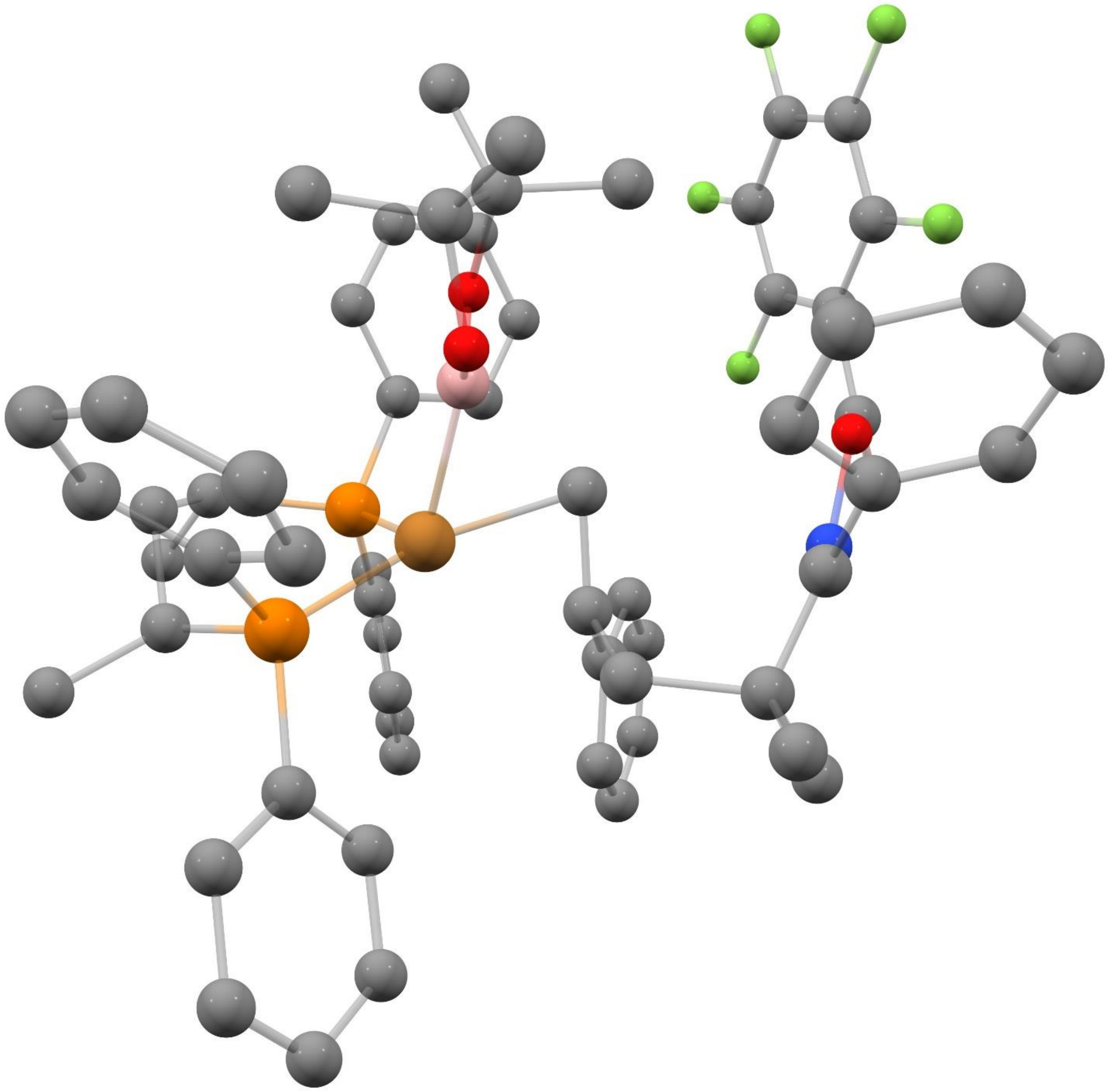

E$_{SCF}$ (wB97X-D/6-31g*-LANL2DZ): -4119.36243071 Hartree
E$_{SCF}$ (wB97X-D/6-311+g-SDD/SMD(THF)):** -4122.77237761 Hartree
Entropy (wB97X-D/6-31g*-LANL2DZ): 380.345 cal mol^{-1} K^{-1}
Thermal Correction to the Energy (wB97X-D/6-31g*-LANL2DZ): 1.183445 Hartree particle^{-1}
Thermal Correction to the Enthalpy (wB97X-D/6-31g*-LANL2DZ): 1.184389 Hartree particle^{-1}
Thermal Correction to the Gibbs Free Energy (wB97X-D/6-31g*-LANL2DZ): 1.003675 Hartree particle^{-1}
Nuclear Repulsion Energy: 15562.9533135448 Hartree
Imaginary Frequency: -171.31 cm^{-1}

P	1.737123000000	2.305321000000	-1.059111000000
P	3.470495000000	-0.863926000000	-0.682578000000
Cu	1.465746000000	0.086968000000	-0.258162000000
B	-0.018050000000	-0.756022000000	-1.418058000000
O	-0.065534000000	-2.111628000000	-1.690232000000
O	-0.914503000000	-0.039801000000	-2.194222000000
C	-0.808765000000	-2.256212000000	-2.917640000000
C	-1.757224000000	-1.012358000000	-2.856410000000
C	0.192079000000	-2.183746000000	-4.069967000000
C	-1.512729000000	-3.602051000000	-2.901522000000
C	-2.206900000000	-0.480262000000	-4.202817000000
C	-2.935377000000	-1.238129000000	-1.918288000000
H	0.688682000000	-1.203772000000	-4.093961000000
H	0.961122000000	-2.945219000000	-3.921571000000
H	-0.297997000000	-2.358181000000	-5.036837000000
H	-2.169538000000	-3.707993000000	-3.781073000000
H	-0.767318000000	-4.413309000000	-2.942683000000
H	-2.113307000000	-3.722873000000	-2.005013000000
H	-2.887651000000	0.366179000000	-4.052757000000
H	-1.354017000000	-0.126763000000	-4.790133000000
H	-2.725269000000	-1.253651000000	-4.776224000000
H	-3.631882000000	-2.016048000000	-2.270013000000
H	-2.524403000000	-1.541794000000	-0.945422000000
H	-3.504188000000	-0.309364000000	-1.774989000000
C	-0.337609000000	-0.285459000000	0.516796000000
H	-0.978681000000	0.570708000000	0.301257000000
C	0.549082000000	-0.189207000000	1.659691000000
H	-0.907398000000	-1.215856000000	0.448279000000
C	0.626875000000	1.033100000000	2.482082000000
C	1.577665000000	1.184798000000	3.506245000000
C	-0.280525000000	2.095699000000	2.320706000000
C	1.632977000000	2.309702000000	4.309699000000
C	-0.227946000000	3.234964000000	3.118853000000
C	0.738592000000	3.364428000000	4.120860000000
H	2.291907000000	0.375903000000	3.689012000000
H	-1.074088000000	2.004922000000	1.591393000000
H	2.384940000000	2.373831000000	5.091146000000
H	-0.968505000000	4.016919000000	2.985674000000
H	0.766534000000	4.249249000000	4.744354000000
C	0.894737000000	-1.538157000000	2.288203000000
H	1.867810000000	-1.534675000000	2.784560000000
H	0.985358000000	-2.262408000000	1.468136000000
C	-0.109944000000	-2.158499000000	3.348408000000
C	-0.037378000000	-1.420910000000	4.693576000000
H	1.009471000000	-1.385246000000	5.047168000000

H	-0.630486000000	-1.945863000000	5.443992000000
H	-0.404106000000	-0.398545000000	4.615219000000
C	0.314564000000	-3.617142000000	3.580828000000
H	1.315676000000	-3.637812000000	4.025955000000
H	0.336676000000	-4.202373000000	2.657050000000
H	-0.373390000000	-4.128322000000	4.281142000000
C	-1.531032000000	-2.073272000000	2.805571000000
N	-2.065980000000	-0.911542000000	2.922774000000
O	-3.218169000000	-0.804950000000	2.122525000000
C	-3.820526000000	0.397229000000	2.269411000000
O	-3.692081000000	1.143846000000	3.204053000000
C	-4.639541000000	0.700153000000	1.059037000000
C	-6.088746000000	1.353943000000	-1.250301000000
C	-4.614567000000	1.992746000000	0.531478000000
C	-5.415298000000	-0.253476000000	0.403962000000
C	-6.146345000000	0.063961000000	-0.738286000000
C	-5.325029000000	2.322001000000	-0.612705000000
C	-2.220533000000	-3.232158000000	2.159817000000
C	-3.537650000000	-5.458232000000	1.103026000000
C	-3.372409000000	-3.734073000000	2.780948000000
C	-1.747847000000	-3.843471000000	1.000850000000
C	-2.406231000000	-4.947324000000	0.476052000000
C	-4.021960000000	-4.845316000000	2.256117000000
H	-3.750245000000	-3.252093000000	3.676912000000
H	-0.884732000000	-3.425605000000	0.502526000000
H	-2.019999000000	-5.421691000000	-0.420350000000
H	-4.918608000000	-5.231357000000	2.747431000000
H	-4.038896000000	-6.331544000000	0.696444000000
F	-5.493727000000	-1.507409000000	0.857485000000
F	-3.871069000000	2.941903000000	1.101586000000
F	-5.279665000000	3.558345000000	-1.106989000000
F	-6.766781000000	1.662324000000	-2.346298000000
F	-6.879978000000	-0.862029000000	-1.347611000000
C	0.196356000000	2.935408000000	-1.814349000000
C	-2.332056000000	3.611877000000	-2.863451000000
C	-0.780276000000	3.533421000000	-1.008085000000
C	-0.125924000000	2.670977000000	-3.158633000000
C	-1.367218000000	3.022261000000	-3.673420000000
C	-2.027293000000	3.863032000000	-1.519323000000
H	-0.567759000000	3.747005000000	0.036110000000
H	0.578156000000	2.150506000000	-3.799863000000
H	-1.586077000000	2.802083000000	-4.717848000000
H	-2.770189000000	4.319960000000	-0.880427000000
H	-3.307268000000	3.861724000000	-3.259991000000
C	2.233974000000	3.627074000000	0.114945000000
C	3.195902000000	5.534190000000	1.914259000000

C	2.963139000000	3.231258000000	1.236489000000
C	1.976151000000	4.982726000000	-0.093476000000
C	2.446935000000	5.929674000000	0.813427000000
C	3.451935000000	4.179331000000	2.130722000000
H	3.129596000000	2.180451000000	1.417155000000
H	1.390918000000	5.301676000000	-0.948268000000
H	2.243634000000	6.986880000000	0.643861000000
H	3.998253000000	3.865225000000	3.011368000000
H	3.562994000000	6.278029000000	2.615973000000
C	4.528768000000	-1.366137000000	0.719298000000
C	6.122303000000	-1.954815000000	2.938313000000
C	5.508017000000	-2.368820000000	0.642546000000
C	4.357311000000	-0.673159000000	1.917144000000
C	5.148795000000	-0.964594000000	3.020936000000
C	6.293366000000	-2.657512000000	1.750110000000
H	5.646528000000	-2.915978000000	-0.288312000000
H	3.566707000000	0.076253000000	1.988598000000
H	4.999218000000	-0.416131000000	3.954688000000
H	7.051996000000	-3.440246000000	1.677702000000
H	6.743808000000	-2.187084000000	3.800427000000
C	3.436570000000	-2.342854000000	-1.771035000000
C	3.161085000000	-4.655700000000	-3.320548000000
C	2.805344000000	-3.475348000000	-1.250458000000
C	3.903350000000	-2.380425000000	-3.083620000000
C	3.767346000000	-3.530058000000	-3.857564000000
C	2.666332000000	-4.623192000000	-2.017500000000
H	2.394206000000	-3.452098000000	-0.244655000000
H	4.368654000000	-1.504406000000	-3.531148000000
H	4.144434000000	-3.540894000000	-4.875978000000
H	2.174322000000	-5.499059000000	-1.593888000000
H	3.053221000000	-5.559070000000	-3.925867000000
C	3.036294000000	2.422885000000	-2.446544000000
H	2.471814000000	2.702736000000	-3.339920000000
C	4.469089000000	0.459975000000	-1.515485000000
H	4.491033000000	1.226124000000	-0.717911000000
C	3.666708000000	1.043969000000	-2.704910000000
H	2.866154000000	0.351096000000	-3.016068000000
H	4.332729000000	1.142505000000	-3.573183000000
C	5.927675000000	0.144274000000	-1.837853000000
H	6.433121000000	1.046908000000	-2.204546000000
H	6.032650000000	-0.622831000000	-2.606260000000
H	6.469642000000	-0.203738000000	-0.942963000000
C	4.102723000000	3.507443000000	-2.224381000000
H	3.660903000000	4.505873000000	-2.173106000000
H	4.803125000000	3.492801000000	-3.074661000000
H	4.671537000000	3.355689000000	-1.306318000000

II-*re*-TS

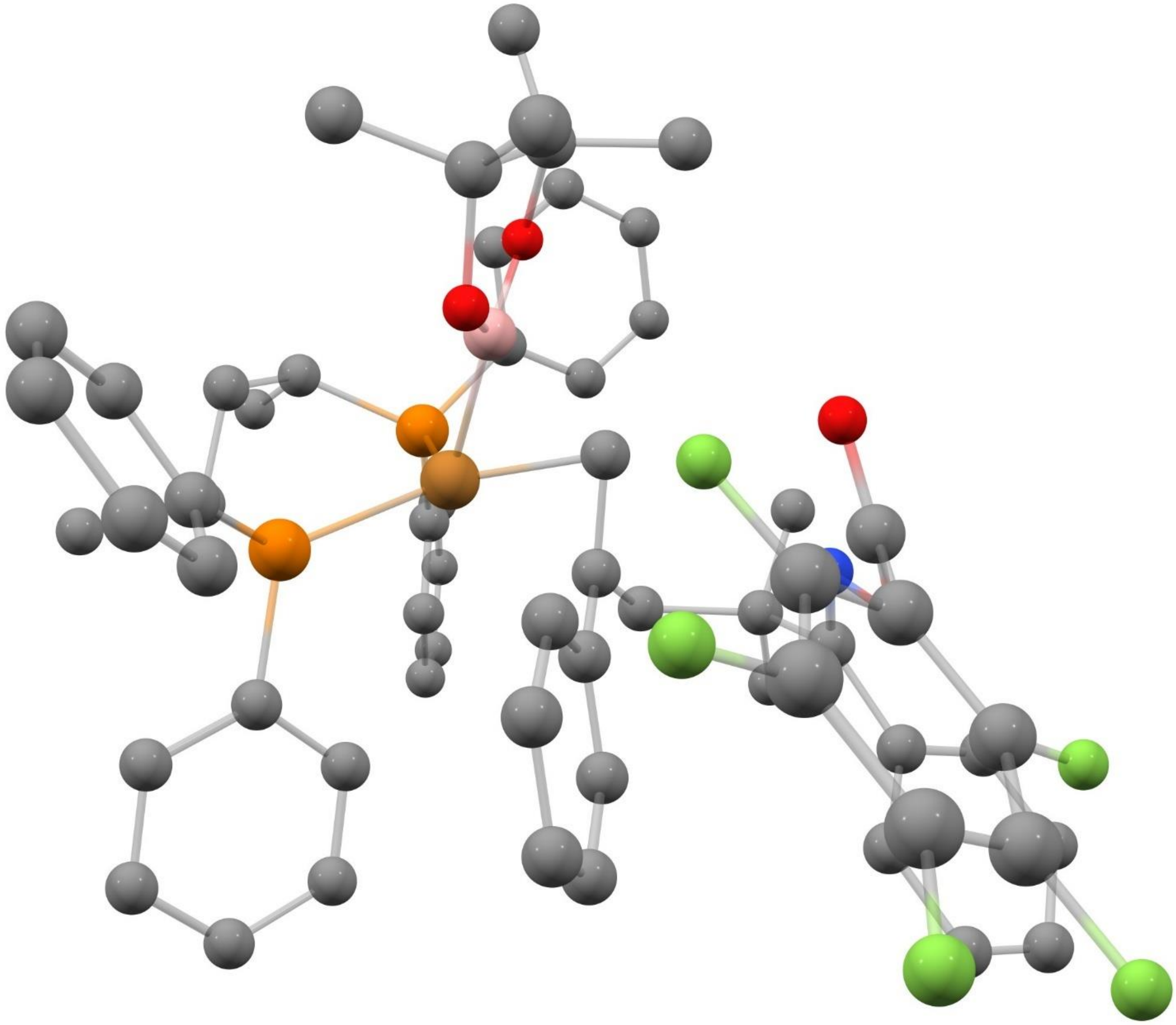

E$_{SCF}$ (wB97X-D/6-31g*-LANL2DZ): -4119.36082816 Hartree
E$_{SCF}$ (wB97X-D/6-311+g-SDD/SMD(THF)):** -4122.77817231 Hartree
Entropy (wB97X-D/6-31g*-LANL2DZ): 382.702 cal mol^{-1} K^{-1}
Thermal Correction to the Energy (wB97X-D/6-31g*-LANL2DZ): 1.183263 Hartree particle^{-1}
Thermal Correction to the Enthalpy (wB97X-D/6-31g*-LANL2DZ): 1.184207 Hartree particle^{-1}
Thermal Correction to the Gibbs Free Energy (wB97X-D/6-31g*-LANL2DZ): 1.002373 Hartree particle^{-1}
Nuclear Repulsion Energy: 15671.7156516519 Hartree
Imaginary Frequency: -167.39 cm^{-1}

P 3.811035000000 0.882589000000 -0.260575000000

P	2.091774000000	-1.679403000000	1.644361000000
Cu	1.707782000000	-0.176021000000	-0.003292000000
B	1.308044000000	-1.123788000000	-1.782971000000
O	0.550865000000	-2.279285000000	-1.855039000000
O	1.927354000000	-0.845554000000	-2.988990000000
C	0.786072000000	-2.899113000000	-3.136794000000
C	1.341606000000	-1.707734000000	-3.987426000000
C	1.814657000000	-4.016452000000	-2.910161000000
C	-0.527381000000	-3.480259000000	-3.629523000000
C	2.423047000000	-2.079753000000	-4.996597000000
C	0.244350000000	-0.895095000000	-4.678842000000
H	2.763311000000	-3.601759000000	-2.532249000000
H	1.425712000000	-4.705989000000	-2.156673000000
H	2.015185000000	-4.565968000000	-3.836715000000
H	-0.417491000000	-3.884200000000	-4.649358000000
H	-0.836825000000	-4.296484000000	-2.966858000000
H	-1.313991000000	-2.731905000000	-3.629480000000
H	2.763249000000	-1.176101000000	-5.510761000000
H	3.288206000000	-2.534885000000	-4.507875000000
H	2.035180000000	-2.777145000000	-5.743615000000
H	-0.208657000000	-1.450563000000	-5.504840000000
H	-0.542922000000	-0.603135000000	-3.981285000000
H	0.692326000000	0.022550000000	-5.085048000000
C	0.280144000000	0.518405000000	-1.211501000000
H	0.764732000000	1.221269000000	-1.887240000000
C	-0.041329000000	1.068919000000	0.101583000000
H	-0.529802000000	-0.007011000000	-1.727402000000
C	-1.061729000000	0.381688000000	0.901884000000
C	-1.477243000000	-0.933297000000	0.590517000000
C	-1.677201000000	0.978113000000	2.021837000000
C	-2.431261000000	-1.598278000000	1.347600000000
C	-2.631058000000	0.309712000000	2.784783000000
C	-3.017632000000	-0.990041000000	2.456371000000
H	-1.025025000000	-1.467555000000	-0.247755000000
H	-1.427167000000	1.999815000000	2.304799000000
H	-2.707833000000	-2.605849000000	1.065391000000
H	-3.069647000000	0.811614000000	3.643068000000
H	-3.768175000000	-1.511880000000	3.052858000000
C	0.228312000000	2.542978000000	0.320333000000
H	1.243575000000	2.781879000000	-0.017328000000
H	0.227359000000	2.772780000000	1.389119000000
C	-0.700621000000	3.634960000000	-0.391199000000
C	-0.149857000000	3.941281000000	-1.793958000000
H	0.890058000000	4.301257000000	-1.723407000000
H	-0.745790000000	4.724036000000	-2.281200000000
H	-0.167628000000	3.057479000000	-2.439160000000

C	-0.587297000000	4.900070000000	0.461927000000
H	0.465057000000	5.113963000000	0.642781000000
H	-1.081437000000	4.774845000000	1.438512000000
H	-1.032868000000	5.780306000000	-0.018820000000
C	-2.145128000000	3.136372000000	-0.526243000000
N	-2.242719000000	2.115380000000	-1.298080000000
O	-3.526823000000	1.543146000000	-1.222049000000
C	-3.576999000000	0.348206000000	-1.846433000000
O	-2.824938000000	-0.049399000000	-2.699231000000
C	-4.688982000000	-0.468645000000	-1.269640000000
C	-6.589444000000	-2.062451000000	0.046077000000
C	-4.503660000000	-1.853263000000	-1.128217000000
C	-5.861503000000	0.083300000000	-0.749374000000
C	-6.809357000000	-0.701256000000	-0.104532000000
C	-5.433438000000	-2.643411000000	-0.464140000000
C	-3.299319000000	3.790885000000	0.143051000000
C	-5.489788000000	5.099557000000	1.294154000000
C	-3.649567000000	5.086449000000	-0.262423000000
C	-4.074350000000	3.157167000000	1.113469000000
C	-5.156402000000	3.812891000000	1.690144000000
C	-4.737748000000	5.737053000000	0.308539000000
H	-3.077400000000	5.576788000000	-1.041595000000
H	-3.821886000000	2.138275000000	1.411612000000
H	-5.748455000000	3.305953000000	2.443652000000
H	-5.008651000000	6.733572000000	-0.026064000000
H	-6.347609000000	5.604385000000	1.736182000000
F	-6.123138000000	1.377043000000	-0.863227000000
F	-3.406840000000	-2.450594000000	-1.578723000000
F	-5.206430000000	-3.942346000000	-0.306889000000
F	-7.484952000000	-2.814618000000	0.674595000000
F	-7.916672000000	-0.157084000000	0.380895000000
C	4.037301000000	1.690242000000	-1.891283000000
C	4.017083000000	2.883750000000	-4.436173000000
C	3.601374000000	3.017447000000	-2.061081000000
C	4.444800000000	0.961303000000	-3.020120000000
C	4.444901000000	1.560333000000	-4.279715000000
C	3.593401000000	3.604384000000	-3.322638000000
H	3.263343000000	3.590516000000	-1.198282000000
H	4.729744000000	-0.087252000000	-2.936890000000
H	4.759922000000	0.978237000000	-5.139616000000
H	3.255560000000	4.638040000000	-3.429852000000
H	4.015433000000	3.346452000000	-5.414036000000
C	4.207223000000	2.213111000000	0.934661000000
C	4.713669000000	4.147872000000	2.899740000000
C	3.428700000000	2.271297000000	2.092656000000
C	5.232566000000	3.147271000000	0.760255000000

C	5.477530000000	4.113262000000	1.739484000000
C	3.677923000000	3.221770000000	3.080140000000
H	2.616764000000	1.567507000000	2.212228000000
H	5.833203000000	3.136268000000	-0.150913000000
H	6.278601000000	4.836537000000	1.590378000000
H	3.070114000000	3.248860000000	3.969703000000
H	4.902060000000	4.899626000000	3.659444000000
C	1.470557000000	-1.368392000000	3.339798000000
C	0.558731000000	-0.755824000000	5.912204000000
C	1.514790000000	-2.336518000000	4.359853000000
C	0.942637000000	-0.109659000000	3.628077000000
C	0.490535000000	0.193313000000	4.909060000000
C	1.066796000000	-2.024152000000	5.639138000000
H	1.893944000000	-3.330306000000	4.140712000000
H	0.844298000000	0.613506000000	2.825612000000
H	0.063004000000	1.174794000000	5.109132000000
H	1.110978000000	-2.778715000000	6.418261000000
H	0.196305000000	-0.527728000000	6.910782000000
C	1.614801000000	-3.419151000000	1.352791000000
C	0.698233000000	-6.005460000000	0.730354000000
C	0.301379000000	-3.802644000000	1.652111000000
C	2.454745000000	-4.350352000000	0.728217000000
C	2.005493000000	-5.636055000000	0.430895000000
C	-0.157770000000	-5.081784000000	1.336749000000
H	-0.373116000000	-3.091171000000	2.119121000000
H	3.482492000000	-4.087398000000	0.466789000000
H	2.678205000000	-6.346419000000	-0.051509000000
H	-1.180178000000	-5.356452000000	1.582568000000
H	0.336430000000	-6.997086000000	0.485895000000
C	5.206815000000	-0.377656000000	-0.093675000000
H	5.551768000000	-0.572761000000	-1.116870000000
C	3.953859000000	-1.648930000000	1.839008000000
H	4.107967000000	-0.635038000000	2.247906000000
C	4.625837000000	-1.709900000000	0.431663000000
H	3.926571000000	-2.083007000000	-0.321202000000
H	5.454156000000	-2.440133000000	0.469105000000
C	4.564426000000	-2.629883000000	2.840405000000
H	5.655822000000	-2.468270000000	2.887615000000
H	4.391497000000	-3.669098000000	2.553061000000
H	4.157794000000	-2.470078000000	3.843016000000
C	6.415394000000	0.085946000000	0.730216000000
H	6.871785000000	0.973669000000	0.293064000000
H	7.167846000000	-0.711280000000	0.755814000000
H	6.142865000000	0.329657000000	1.760063000000

III-(*S*)

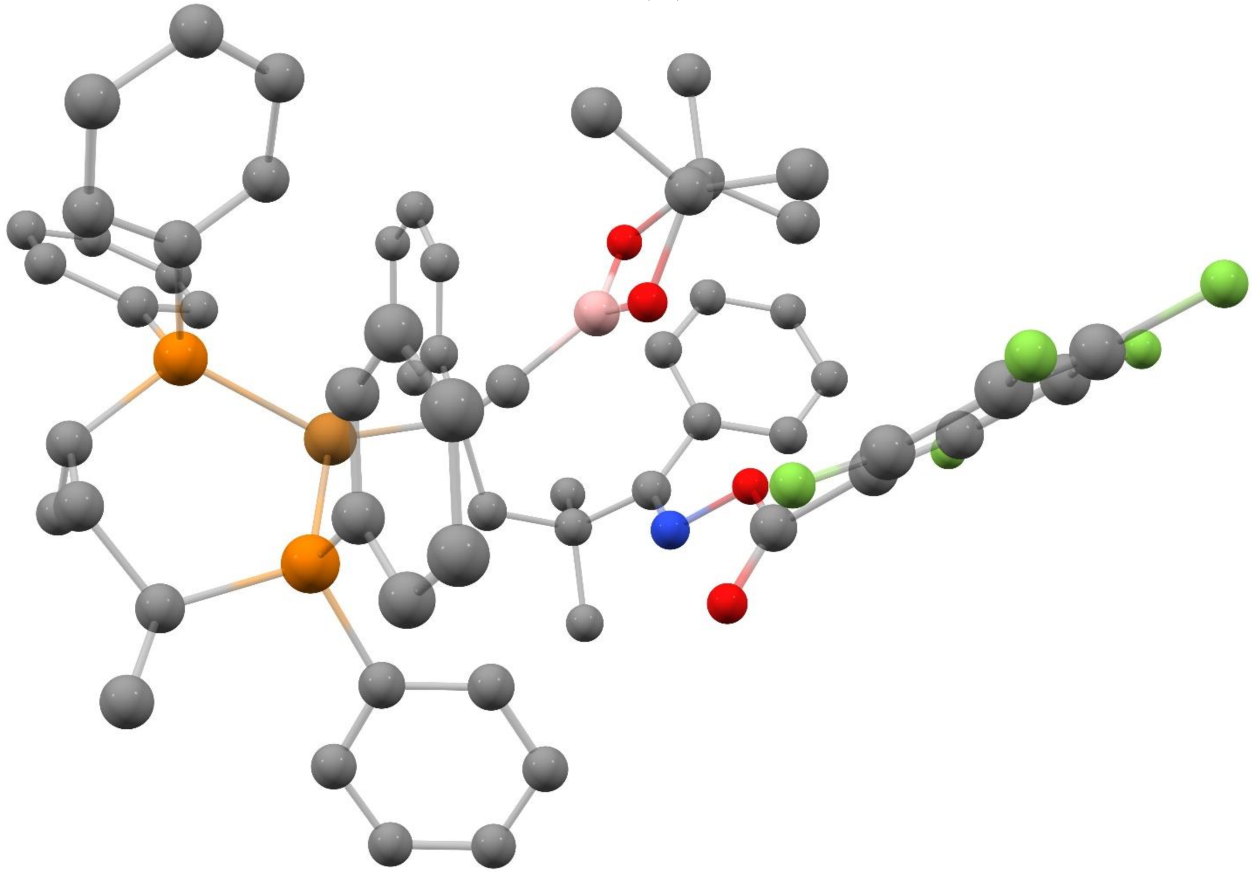

E$_{SCF}$ (wB97X-D/6-31g*-LANL2DZ): -4119.41656560 Hartree
E$_{SCF}$ (wB97X-D/6-311+g-SDD/SMD(THF)):** -4122.81690949 Hartree
Entropy (wB97X-D/6-31g*-LANL2DZ): 381.200 cal mol^{-1} K^{-1}
Thermal Correction to the Energy (wB97X-D/6-31g*-LANL2DZ): 1.186223 Hartree particle^{-1}
Thermal Correction to the Enthalpy (wB97X-D/6-31g*-LANL2DZ): 1.187167 Hartree particle^{-1}
Thermal Correction to the Gibbs Free Energy (wB97X-D/6-31g*-LANL2DZ): 1.006047 Hartree particle^{-1}
Nuclear Repulsion Energy: 15360.8831877652 Hartree

C	1.541599000000	2.593375000000	0.519865000000
C	2.253519000000	3.493281000000	1.358019000000
C	1.504989000000	2.960908000000	-0.849576000000
C	2.878011000000	4.632274000000	0.869922000000
C	2.148324000000	4.093085000000	-1.336408000000
C	2.845967000000	4.949672000000	-0.488966000000
H	2.328532000000	3.282952000000	2.419793000000
H	0.931466000000	2.359358000000	-1.544793000000
H	3.402319000000	5.282731000000	1.567202000000

H	2.084442000000	4.315814000000	-2.399453000000
H	3.334603000000	5.841414000000	-0.869382000000
C	0.956124000000	1.343874000000	1.020887000000
C	0.031787000000	0.533753000000	0.068998000000
H	0.645927000000	0.095093000000	-0.751628000000
C	0.616369000000	1.224960000000	2.511411000000
H	0.424960000000	0.166936000000	2.715590000000
H	1.495833000000	1.478394000000	3.119907000000
C	-0.582525000000	1.984076000000	3.190572000000
C	-1.842785000000	1.790782000000	2.336393000000
H	-0.317399000000	-0.356734000000	0.605579000000
C	-2.543852000000	2.944746000000	1.711029000000
C	-3.847735000000	5.213820000000	0.729934000000
C	-3.888053000000	3.176846000000	2.018883000000
C	-1.862995000000	3.849469000000	0.891908000000
C	-2.514116000000	4.977413000000	0.405934000000
C	-4.536936000000	4.305422000000	1.530074000000
H	-4.422778000000	2.472300000000	2.647052000000
H	-0.831941000000	3.664679000000	0.612767000000
H	-1.971675000000	5.668905000000	-0.231584000000
H	-5.579642000000	4.476985000000	1.779951000000
H	-4.351666000000	6.100135000000	0.354955000000
C	-0.822867000000	1.318471000000	4.562397000000
H	0.088518000000	1.391726000000	5.167237000000
H	-1.077969000000	0.262708000000	4.453041000000
H	-1.632732000000	1.819965000000	5.106150000000
C	-0.273528000000	3.463682000000	3.463640000000
H	0.020624000000	4.021801000000	2.575893000000
H	0.560061000000	3.517576000000	4.173294000000
H	-1.132788000000	3.970476000000	3.918274000000
N	-2.211644000000	0.565509000000	2.272768000000
O	-3.306464000000	0.382132000000	1.392763000000
C	-3.603177000000	-0.920031000000	1.243445000000
O	-3.070449000000	-1.846459000000	1.793585000000
C	-4.726499000000	-1.096292000000	0.271751000000
C	-6.825864000000	-1.564153000000	-1.527713000000
C	-4.718252000000	-2.209955000000	-0.570998000000
C	-5.813068000000	-0.225430000000	0.189296000000
C	-6.856313000000	-0.448774000000	-0.700212000000
C	-5.754728000000	-2.444992000000	-1.463946000000
F	-3.697923000000	-3.052883000000	-0.585391000000
F	-5.707260000000	-3.490688000000	-2.284993000000
F	-7.816406000000	-1.783432000000	-2.381595000000
F	-7.888398000000	0.387939000000	-0.751544000000
F	-5.901604000000	0.840188000000	0.978737000000
C	6.046869000000	-1.390348000000	1.302193000000

H	6.563911000000	-2.262636000000	1.719344000000
H	6.786591000000	-0.601461000000	1.139909000000
H	5.336184000000	-1.025441000000	2.053250000000
C	5.344747000000	-1.765084000000	-0.009383000000
H	6.127330000000	-1.953895000000	-0.756234000000
C	4.528608000000	-3.079130000000	0.105152000000
P	4.353799000000	-0.300712000000	-0.592763000000
H	3.925134000000	-3.237243000000	-0.797840000000
H	5.269138000000	-3.891072000000	0.107686000000
C	3.638154000000	-3.309884000000	1.346042000000
H	4.181059000000	-2.952760000000	2.229058000000
C	3.362232000000	-4.807102000000	1.518202000000
P	2.085681000000	-2.269621000000	1.283880000000
H	2.696030000000	-5.000556000000	2.364477000000
H	2.893780000000	-5.222286000000	0.619622000000
H	4.299291000000	-5.348865000000	1.693461000000
Cu	2.491712000000	-0.009262000000	0.707083000000
B	-1.171937000000	0.947204000000	-0.862850000000
H	-1.172251000000	-0.990868000000	-3.703736000000
C	-1.332842000000	0.091120000000	-3.719145000000
H	-1.624165000000	0.390984000000	-4.731112000000
H	-0.381184000000	0.573348000000	-3.471450000000
C	-2.408979000000	0.440958000000	-2.685548000000
C	-2.506237000000	1.971372000000	-2.396623000000
C	-3.720969000000	-0.222152000000	-3.067881000000
O	-1.942084000000	-0.065963000000	-1.422625000000
C	-2.256366000000	2.873003000000	-3.597120000000
C	-3.809822000000	2.359888000000	-1.698806000000
O	-1.427027000000	2.155091000000	-1.460416000000
H	-1.261440000000	2.710803000000	-4.019101000000
H	-3.004970000000	2.695121000000	-4.377526000000
H	-2.325206000000	3.920198000000	-3.287765000000
H	-3.737946000000	3.392210000000	-1.350510000000
H	-4.663646000000	2.282503000000	-2.380567000000
H	-3.991075000000	1.730183000000	-0.823422000000
H	-4.493779000000	-0.037831000000	-2.321374000000
H	-4.075260000000	0.162489000000	-4.030754000000
H	-3.576452000000	-1.303218000000	-3.162252000000
C	1.048242000000	-3.127059000000	0.036421000000
C	1.099035000000	-2.670279000000	-1.284602000000
C	0.218068000000	-4.212715000000	0.340125000000
C	0.350099000000	-3.285696000000	-2.282678000000
C	-0.547676000000	-4.814148000000	-0.652781000000
C	-0.482377000000	-4.353944000000	-1.966295000000
H	1.719746000000	-1.819920000000	-1.543646000000
H	0.152092000000	-4.576229000000	1.360854000000

H	0.415571000000	-2.914563000000	-3.301201000000
H	-1.202381000000	-5.641882000000	-0.397782000000
H	-1.085611000000	-4.824292000000	-2.737247000000
C	1.238977000000	-2.579722000000	2.875065000000
C	1.936896000000	-2.879725000000	4.050019000000
C	-0.142034000000	-2.360148000000	2.950185000000
C	1.265618000000	-2.973703000000	5.266718000000
C	-0.811010000000	-2.458954000000	4.164277000000
C	-0.109456000000	-2.765637000000	5.327231000000
H	3.010553000000	-3.043902000000	4.031142000000
H	-0.720985000000	-2.105223000000	2.068615000000
H	1.822398000000	-3.210830000000	6.168696000000
H	-1.880709000000	-2.277764000000	4.181019000000
H	-0.629758000000	-2.837152000000	6.277803000000
C	5.585807000000	1.030637000000	-0.801225000000
C	5.278775000000	2.284363000000	-0.270906000000
C	6.781176000000	0.850879000000	-1.507758000000
C	6.162179000000	3.349041000000	-0.438457000000
C	7.666095000000	1.910596000000	-1.662067000000
C	7.355800000000	3.161749000000	-1.126189000000
H	4.342180000000	2.438185000000	0.258412000000
H	7.016317000000	-0.114345000000	-1.950396000000
H	5.899276000000	4.322184000000	-0.035991000000
H	8.593727000000	1.766890000000	-2.208464000000
H	8.045673000000	3.990762000000	-1.255657000000
C	3.881439000000	-0.674311000000	-2.325393000000
C	2.950657000000	0.196476000000	-2.908031000000
C	4.345111000000	-1.763091000000	-3.067051000000
C	2.487865000000	-0.025656000000	-4.199819000000
C	3.867197000000	-1.994182000000	-4.355588000000
C	2.935796000000	-1.130155000000	-4.922605000000
H	2.590382000000	1.052939000000	-2.341613000000
H	5.075128000000	-2.449035000000	-2.648271000000
H	1.772115000000	0.661521000000	-4.641069000000
H	4.226195000000	-2.852433000000	-4.915834000000
H	2.563760000000	-1.312572000000	-5.926373000000

III-(*R*)

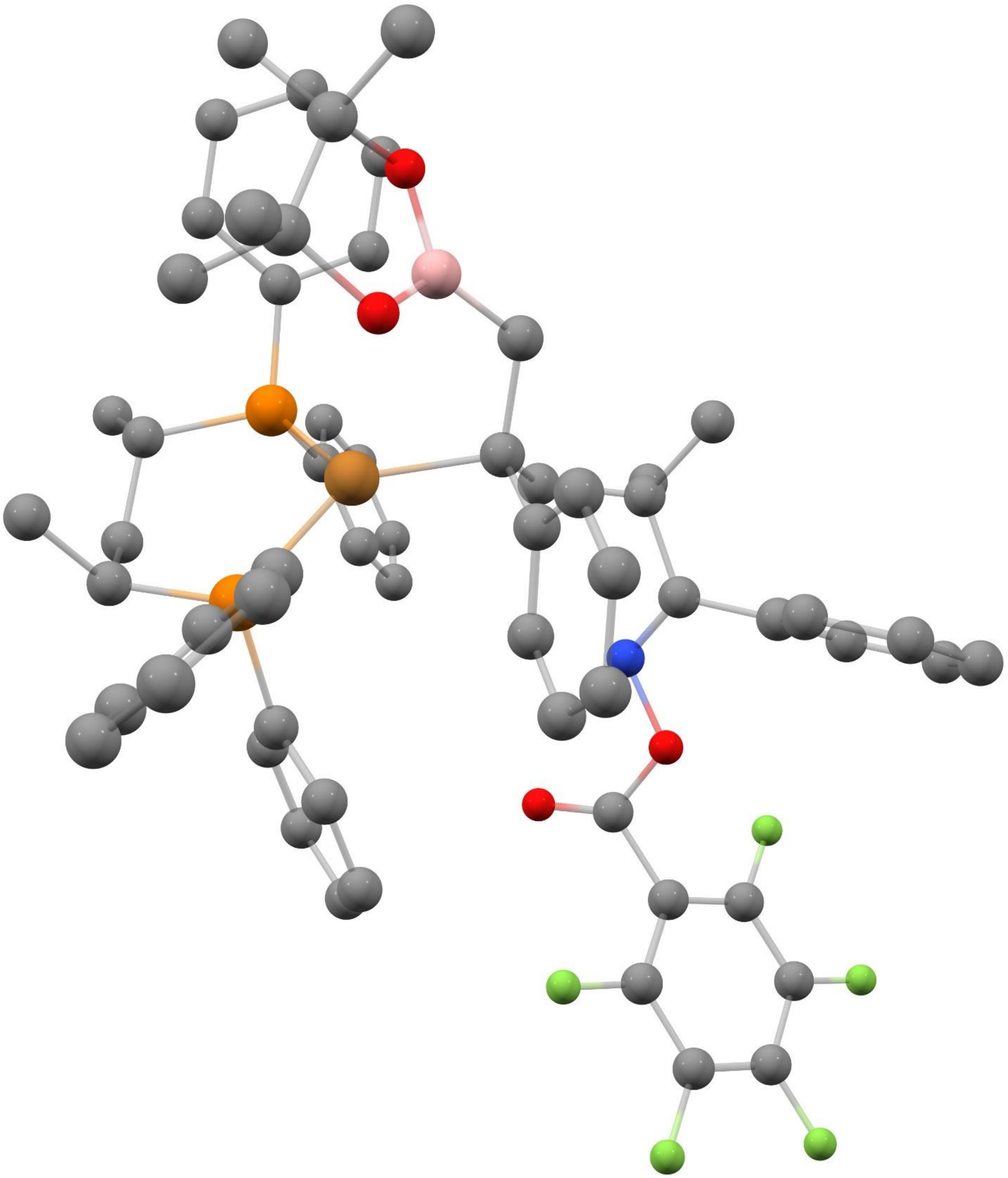

E$_{SCF}$ (wB97X-D/6-31g*-LANL2DZ): -4119.42248043 Hartree
E$_{SCF}$ (wB97X-D/6-311+g-SDD/SMD(THF)):** -4122.82687899 Hartree
Entropy (wB97X-D/6-31g*-LANL2DZ): 387.636 cal mol^{-1} K^{-1}

Thermal Correction to the Energy (wB97X-D/6-31g*-LANL2DZ): 1.185619 Hartree particle^{-1}
Thermal Correction to the Enthalpy (wB97X-D/6-31g*-LANL2DZ): 1.186563 Hartree particle^{-1}
Thermal Correction to the Gibbs Free Energy (wB97X-D/6-31g*-LANL2DZ): 1.002385 Hartree particle^{-1}
Nuclear Repulsion Energy: 14998.9611016913 Hartree

C	-0.122242000000	-0.334434000000	-1.884917000000
C	0.957607000000	0.432149000000	-1.383265000000
C	-0.353150000000	-0.223491000000	-3.271705000000
C	1.739683000000	1.236463000000	-2.196791000000
C	0.423759000000	0.598846000000	-4.090715000000
C	1.482194000000	1.334459000000	-3.567873000000
H	1.172605000000	0.409063000000	-0.318710000000
H	-1.170429000000	-0.769709000000	-3.729293000000
H	2.558866000000	1.798579000000	-1.753484000000
H	0.195880000000	0.654043000000	-5.153463000000
H	2.091002000000	1.969498000000	-4.205339000000
C	-0.966427000000	-1.159066000000	-0.974580000000
C	-2.044286000000	-2.011792000000	-1.690211000000
H	-1.660691000000	-2.548336000000	-2.579423000000
C	-0.160879000000	-1.994643000000	0.047634000000
H	0.332801000000	-1.355020000000	0.785178000000
H	-0.888058000000	-2.577977000000	0.628548000000
C	0.941646000000	-3.017785000000	-0.401043000000
C	2.311305000000	-2.337689000000	-0.555365000000
H	-2.363307000000	-2.817134000000	-1.015118000000
C	3.196284000000	-2.632401000000	-1.719414000000
C	4.874438000000	-3.243301000000	-3.867895000000
C	4.271589000000	-3.508850000000	-1.546296000000
C	2.963221000000	-2.062896000000	-2.972326000000
C	3.805404000000	-2.365832000000	-4.039431000000
C	5.105080000000	-3.816601000000	-2.618039000000
H	4.460343000000	-3.941083000000	-0.567241000000
H	2.132391000000	-1.379951000000	-3.113905000000
H	3.619984000000	-1.910649000000	-5.008240000000
H	5.937838000000	-4.499485000000	-2.474164000000
H	5.526748000000	-3.479378000000	-4.704152000000
C	1.114588000000	-4.039280000000	0.747325000000
H	0.207304000000	-4.648250000000	0.838925000000
H	1.288779000000	-3.526569000000	1.698687000000
H	1.957618000000	-4.716192000000	0.558132000000
C	0.561063000000	-3.784272000000	-1.671977000000
H	0.459320000000	-3.123903000000	-2.536678000000
H	-0.398745000000	-4.286336000000	-1.515459000000

H	1.308918000000	-4.547901000000	-1.914924000000
N	2.676006000000	-1.614855000000	0.437292000000
O	3.976377000000	-1.096275000000	0.209095000000
C	4.405280000000	-0.261466000000	1.160511000000
O	3.800356000000	0.157523000000	2.109418000000
C	5.841357000000	0.082775000000	0.851467000000
C	8.517334000000	0.719084000000	0.324438000000
C	6.227148000000	1.390302000000	0.581282000000
C	6.826232000000	-0.900308000000	0.849636000000
C	8.156756000000	-0.596047000000	0.593727000000
C	7.550561000000	1.717336000000	0.314963000000
F	5.318274000000	2.364491000000	0.541694000000
F	7.895991000000	2.974921000000	0.045983000000
F	9.786677000000	1.021895000000	0.075439000000
F	9.085860000000	-1.549354000000	0.608913000000
F	6.509771000000	-2.166674000000	1.121273000000
C	-4.111055000000	3.619759000000	1.303853000000
H	-4.796191000000	3.949373000000	2.095341000000
H	-3.956633000000	4.457347000000	0.619933000000
H	-4.611604000000	2.827339000000	0.740005000000
C	-2.796007000000	3.138512000000	1.929037000000
H	-2.284578000000	4.011112000000	2.356853000000
C	-3.080823000000	2.188487000000	3.121819000000
P	-1.617268000000	2.423659000000	0.646306000000
H	-2.150717000000	1.806096000000	3.556311000000
H	-3.519468000000	2.823532000000	3.904728000000
C	-4.051340000000	0.999655000000	2.936248000000
H	-4.886185000000	1.325360000000	2.303561000000
C	-4.601566000000	0.580317000000	4.304995000000
P	-3.238727000000	-0.392342000000	1.989920000000
H	-5.224353000000	-0.317614000000	4.247658000000
H	-3.782810000000	0.372456000000	5.002652000000
H	-5.210260000000	1.387552000000	4.730848000000
Cu	-1.918773000000	0.200899000000	0.214970000000
B	-3.357684000000	-1.272312000000	-2.111250000000
H	-5.969864000000	-2.530085000000	-4.190827000000
C	-5.648035000000	-1.484127000000	-4.199460000000
H	-6.381711000000	-0.899615000000	-4.764965000000
H	-4.685152000000	-1.431442000000	-4.718550000000
C	-5.528473000000	-0.991959000000	-2.755200000000
C	-4.818316000000	0.400562000000	-2.637795000000
C	-6.879072000000	-1.075648000000	-2.059057000000
O	-4.600760000000	-1.866468000000	-2.087028000000
C	-5.018531000000	1.329991000000	-3.827232000000
C	-5.145698000000	1.143984000000	-1.341951000000
O	-3.430409000000	0.029997000000	-2.561859000000

H	-4.628379000000	0.890903000000	-4.748644000000
H	-6.082802000000	1.553496000000	-3.967283000000
H	-4.491574000000	2.272978000000	-3.649540000000
H	-4.463804000000	1.995387000000	-1.260461000000
H	-6.175288000000	1.518348000000	-1.334451000000
H	-5.000146000000	0.498315000000	-0.468071000000
H	-6.787857000000	-0.879523000000	-0.988781000000
H	-7.583648000000	-0.357863000000	-2.496175000000
H	-7.293836000000	-2.081673000000	-2.178975000000
C	-2.120907000000	-1.153342000000	3.229919000000
C	-0.779323000000	-0.752903000000	3.221111000000
C	-2.533346000000	-2.109313000000	4.165356000000
C	0.135130000000	-1.287675000000	4.124808000000
C	-1.620676000000	-2.649117000000	5.068153000000
C	-0.286724000000	-2.239470000000	5.050218000000
H	-0.428183000000	-0.035728000000	2.483726000000
H	-3.566920000000	-2.444851000000	4.180231000000
H	1.174495000000	-0.974726000000	4.079341000000
H	-1.951149000000	-3.395866000000	5.784909000000
H	0.423589000000	-2.671169000000	5.749782000000
C	-4.557214000000	-1.634533000000	1.731883000000
C	-5.922436000000	-1.336094000000	1.780857000000
C	-4.165400000000	-2.917980000000	1.327638000000
C	-6.872350000000	-2.301945000000	1.448955000000
C	-5.111386000000	-3.880050000000	0.996827000000
C	-6.470677000000	-3.574820000000	1.056338000000
H	-6.264603000000	-0.348266000000	2.076201000000
H	-3.107845000000	-3.163072000000	1.267079000000
H	-7.928924000000	-2.051900000000	1.492874000000
H	-4.785982000000	-4.866026000000	0.678181000000
H	-7.211061000000	-4.323792000000	0.790575000000
C	-1.762853000000	3.521996000000	-0.809143000000
C	-1.849392000000	2.925400000000	-2.069025000000
C	-1.711473000000	4.919251000000	-0.711417000000
C	-1.890755000000	3.716507000000	-3.217942000000
C	-1.766477000000	5.706389000000	-1.855628000000
C	-1.855527000000	5.103160000000	-3.113373000000
H	-1.876111000000	1.843056000000	-2.163347000000
H	-1.609293000000	5.393234000000	0.262549000000
H	-1.938741000000	3.236337000000	-4.191162000000
H	-1.726780000000	6.788917000000	-1.771272000000
H	-1.886164000000	5.718969000000	-4.008154000000
C	0.047503000000	2.872408000000	1.291253000000
C	1.028924000000	3.340018000000	0.408183000000
C	0.405930000000	2.659209000000	2.628735000000
C	2.324380000000	3.588430000000	0.851417000000

C	1.703115000000	2.906274000000	3.070351000000
C	2.668747000000	3.369339000000	2.182378000000
H	0.788326000000	3.499301000000	-0.637707000000
H	-0.316539000000	2.288141000000	3.346675000000
H	3.070204000000	3.944802000000	0.146662000000
H	1.960251000000	2.718180000000	4.108756000000
H	3.685504000000	3.541778000000	2.520549000000

III-TS-inv

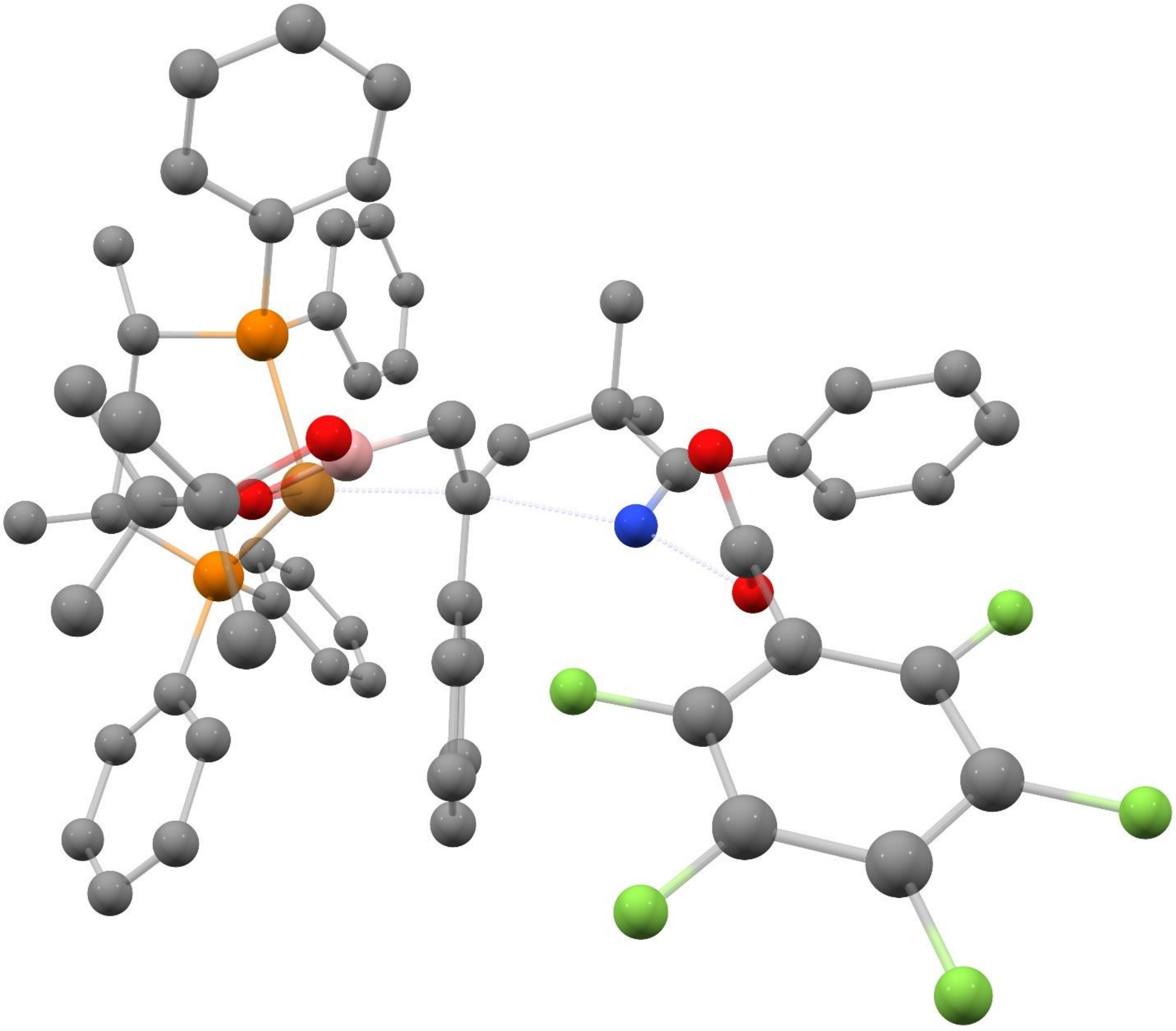

E$_{SCF}$ (wB97X-D/6-31g*-LANL2DZ): -4119.36902554 Hartree
E$_{SCF}$ (wB97X-D/6-311+g-SDD/SMD(THF)):** -4122.80010388 Hartree
Entropy (wB97X-D/6-31g*-LANL2DZ): 388.401 cal mol^{-1} K^{-1}
Thermal Correction to the Energy (wB97X-D/6-31g*-LANL2DZ): 1.183839 Hartree particle^{-1}

Thermal Correction to the Enthalpy (wB97X-D/6-31g*-LANL2DZ): 1.184783 Hartree particle^{-1}
Thermal Correction to the Gibbs Free Energy (wB97X-D/6-31g*-LANL2DZ): 1.000241 Hartree particle^{-1}
Nuclear Repulsion Energy: 15053.0531410985 Hartree
Imaginary Frequency: -512.10 cm^{-1}

C	0.451047000000	-0.361128000000	-1.217528000000
C	-0.046812000000	-0.375190000000	-2.542895000000
C	1.373436000000	-1.389081000000	-0.903554000000
C	0.320956000000	-1.354810000000	-3.454841000000
C	1.727841000000	-2.373965000000	-1.815986000000
C	1.199852000000	-2.379869000000	-3.104616000000
H	-0.739246000000	0.396829000000	-2.867467000000
H	1.900027000000	-1.358582000000	0.043780000000
H	-0.082412000000	-1.313544000000	-4.464993000000
H	2.465779000000	-3.116362000000	-1.520656000000
H	1.485968000000	-3.139194000000	-3.825507000000
C	0.154279000000	0.684456000000	-0.231696000000
C	0.633957000000	0.503538000000	1.213841000000
H	1.702889000000	0.744558000000	1.335981000000
C	-0.360962000000	2.043990000000	-0.659550000000
H	-0.804148000000	1.972305000000	-1.658229000000
H	-1.174203000000	2.394545000000	-0.010874000000
C	0.661264000000	3.216353000000	-0.694797000000
C	2.074289000000	2.654301000000	-0.974073000000
H	0.093723000000	1.212510000000	1.858755000000
C	3.172124000000	3.634605000000	-1.239731000000
C	5.193702000000	5.498447000000	-1.687963000000
C	3.324264000000	4.207619000000	-2.500413000000
C	4.038153000000	3.985286000000	-0.203213000000
C	5.043347000000	4.917898000000	-0.430062000000
C	4.338237000000	5.135970000000	-2.723549000000
H	2.664830000000	3.916004000000	-3.312047000000
H	3.945432000000	3.482286000000	0.754797000000
H	5.722245000000	5.179778000000	0.376003000000
H	4.460548000000	5.571045000000	-3.711139000000
H	5.984311000000	6.222244000000	-1.863115000000
C	0.217653000000	4.237984000000	-1.751209000000
H	-0.815017000000	4.537854000000	-1.534795000000
H	0.235451000000	3.802568000000	-2.756728000000
H	0.841872000000	5.136292000000	-1.749712000000
C	0.746977000000	3.929996000000	0.669685000000
H	1.024496000000	3.235432000000	1.467606000000
H	-0.228798000000	4.372941000000	0.904314000000
H	1.483200000000	4.739560000000	0.648029000000

N	2.248892000000	1.412231000000	-0.829456000000
O	4.143084000000	1.093882000000	-1.036545000000
C	4.528325000000	0.736896000000	0.126308000000
O	4.104711000000	1.075040000000	1.227573000000
C	5.678386000000	-0.269933000000	0.072190000000
C	7.781001000000	-2.120963000000	0.007675000000
C	5.460492000000	-1.613604000000	0.337253000000
C	6.973485000000	0.127641000000	-0.224964000000
C	8.025865000000	-0.779432000000	-0.256490000000
C	6.491293000000	-2.543059000000	0.303924000000
F	4.230071000000	-2.056869000000	0.624507000000
F	6.253827000000	-3.832558000000	0.554597000000
F	8.780932000000	-3.001161000000	-0.022169000000
F	9.265775000000	-0.375760000000	-0.536032000000
F	7.237896000000	1.410907000000	-0.477522000000
C	-4.857334000000	-2.835134000000	0.790410000000
H	-5.732213000000	-2.930071000000	1.444664000000
H	-4.735101000000	-3.773419000000	0.244056000000
H	-3.976071000000	-2.707422000000	1.426535000000
C	-5.037590000000	-1.646722000000	-0.159232000000
H	-5.830517000000	-1.904764000000	-0.874063000000
C	-5.544205000000	-0.396081000000	0.604047000000
P	-3.483983000000	-1.380610000000	-1.169937000000
H	-5.602250000000	0.474860000000	-0.058054000000
H	-6.584326000000	-0.623651000000	0.875041000000
C	-4.851357000000	0.049721000000	1.912272000000
H	-4.567727000000	-0.839384000000	2.488197000000
C	-5.842810000000	0.878007000000	2.738939000000
P	-3.241117000000	0.935636000000	1.555020000000
H	-5.385744000000	1.296780000000	3.639772000000
H	-6.239284000000	1.709059000000	2.145960000000
H	-6.688056000000	0.252267000000	3.048137000000
Cu	-1.861258000000	-0.320989000000	0.103133000000
B	0.394620000000	-0.910385000000	1.840679000000
H	2.707173000000	-3.506117000000	2.302802000000
C	1.666692000000	-3.716819000000	2.044203000000
H	1.477845000000	-4.786255000000	2.183597000000
H	1.530730000000	-3.469798000000	0.987328000000
C	0.756998000000	-2.868393000000	2.935105000000
C	-0.729065000000	-2.824151000000	2.433310000000
C	0.921958000000	-3.278754000000	4.392302000000
O	1.177269000000	-1.500794000000	2.789378000000
C	-1.184908000000	-4.080327000000	1.709438000000
C	-1.718188000000	-2.447103000000	3.534337000000
O	-0.693696000000	-1.713492000000	1.505650000000
H	-0.554632000000	-4.298958000000	0.846670000000

H	-1.156878000000	-4.937996000000	2.390870000000
H	-2.210476000000	-3.964329000000	1.348721000000
H	-2.690510000000	-2.228821000000	3.080257000000
H	-1.850672000000	-3.261353000000	4.253596000000
H	-1.388251000000	-1.551861000000	4.068930000000
H	0.414099000000	-2.584869000000	5.066060000000
H	0.526973000000	-4.287583000000	4.557084000000
H	1.985012000000	-3.281695000000	4.649243000000
C	-3.816820000000	2.485237000000	0.761749000000
C	-3.804568000000	2.540288000000	-0.636792000000
C	-4.298046000000	3.588618000000	1.476325000000
C	-4.279804000000	3.661385000000	-1.310458000000
C	-4.757472000000	4.716645000000	0.803936000000
C	-4.753996000000	4.753233000000	-0.589418000000
H	-3.403053000000	1.707421000000	-1.206913000000
H	-4.302533000000	3.570899000000	2.562222000000
H	-4.267561000000	3.677810000000	-2.396039000000
H	-5.121025000000	5.569247000000	1.369708000000
H	-5.114697000000	5.635277000000	-1.110144000000
C	-2.604737000000	1.463832000000	3.185409000000
C	-2.853083000000	0.744742000000	4.358479000000
C	-1.708411000000	2.539299000000	3.236071000000
C	-2.222557000000	1.092852000000	5.550852000000
C	-1.078552000000	2.885898000000	4.425177000000
C	-1.332518000000	2.161367000000	5.587518000000
H	-3.535456000000	-0.099808000000	4.353637000000
H	-1.494251000000	3.107032000000	2.335600000000
H	-2.428080000000	0.522599000000	6.451881000000
H	-0.381209000000	3.717815000000	4.439306000000
H	-0.835847000000	2.426646000000	6.515528000000
C	-3.039856000000	-3.042097000000	-1.781243000000
C	-1.719121000000	-3.463211000000	-1.626761000000
C	-3.960385000000	-3.873622000000	-2.432972000000
C	-1.322741000000	-4.710626000000	-2.106765000000
C	-3.566300000000	-5.121176000000	-2.897809000000
C	-2.244368000000	-5.540645000000	-2.733011000000
H	-0.991878000000	-2.814420000000	-1.145745000000
H	-4.983847000000	-3.539021000000	-2.585803000000
H	-0.286683000000	-5.015467000000	-1.997869000000
H	-4.283624000000	-5.764597000000	-3.398660000000
H	-1.935619000000	-6.512739000000	-3.106339000000
C	-4.071192000000	-0.568141000000	-2.711671000000
C	-3.234344000000	-0.647672000000	-3.833230000000
C	-5.257379000000	0.164328000000	-2.815505000000
C	-3.563935000000	0.005614000000	-5.014252000000
C	-5.586970000000	0.819931000000	-4.000692000000

C	-4.739372000000	0.749282000000	-5.100465000000
H	-2.320808000000	-1.230492000000	-3.778652000000
H	-5.946377000000	0.232610000000	-1.981845000000
H	-2.900410000000	-0.069599000000	-5.870582000000
H	-6.513624000000	1.383301000000	-4.059370000000
H	-4.995985000000	1.261969000000	-6.022586000000

III-TS-ret

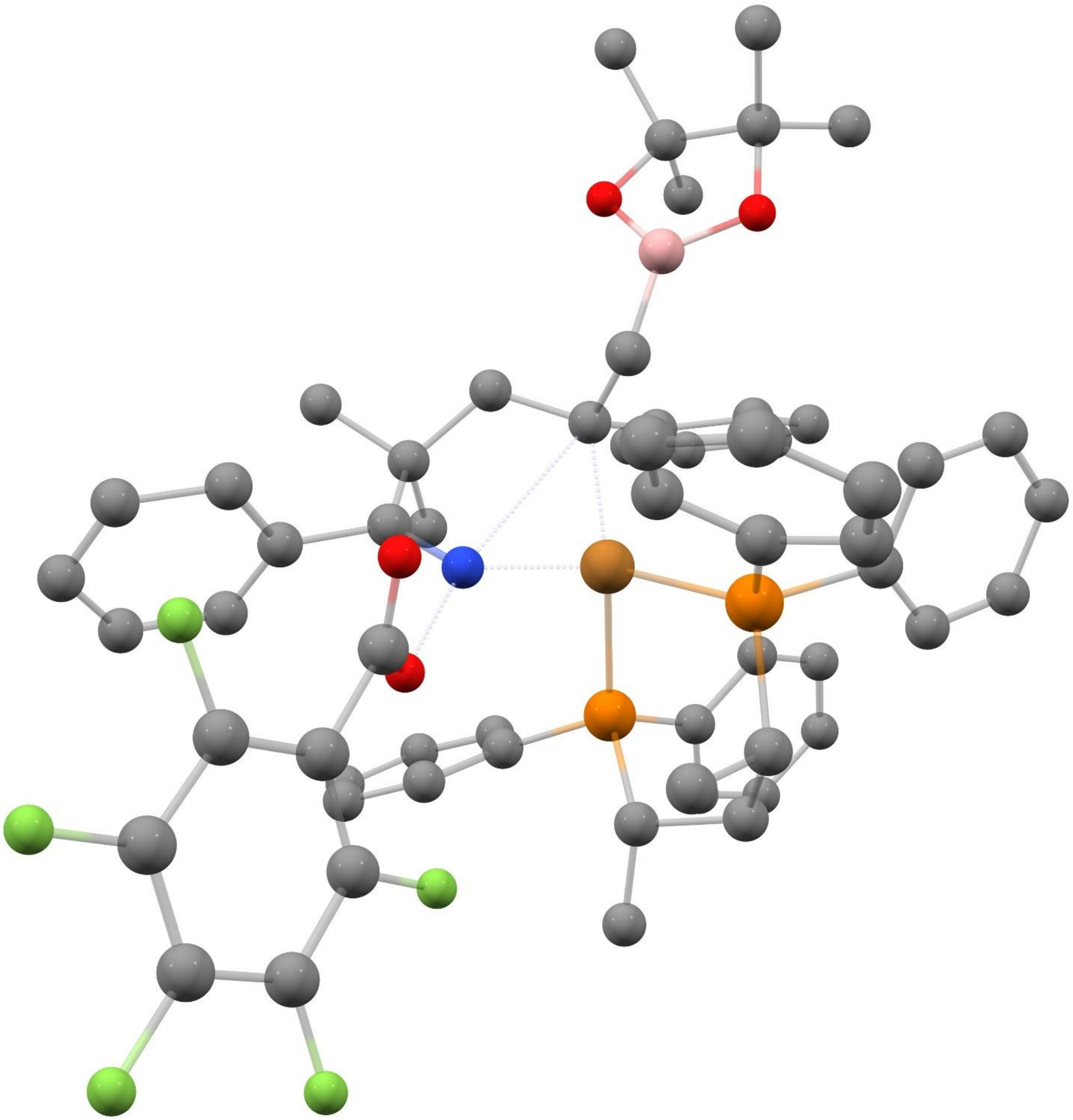

E_{SCF} (wB97X-D/6-31g*-LANL2DZ): -4119.40963034 Hartree

E_{SCF} (wB97X-D/6-311+g-SDD/SMD(THF)):** -4122.82372375 Hartree
Entropy (wB97X-D/6-31g*-LANL2DZ): 381.429 cal mol^{-1} K^{-1}
Thermal Correction to the Energy (wB97X-D/6-31g*-LANL2DZ): 1.184685 Hartree $particle^{-1}$
Thermal Correction to the Enthalpy (wB97X-D/6-31g*-LANL2DZ): 1.185630 Hartree $particle^{-1}$
Thermal Correction to the Gibbs Free Energy (wB97X-D/6-31g*-LANL2DZ): 1.004400 Hartree $particle^{-1}$
Nuclear Repulsion Energy: 15316.5205348668 Hartree
Imaginary Frequency: -309.90 cm^{-1}

C	-3.027303000000	-0.003479000000	-1.227630000000
C	-3.332117000000	0.751368000000	-2.381506000000
C	-3.918816000000	0.166090000000	-0.144105000000
C	-4.425001000000	1.608148000000	-2.439121000000
C	-5.012506000000	1.021200000000	-0.198661000000
C	-5.281435000000	1.756723000000	-1.350502000000
H	-2.710631000000	0.662728000000	-3.263049000000
H	-3.767198000000	-0.395682000000	0.764973000000
H	-4.613311000000	2.161572000000	-3.355877000000
H	-5.654171000000	1.104403000000	0.675373000000
H	-6.136562000000	2.424200000000	-1.402418000000
C	-1.861633000000	-0.930674000000	-1.126258000000
C	-2.103095000000	-2.092156000000	-0.120056000000
H	-1.246055000000	-2.774586000000	-0.164890000000
C	-1.382158000000	-1.522782000000	-2.457960000000
H	-1.249199000000	-2.602970000000	-2.324032000000
H	-2.168980000000	-1.441393000000	-3.221326000000
C	-0.085528000000	-1.024675000000	-3.135028000000
C	1.119504000000	-1.016211000000	-2.151539000000
H	-2.133359000000	-1.753767000000	0.920229000000
C	2.430170000000	-1.520119000000	-2.669566000000
C	4.847634000000	-2.516247000000	-3.631984000000
C	2.810117000000	-2.829116000000	-2.375547000000
C	3.246840000000	-0.717277000000	-3.464426000000
C	4.454685000000	-1.215748000000	-3.940125000000
C	4.022651000000	-3.320162000000	-2.851240000000
H	2.179308000000	-3.443472000000	-1.743056000000
H	2.949163000000	0.300351000000	-3.690424000000
H	5.092595000000	-0.582674000000	-4.550070000000
H	4.323621000000	-4.332192000000	-2.599938000000
H	5.793988000000	-2.901359000000	-3.999665000000
C	0.200112000000	-1.952648000000	-4.330038000000
H	-0.684805000000	-1.968948000000	-4.974468000000
H	0.399453000000	-2.979983000000	-4.008584000000
H	1.049167000000	-1.612352000000	-4.928865000000

C	-0.199017000000	0.418229000000	-3.653089000000
H	-0.536178000000	1.088812000000	-2.861257000000
H	-0.920960000000	0.450843000000	-4.476760000000
H	0.755431000000	0.795107000000	-4.036940000000
N	0.993660000000	-0.545577000000	-0.976952000000
O	2.689374000000	-0.334127000000	-0.360199000000
C	3.129784000000	-1.336196000000	0.332957000000
O	2.559069000000	-2.380403000000	0.590859000000
C	4.560807000000	-1.108060000000	0.783881000000
C	7.237499000000	-0.759234000000	1.571821000000
C	5.461448000000	-2.174544000000	0.783764000000
C	5.047171000000	0.138401000000	1.175384000000
C	6.363380000000	0.319382000000	1.578166000000
C	6.786654000000	-2.008528000000	1.168677000000
F	5.093737000000	-3.386923000000	0.387939000000
F	7.627761000000	-3.039719000000	1.148011000000
F	8.500488000000	-0.594890000000	1.947143000000
F	6.791424000000	1.519304000000	1.965703000000
F	4.260178000000	1.217173000000	1.198476000000
C	1.726402000000	0.938686000000	3.239259000000
H	2.490448000000	1.672139000000	3.522150000000
H	1.702174000000	0.155122000000	3.999184000000
H	2.044296000000	0.478606000000	2.298042000000
C	0.362268000000	1.632518000000	3.098775000000
H	-0.033279000000	1.830226000000	4.103831000000
C	0.526981000000	3.004504000000	2.410666000000
P	-0.845433000000	0.452420000000	2.278613000000
H	-0.431455000000	3.526407000000	2.313730000000
H	1.110915000000	3.611322000000	3.115977000000
C	1.268560000000	3.070029000000	1.056835000000
H	2.018433000000	2.272912000000	1.018257000000
C	1.988750000000	4.417760000000	0.941604000000
P	0.119778000000	2.653243000000	-0.362315000000
H	2.445437000000	4.563569000000	-0.041199000000
H	1.298765000000	5.247313000000	1.130235000000
H	2.785170000000	4.471540000000	1.693077000000
Cu	-0.583774000000	0.389597000000	-0.121940000000
B	-3.460270000000	-2.826639000000	-0.433129000000
H	-5.564237000000	-5.080103000000	1.446340000000
C	-5.512168000000	-5.082890000000	0.353447000000
H	-6.345612000000	-5.678776000000	-0.032672000000
H	-4.572896000000	-5.563057000000	0.061602000000
C	-5.569126000000	-3.638834000000	-0.147997000000
C	-5.234439000000	-3.496560000000	-1.677723000000
C	-6.875685000000	-2.981405000000	0.278659000000
O	-4.482452000000	-2.934879000000	0.478833000000

C	-5.577100000000	-4.717513000000	-2.521748000000
C	-5.827924000000	-2.237470000000	-2.316252000000
O	-3.805467000000	-3.324500000000	-1.667057000000
H	-5.039992000000	-5.606215000000	-2.182763000000
H	-6.653249000000	-4.921518000000	-2.486700000000
H	-5.299592000000	-4.530876000000	-3.563643000000
H	-5.386716000000	-2.108482000000	-3.309070000000
H	-6.914634000000	-2.318241000000	-2.426275000000
H	-5.595724000000	-1.341270000000	-1.733703000000
H	-6.857761000000	-1.907467000000	0.079137000000
H	-7.726588000000	-3.426874000000	-0.248986000000
H	-7.026528000000	-3.126434000000	1.353194000000
C	-1.175955000000	3.941052000000	-0.295536000000
C	-2.472776000000	3.524245000000	0.015934000000
C	-0.931500000000	5.308203000000	-0.495640000000
C	-3.501329000000	4.453650000000	0.157381000000
C	-1.959395000000	6.233513000000	-0.361144000000
C	-3.245286000000	5.807297000000	-0.025696000000
H	-2.690596000000	2.467590000000	0.143083000000
H	0.060838000000	5.646583000000	-0.777913000000
H	-4.499555000000	4.102084000000	0.397950000000
H	-1.759275000000	7.289157000000	-0.520071000000
H	-4.046263000000	6.533577000000	0.079399000000
C	1.119921000000	3.053821000000	-1.841542000000
C	2.430838000000	2.568223000000	-1.927665000000
C	0.574843000000	3.727208000000	-2.939794000000
C	3.188905000000	2.792486000000	-3.073410000000
C	1.328736000000	3.928630000000	-4.092462000000
C	2.641152000000	3.469215000000	-4.160706000000
H	2.864261000000	1.999093000000	-1.110359000000
H	-0.447888000000	4.090082000000	-2.901086000000
H	4.209747000000	2.423152000000	-3.117048000000
H	0.888462000000	4.450358000000	-4.937118000000
H	3.232972000000	3.635795000000	-5.055919000000
C	-0.478807000000	-1.096221000000	3.181163000000
C	0.214713000000	-2.095919000000	2.500063000000
C	-0.765086000000	-1.263180000000	4.542678000000
C	0.629897000000	-3.246075000000	3.167919000000
C	-0.365111000000	-2.417761000000	5.204051000000
C	0.335916000000	-3.410068000000	4.516722000000
H	0.460697000000	-1.973452000000	1.450545000000
H	-1.306014000000	-0.491307000000	5.083930000000
H	1.192363000000	-3.998118000000	2.624486000000
H	-0.593922000000	-2.543213000000	6.258475000000
H	0.654402000000	-4.307740000000	5.039025000000
C	-2.499129000000	0.907114000000	2.939273000000

C	-3.445560000000	-0.102093000000	3.168383000000
C	-2.897647000000	2.236589000000	3.104397000000
C	-4.746700000000	0.211537000000	3.544115000000
C	-4.200556000000	2.551649000000	3.481871000000
C	-5.130665000000	1.541372000000	3.701601000000
H	-3.173171000000	-1.145620000000	3.039619000000
H	-2.202487000000	3.048167000000	2.927116000000
H	-5.462184000000	-0.589217000000	3.704881000000
H	-4.484520000000	3.593469000000	3.598326000000
H	-6.147751000000	1.786857000000	3.992630000000

IV-(*S*)

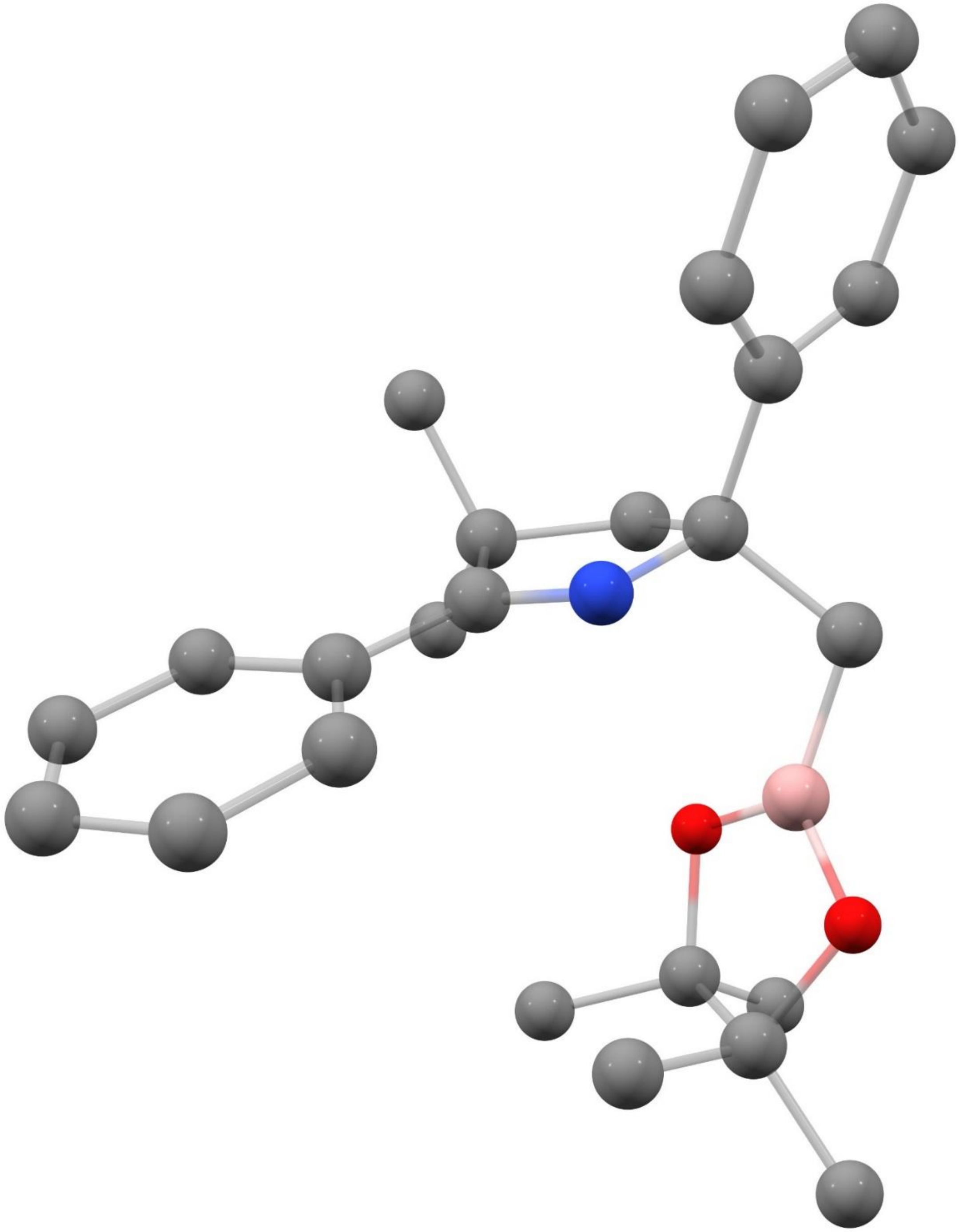

E$_{SCF}$ (wB97X-D/6-31g*): -1201.75879826 Hartree
E$_{SCF}$ (wB97X-D/6-311+g/SMD(THF)):** -1202.51816136 Hartree
Entropy (wB97X-D/6-31g*): 176.994 cal mol^{-1} K^{-1}
Thermal Correction to the Energy (wB97X-D/6-31g*): 0.558630 Hartree particle^{-1}
Thermal Correction to the Enthalpy (wB97X-D/6-31g*): 0.559574 Hartree particle^{-1}
Thermal Correction to the Gibbs Free Energy (wB97X-D/6-31g*): 0.475479 Hartree particle^{-1}

Nuclear Repulsion Energy: 2903.5115764206 Hartree

C	-1.450113000000	-0.837411000000	0.163816000000
C	-1.328999000000	-0.474734000000	1.666808000000
C	-1.109624000000	1.052607000000	1.684195000000
H	-0.449833000000	-0.973199000000	2.088551000000
H	-2.201581000000	-0.773108000000	2.254520000000
C	-0.546784000000	1.232380000000	0.268466000000
N	-0.775964000000	0.265684000000	-0.534680000000
C	0.241773000000	2.390765000000	-0.221609000000
C	1.808753000000	4.485242000000	-1.228060000000
C	0.172667000000	3.665795000000	0.345131000000
C	1.105546000000	2.180382000000	-1.306175000000
C	1.884656000000	3.216850000000	-1.801492000000
C	0.947893000000	4.706843000000	-0.158437000000
H	-0.498417000000	3.862238000000	1.173698000000
H	1.150293000000	1.186105000000	-1.740909000000
H	2.553309000000	3.035518000000	-2.638068000000
H	0.876829000000	5.693876000000	0.288759000000
H	2.418014000000	5.297003000000	-1.614446000000
C	-2.901344000000	-0.949162000000	-0.301012000000
C	-5.565335000000	-1.211044000000	-1.148558000000
C	-3.765556000000	-1.854378000000	0.322137000000
C	-3.388743000000	-0.185292000000	-1.361859000000
C	-4.711552000000	-0.313402000000	-1.780106000000
C	-5.085040000000	-1.985152000000	-0.094722000000
H	-3.402522000000	-2.467388000000	1.143793000000
H	-2.721717000000	0.510567000000	-1.859575000000
H	-5.073234000000	0.292977000000	-2.605780000000
H	-5.740563000000	-2.692546000000	0.405357000000
H	-6.596724000000	-1.309588000000	-1.474192000000
C	-0.153890000000	1.460425000000	2.809119000000
H	0.827736000000	0.996783000000	2.675432000000
H	-0.011411000000	2.543127000000	2.869273000000
H	-0.557637000000	1.124667000000	3.771124000000
C	-2.451838000000	1.798437000000	1.816715000000
H	-3.160292000000	1.477460000000	1.047184000000
H	-2.894404000000	1.586343000000	2.796670000000
H	-2.330171000000	2.883330000000	1.734350000000
C	-0.686722000000	-2.136871000000	-0.205122000000
H	-1.009933000000	-2.941817000000	0.466499000000
H	-0.966251000000	-2.423165000000	-1.224082000000
B	0.876200000000	-1.937822000000	-0.132902000000
O	1.665126000000	-1.696056000000	-1.226790000000
O	1.596482000000	-1.988013000000	1.036748000000
C	3.040251000000	-1.775932000000	-0.808120000000

C	2.942054000000	-1.556842000000	0.744969000000
C	3.912243000000	-2.391466000000	1.570949000000
H	3.759454000000	-2.186059000000	2.634600000000
H	3.763452000000	-3.460968000000	1.408898000000
H	4.948151000000	-2.138357000000	1.319859000000
C	3.041901000000	-0.087929000000	1.156143000000
H	2.811520000000	-0.004613000000	2.222381000000
H	4.049171000000	0.307763000000	0.993118000000
H	2.331769000000	0.533303000000	0.602264000000
C	3.841867000000	-0.714822000000	-1.550830000000
H	3.426322000000	0.283742000000	-1.398163000000
H	4.885226000000	-0.715135000000	-1.216302000000
H	3.826856000000	-0.930087000000	-2.623366000000
C	3.536409000000	-3.171211000000	-1.193520000000
H	2.964812000000	-3.949968000000	-0.679007000000
H	3.399655000000	-3.308821000000	-2.269835000000
H	4.597169000000	-3.302606000000	-0.957956000000

IV-(*R*)

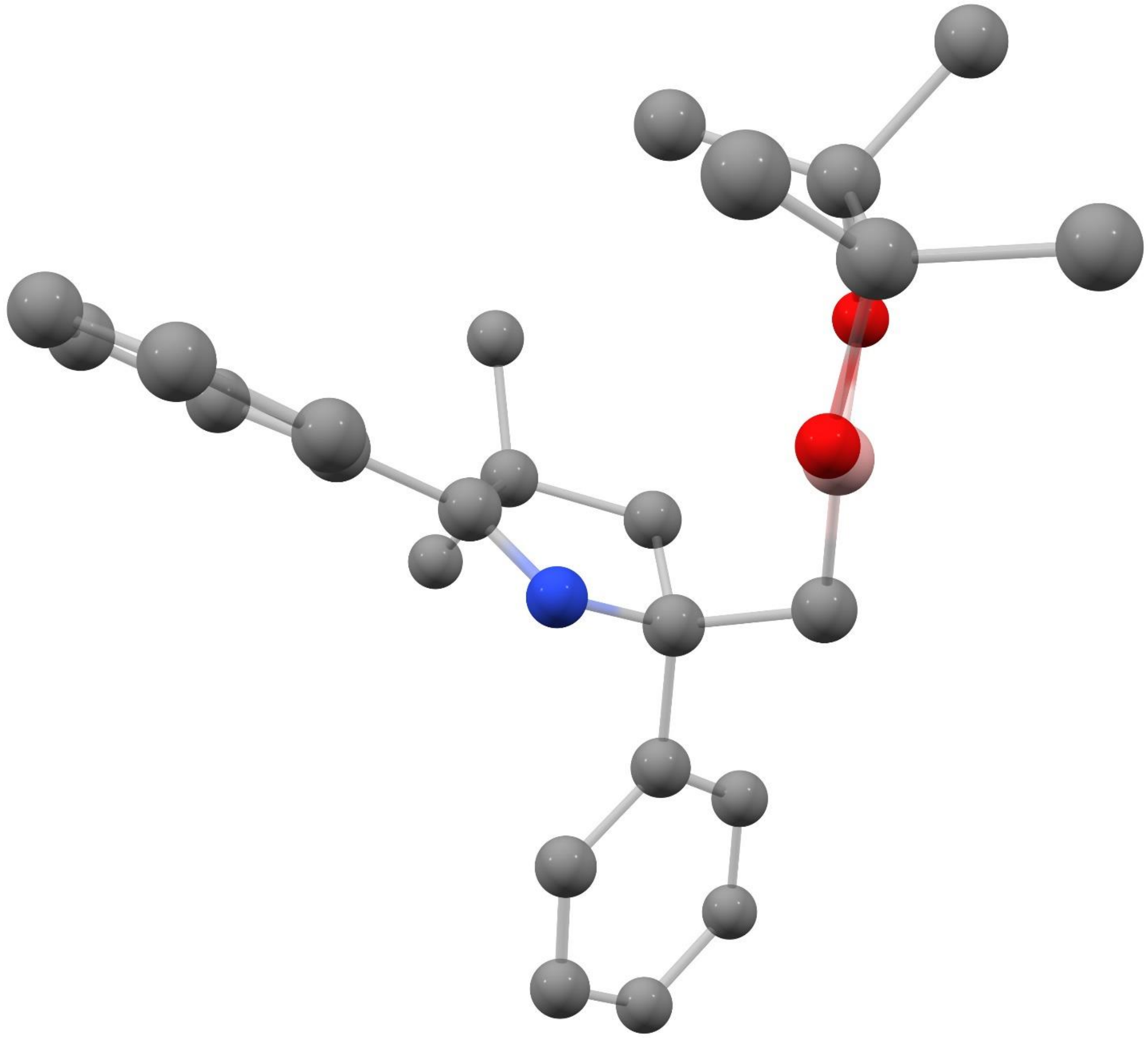

E$_{SCF}$ (wB97X-D/6-31g*): -1201.75879826 Hartree
E$_{SCF}$ (wB97X-D/6-311+g/SMD(THF)):** -1202.51816136 Hartree
Entropy (wB97X-D/6-31g*): 176.994 cal mol^{-1} K^{-1}
Thermal Correction to the Energy (wB97X-D/6-31g*): 0.558630 Hartree particle^{-1}
Thermal Correction to the Enthalpy (wB97X-D/6-31g*): 0.559574 Hartree particle^{-1}
Thermal Correction to the Gibbs Free Energy (wB97X-D/6-31g*): 0.475479 Hartree particle^{-1}
Nuclear Repulsion Energy: 2903.5115764206 Hartree

C	1.450113000000	-0.837411000000	0.163816000000
C	1.328999000000	-0.474734000000	1.666808000000
C	1.109624000000	1.052607000000	1.684195000000
H	0.449833000000	-0.973199000000	2.088551000000

H	2.201581000000	-0.773108000000	2.254520000000
C	0.546784000000	1.232380000000	0.268466000000
N	0.775964000000	0.265684000000	-0.534680000000
C	-0.241773000000	2.390765000000	-0.221609000000
C	-1.808753000000	4.485242000000	-1.228060000000
C	-0.172667000000	3.665795000000	0.345131000000
C	-1.105546000000	2.180382000000	-1.306175000000
C	-1.884656000000	3.216850000000	-1.801492000000
C	-0.947893000000	4.706843000000	-0.158437000000
H	0.498417000000	3.862238000000	1.173698000000
H	-1.150293000000	1.186105000000	-1.740909000000
H	-2.553309000000	3.035518000000	-2.638068000000
H	-0.876829000000	5.693876000000	0.288759000000
H	-2.418014000000	5.297003000000	-1.614446000000
C	2.901344000000	-0.949162000000	-0.301012000000
C	5.565335000000	-1.211044000000	-1.148558000000
C	3.765556000000	-1.854378000000	0.322137000000
C	3.388743000000	-0.185292000000	-1.361859000000
C	4.711552000000	-0.313402000000	-1.780106000000
C	5.085040000000	-1.985152000000	-0.094722000000
H	3.402522000000	-2.467388000000	1.143793000000
H	2.721717000000	0.510567000000	-1.859575000000
H	5.073234000000	0.292977000000	-2.605780000000
H	5.740563000000	-2.692546000000	0.405357000000
H	6.596724000000	-1.309588000000	-1.474192000000
C	0.153890000000	1.460425000000	2.809119000000
H	-0.827736000000	0.996783000000	2.675432000000
H	0.011411000000	2.543127000000	2.869273000000
H	0.557637000000	1.124667000000	3.771124000000
C	2.451838000000	1.798437000000	1.816715000000
H	3.160292000000	1.477460000000	1.047184000000
H	2.894404000000	1.586343000000	2.796670000000
H	2.330171000000	2.883330000000	1.734350000000
C	0.686722000000	-2.136871000000	-0.205122000000
H	1.009933000000	-2.941817000000	0.466499000000
H	0.966251000000	-2.423165000000	-1.224082000000
B	-0.876200000000	-1.937822000000	-0.132902000000
O	-1.665126000000	-1.696056000000	-1.226790000000
O	-1.596482000000	-1.988013000000	1.036748000000
C	-3.040251000000	-1.775932000000	-0.808120000000
C	-2.942054000000	-1.556842000000	0.744969000000
C	-3.912243000000	-2.391466000000	1.570949000000
H	-3.759454000000	-2.186059000000	2.634600000000
H	-3.763452000000	-3.460968000000	1.408898000000
H	-4.948151000000	-2.138357000000	1.319859000000
C	-3.041901000000	-0.087929000000	1.156143000000

H	-2.811520000000	-0.004613000000	2.222381000000
H	-4.049171000000	0.307763000000	0.993118000000
H	-2.331769000000	0.533303000000	0.602264000000
C	-3.841867000000	-0.714822000000	-1.550830000000
H	-3.426322000000	0.283742000000	-1.398163000000
H	-4.885226000000	-0.715135000000	-1.216302000000
H	-3.826856000000	-0.930087000000	-2.623366000000
C	-3.536409000000	-3.171211000000	-1.193520000000
H	-2.964812000000	-3.949968000000	-0.679007000000
H	-3.399655000000	-3.308821000000	-2.269835000000
H	-4.597169000000	-3.302606000000	-0.957956000000

[Cu]–OBz^{F}

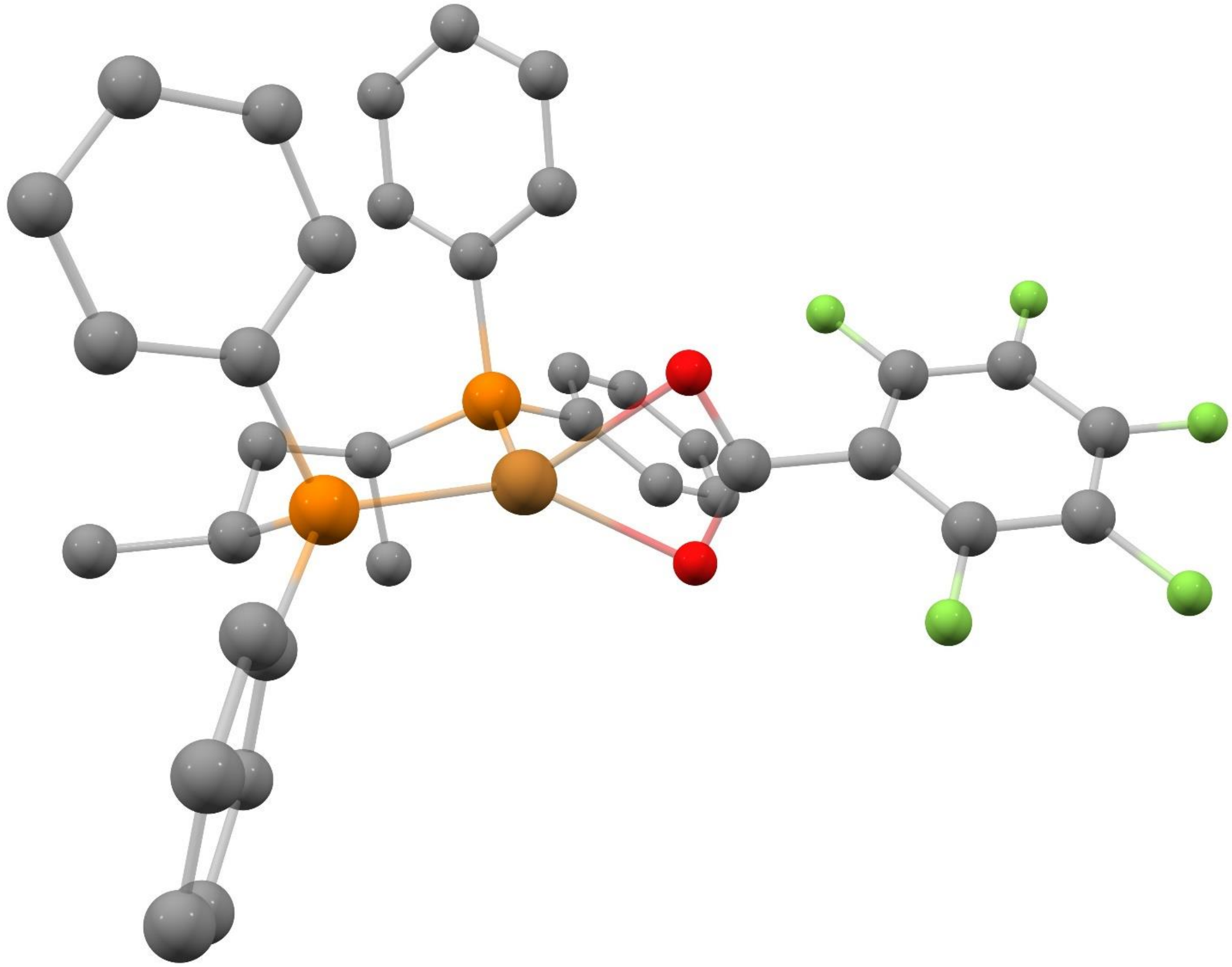

E$_{SCF}$ (wB97X-D/6-31g*-LANL2DZ): -2917.78415464 Hartree
E$_{SCF}$ (wB97X-D/6-311+g-SDD/SMD(THF)):** -2920.41632673 Hartree
Entropy (wB97X-D/6-31g*-LANL2DZ): 176.994 cal mol^{-1} K^{-1}
Thermal Correction to the Energy (wB97X-D/6-31g*-LANL2DZ): 0.626355 Hartree particle^{-1}
Thermal Correction to the Enthalpy (wB97X-D/6-31g*-LANL2DZ): 0.627299 Hartree particle^{-1}
Thermal Correction to the Gibbs Free Energy (wB97X-D/6-31g*-LANL2DZ): 0.500742 Hartree particle^{-1}
Nuclear Repulsion Energy: 6859.5477047772 Hartree

C	1.437752000000	-1.666093000000	-3.376706000000
H	2.315422000000	-1.821290000000	-4.015548000000
H	0.586821000000	-2.174605000000	-3.837853000000
H	1.209994000000	-0.593443000000	-3.365392000000
C	1.703776000000	-2.209459000000	-1.967450000000
H	1.727288000000	-3.304829000000	-2.040345000000

C	3.079145000000	-1.772090000000	-1.405081000000
H	3.172037000000	-2.066164000000	-0.351229000000
H	3.825714000000	-2.372421000000	-1.942462000000
C	3.531852000000	-0.299188000000	-1.563019000000
H	3.175398000000	0.071220000000	-2.531317000000
C	5.064071000000	-0.242605000000	-1.562263000000
H	5.439882000000	0.784137000000	-1.597616000000
H	5.470661000000	-0.726642000000	-0.668279000000
H	5.455599000000	-0.774497000000	-2.437080000000
P	0.293105000000	-1.733923000000	-0.855286000000
P	2.729145000000	0.842125000000	-0.305843000000
C	-1.186982000000	-2.386145000000	-1.701114000000
C	-2.124514000000	-1.468084000000	-2.183462000000
C	-1.405151000000	-3.756806000000	-1.877449000000
C	-3.264163000000	-1.920319000000	-2.845437000000
C	-2.542417000000	-4.203630000000	-2.539994000000
C	-3.471978000000	-3.284122000000	-3.025657000000
H	-1.966790000000	-0.403499000000	-2.024888000000
H	-0.692236000000	-4.475745000000	-1.480680000000
H	-3.992108000000	-1.203441000000	-3.213222000000
H	-2.709679000000	-5.268558000000	-2.672018000000
H	-4.363151000000	-3.635027000000	-3.537862000000
C	0.448100000000	-2.782550000000	0.635633000000
C	-0.332140000000	-2.404480000000	1.737899000000
C	1.305580000000	-3.879531000000	0.752712000000
C	-0.254706000000	-3.119110000000	2.928553000000
C	1.389346000000	-4.585117000000	1.951018000000
C	0.609359000000	-4.207279000000	3.039582000000
H	-0.978798000000	-1.532276000000	1.667854000000
H	1.924235000000	-4.190840000000	-0.083984000000
H	-0.864851000000	-2.817034000000	3.774462000000
H	2.065889000000	-5.430870000000	2.031644000000
H	0.675323000000	-4.758317000000	3.973211000000
C	3.449558000000	0.294237000000	1.285111000000
C	2.628106000000	-0.433020000000	2.152069000000
C	4.787138000000	0.513116000000	1.642059000000
C	3.138037000000	-0.967981000000	3.332148000000
C	5.291747000000	-0.007646000000	2.828263000000
C	4.470830000000	-0.758440000000	3.669414000000
H	1.580031000000	-0.579723000000	1.912145000000
H	5.431142000000	1.104418000000	0.997921000000
H	2.485074000000	-1.543376000000	3.981029000000
H	6.328729000000	0.171124000000	3.096847000000
H	4.871043000000	-1.169425000000	4.591656000000
C	3.479083000000	2.480744000000	-0.629693000000
C	3.723732000000	2.913139000000	-1.937848000000

C	3.695929000000	3.373201000000	0.426103000000
C	4.198590000000	4.197996000000	-2.182360000000
C	4.168983000000	4.658169000000	0.179438000000
C	4.425787000000	5.073234000000	-1.124243000000
H	3.543682000000	2.250588000000	-2.779873000000
H	3.495790000000	3.064018000000	1.447940000000
H	4.387603000000	4.515207000000	-3.203526000000
H	4.334145000000	5.337035000000	1.010649000000
H	4.794516000000	6.076355000000	-1.315464000000
Cu	0.481348000000	0.527373000000	-0.363173000000
O	-1.304288000000	1.578730000000	-0.843088000000
C	-1.770749000000	1.290031000000	0.291441000000
O	-1.105964000000	0.793223000000	1.228465000000
C	-3.253699000000	1.489668000000	0.501240000000
C	-6.013985000000	1.798200000000	0.852671000000
C	-3.854462000000	2.736449000000	0.381122000000
C	-4.064951000000	0.401031000000	0.799888000000
C	-5.436326000000	0.539990000000	0.968585000000
C	-5.222456000000	2.902031000000	0.561178000000
F	-3.121017000000	3.814106000000	0.111245000000
F	-5.779894000000	4.107354000000	0.457713000000
F	-7.325543000000	1.945336000000	1.019058000000
F	-6.199435000000	-0.518640000000	1.237018000000
F	-3.541462000000	-0.822582000000	0.898648000000

NMR Spectra

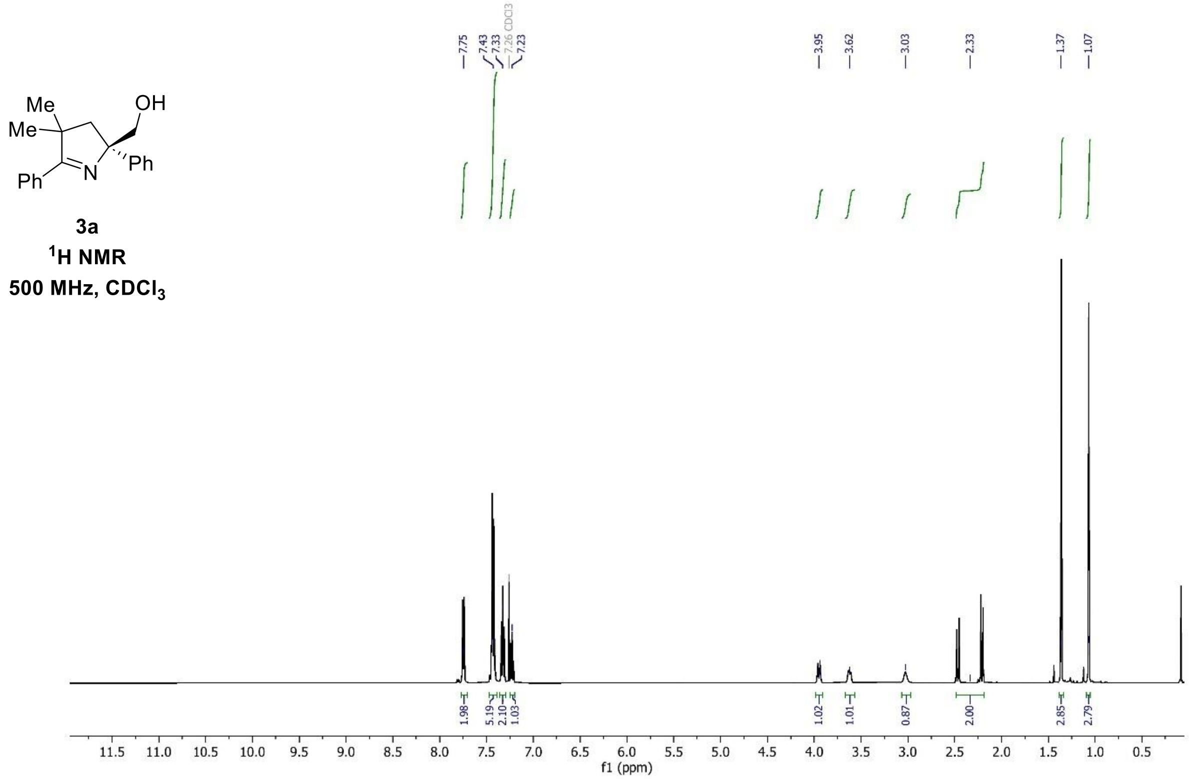

3a
^{1}H NMR
500 MHz, $CDCl_3$

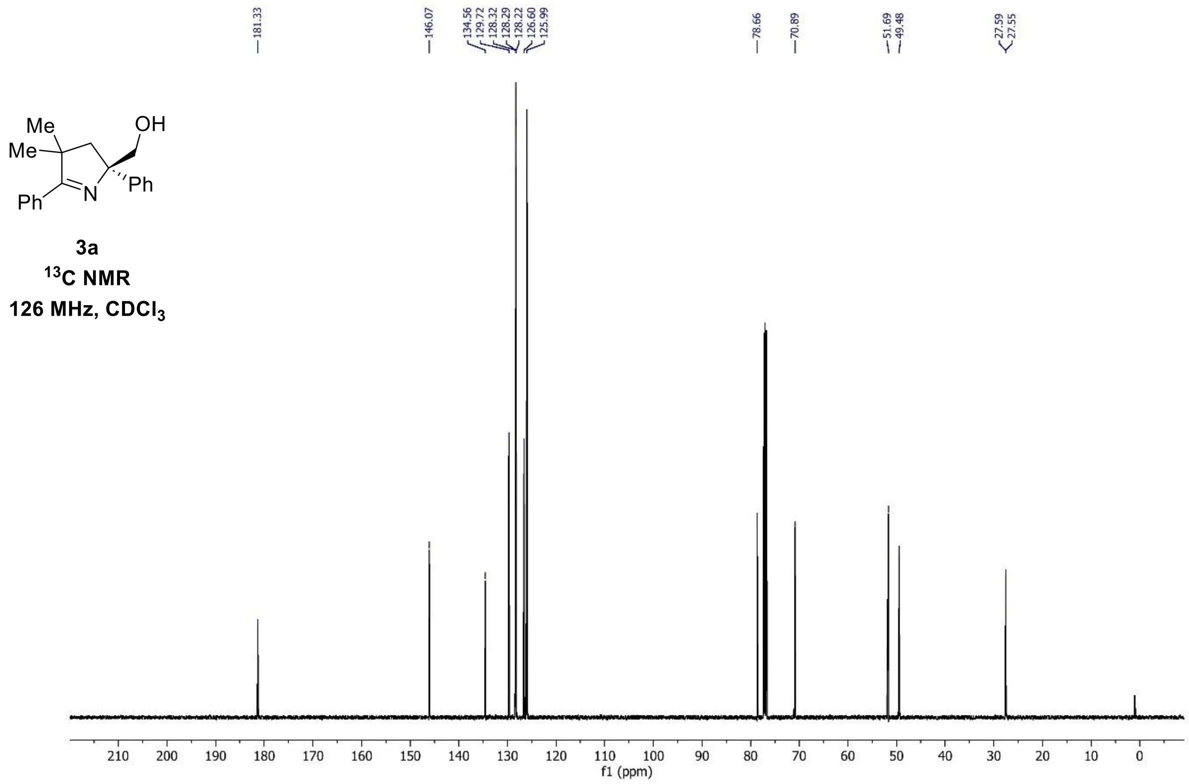

3a
^{13}C NMR
126 MHz, $CDCl_3$

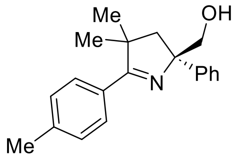

3b
^{1}H NMR
500 MHz, $CDCl_3$

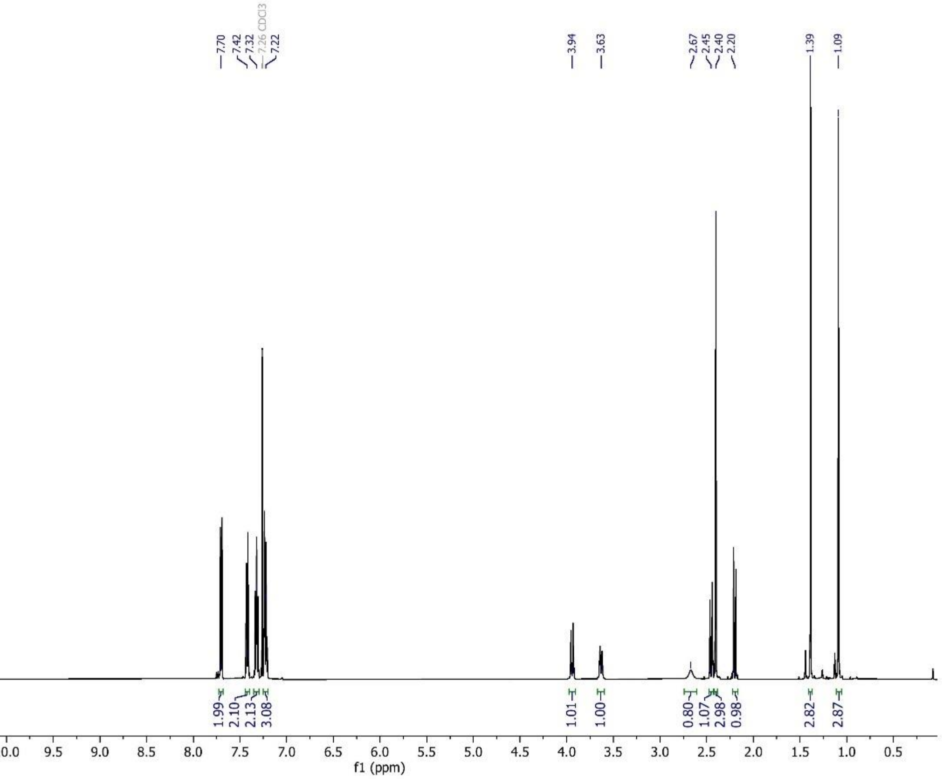

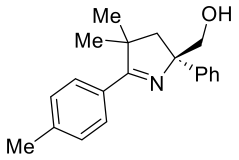

3b
^{13}C NMR
126 MHz, $CDCl_3$

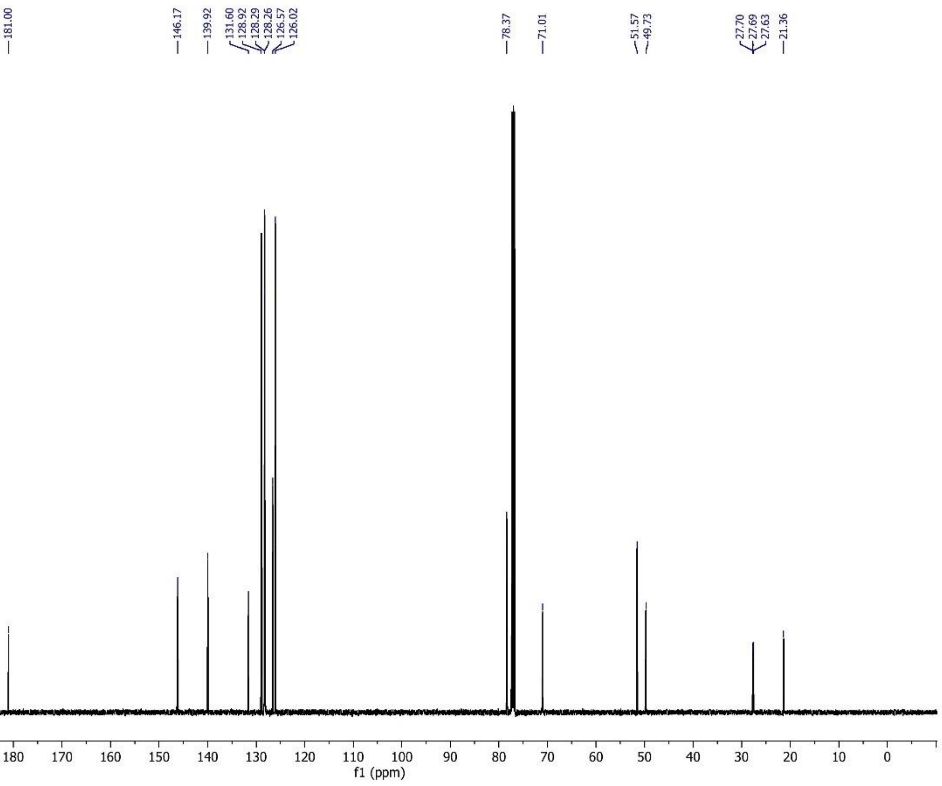

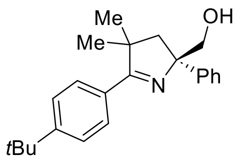

3c

^{1}H NMR

500 MHz, $CDCl_3$

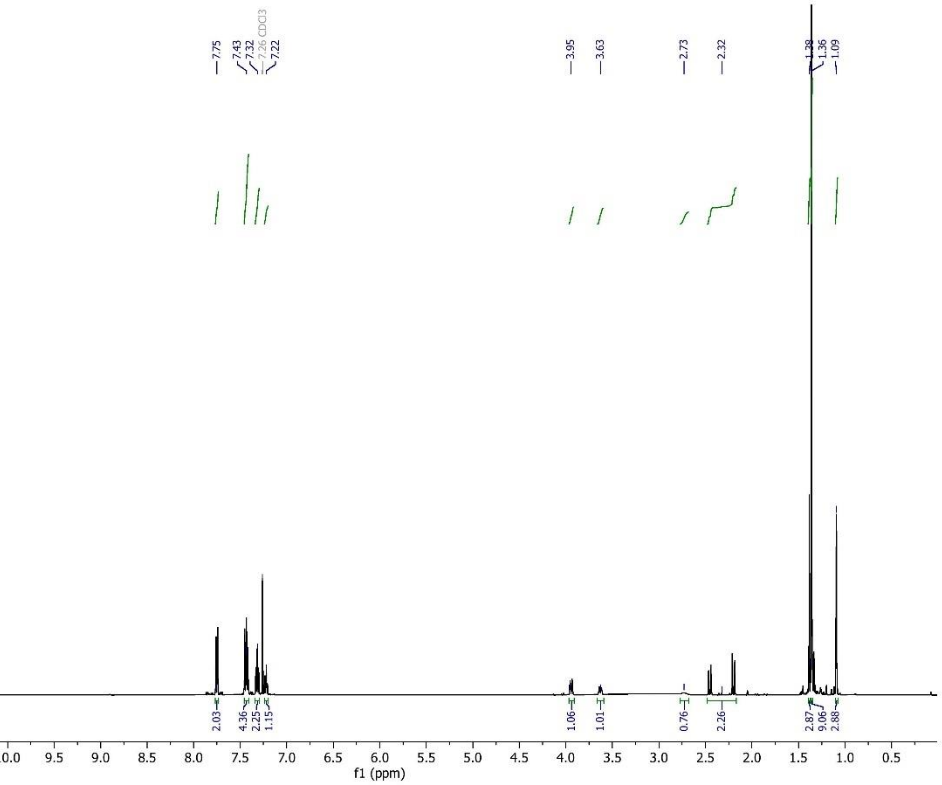

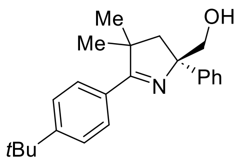

3c

^{13}C NMR

126 MHz, $CDCl_3$

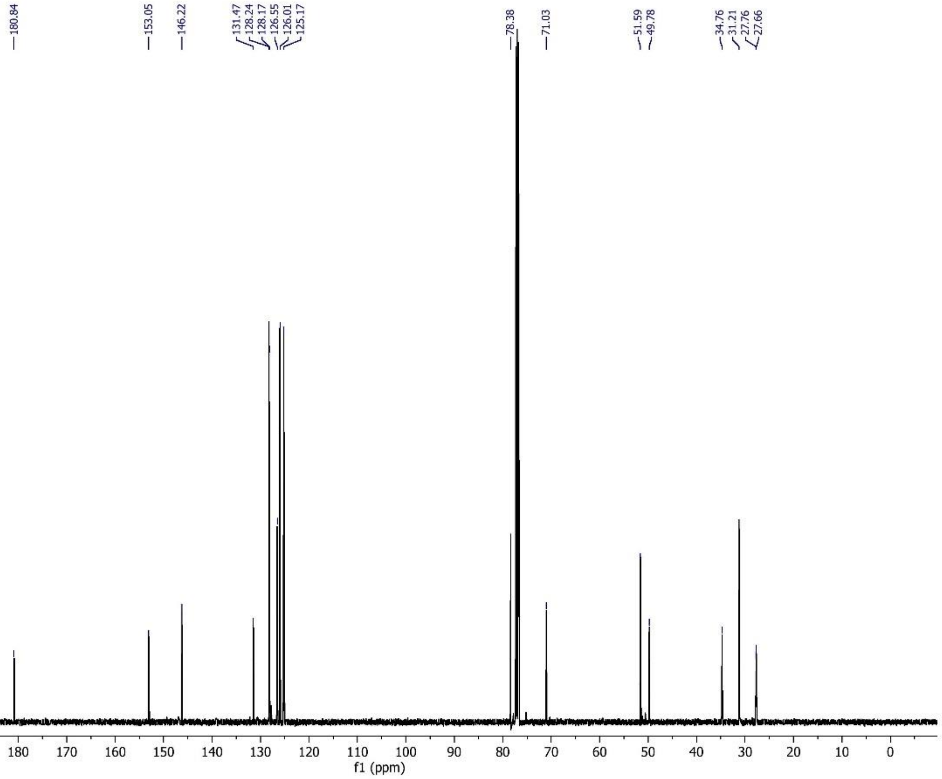

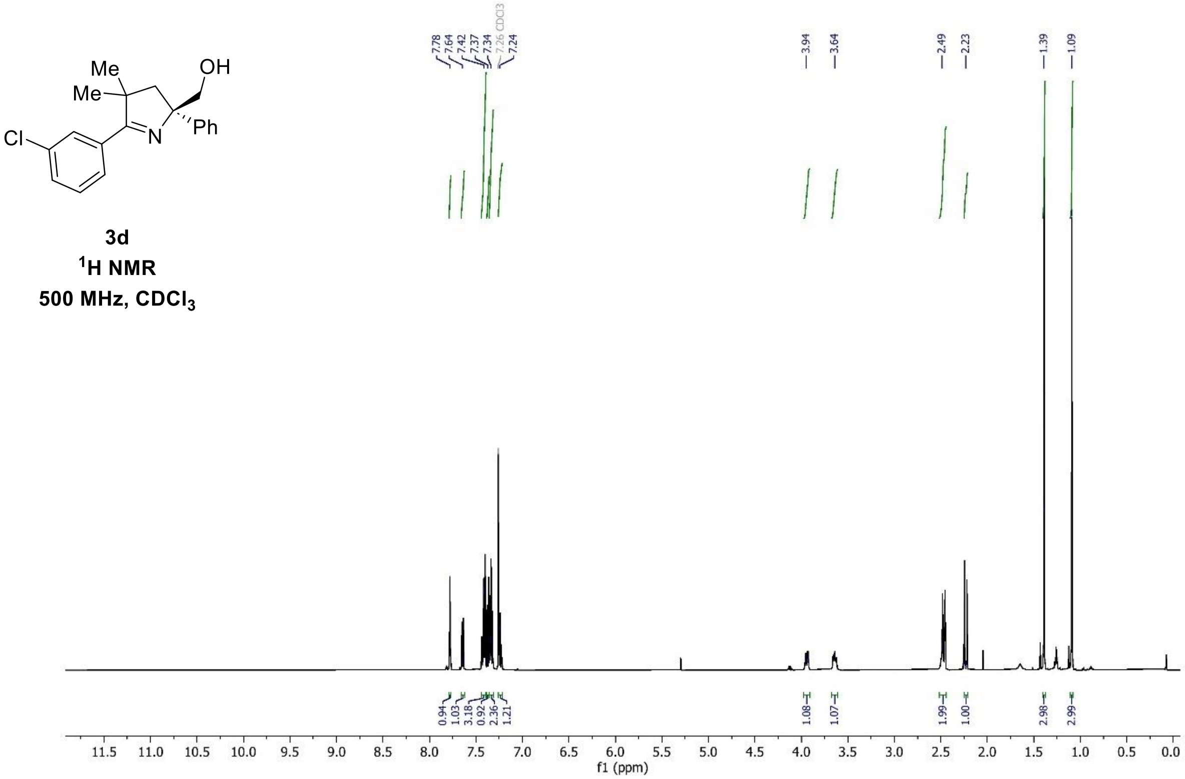

3d
^{1}H NMR
500 MHz, $CDCl_3$

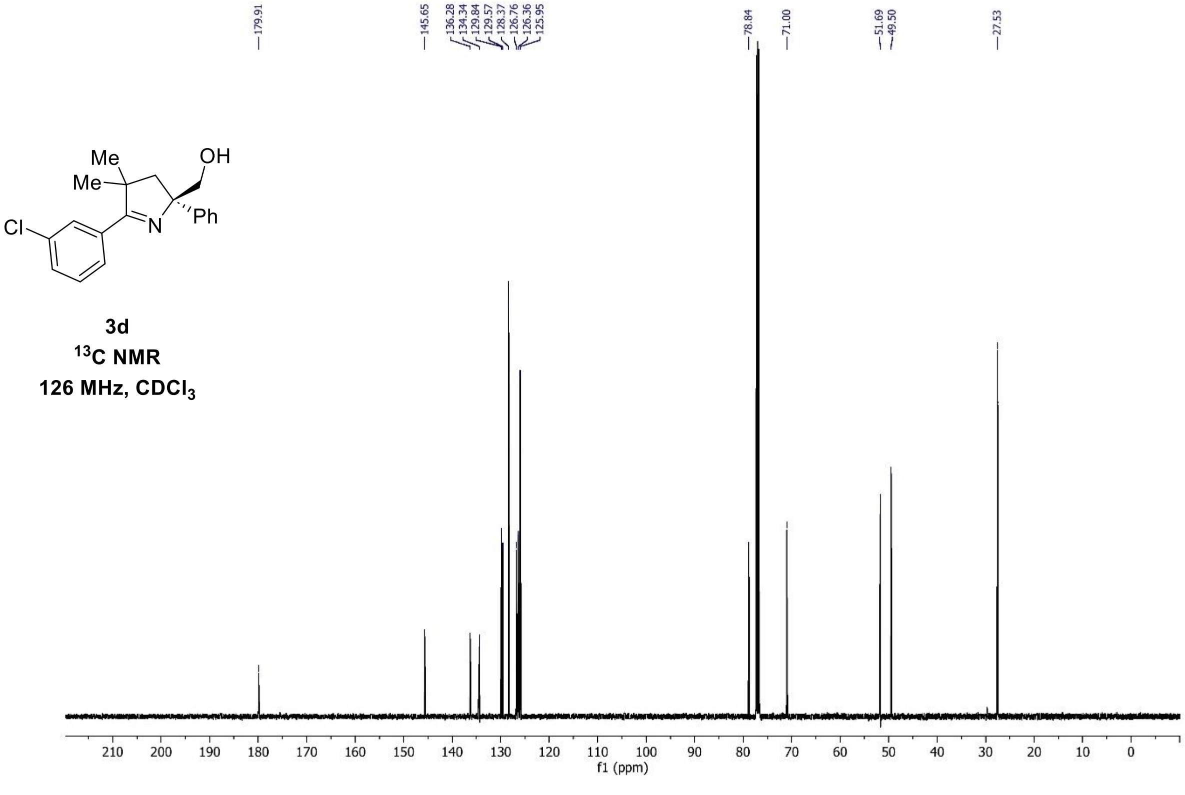

3d
^{13}C NMR
126 MHz, $CDCl_3$

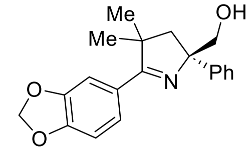

3e
^{1}H NMR
500 MHz, $CDCl_3$

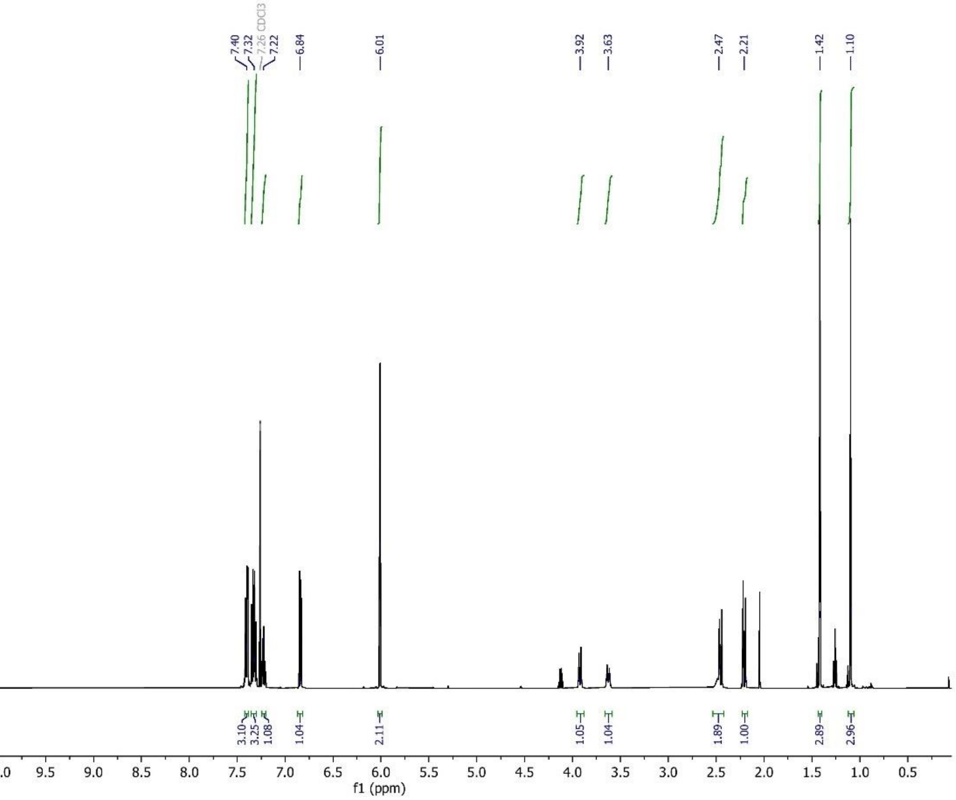

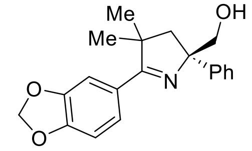

3e
^{13}C NMR
126 MHz, $CDCl_3$

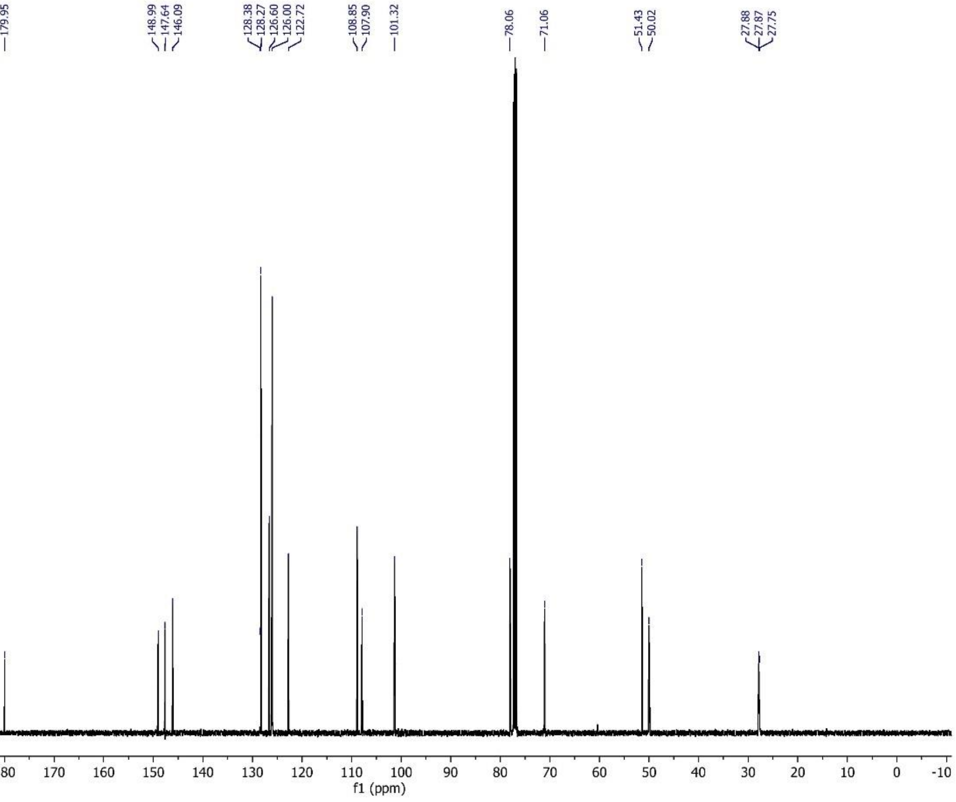

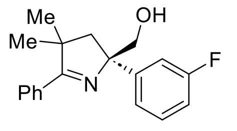

3f
^{1}H NMR
500 MHz, $CDCl_3$

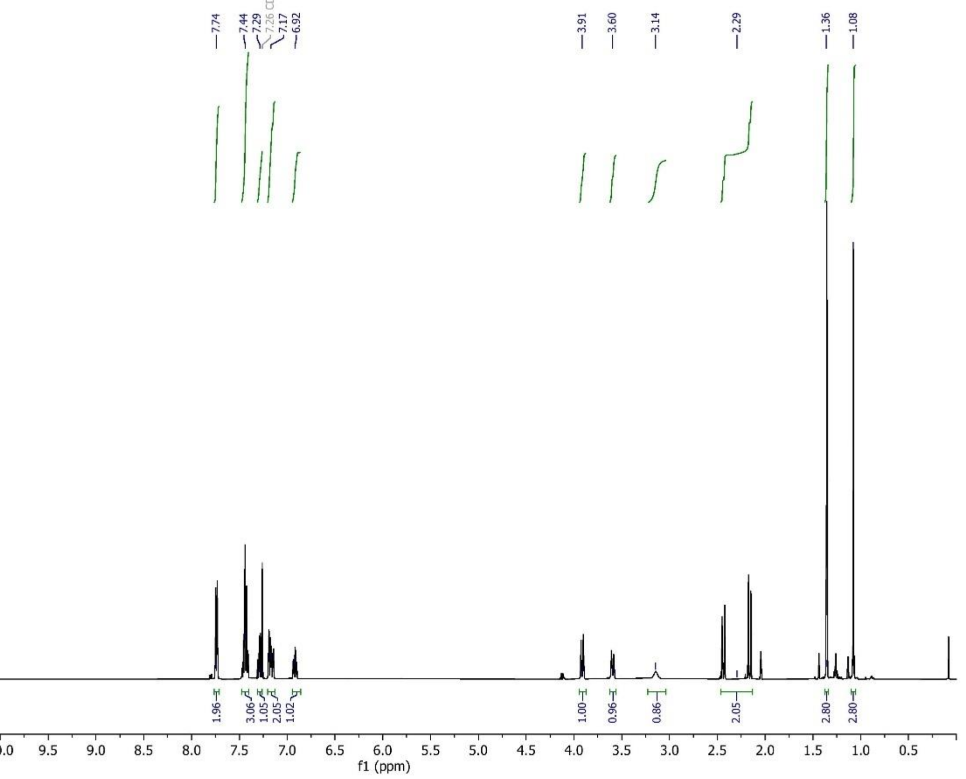

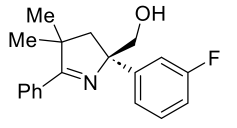

3f
^{13}C NMR
126 MHz, $CDCl_3$

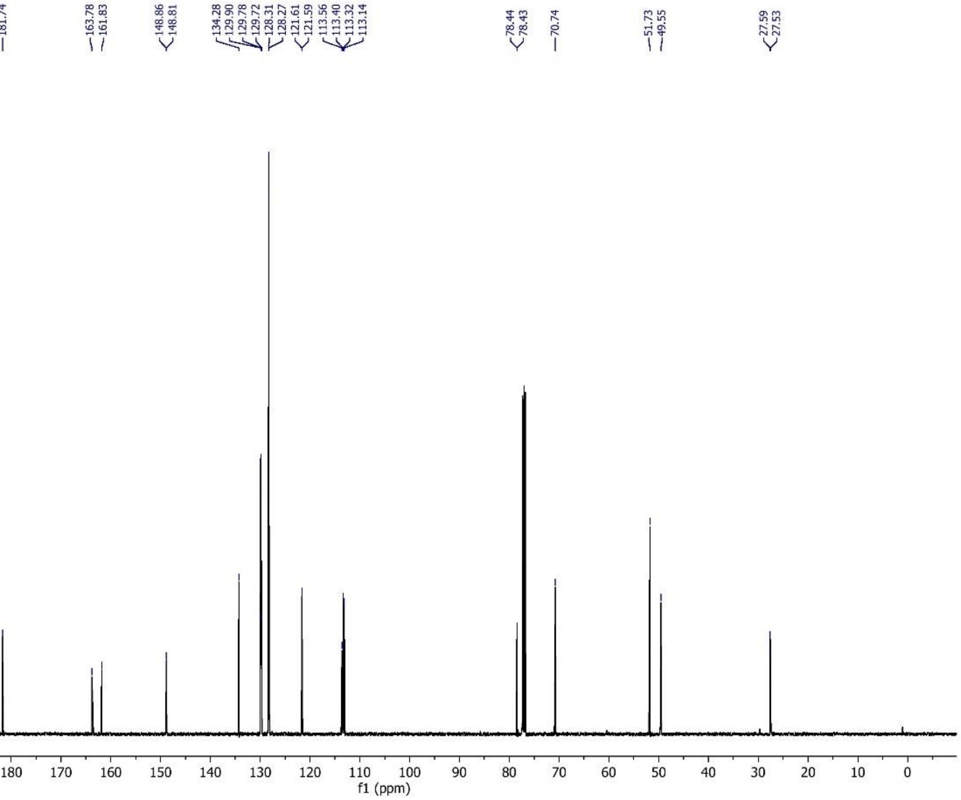

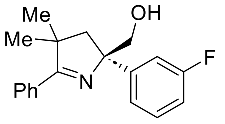

3f
^{19}F NMR
376 MHz, $CDCl_3$

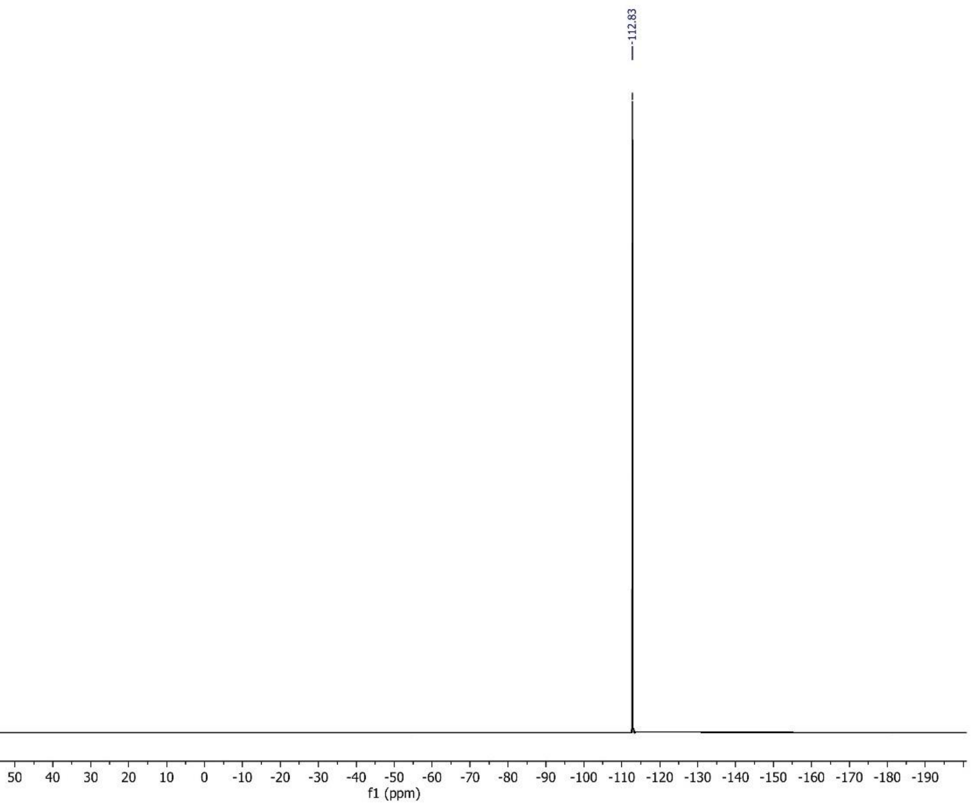

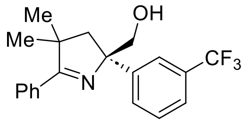

3g
^{1}H NMR
500 MHz, CDCl$_3$

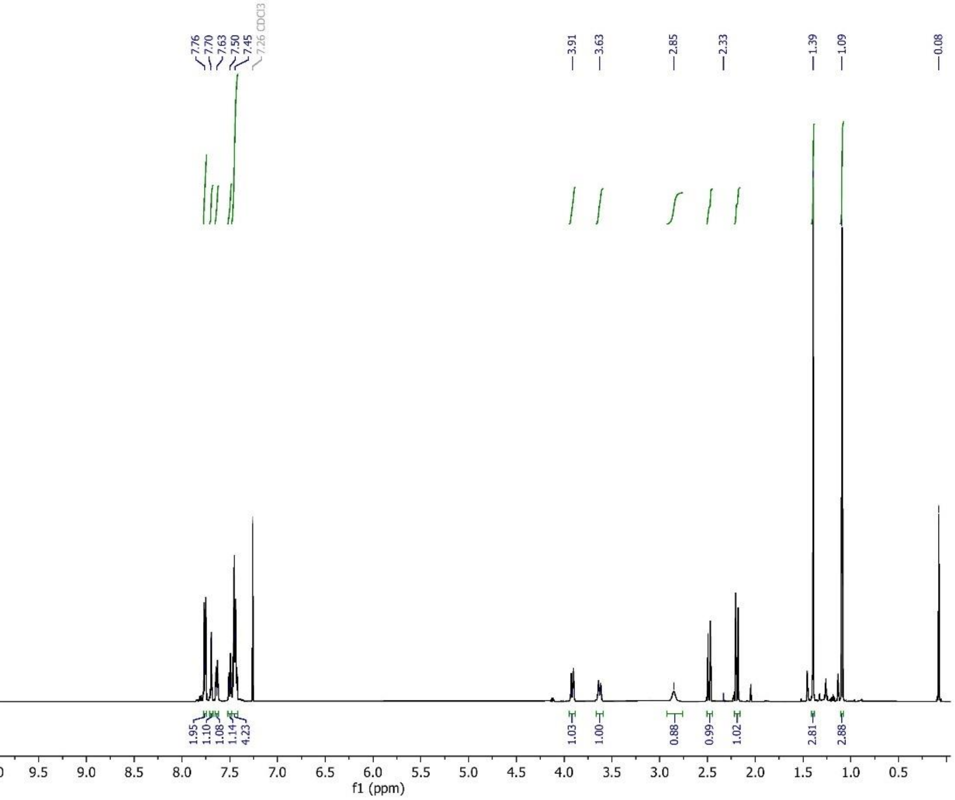

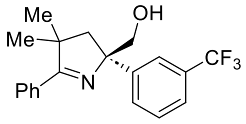

3g
^{13}C NMR
126 MHz, CDCl$_3$

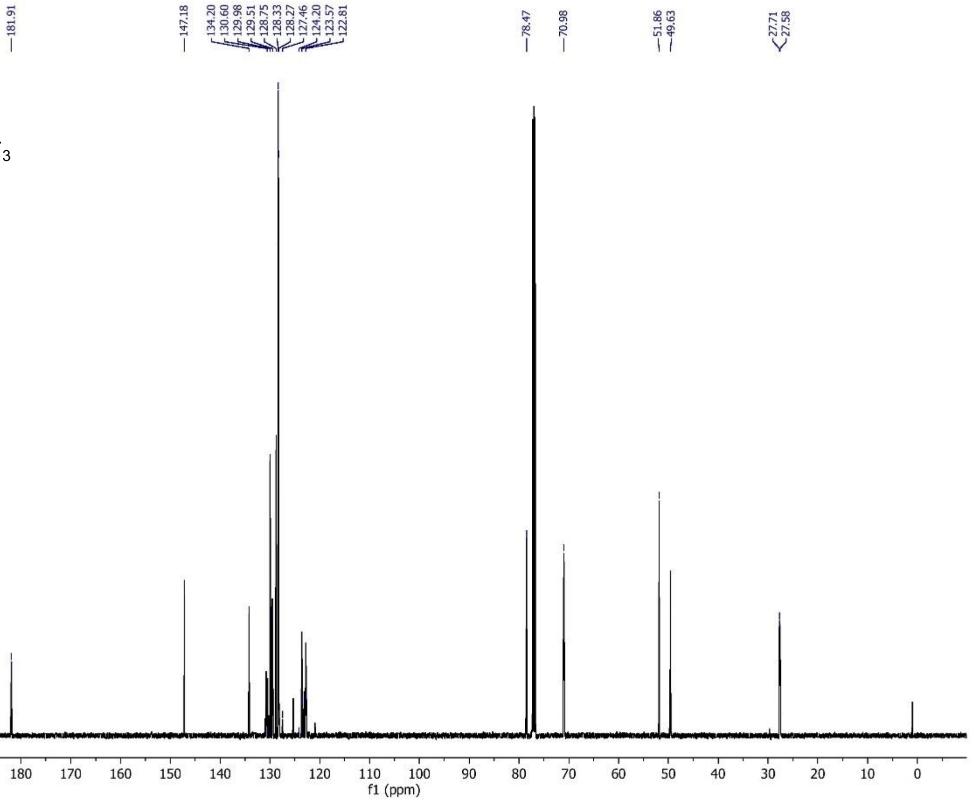

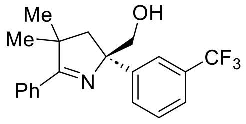

3g
^{19}F NMR
376 MHz, $CDCl_3$

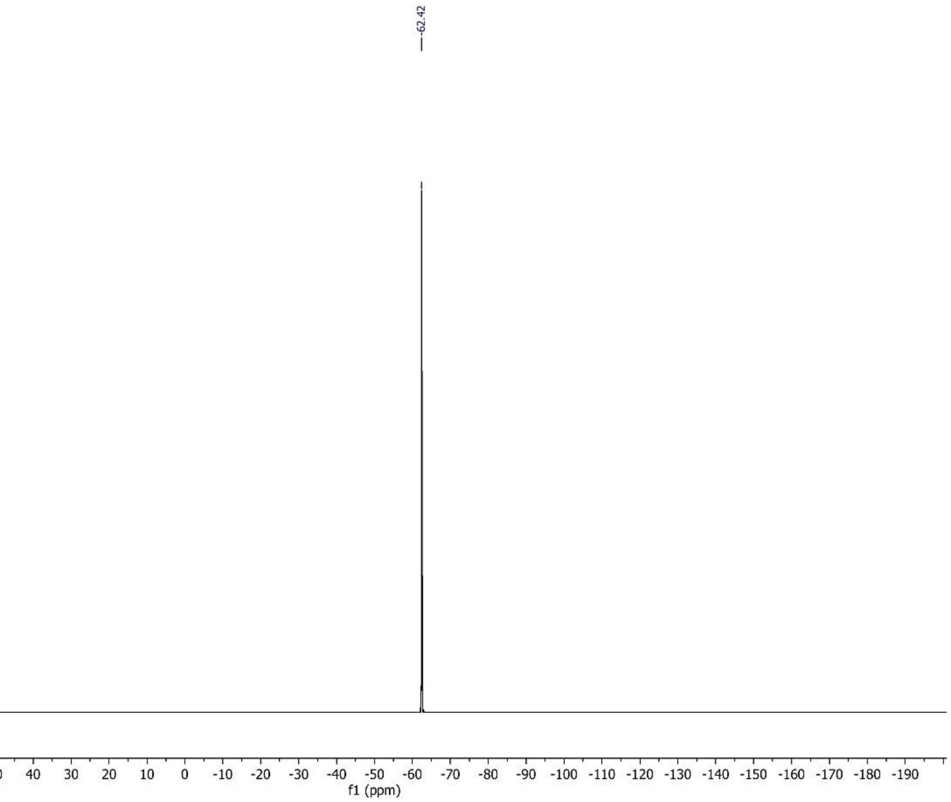

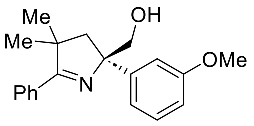

3h
^{1}H NMR
500 MHz, $CDCl_3$

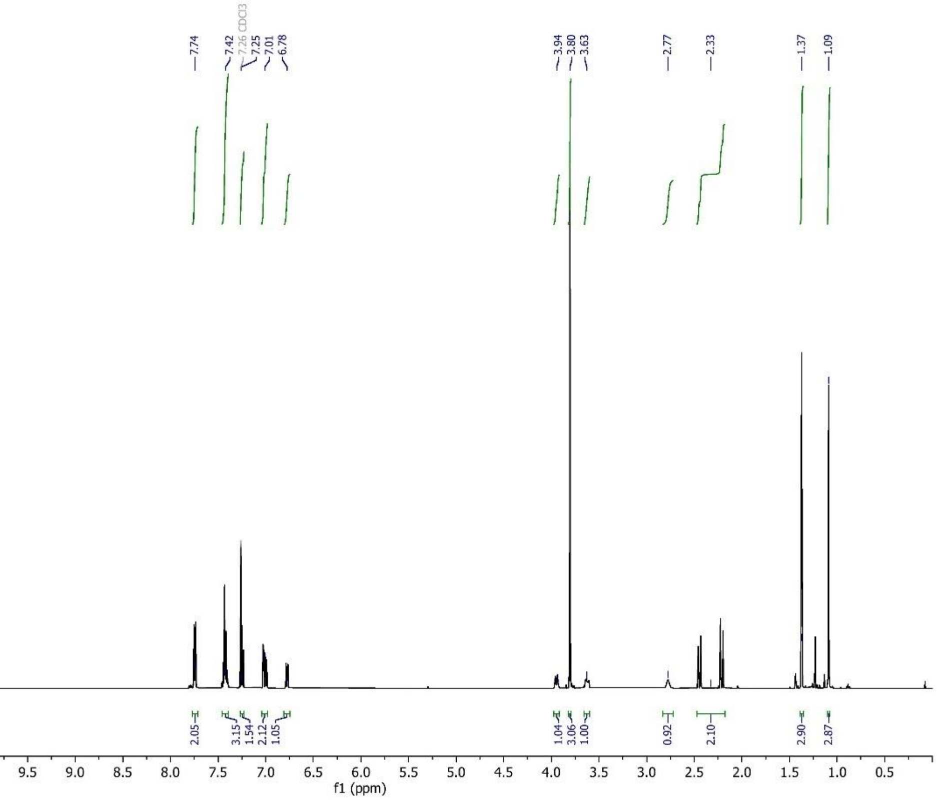

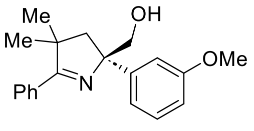

3h
^{13}C NMR
126 MHz, $CDCl_3$

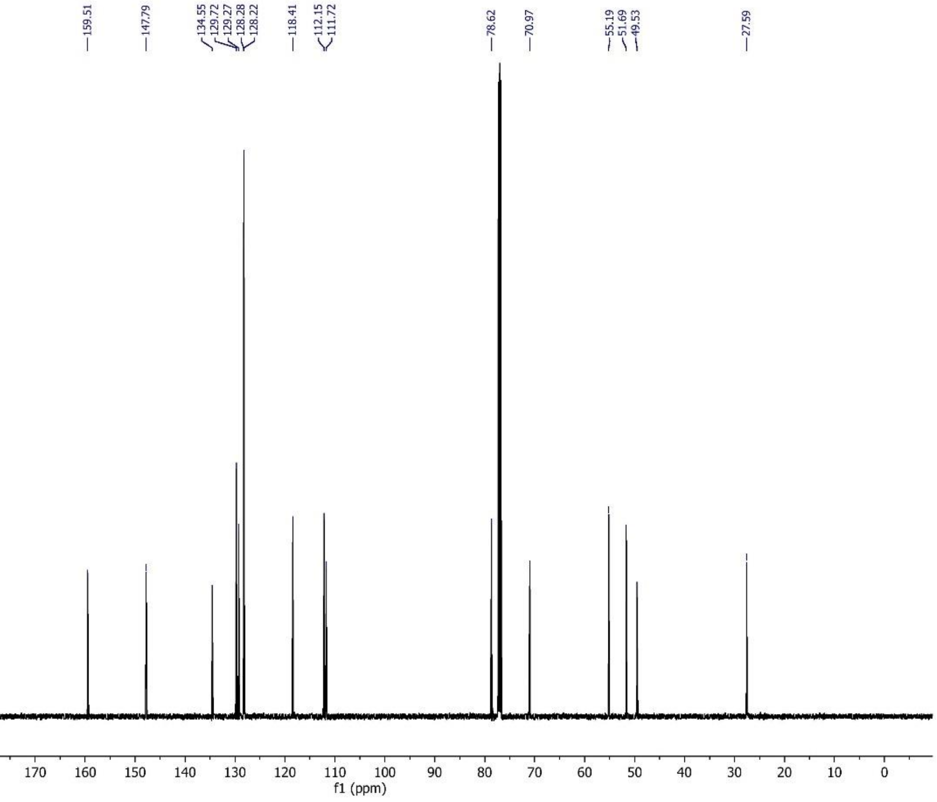

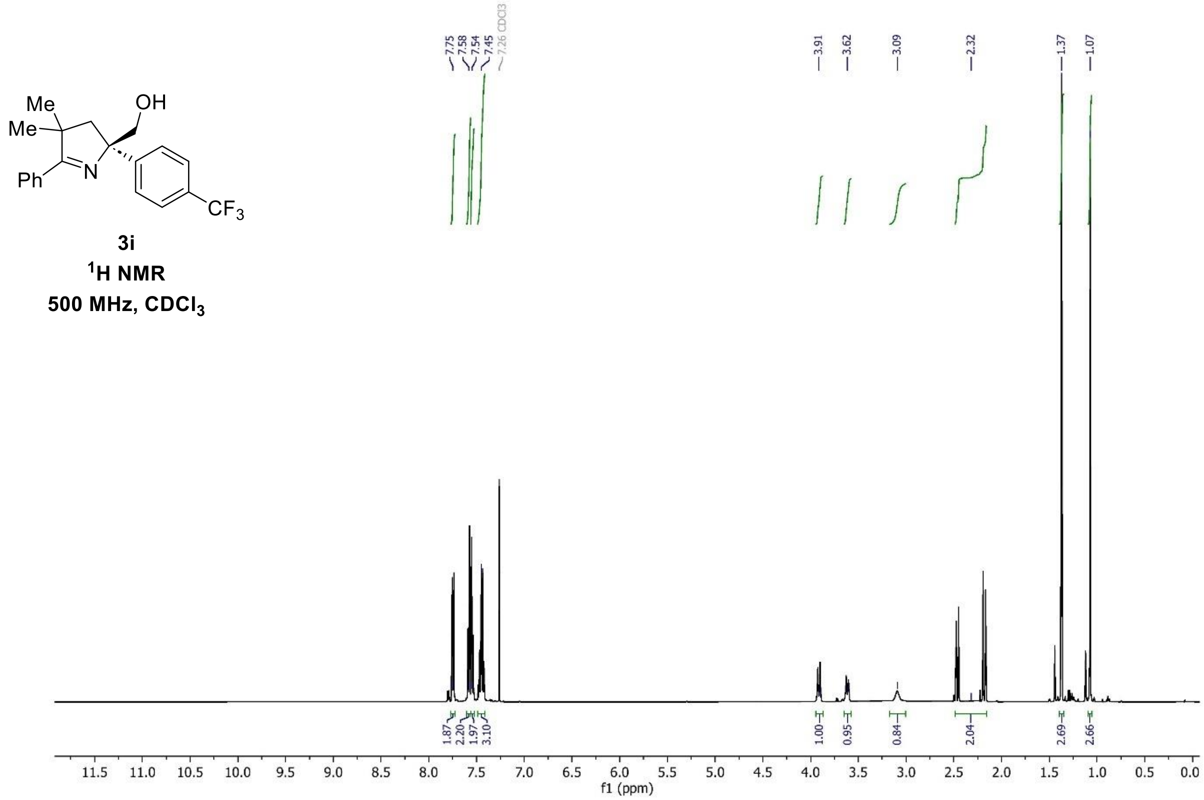

3i
^{1}H NMR
500 MHz, $CDCl_3$

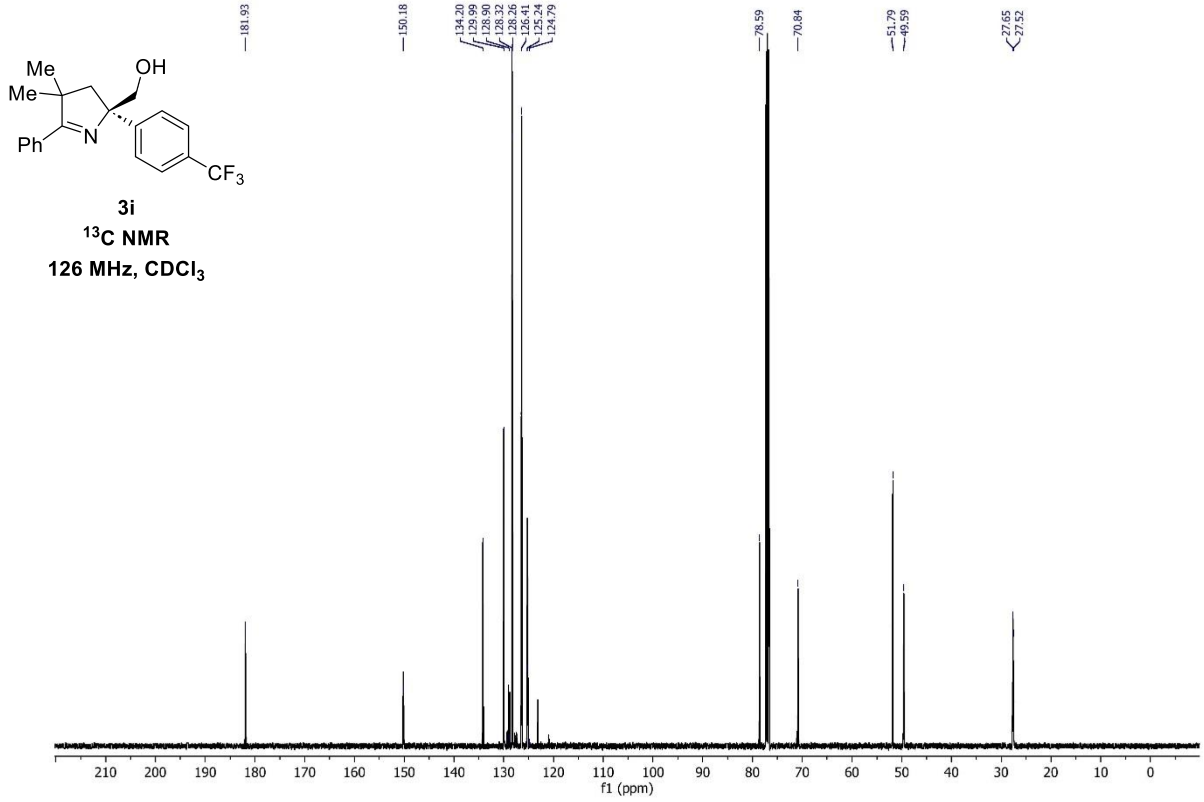

3i
^{13}C NMR
126 MHz, $CDCl_3$

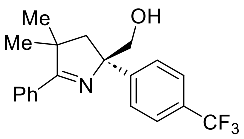

3i

^{19}F NMR

376 MHz, $CDCl_3$

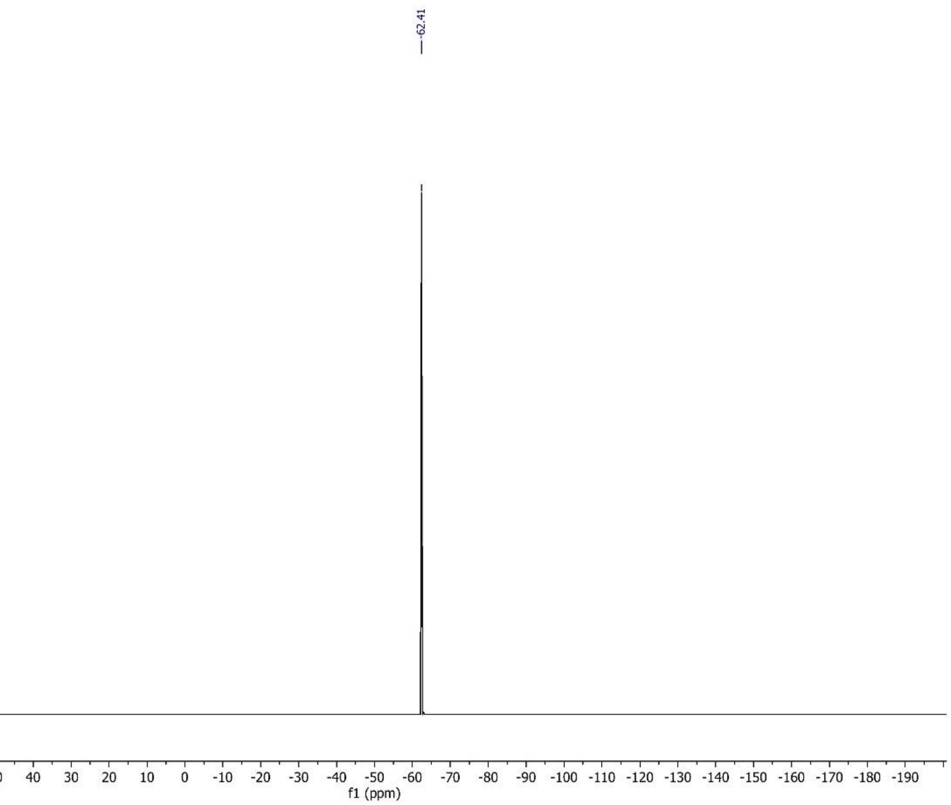

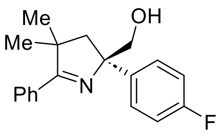

3j
^{1}H NMR
500 MHz, $CDCl_3$

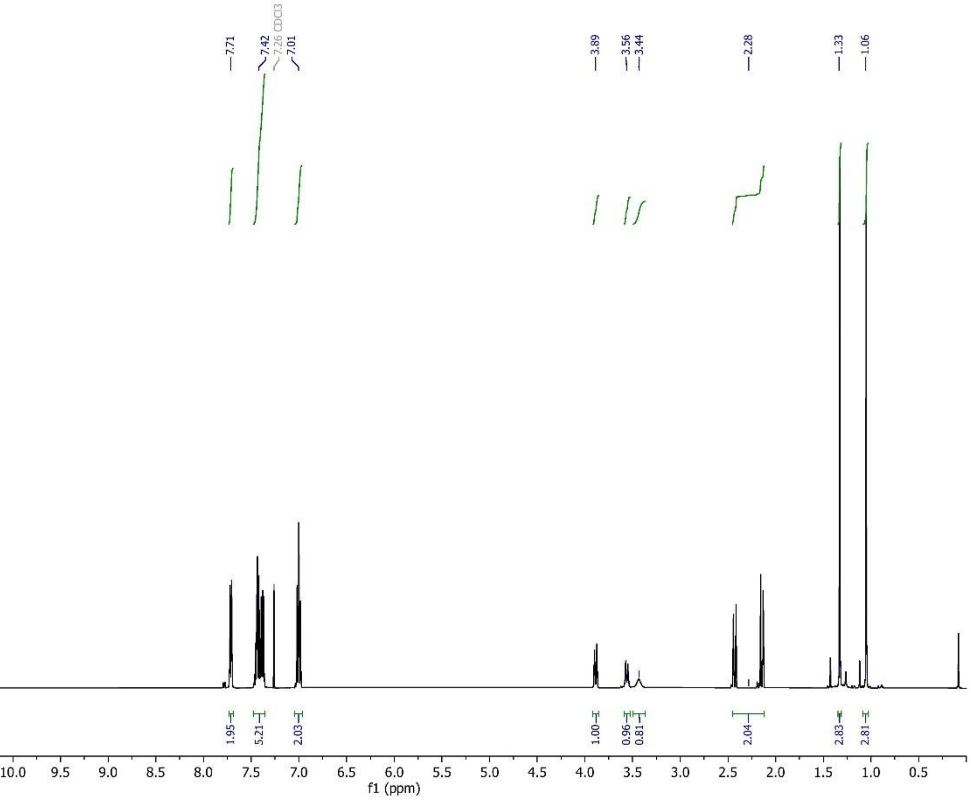

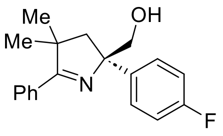

3j
^{13}C NMR
126 MHz, $CDCl_3$

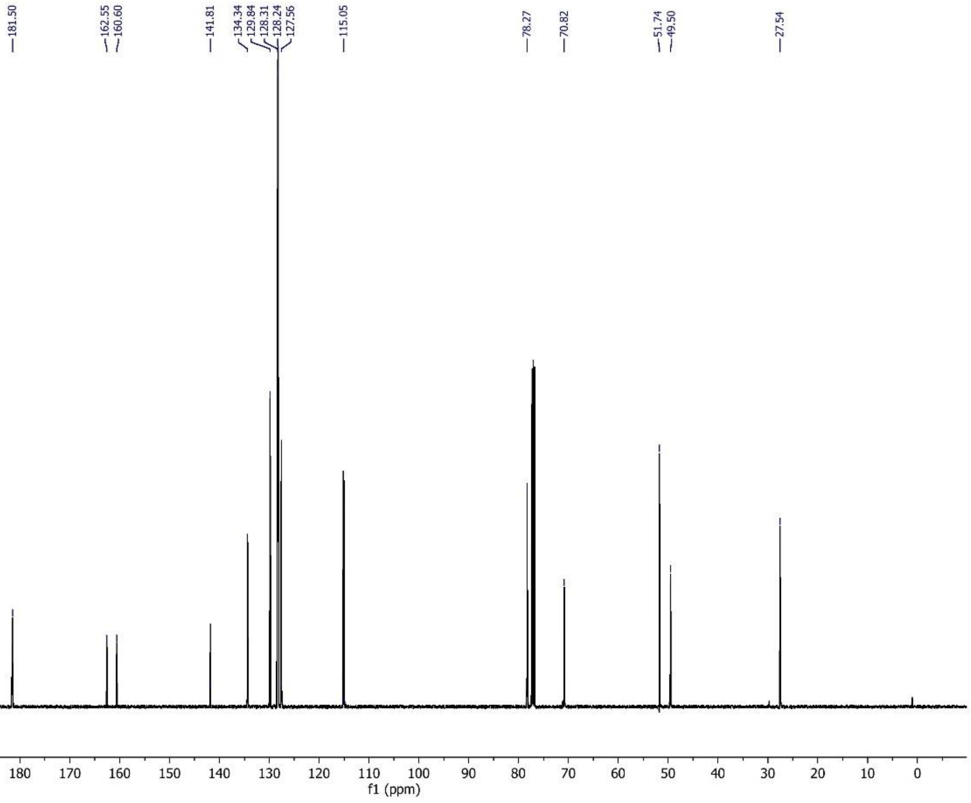

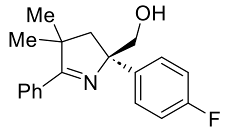

3j
^{19}F NMR
376 MHz, $CDCl_3$

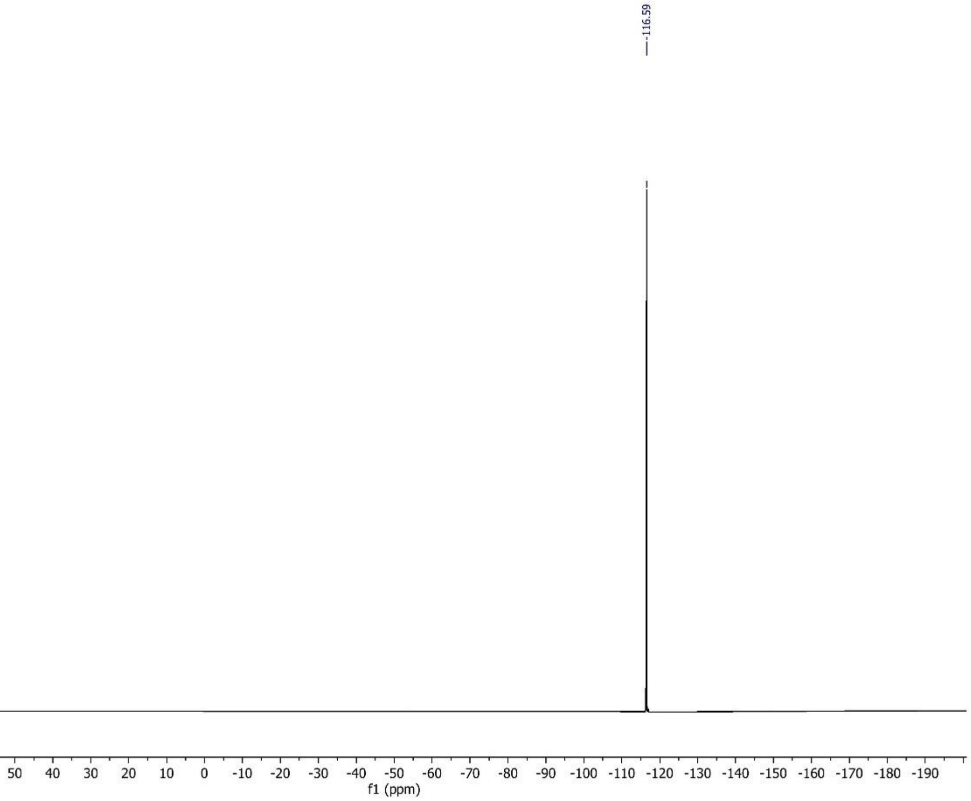

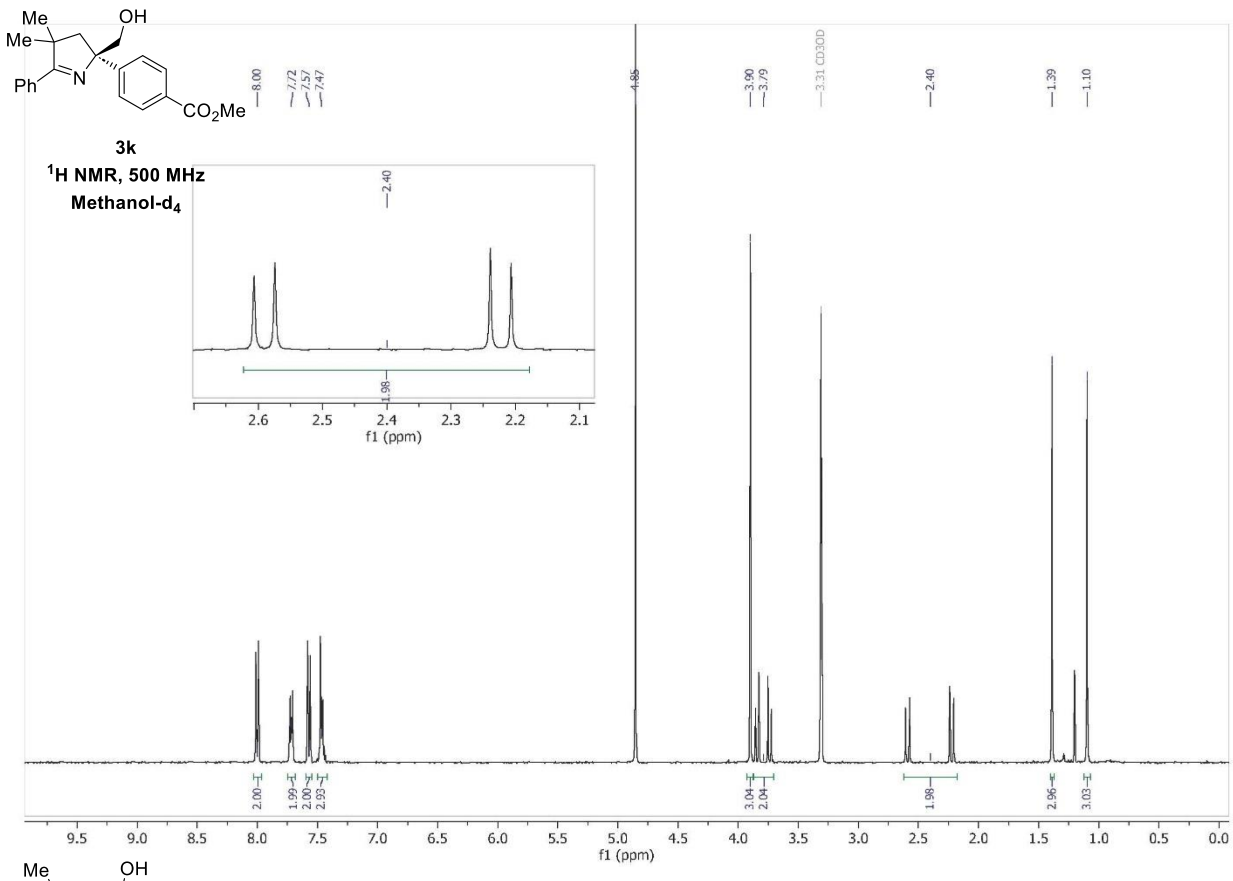
3k
^{1}H NMR, 500 MHz
Methanol-d$_4$

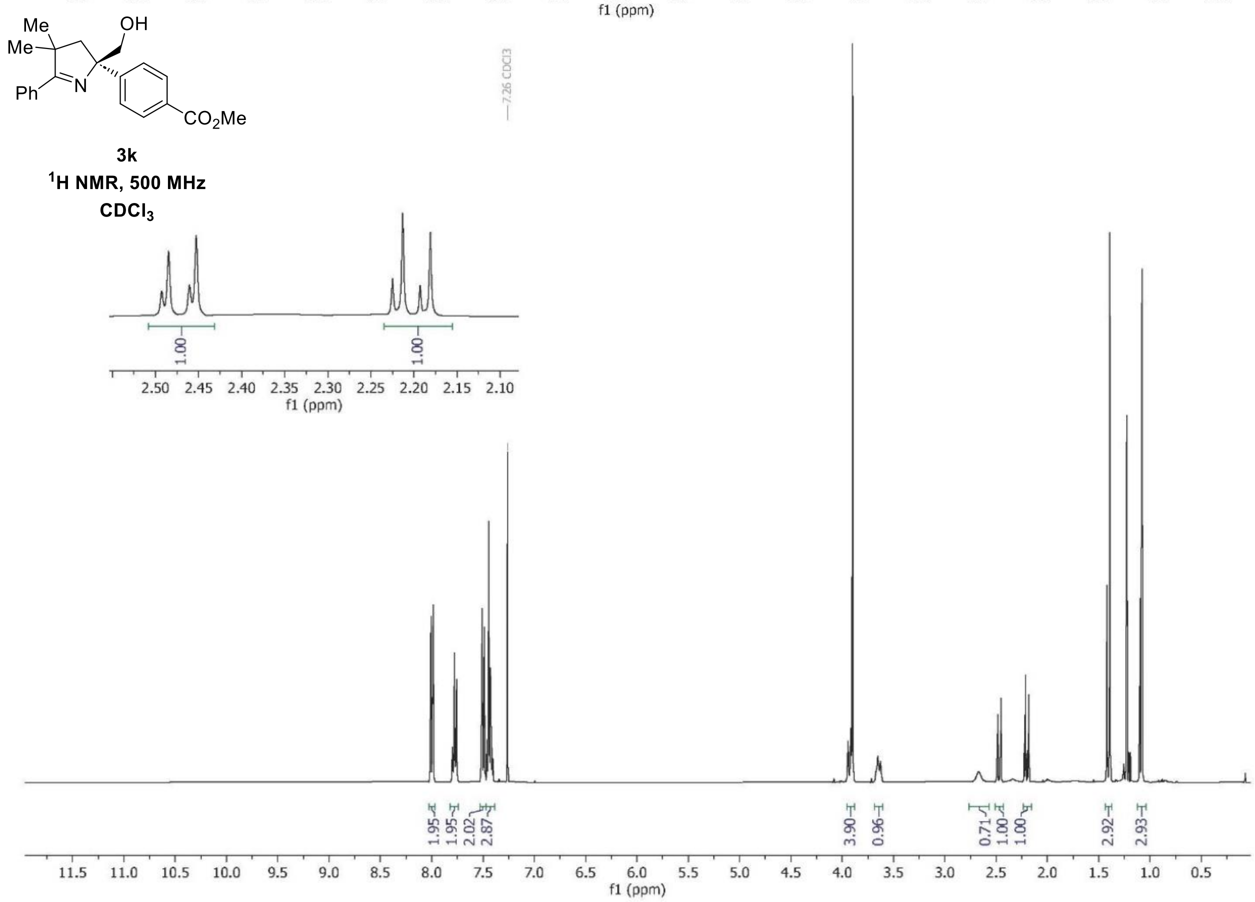
3k
^{1}H NMR, 500 MHz
CDCl$_3$

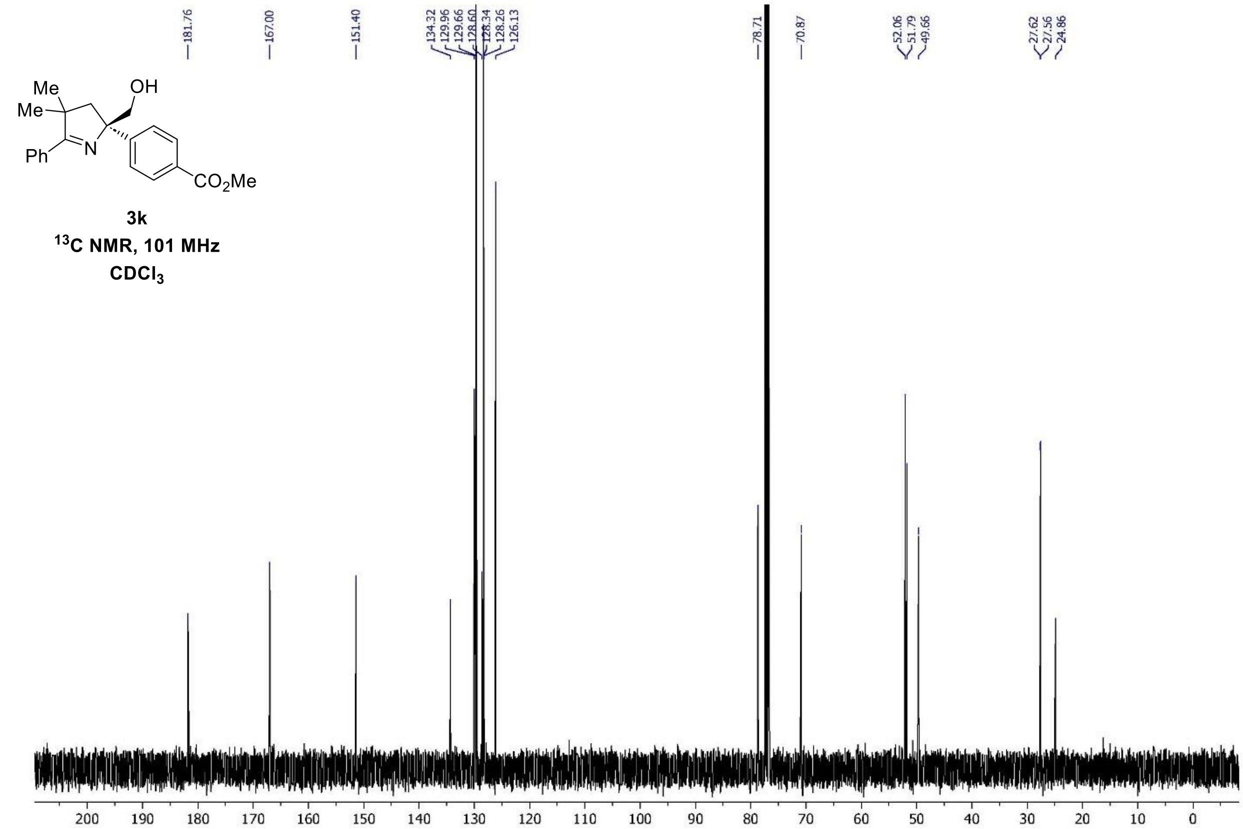

3k

^{13}C NMR, 101 MHz

$CDCl_3$

3k DOSY Transform, $CDCl_3$, 400 MHz

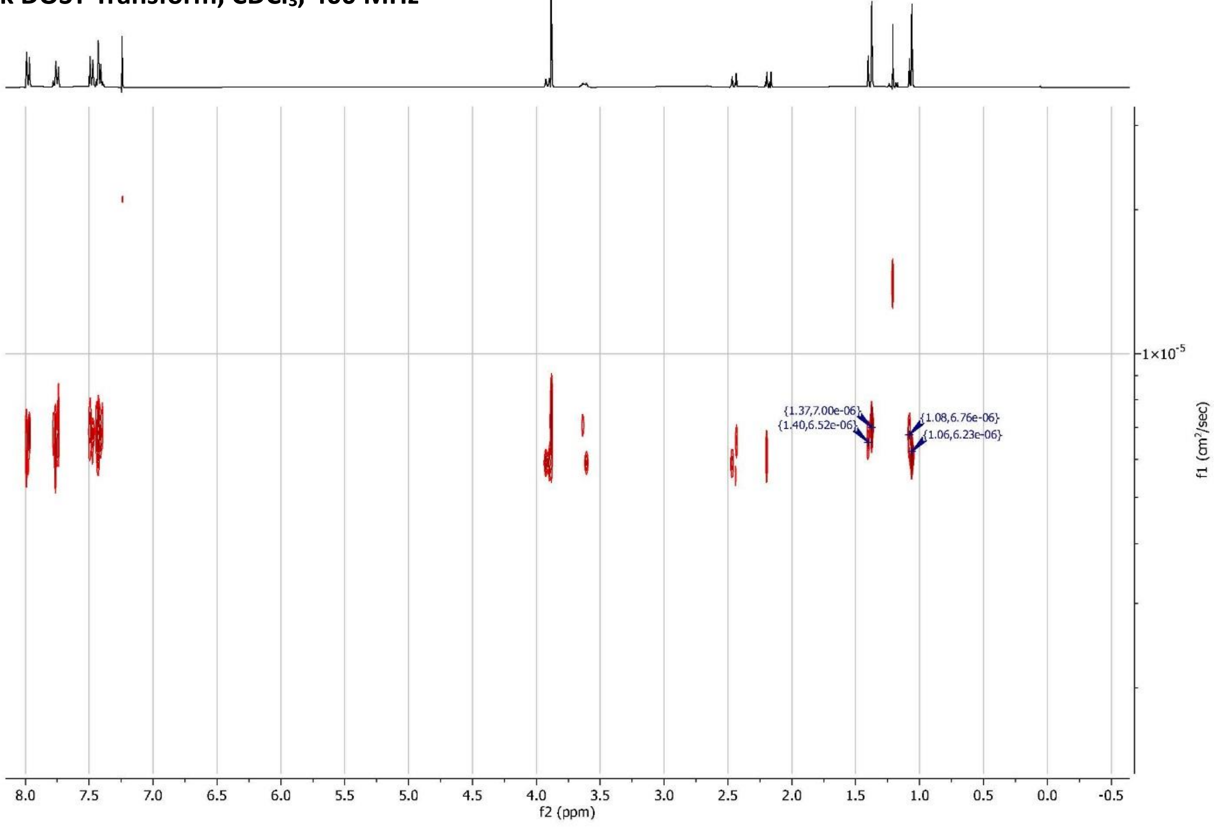

3k DOSY Transform, $CDCl_3$, 400 MHz (Zoomed)

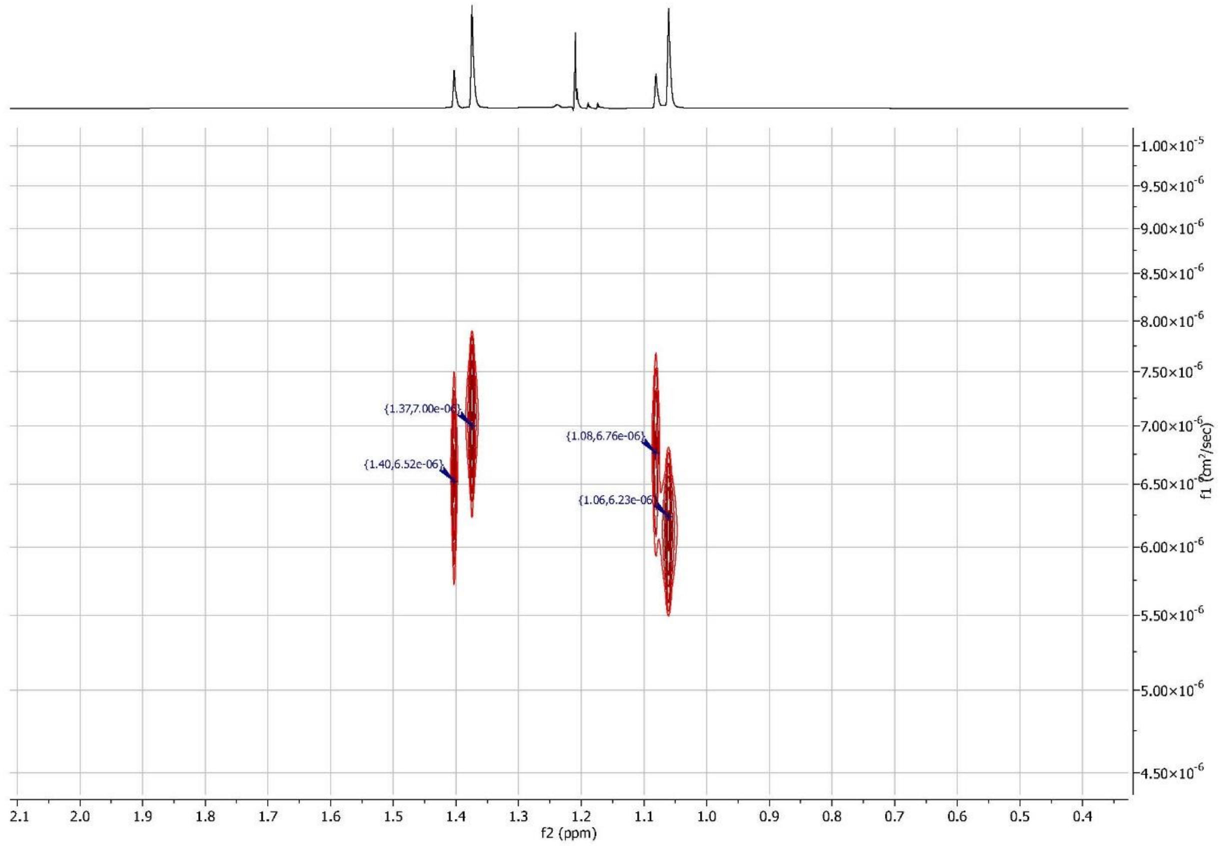

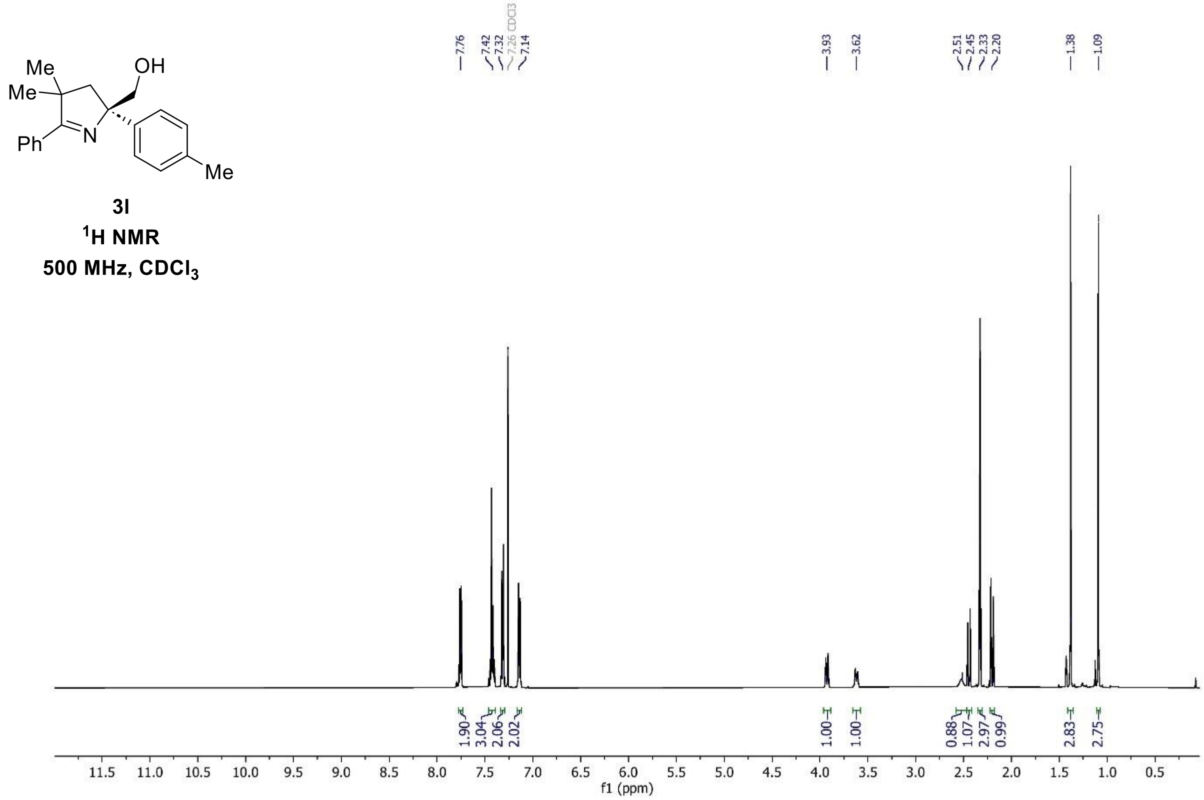

3l
^{1}H NMR
500 MHz, $CDCl_3$

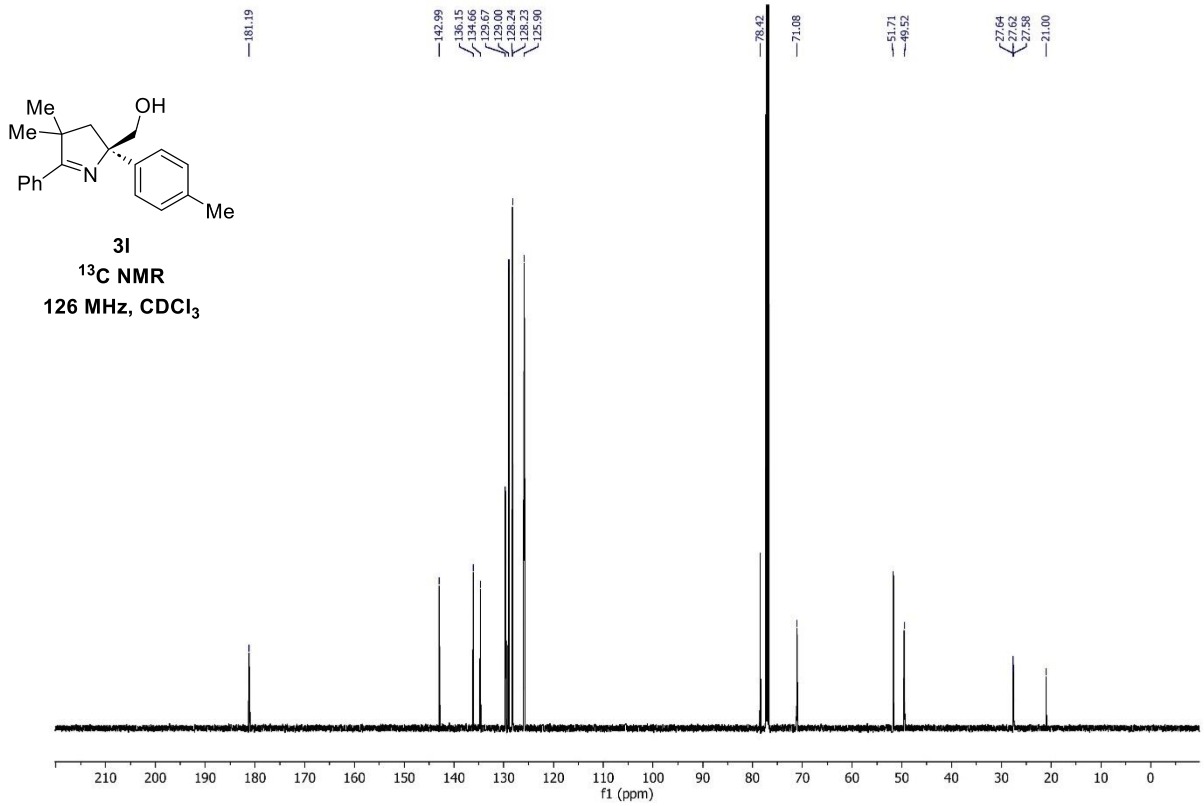

3l
^{13}C NMR
126 MHz, $CDCl_3$

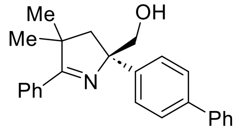

3m
^{1}H NMR
500 MHz, $CDCl_3$

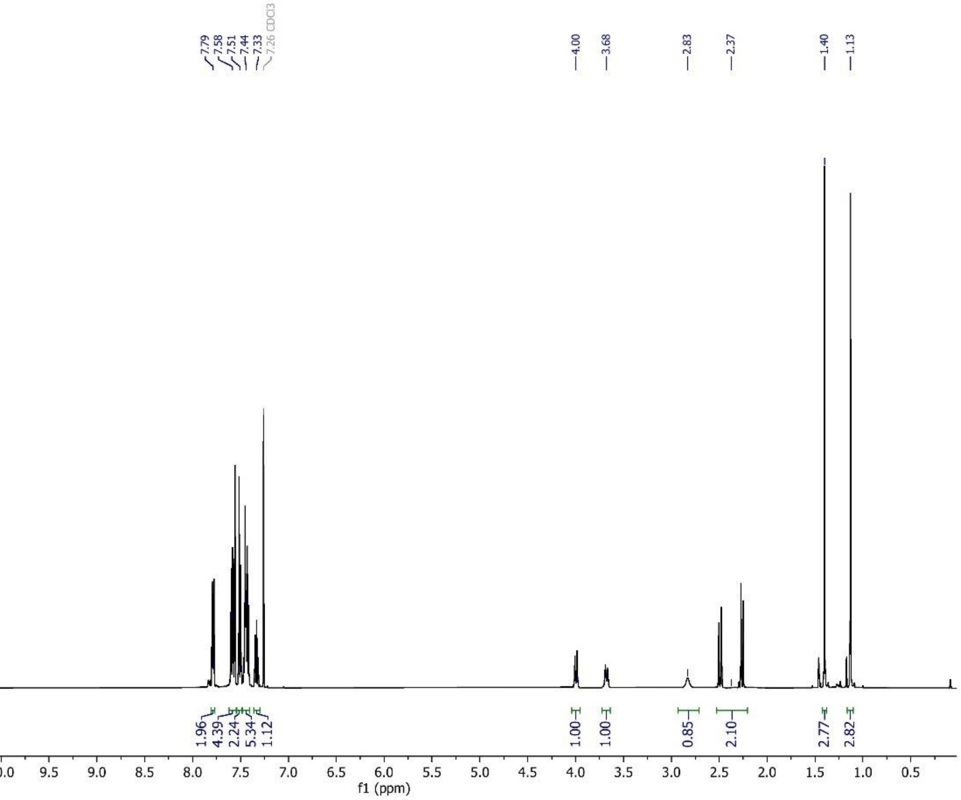

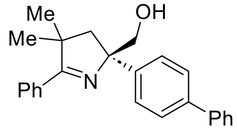

3m
^{13}C NMR
126 MHz, $CDCl_3$

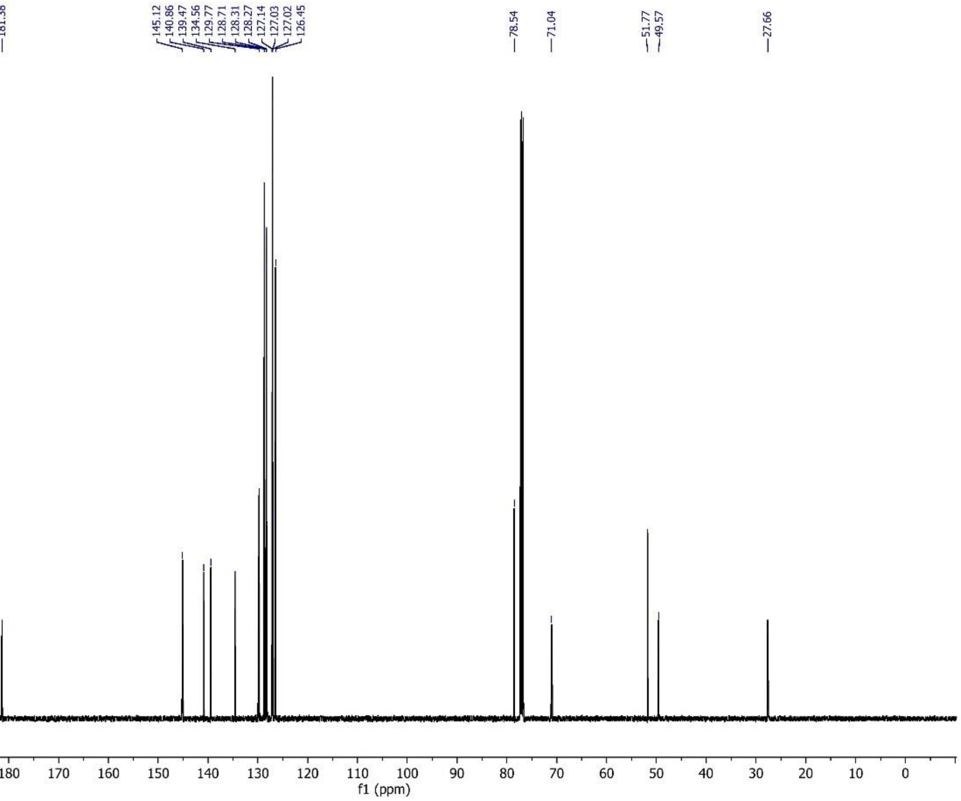

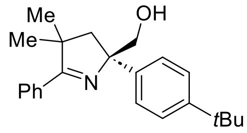

3n
^{1}H NMR
500 MHz, $CDCl_3$

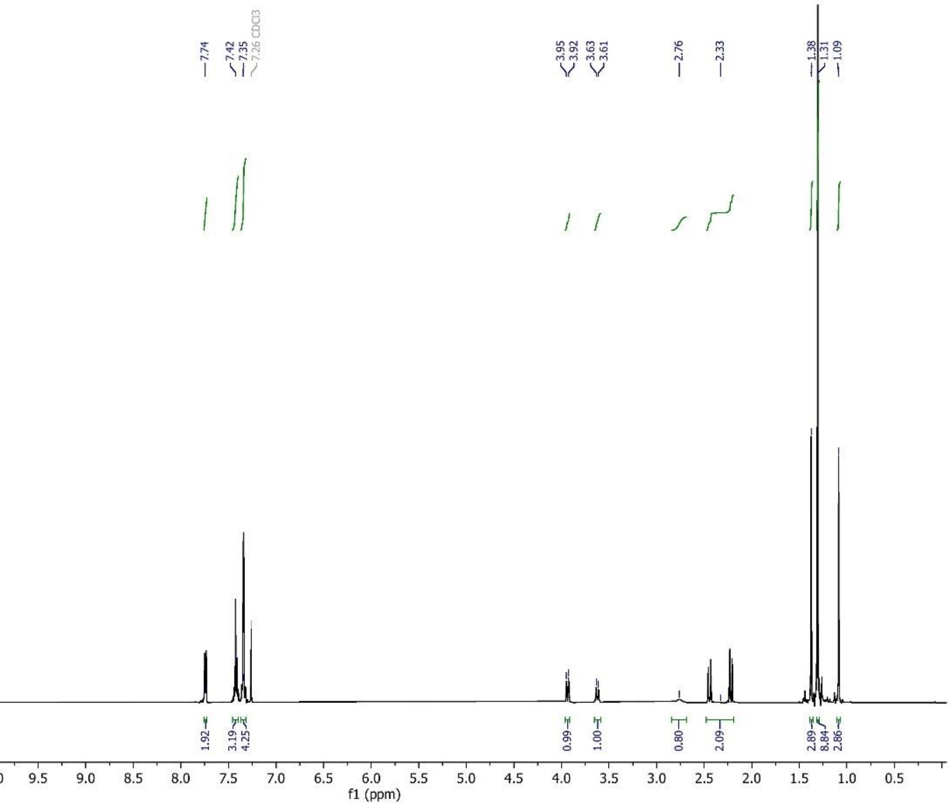

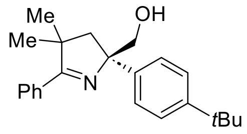

3n
^{13}C NMR
126 MHz, $CDCl_3$

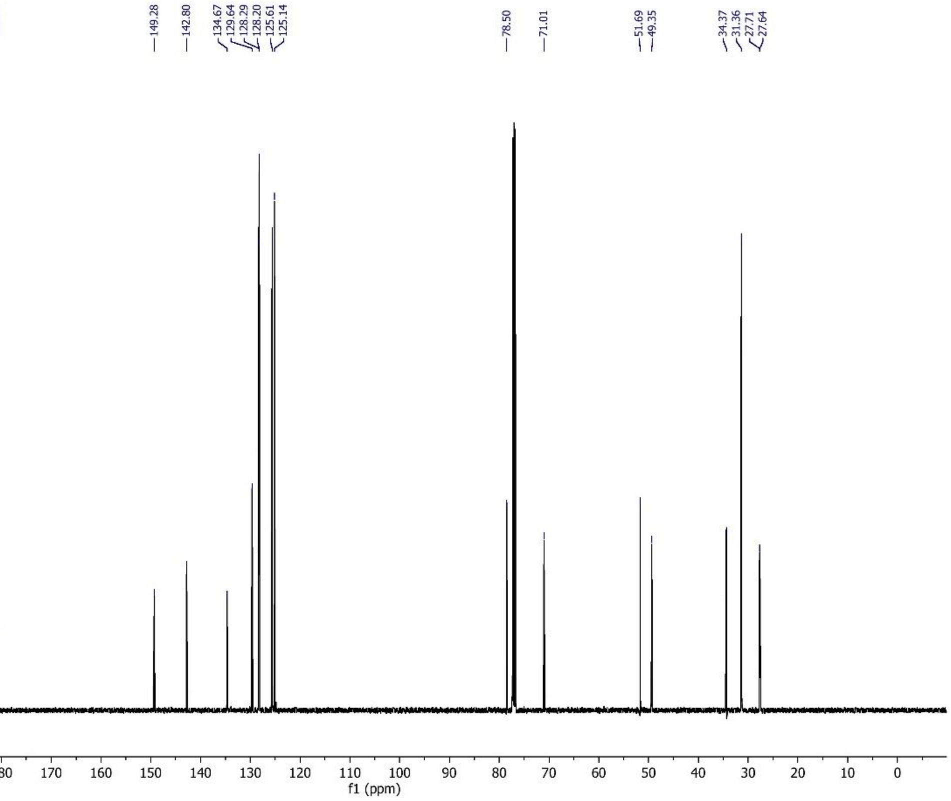

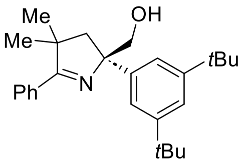

3o, ^{1}H NMR, 500 MHz, CDCl$_3$

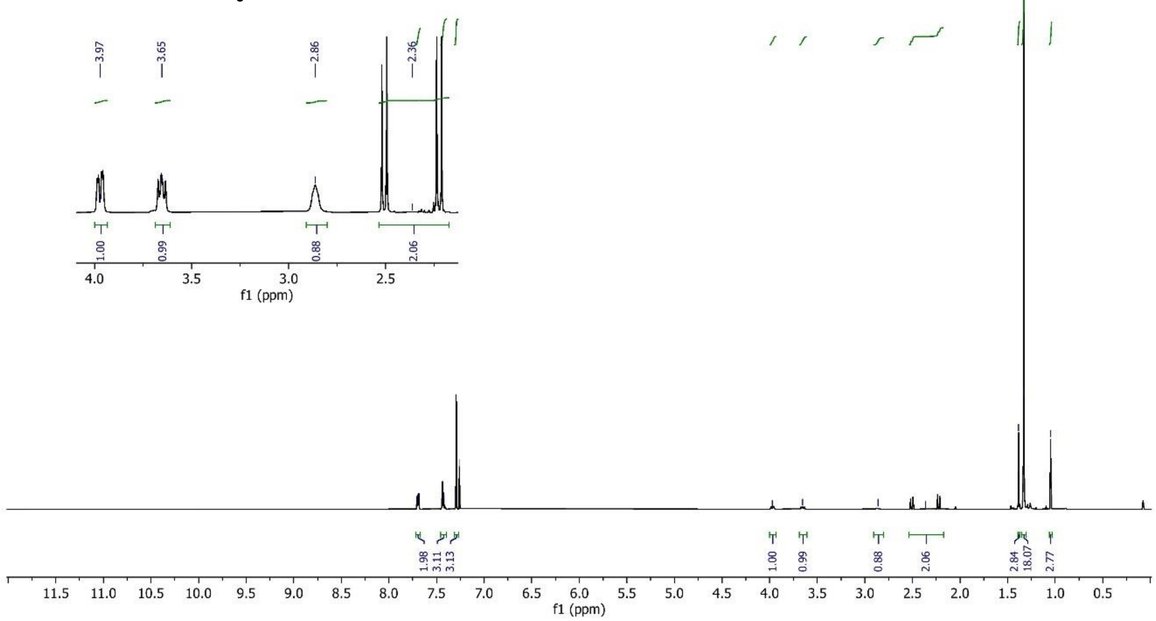

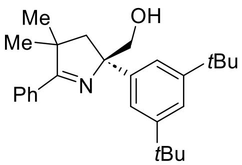

3o
^{13}C NMR
126 MHz, CDCl$_3$

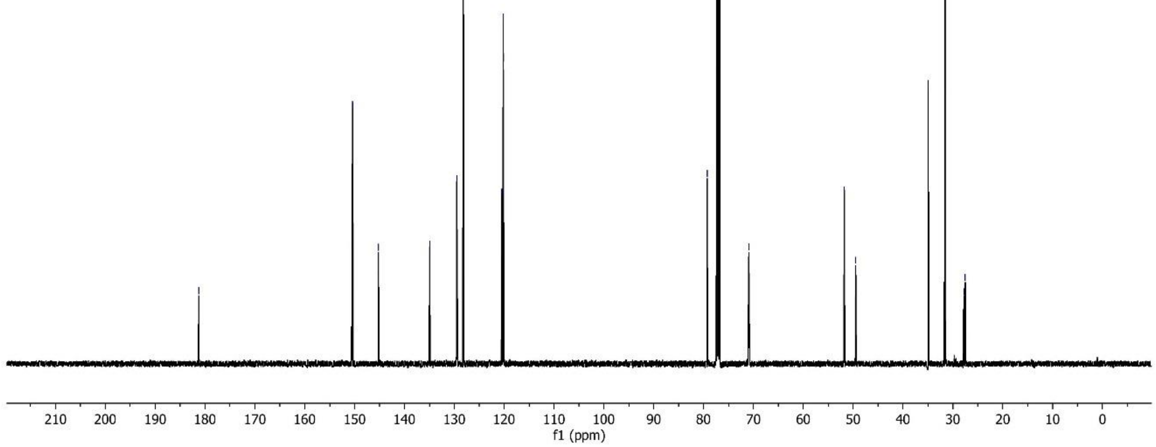

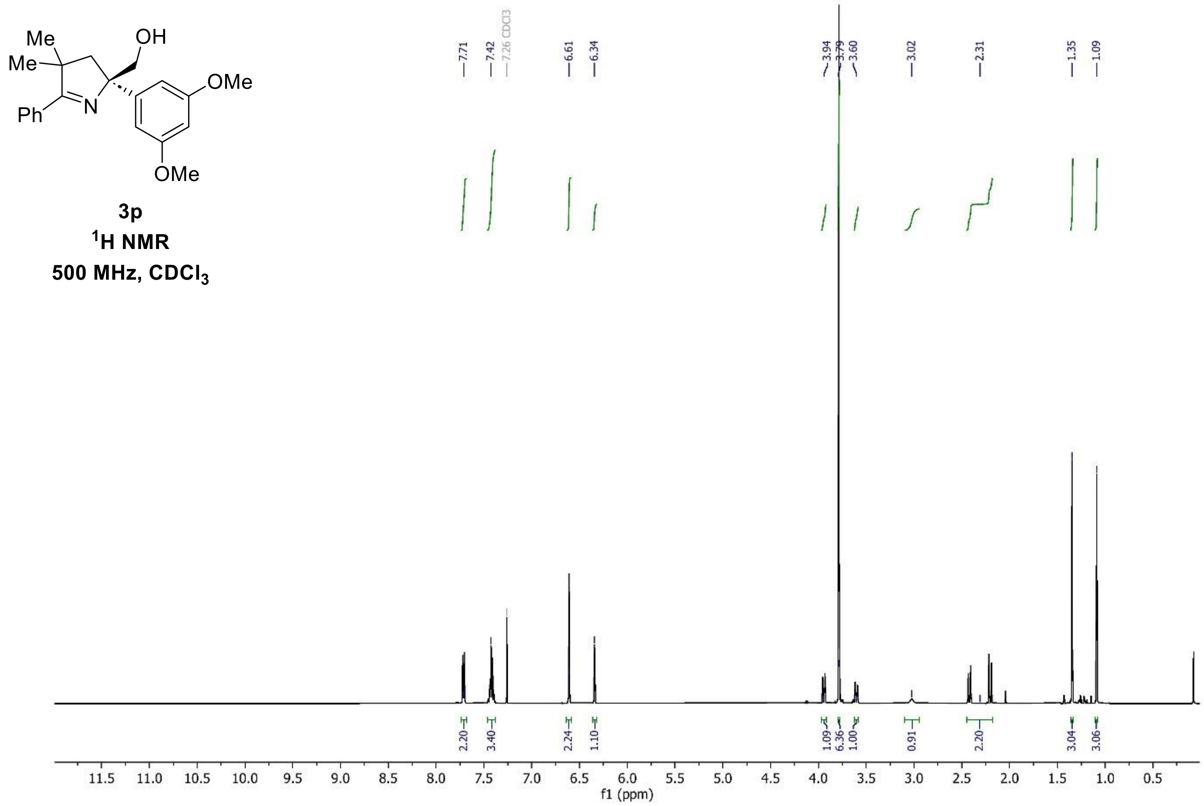
Me
Me
OH
OMe
Ph
N
OMe
3p
1H NMR
500 MHz, CDCl3
7.71
7.42
7.26 CDCl3
6.61
6.34
3.94
3.79
3.60
3.02
2.31
1.35
1.09
2.20
3.40
2.24
1.10
1.09
6.36
1.00
0.91
2.20
3.04
3.06
f1 (ppm)

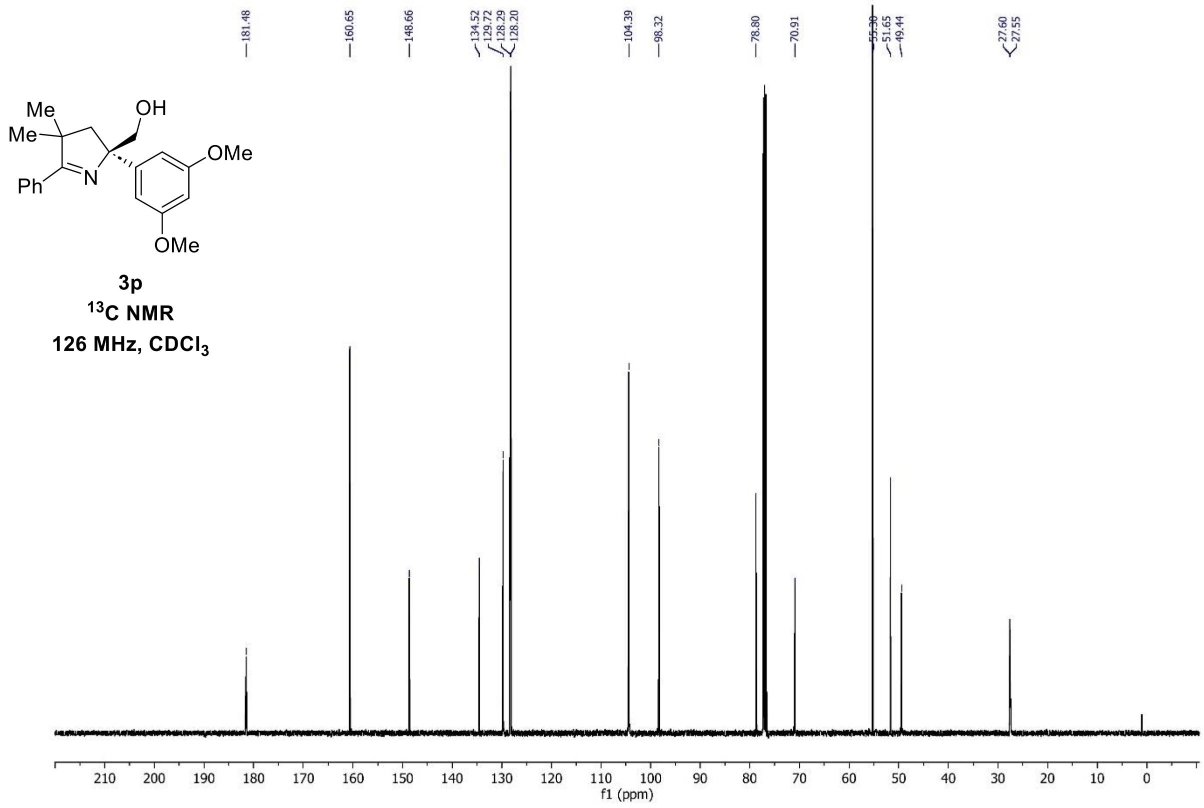
181.48
160.65
148.66
134.52
129.72
128.29
128.20
104.39
98.32
78.80
70.91
55.30
51.65
49.44
27.60
27.55
Me
Me
OH
OMe
Ph
N
OMe
3p
13C NMR
126 MHz, CDCl3
f1 (ppm)

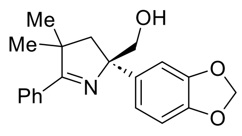

3q
^{1}H NMR
500 MHz, $CDCl_3$

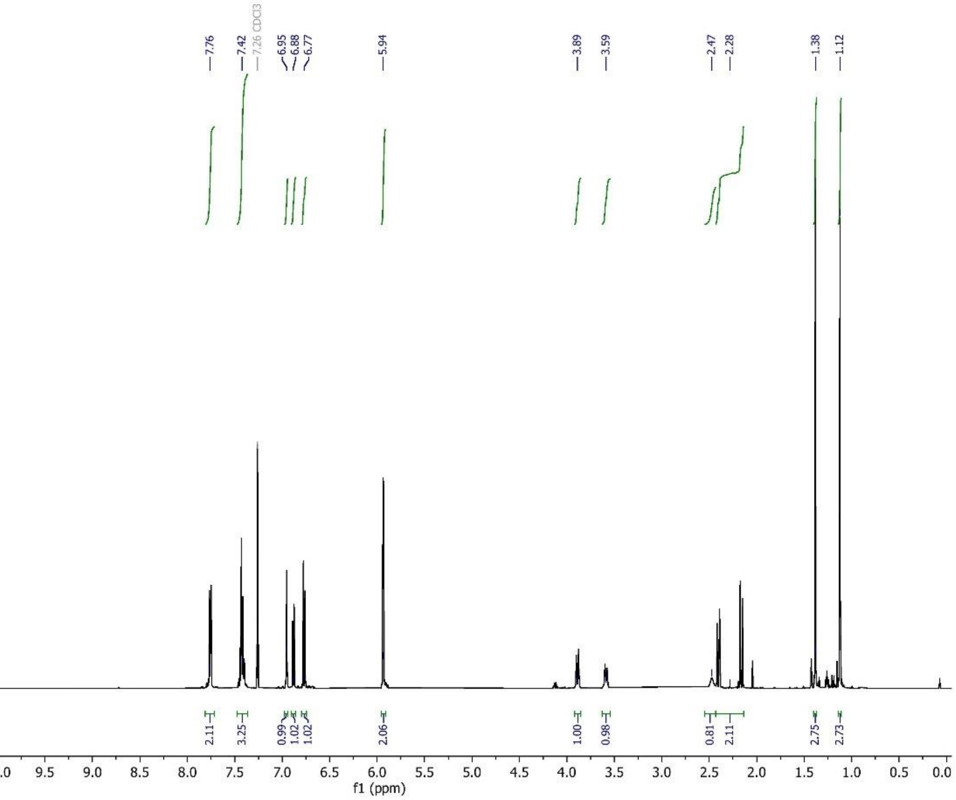

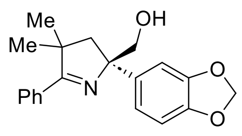

3q
^{13}C NMR
126 MHz, $CDCl_3$

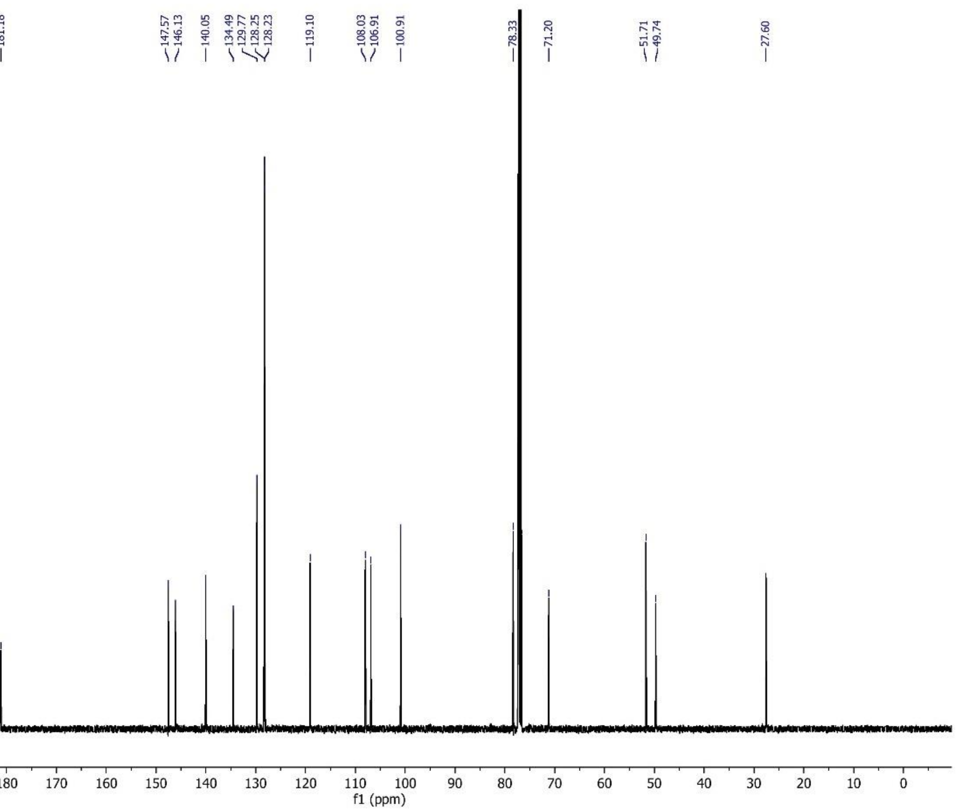

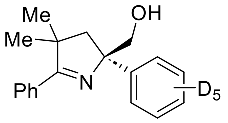

3r
^{1}H NMR
500 MHz, $CDCl_3$

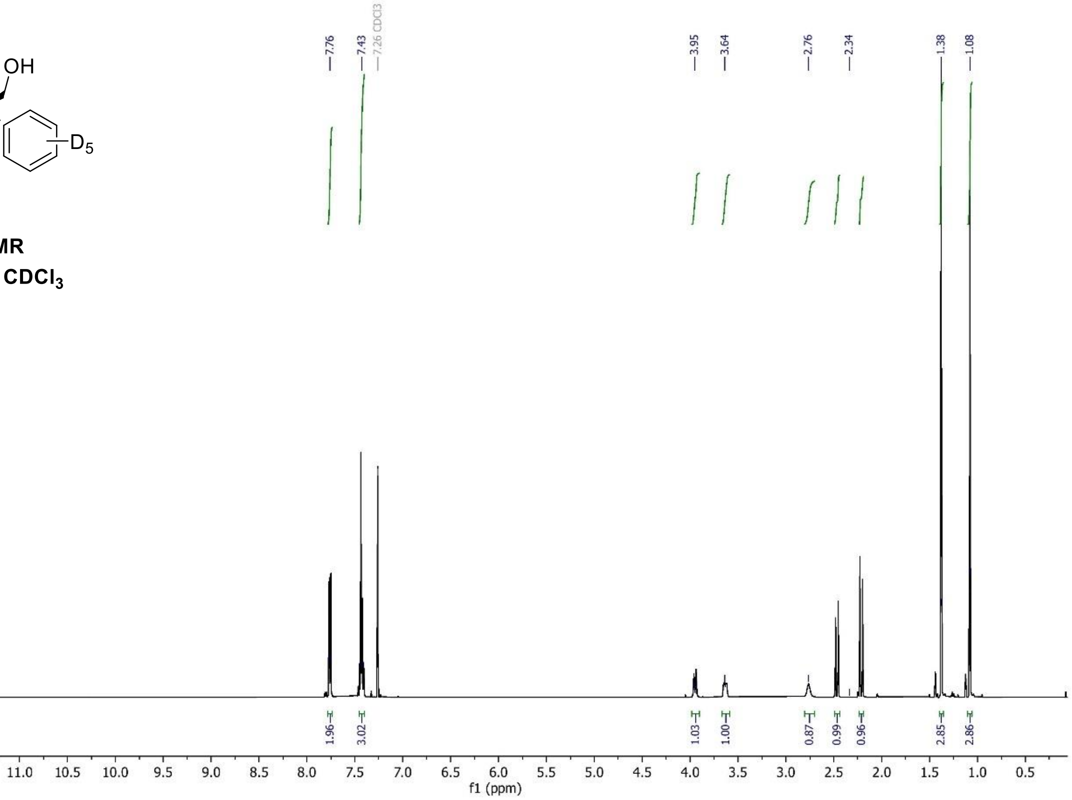

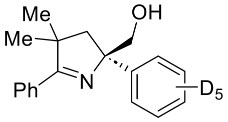

3r
^{13}C NMR
126 MHz, $CDCl_3$

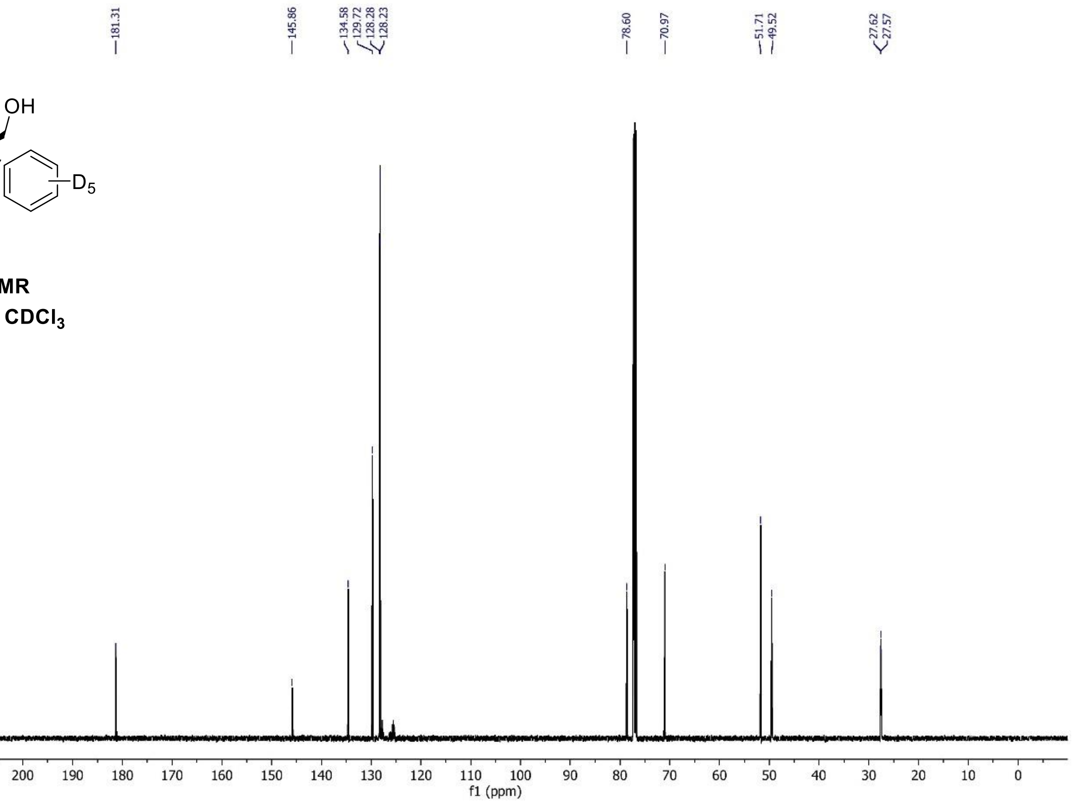

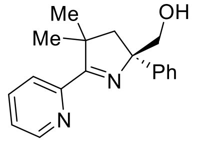

3s
^{1}H NMR
500 MHz, $CDCl_3$

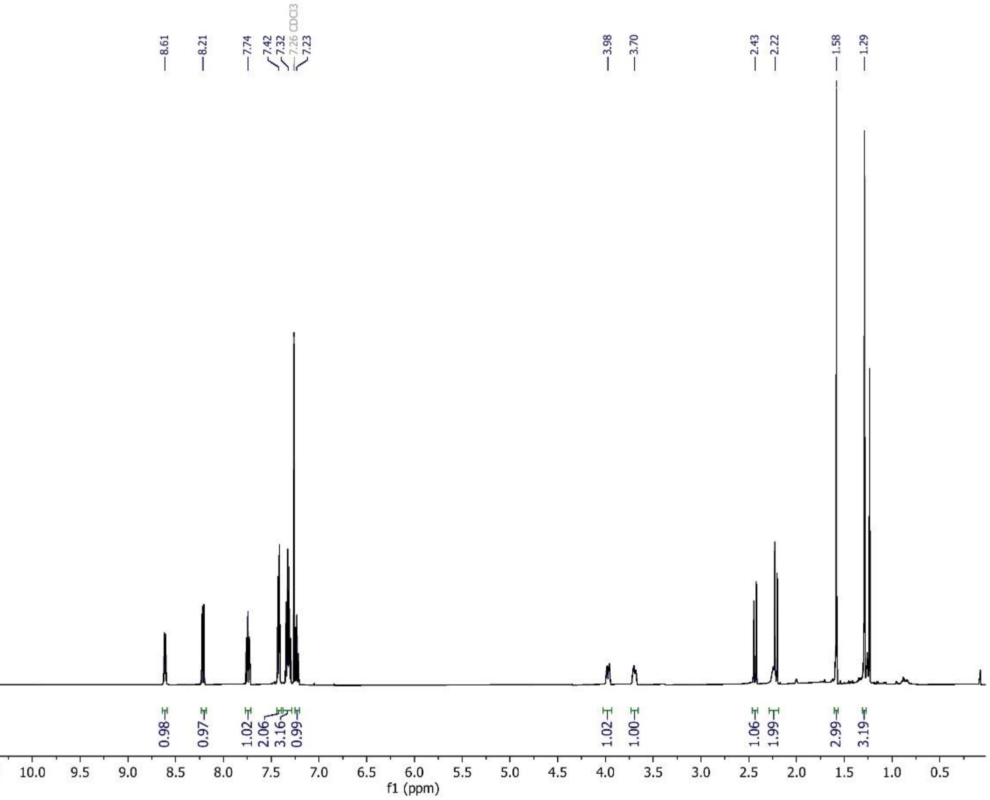

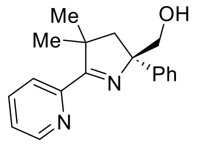

3s
^{13}C NMR
126 MHz, $CDCl_3$

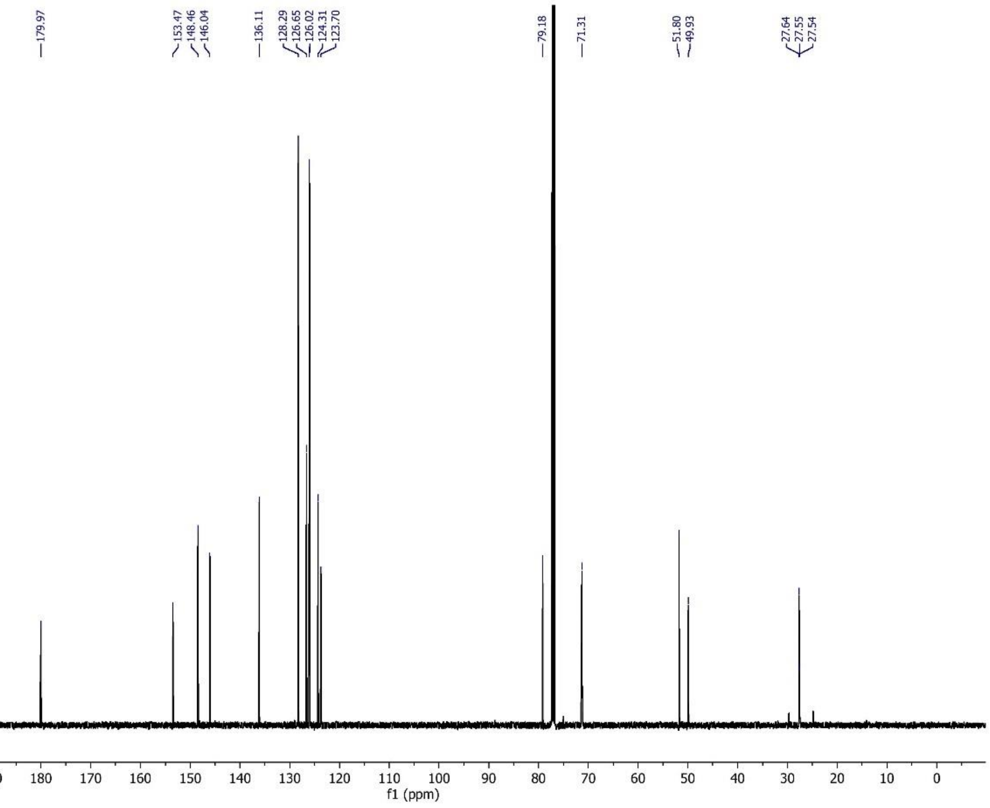

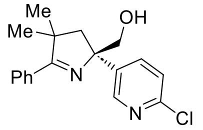

3t
^{1}H NMR, 500 MHz
$CDCl_3$

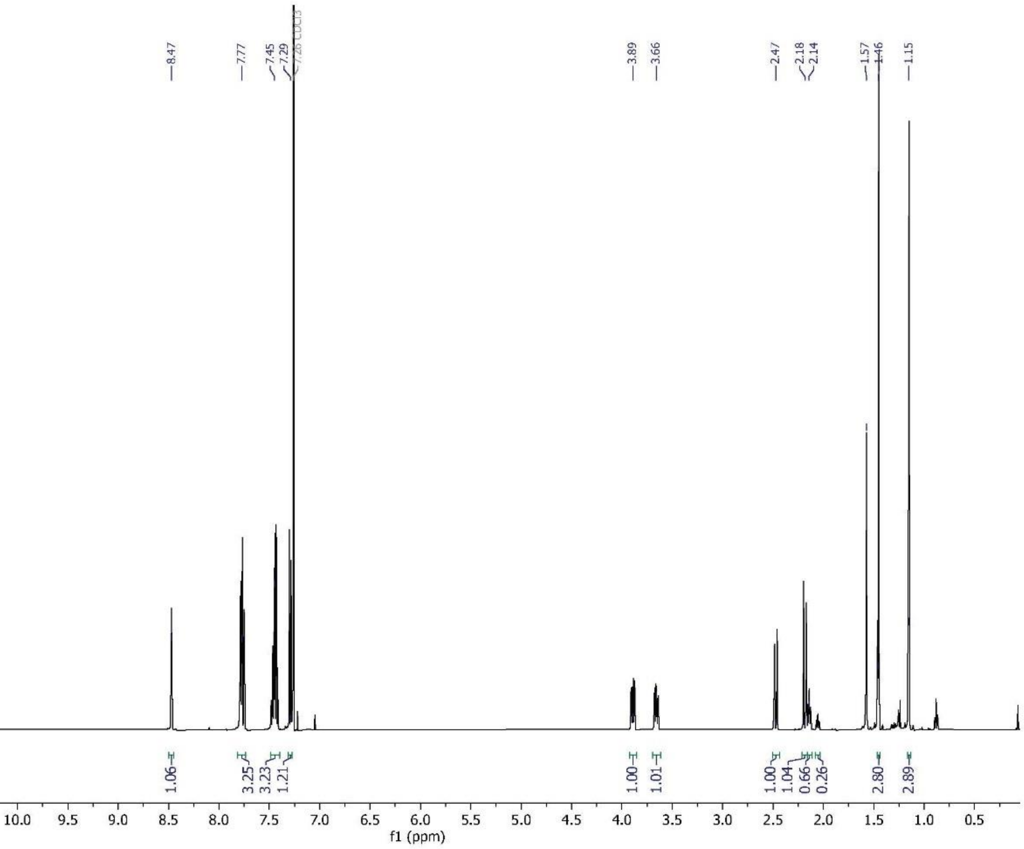

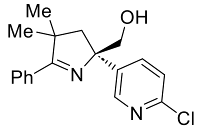

3t
^{13}C NMR, 126 MHz
$CDCl_3$

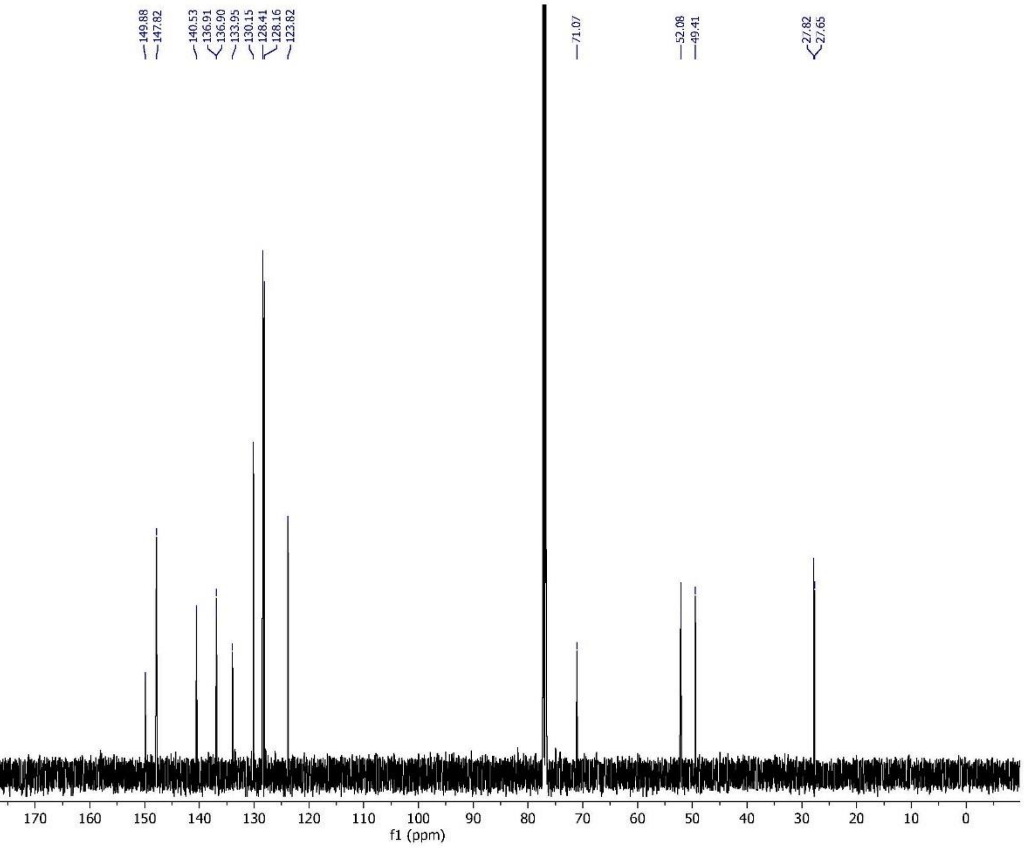

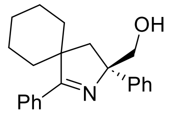

3u
^{1}H NMR
500 MHz, $CDCl_3$

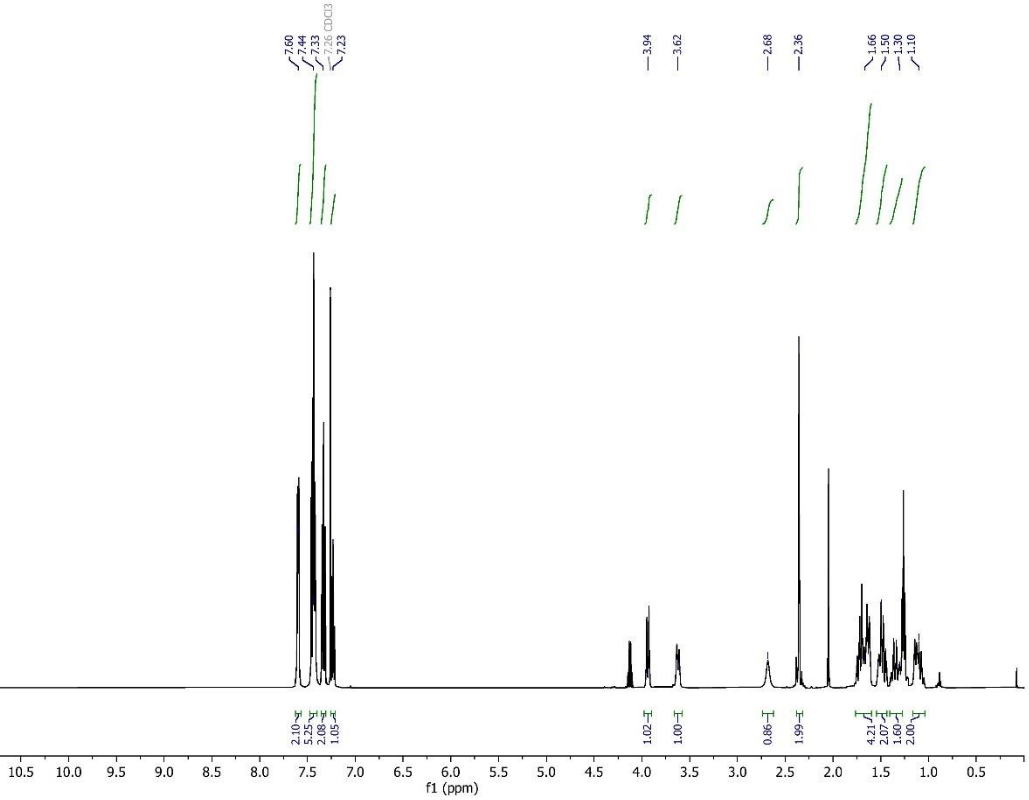

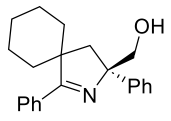

3u
^{13}C NMR
126 MHz, $CDCl_3$

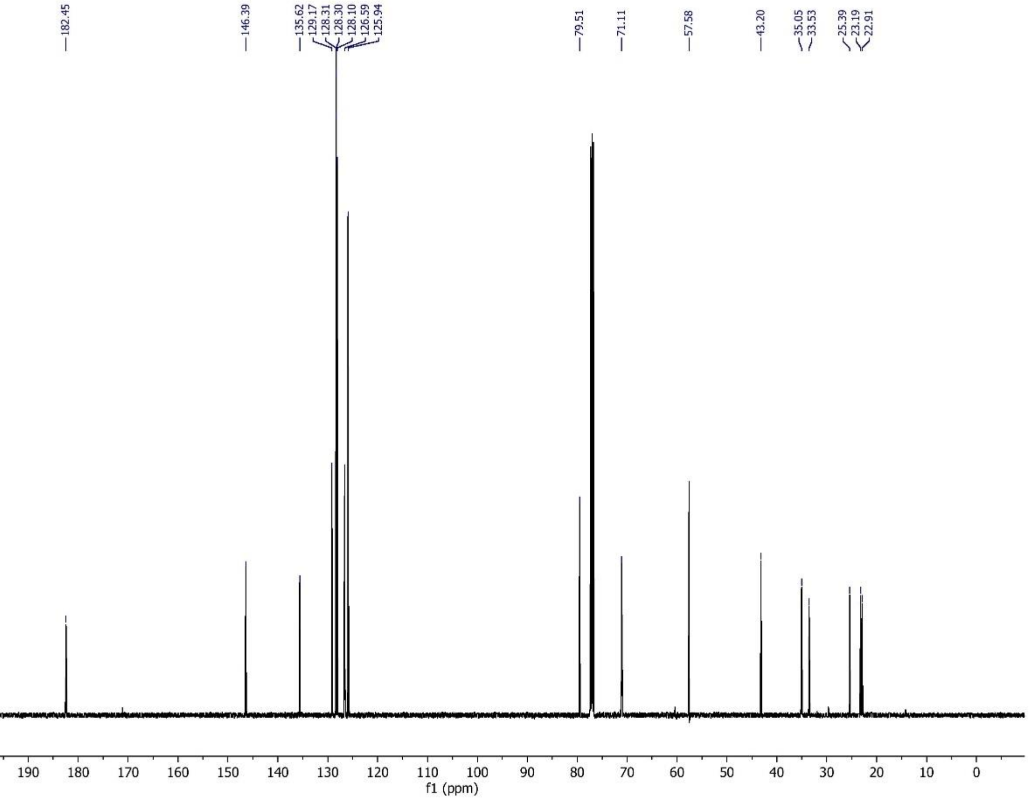

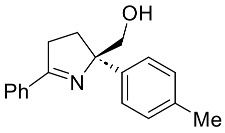

3v
^{1}H NMR
500 MHz, $CDCl_3$

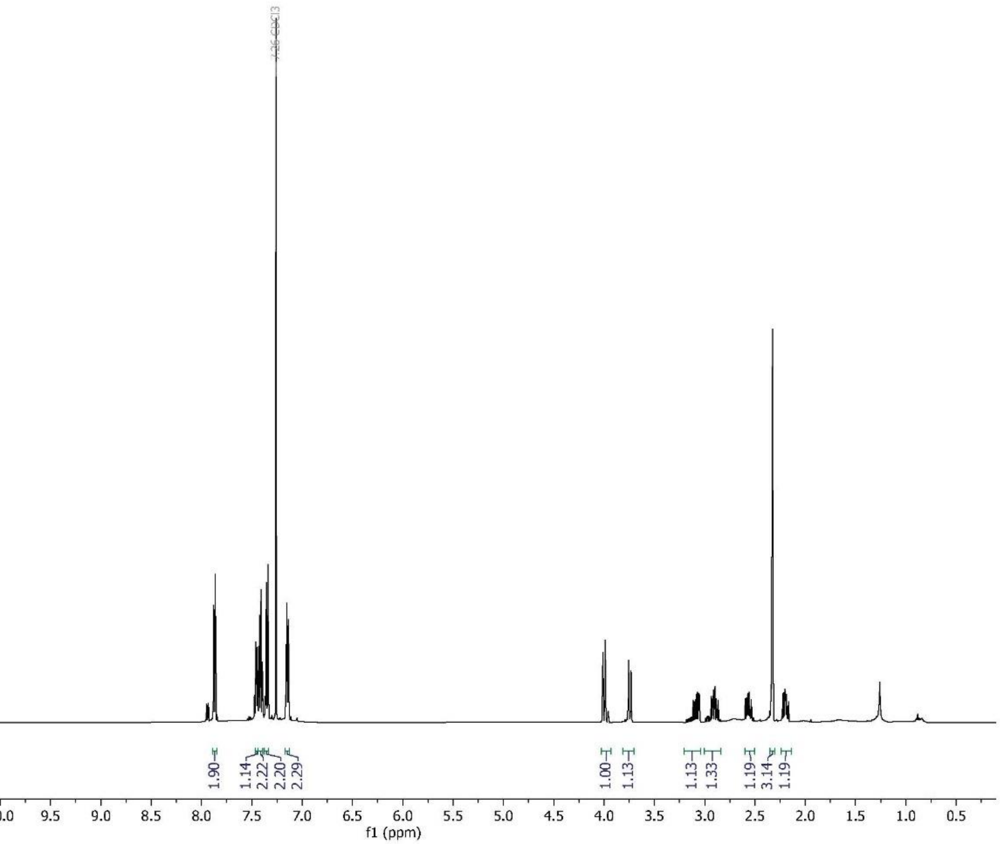

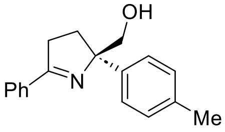

3v
^{13}C NMR
126 MHz, $CDCl_3$

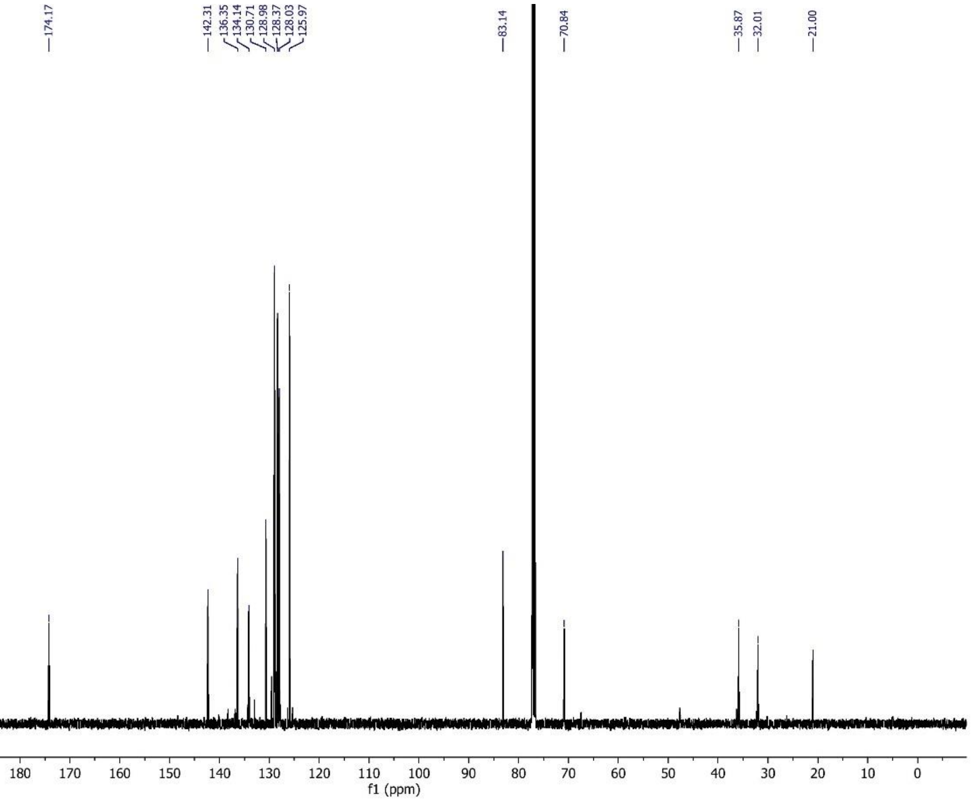

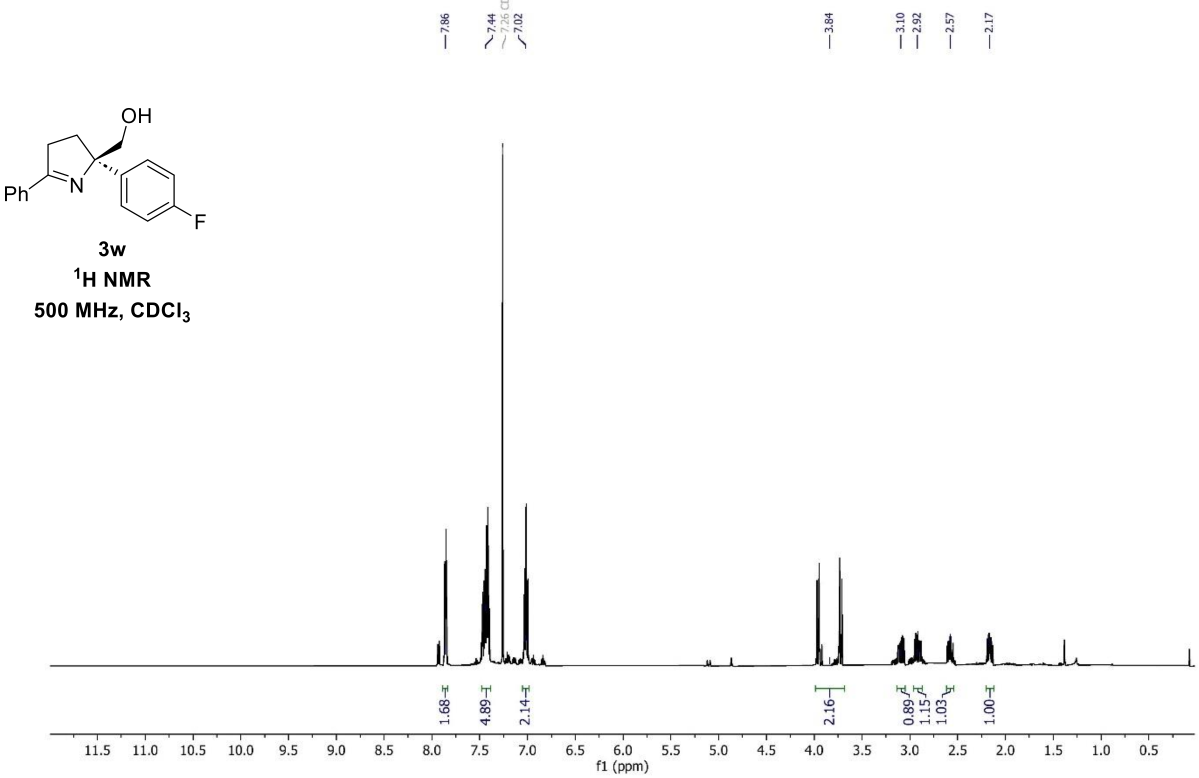

3w
^{1}H NMR
500 MHz, $CDCl_3$

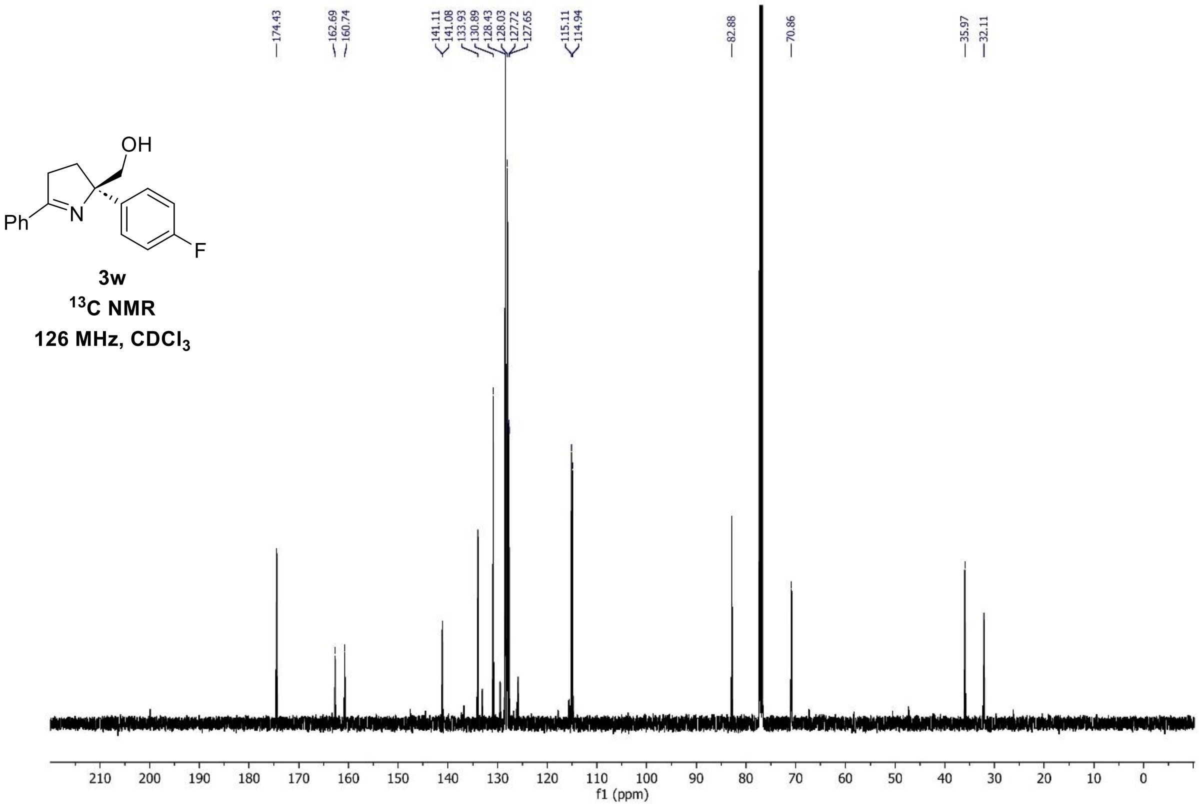

3w
^{13}C NMR
126 MHz, $CDCl_3$

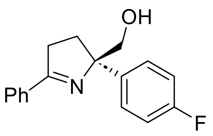

3w
^{19}F NMR
376 MHz, $CDCl_3$

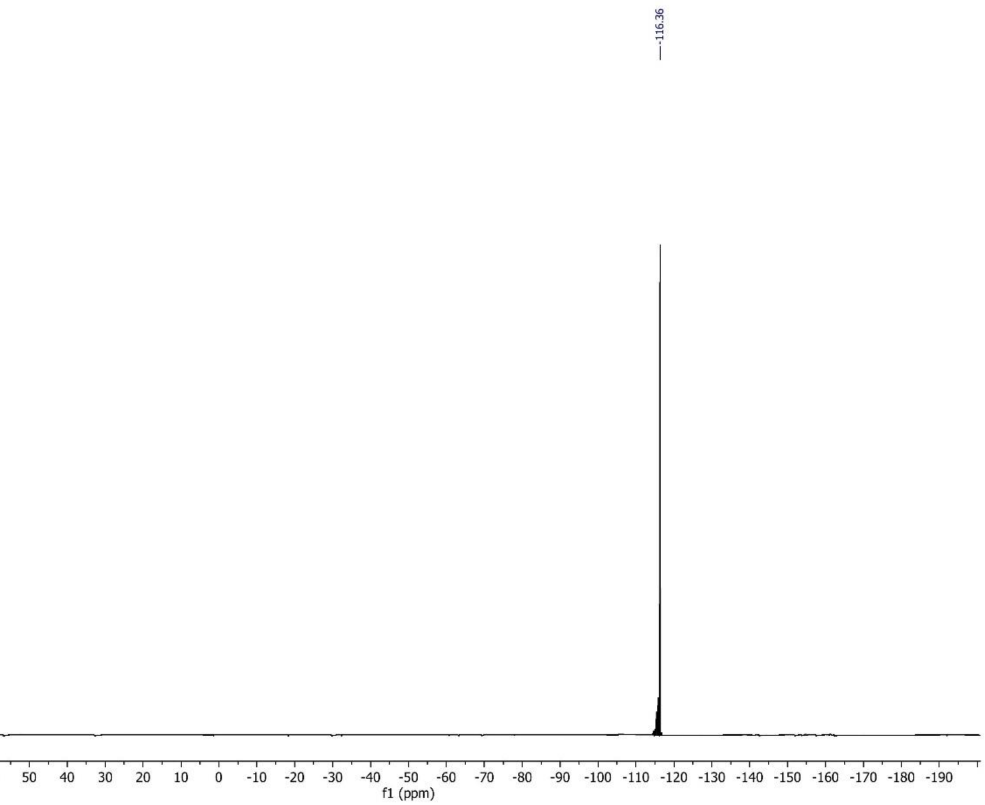

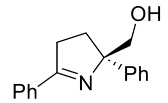

3x
^{1}H NMR
500 MHz, $CDCl_3$

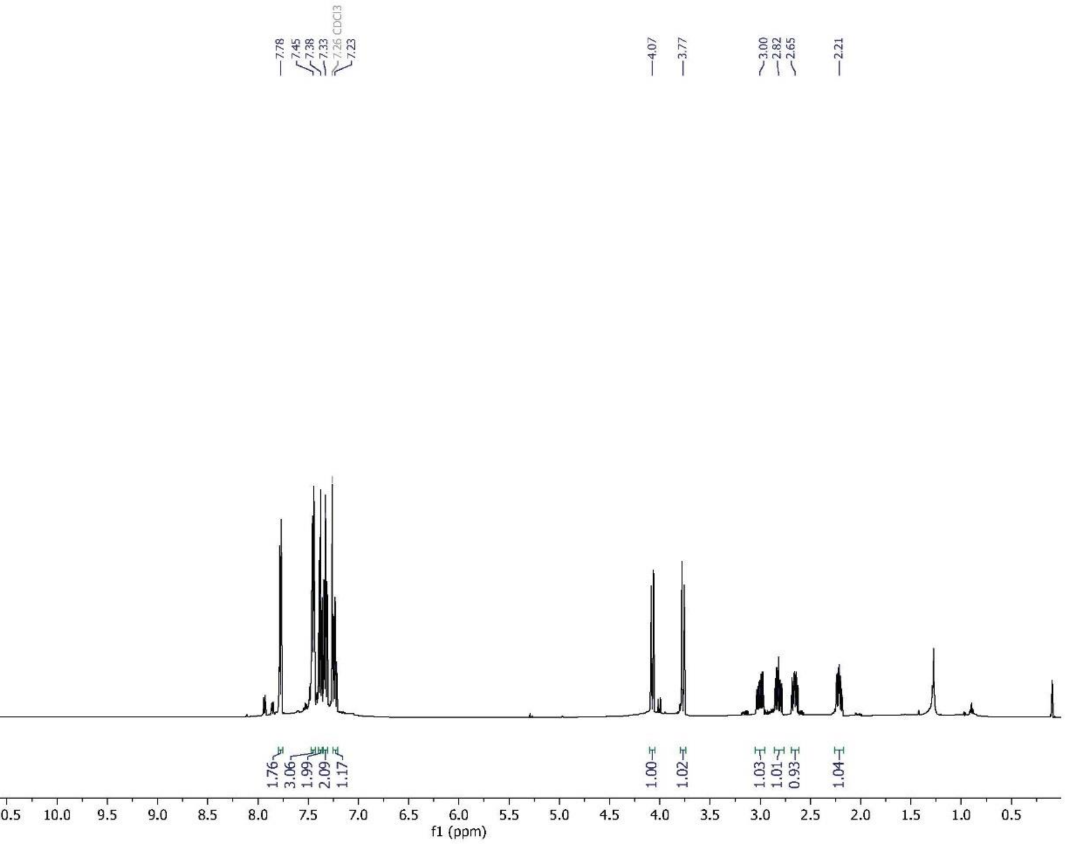

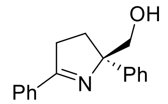

3x
^{13}C NMR
126 MHz, $CDCl_3$

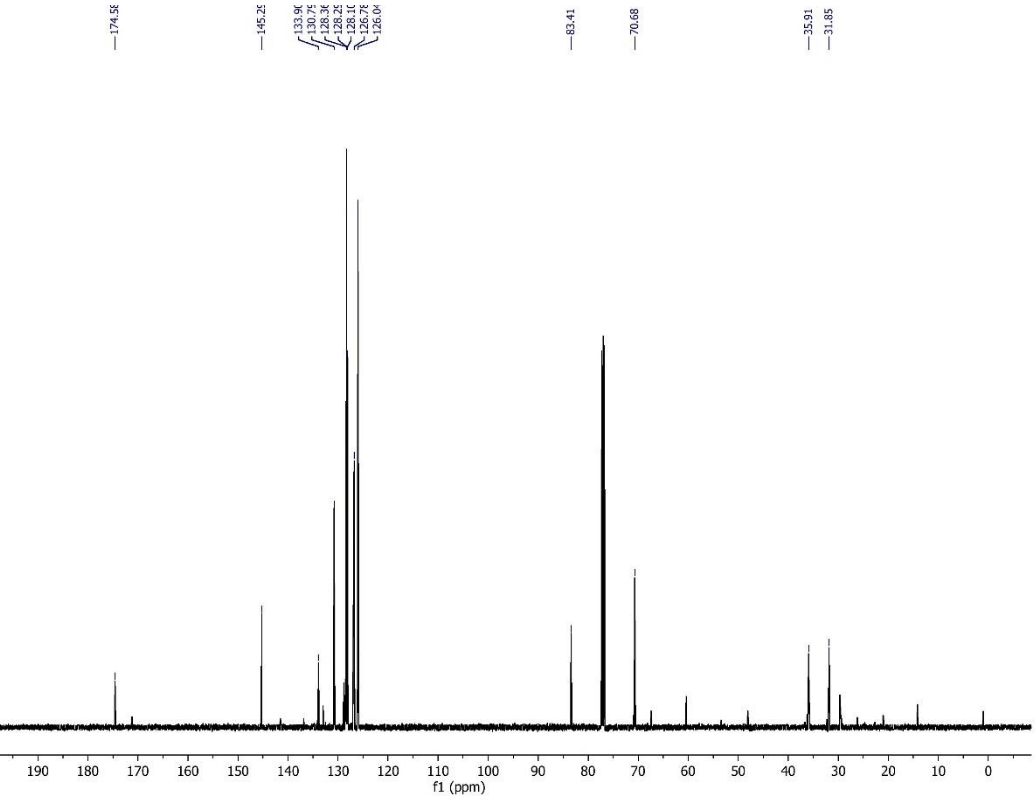

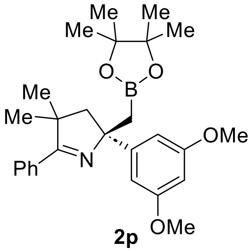

2p

^{1}H NMR

500 MHz, $CDCl_3$

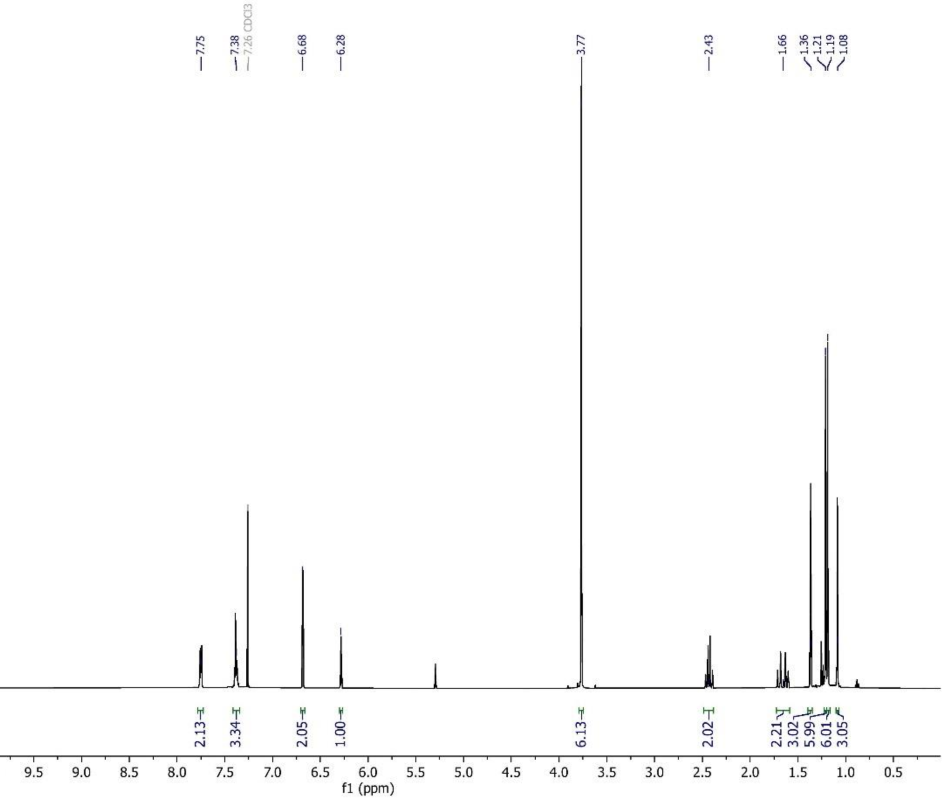

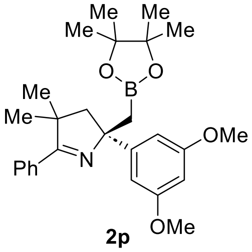

2p

^{13}C NMR

126 MHz, $CDCl_3$

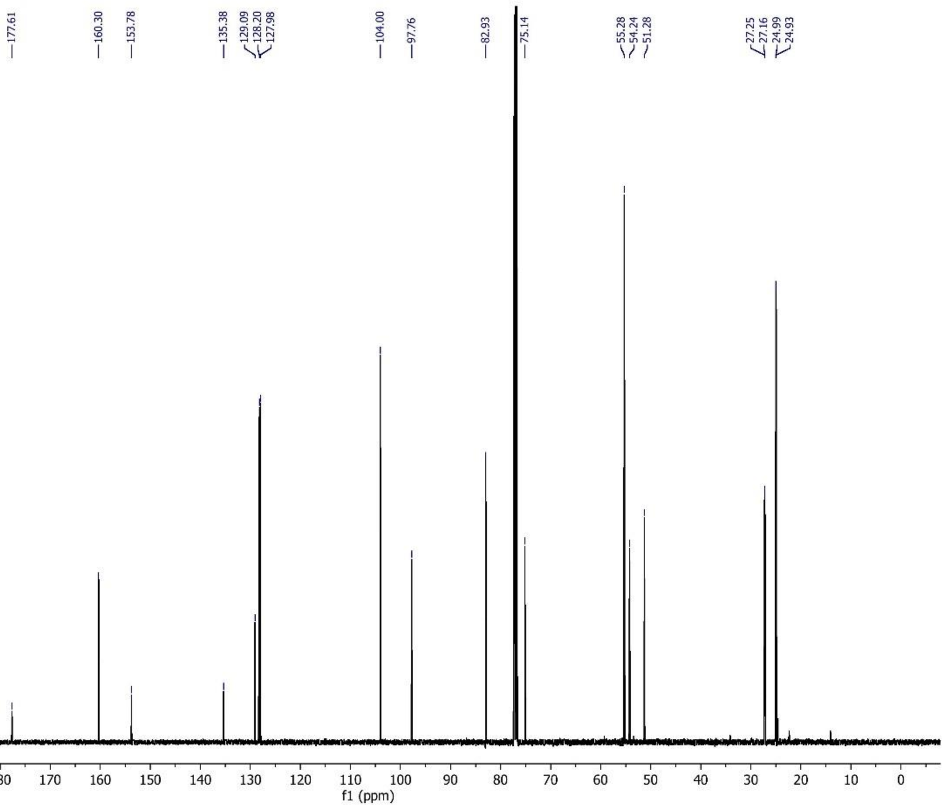

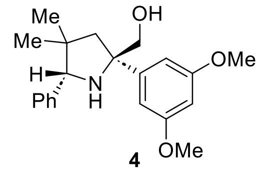

4

^{1}H NMR

500 MHz, $CDCl_3$

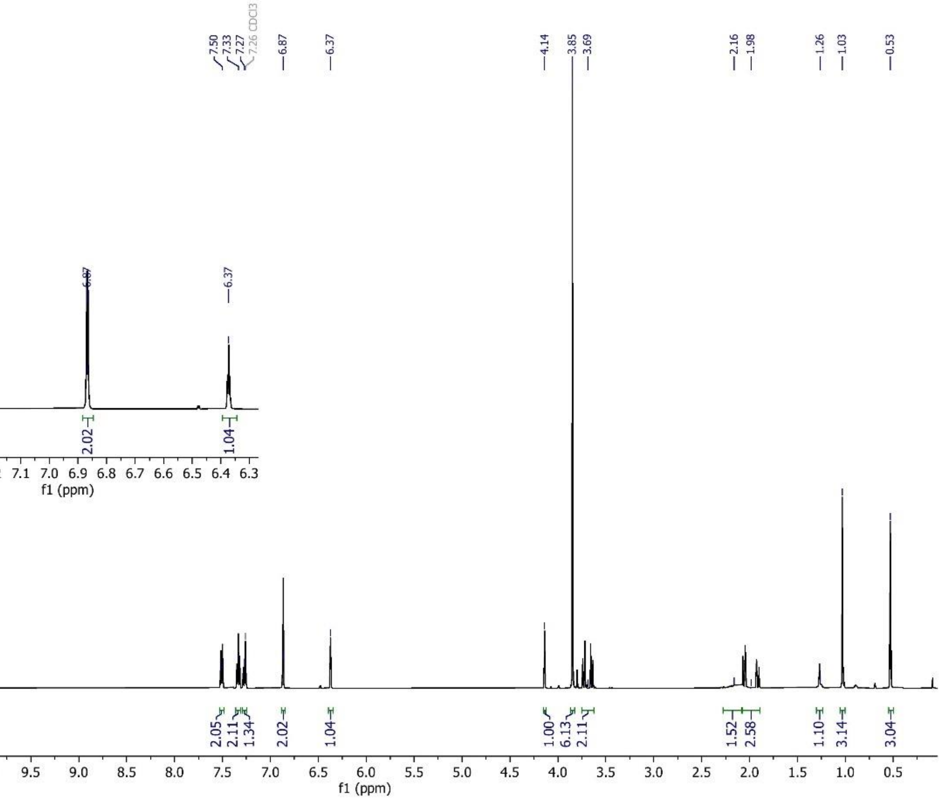

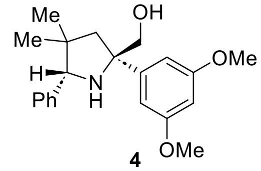

4

^{13}C NMR

101 MHz, $CDCl_3$

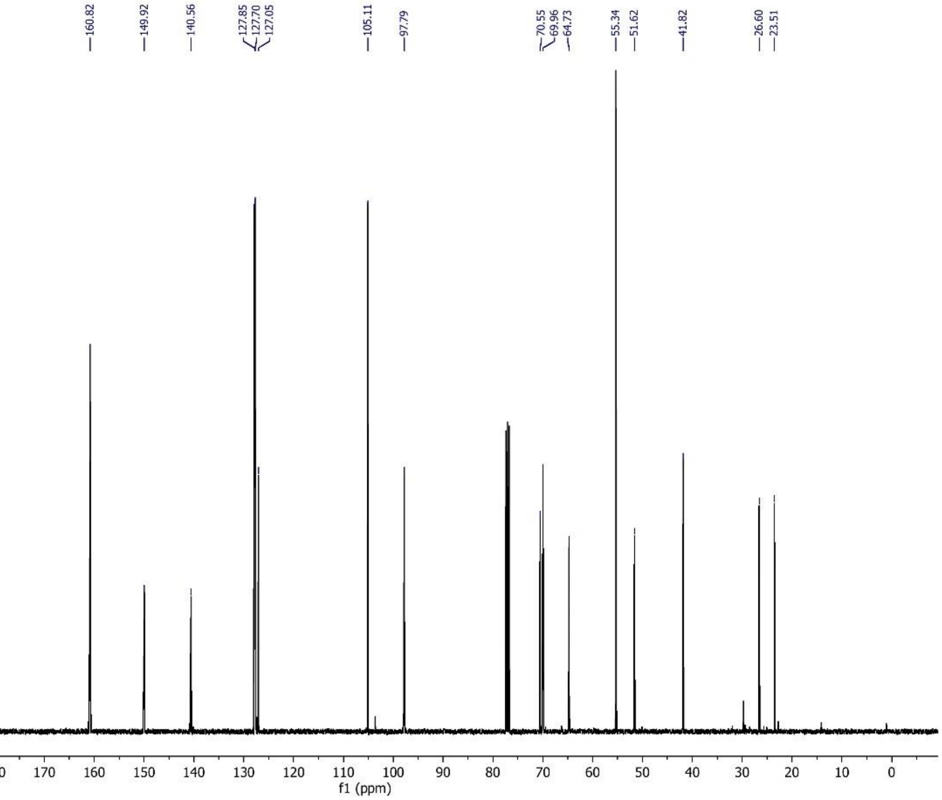

HSQC (**4**)

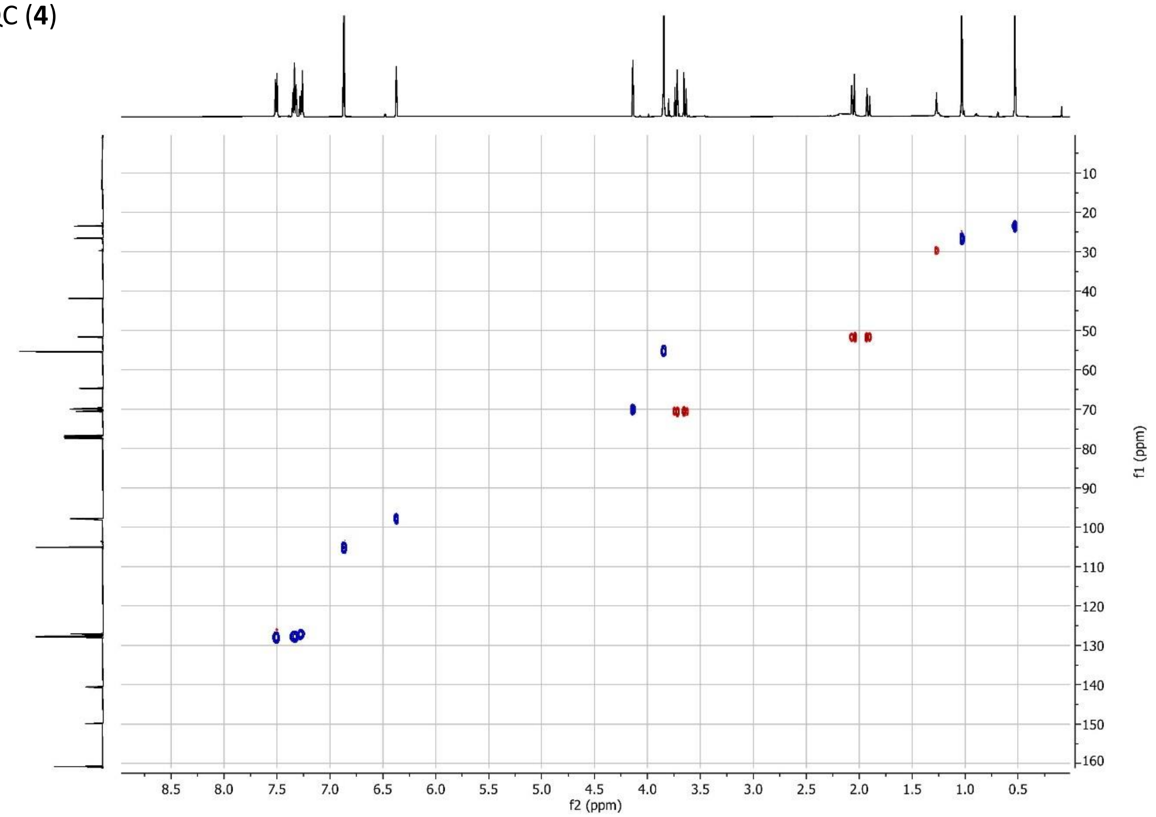

HMBC (**4**)

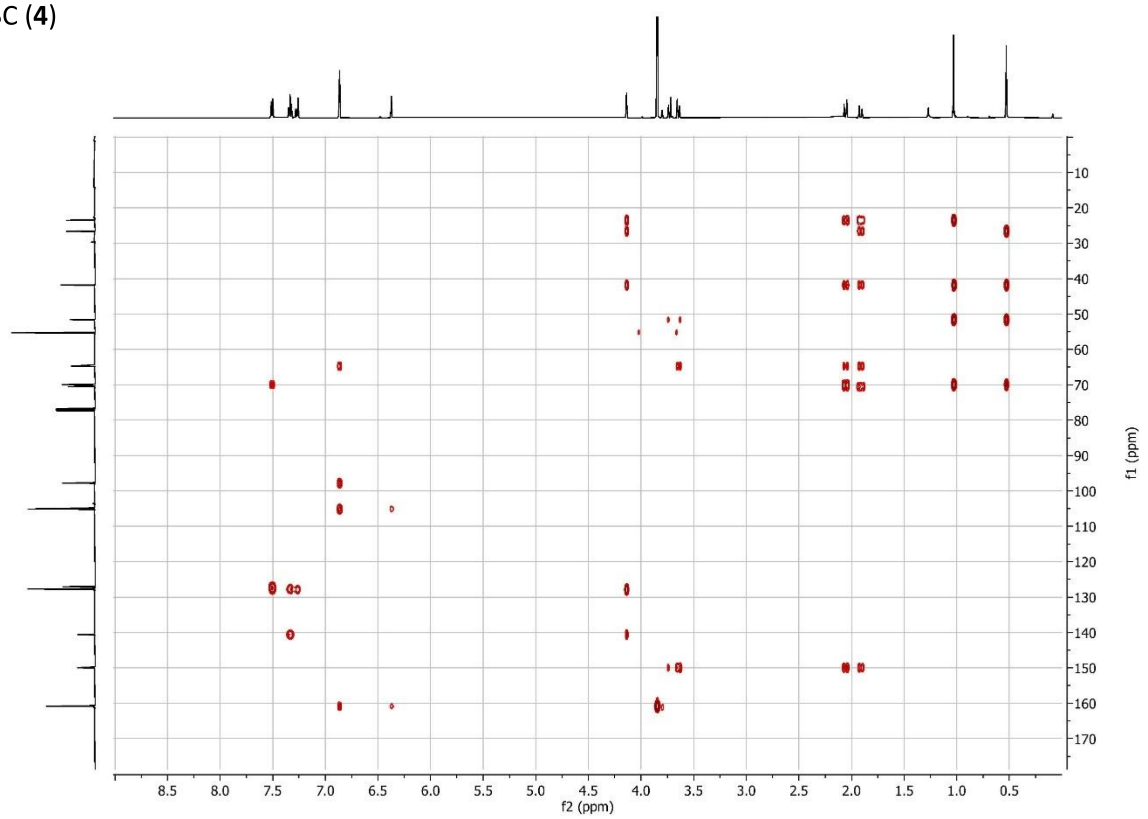

NOESY (**4**)

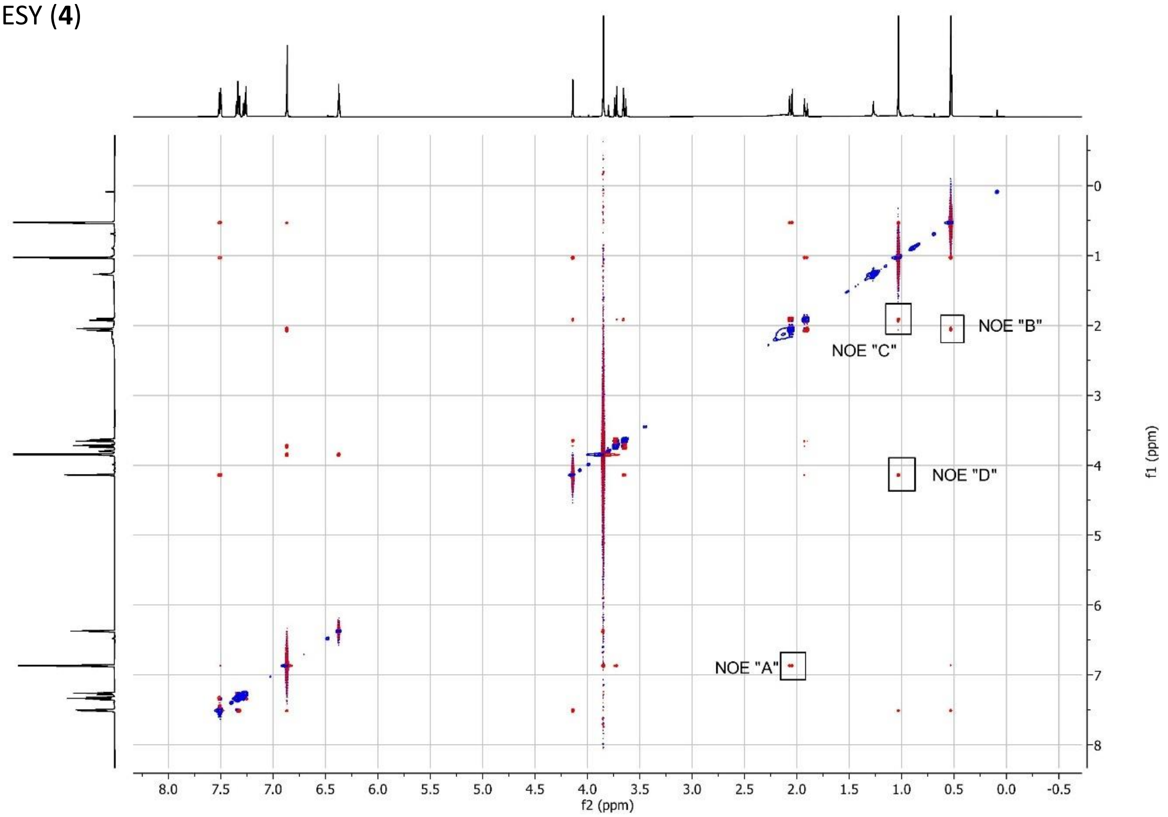
NOE "A"
NOE "B"
NOE "C"
NOE "D"
f2 (ppm)
f1 (ppm)

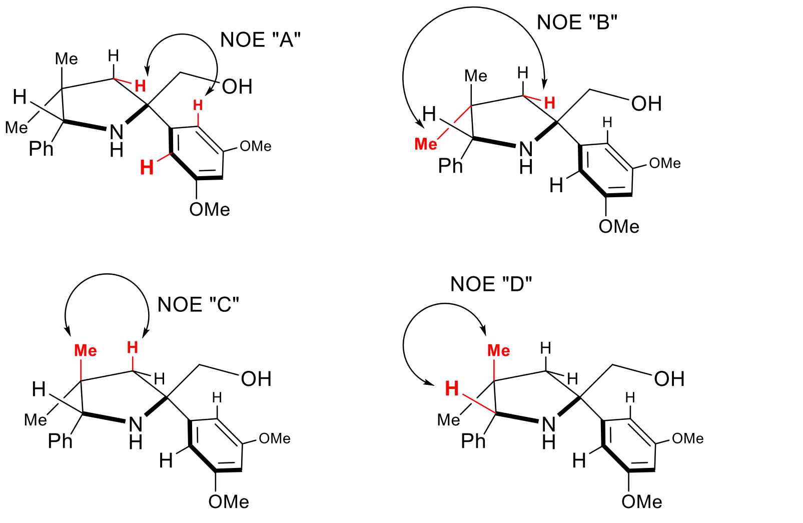
NOE "A"
NOE "B"
NOE "C"
NOE "D"
Me
Ph
OH
OMe
OMe

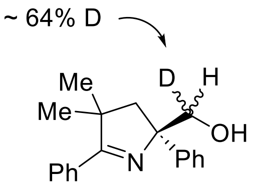

3a-D
^{1}H NMR
500 MHz, $CDCl_3$

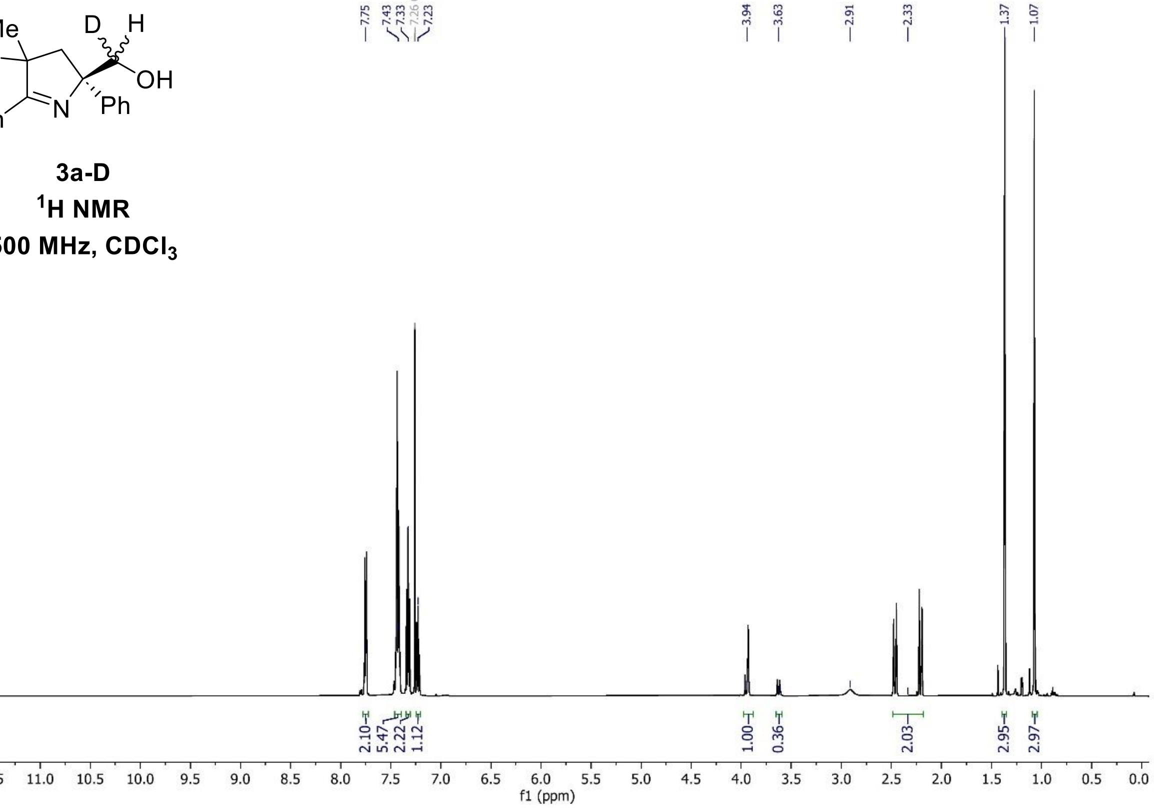

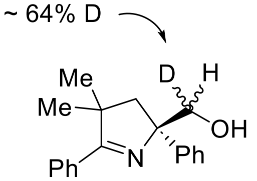

3a-D
^{13}C NMR
101 MHz, $CDCl_3$

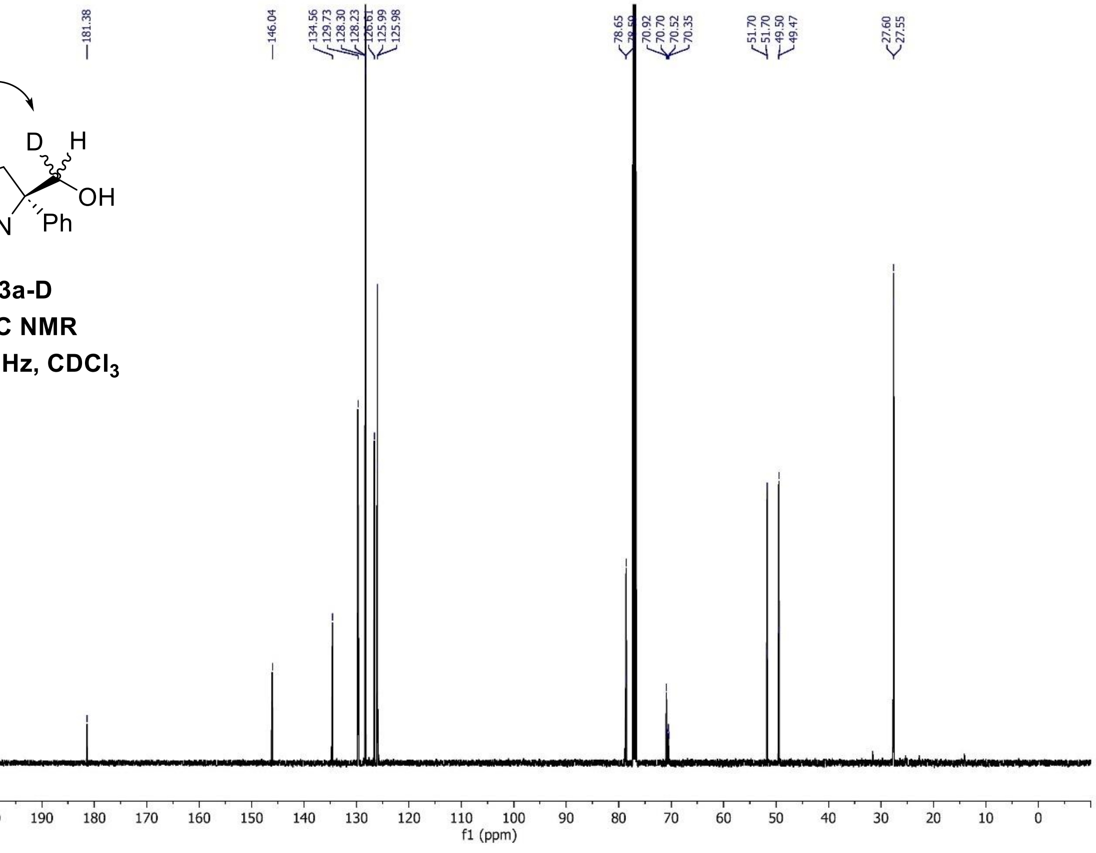

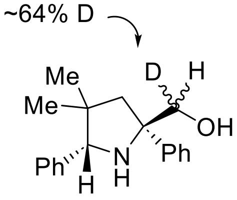

INT-S10
^{1}H NMR
600 MHz, $CDCl_3$

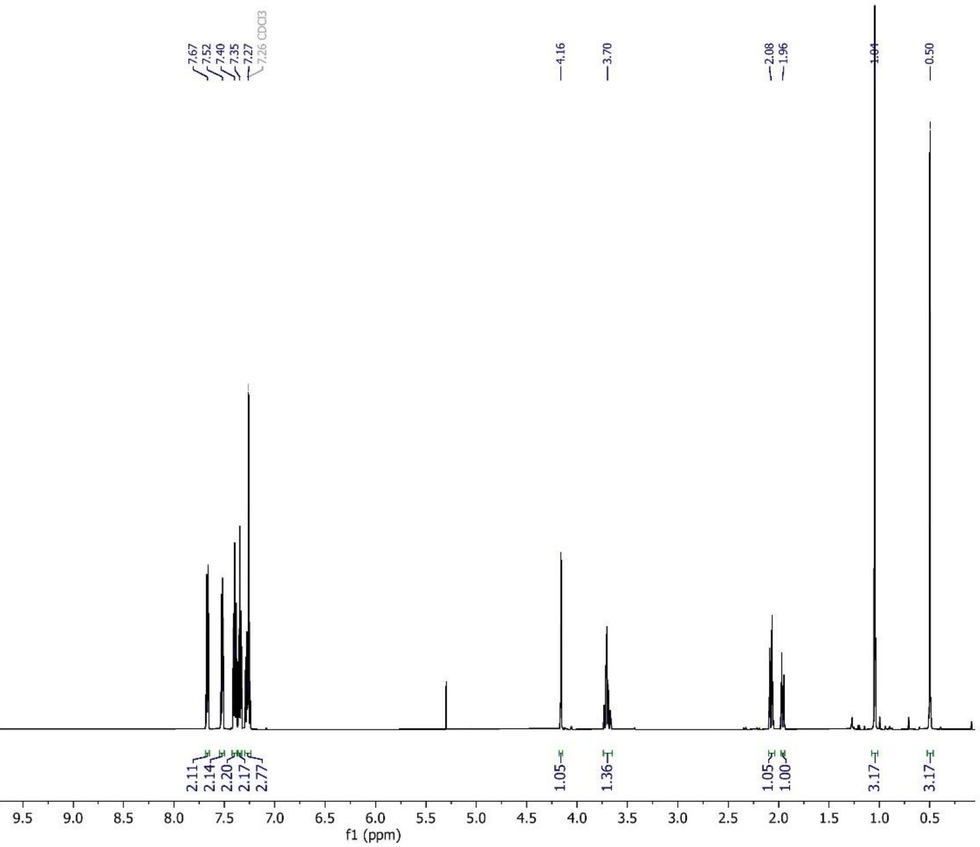

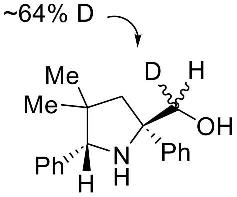

INT-S10
^{13}C NMR
101 MHz, $CDCl_3$

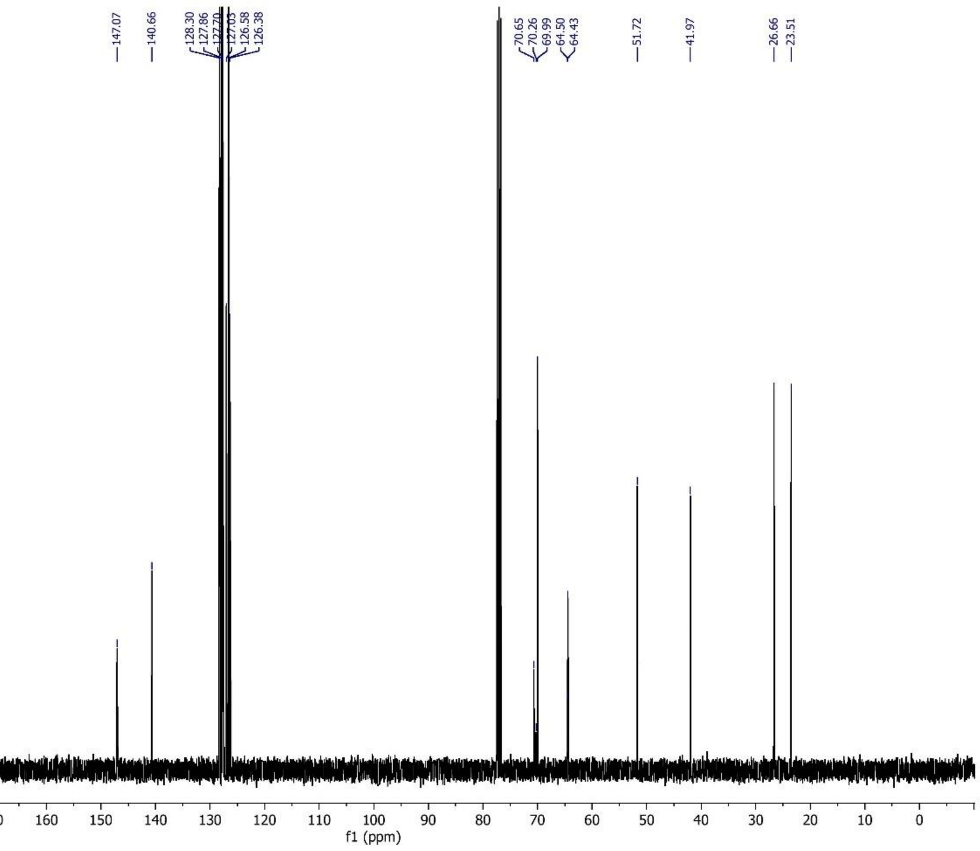

HSQC (**INT-S10**)

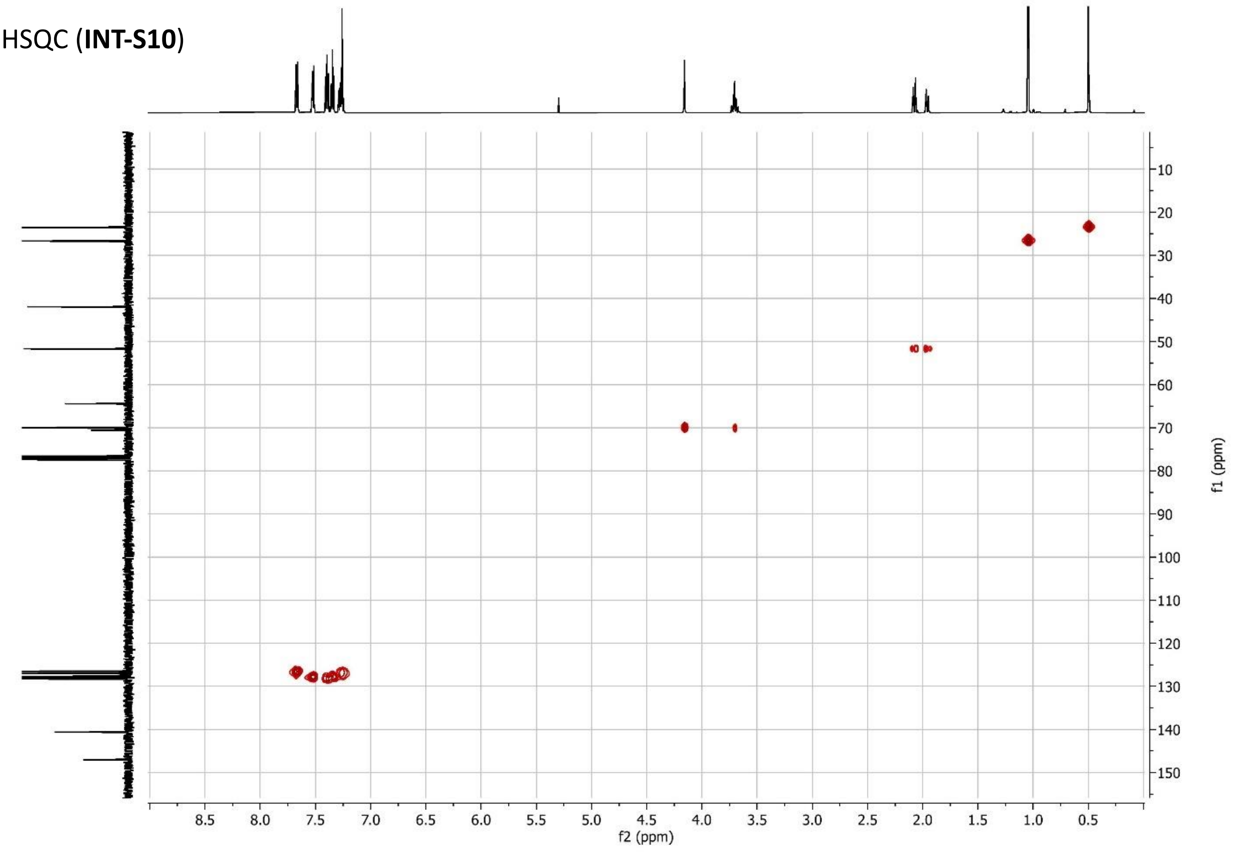

HMBC (**INT-S10**)

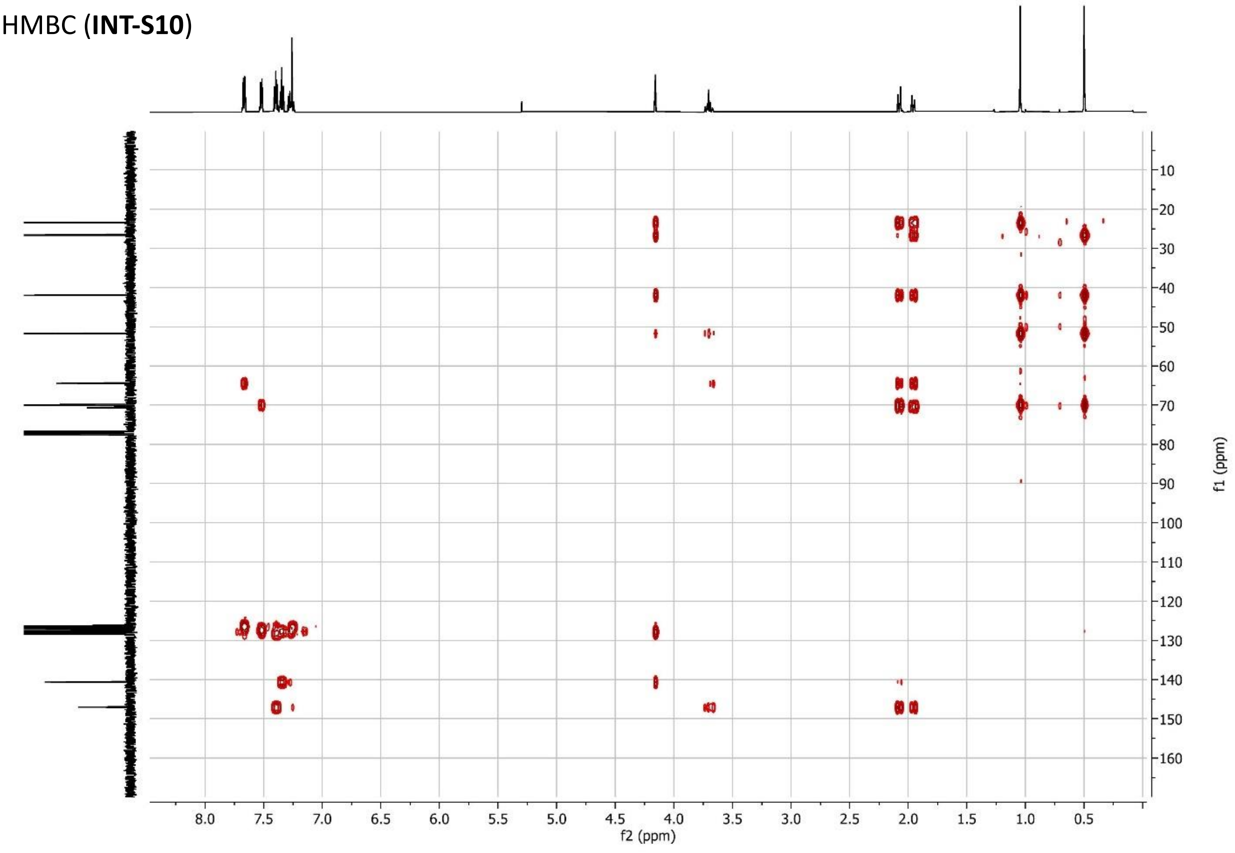

NOESY (**INT-S10**)

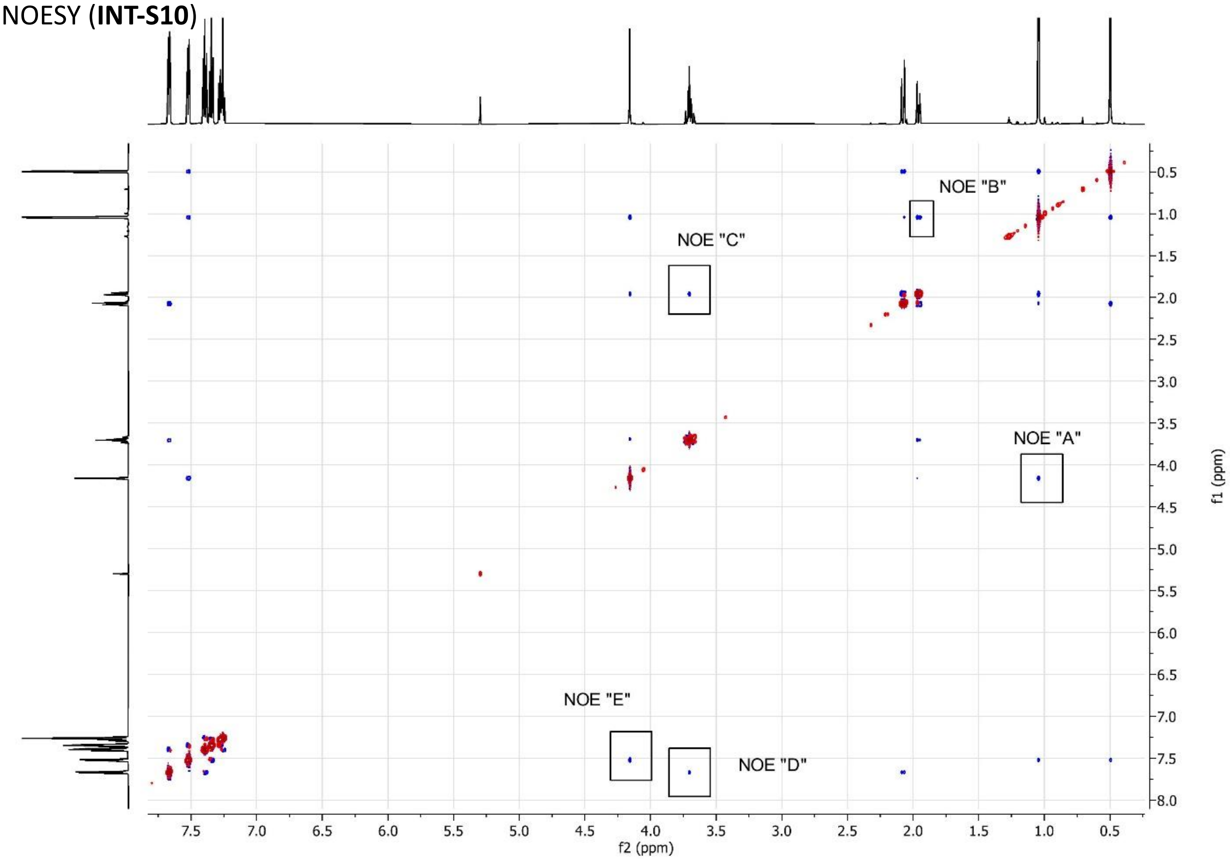

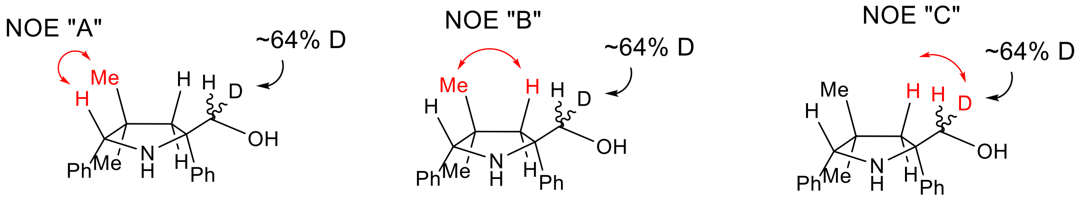

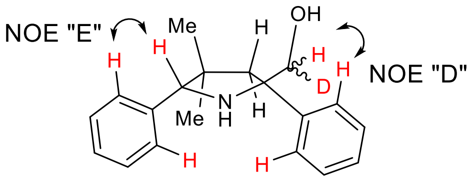

~64% D

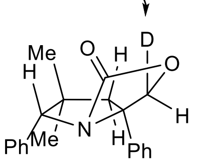

5

^{1}H NMR

600 MHz, $CDCl_3$

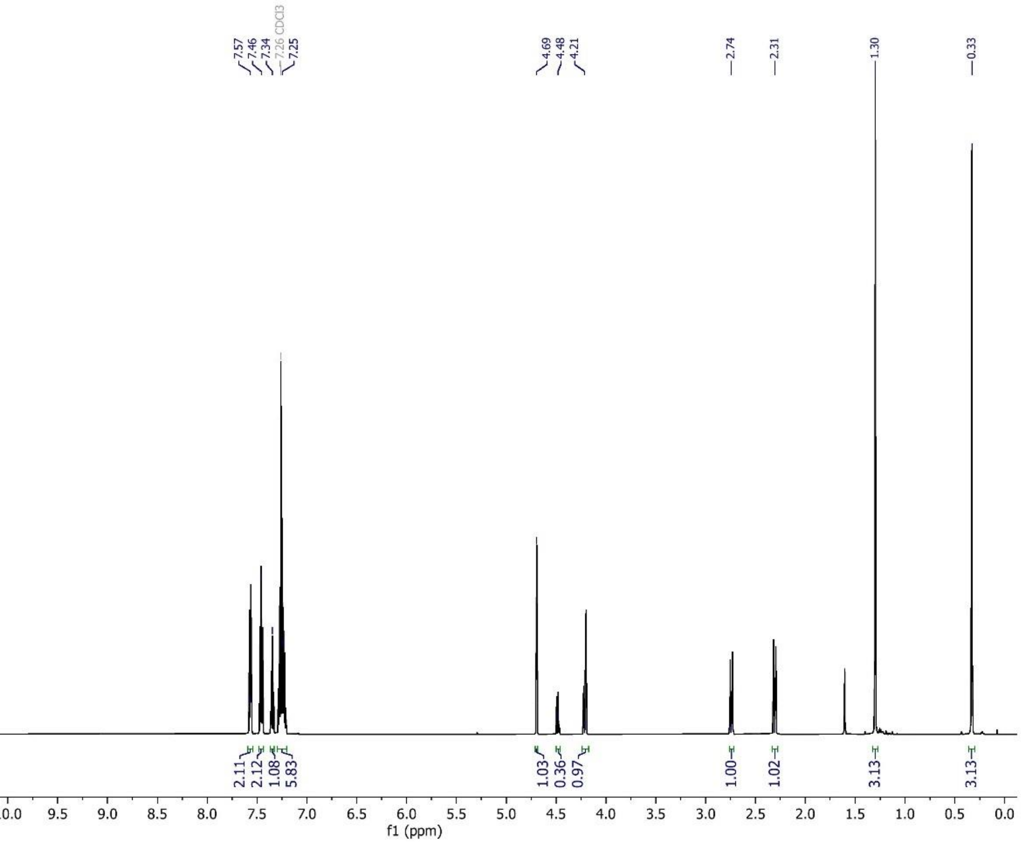

~64% D

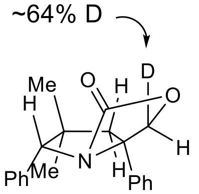

5

^{13}C NMR

101 MHz, $CDCl_3$

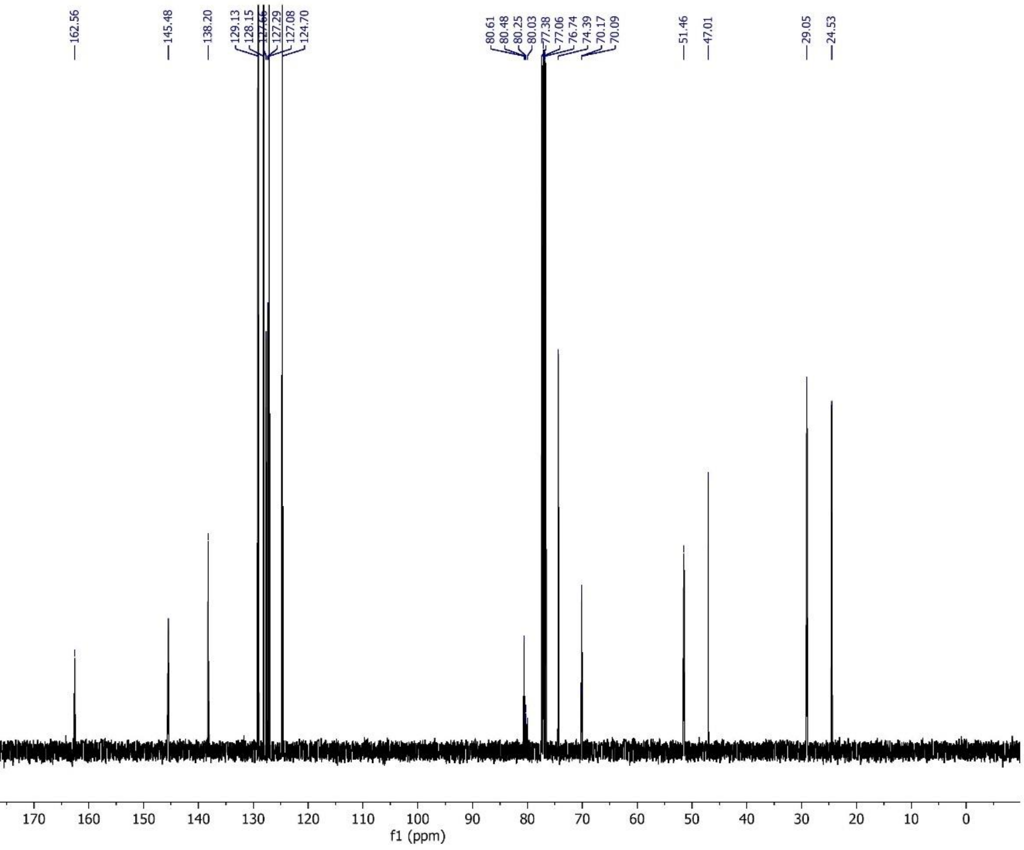

HSQC (**5**)

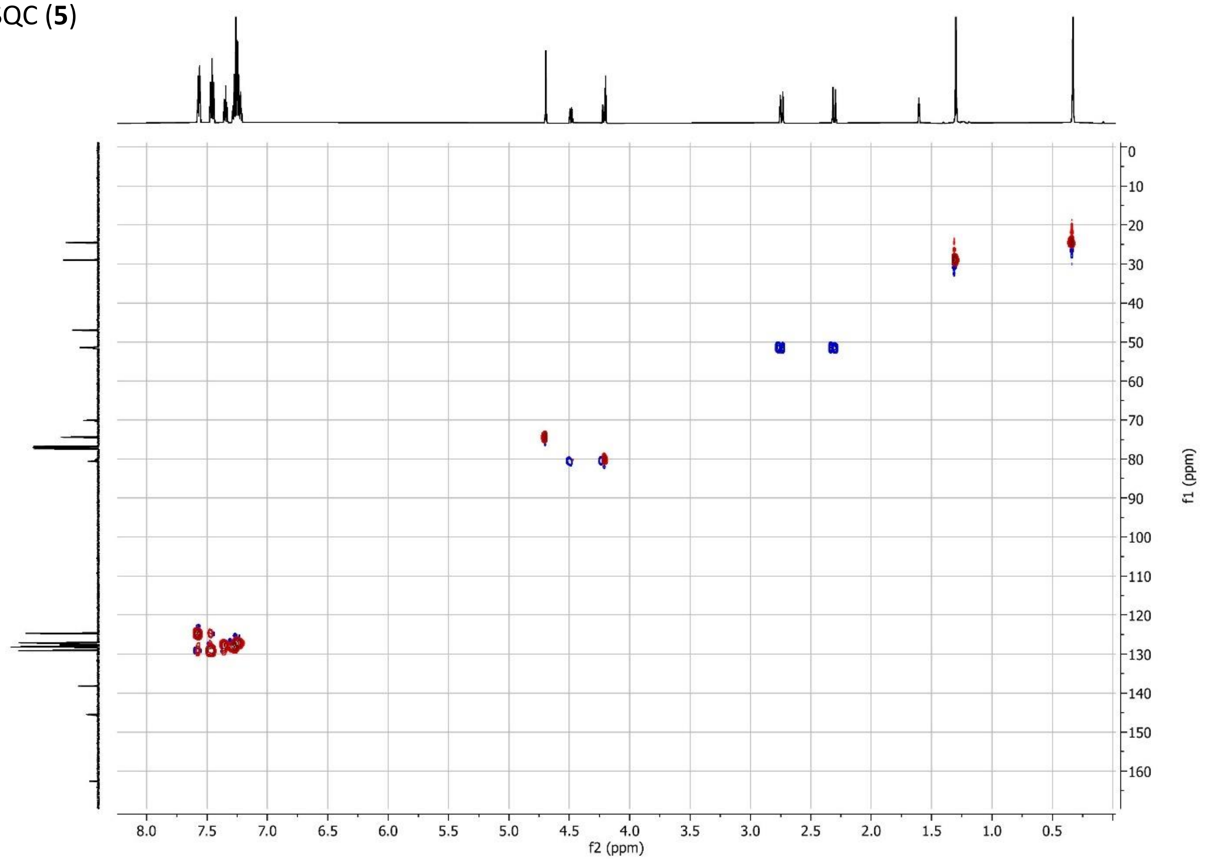

HMBC (**5**)

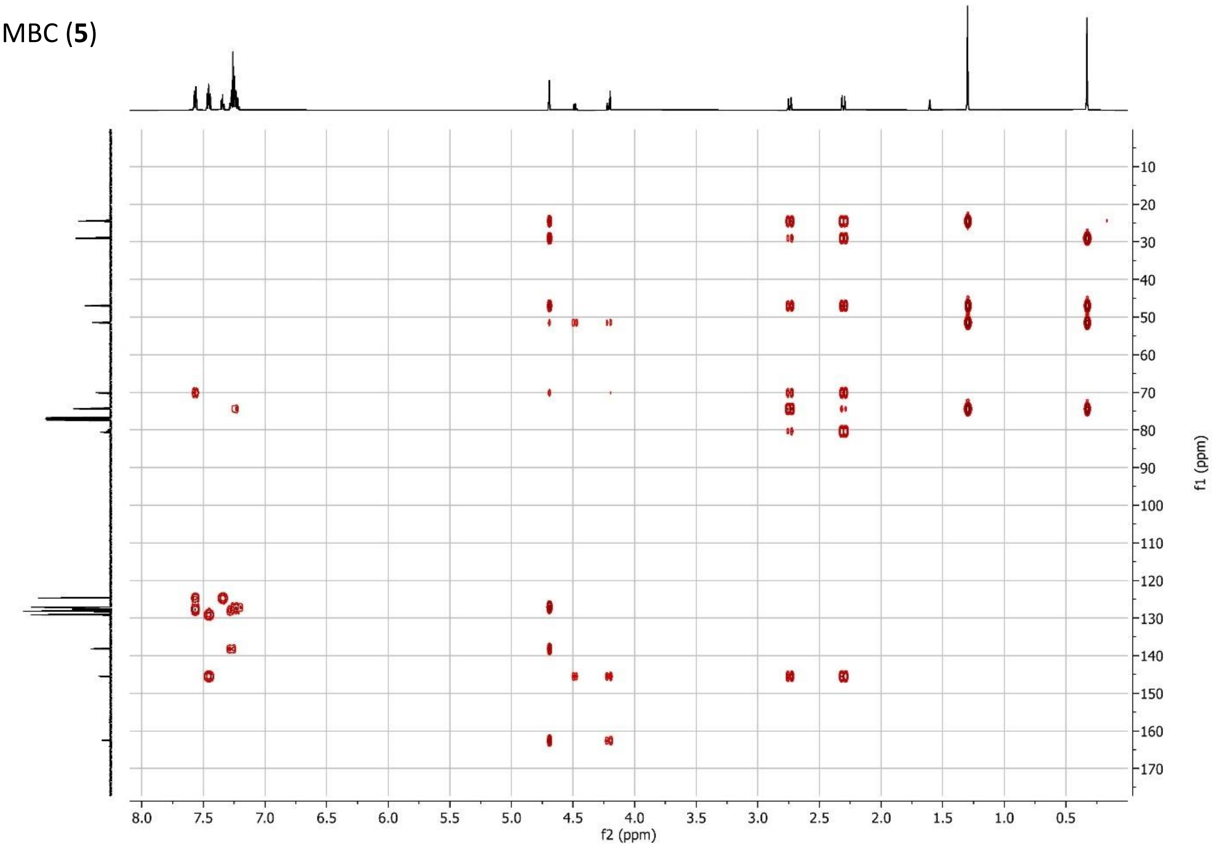

NOESY (**5**)

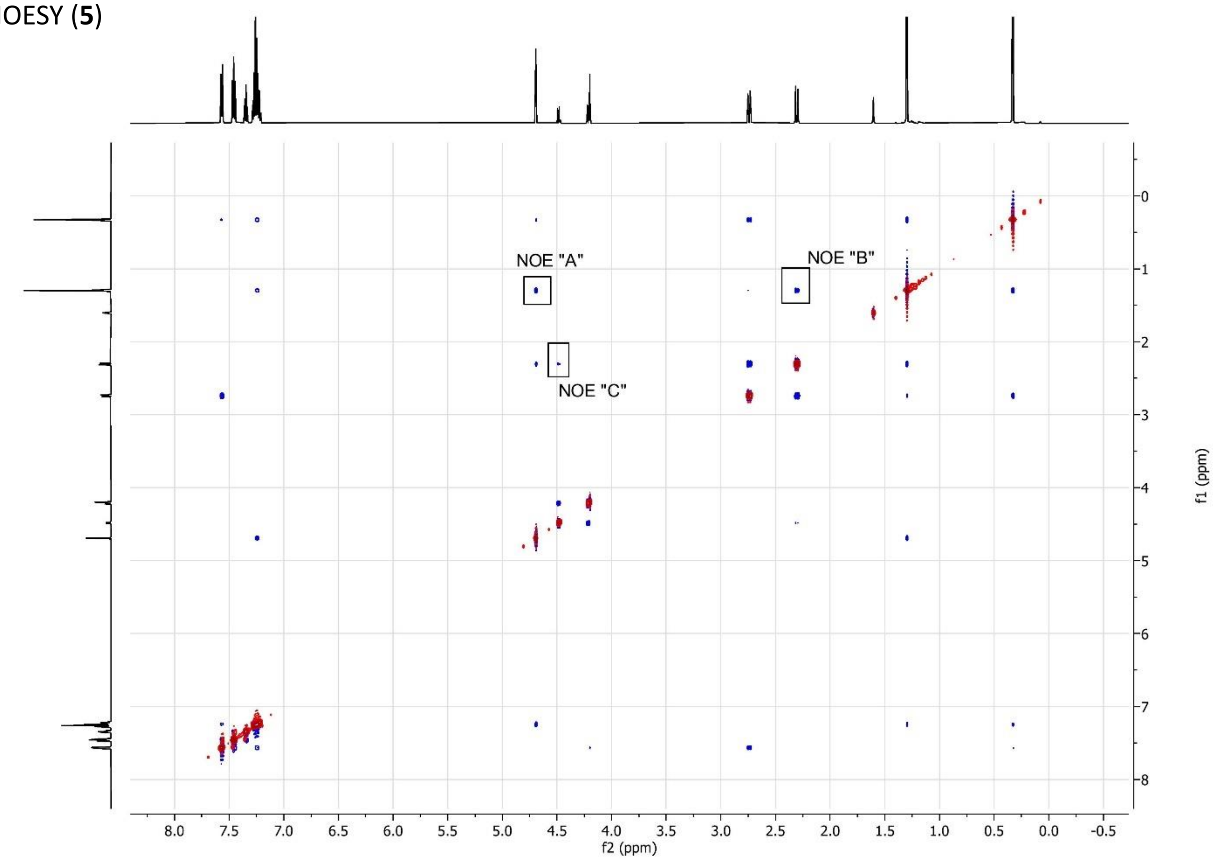

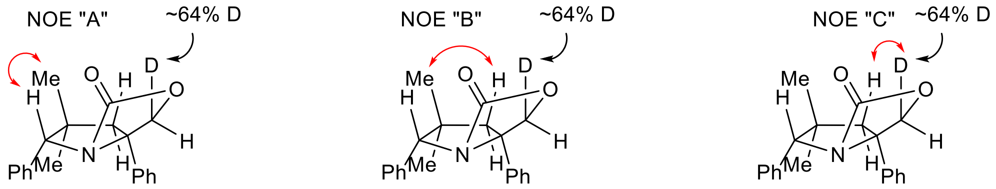

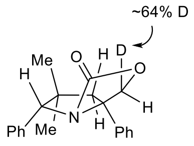

5
Deuterium NMR, 600 MHz
$CHCl_3$ - $CDCl_3$ spiked

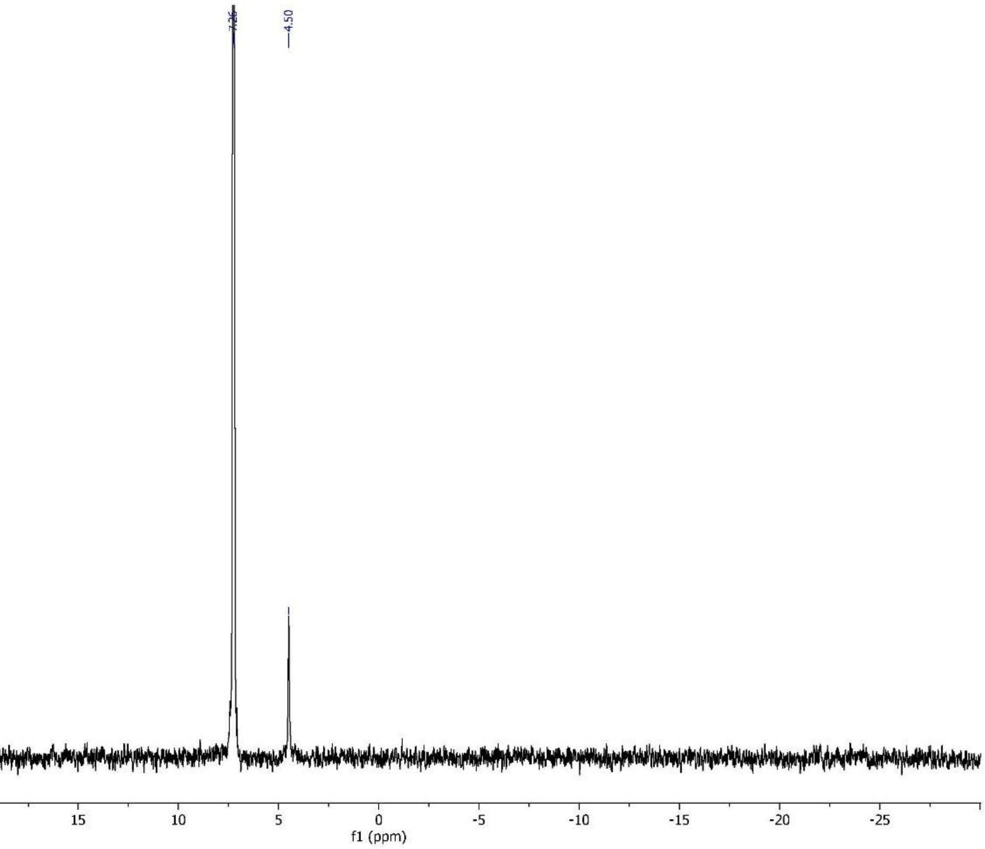

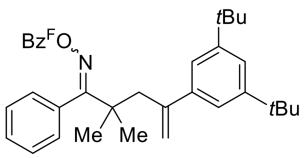

1o
^{1}H NMR
300 MHz, $CDCl_3$

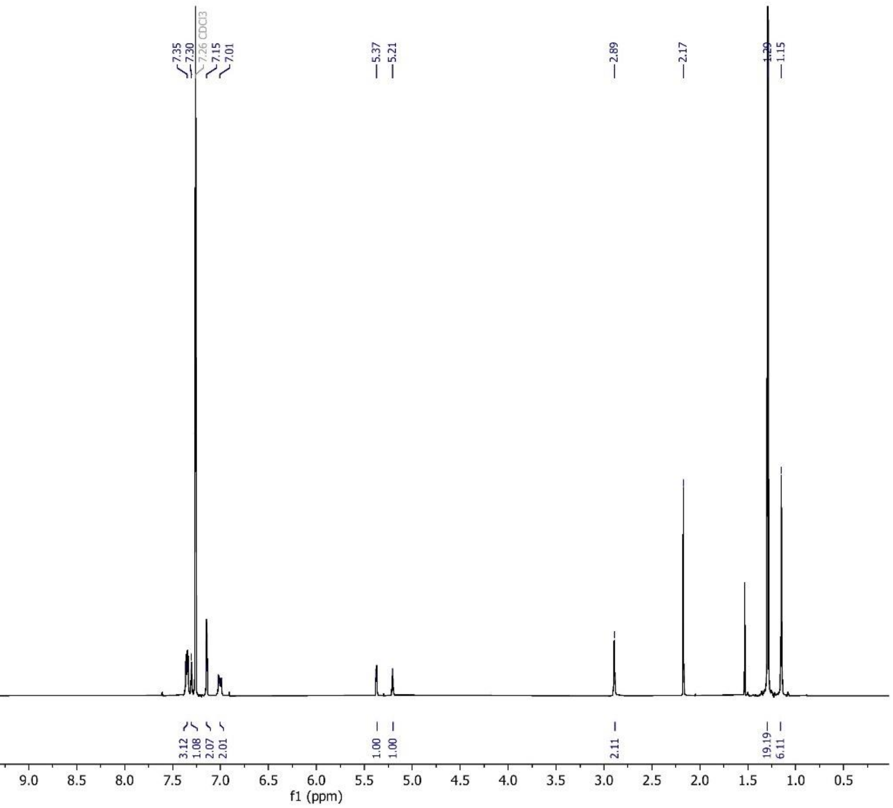

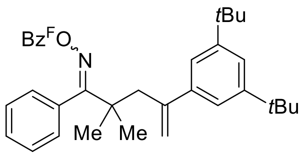

1o
^{13}C NMR
126 MHz, $CDCl_3$

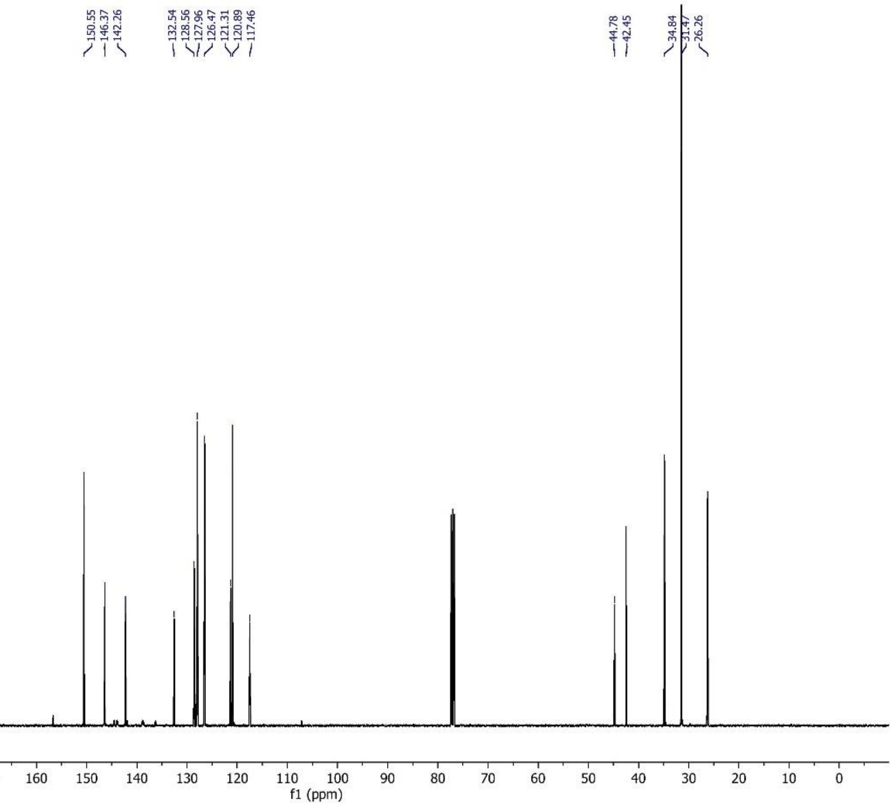

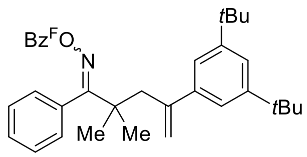

1o
^{19}F NMR
282 MHz, $CDCl_3$

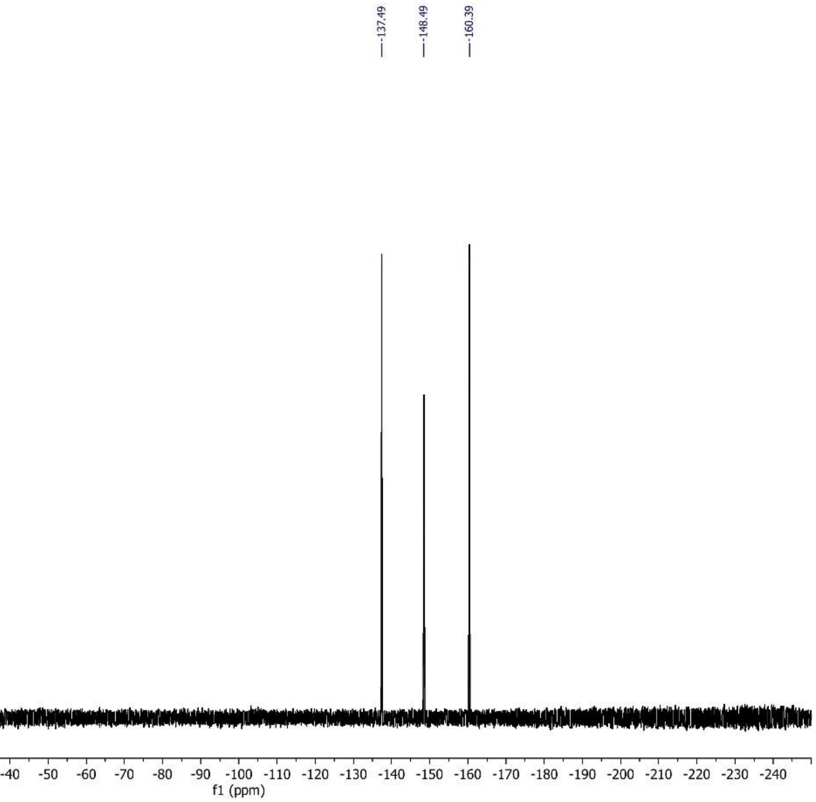

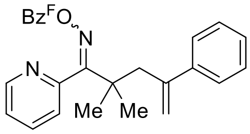

1s

^{1}H NMR

400 MHz, $CDCl_3$

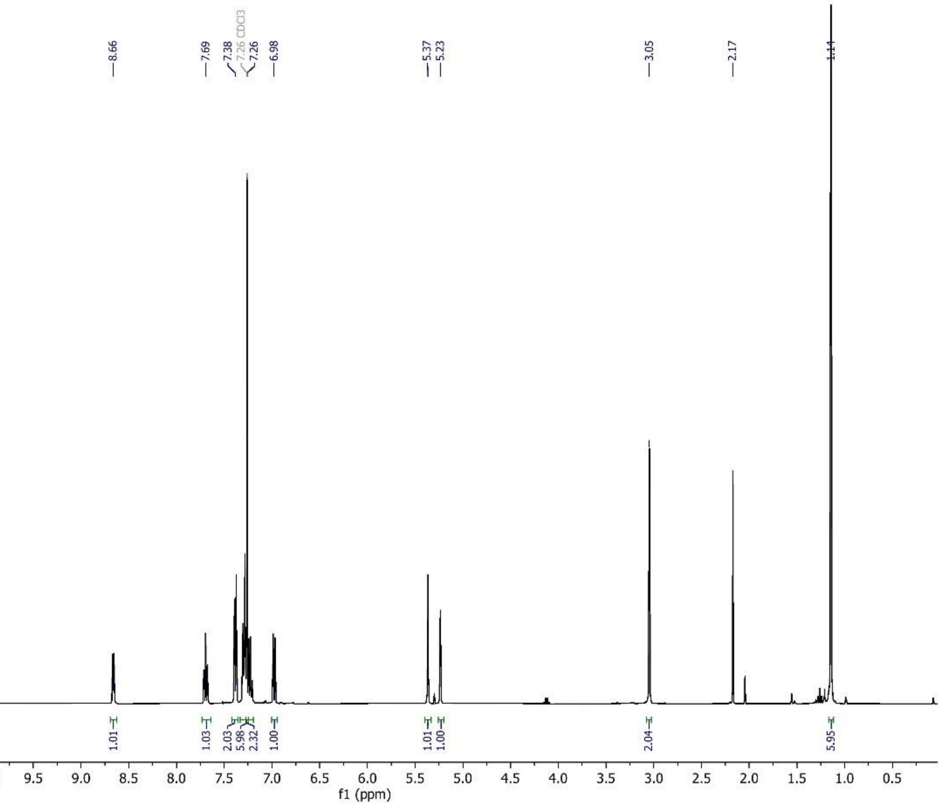

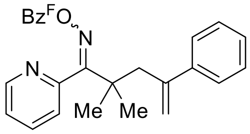

1s

^{13}C NMR

101 MHz, $CDCl_3$

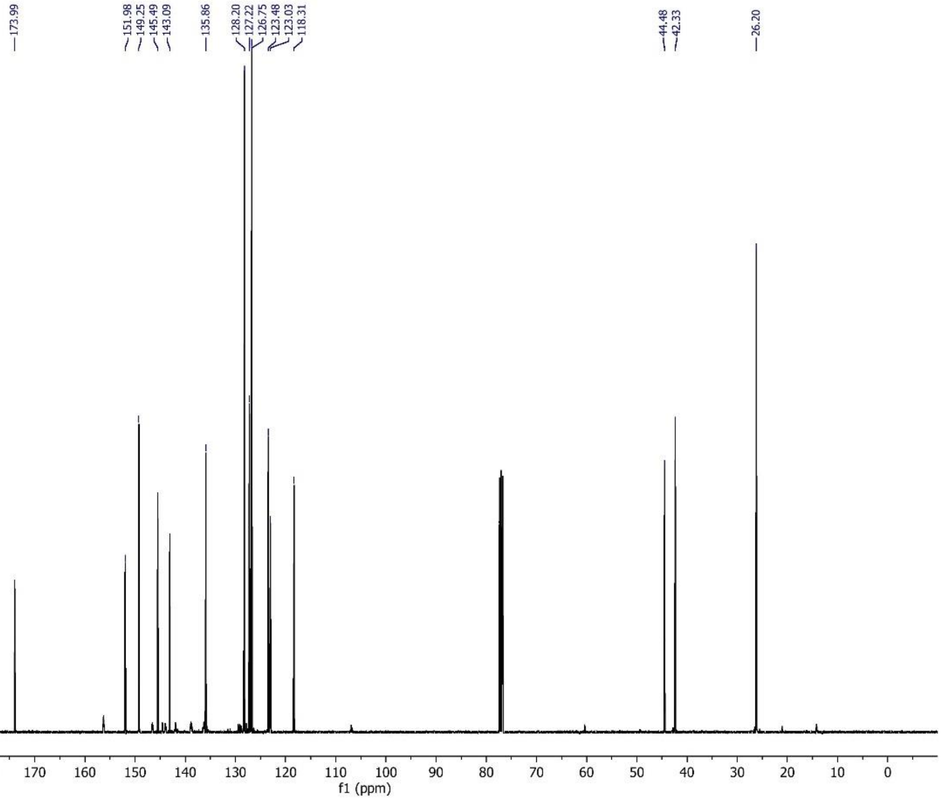

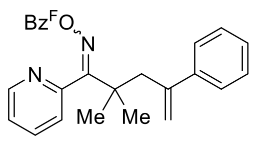

1s
^{19}F NMR
376 MHz, $CDCl_3$

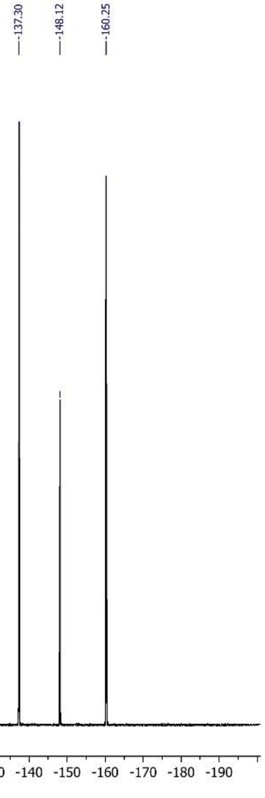

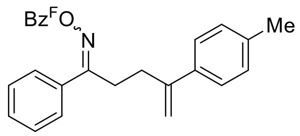

1v
^{1}H NMR
400 MHz, CDCl$_3$

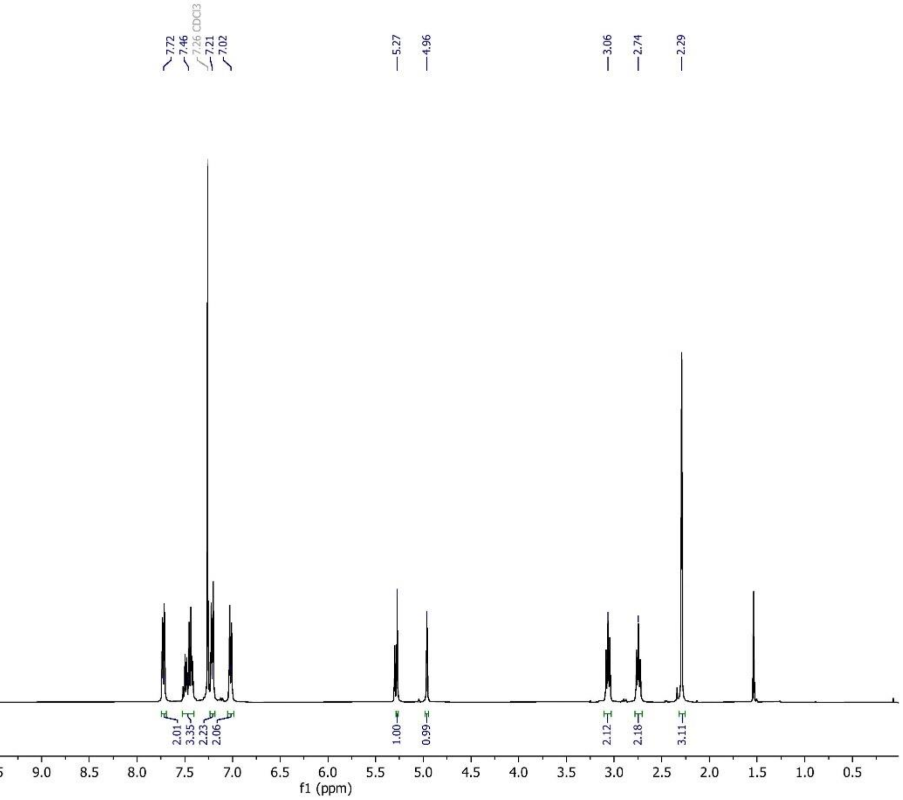

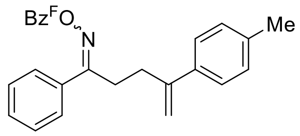

1v
^{13}C NMR
101 MHz, CDCl$_3$

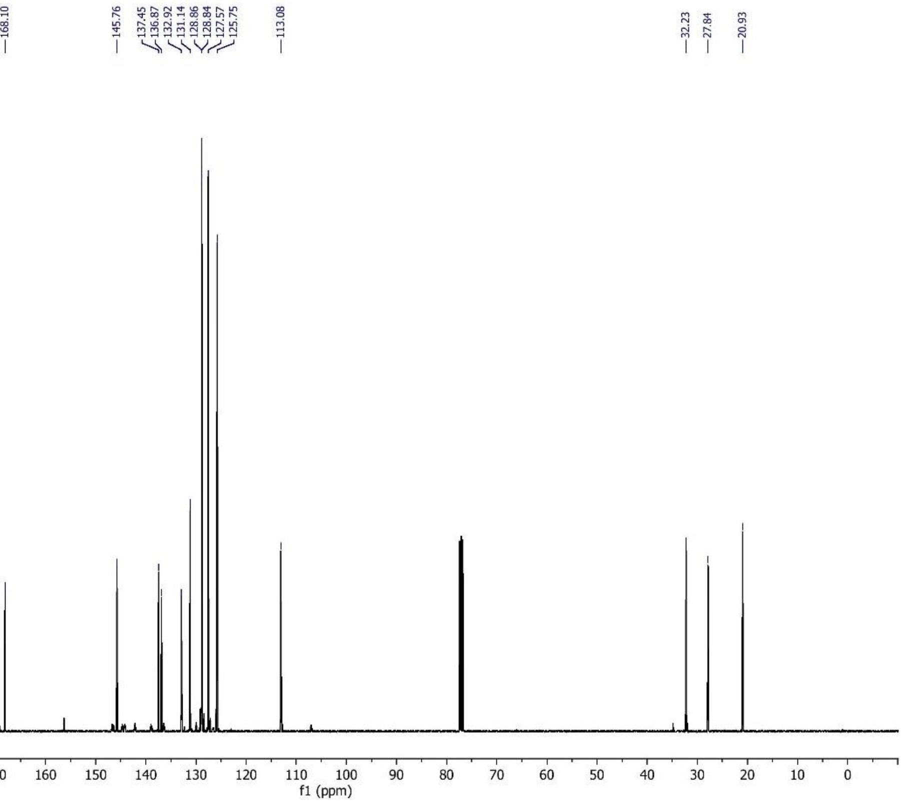

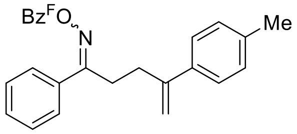

1v
^{19}F NMR
376 MHz, $CDCl_3$

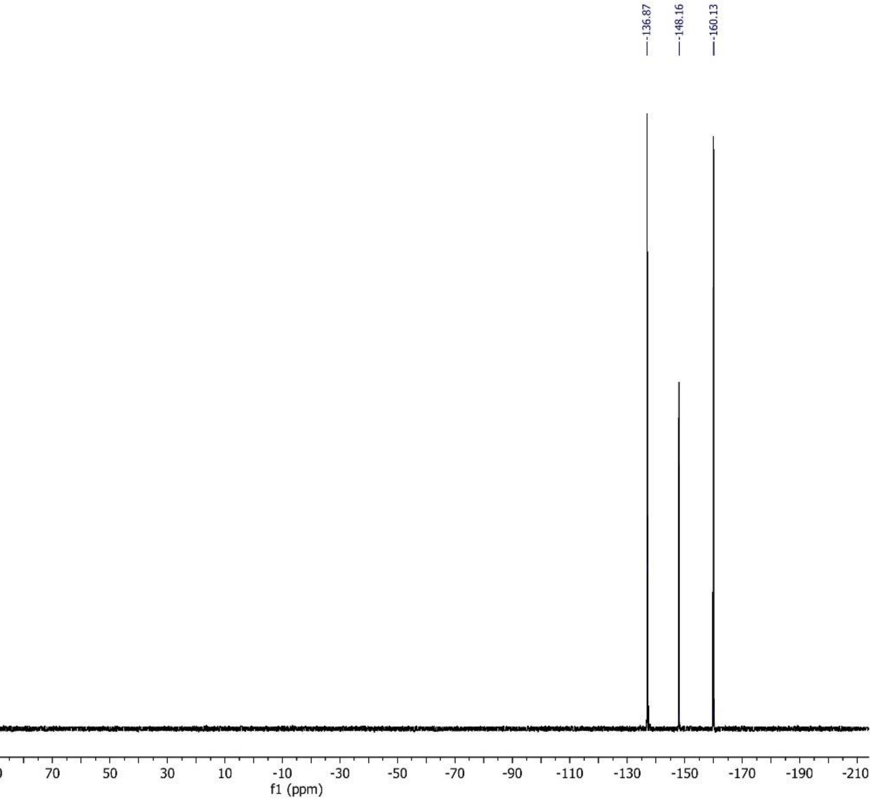

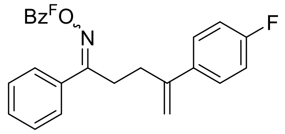

1w
^{1}H NMR
400 MHz, $CDCl_3$

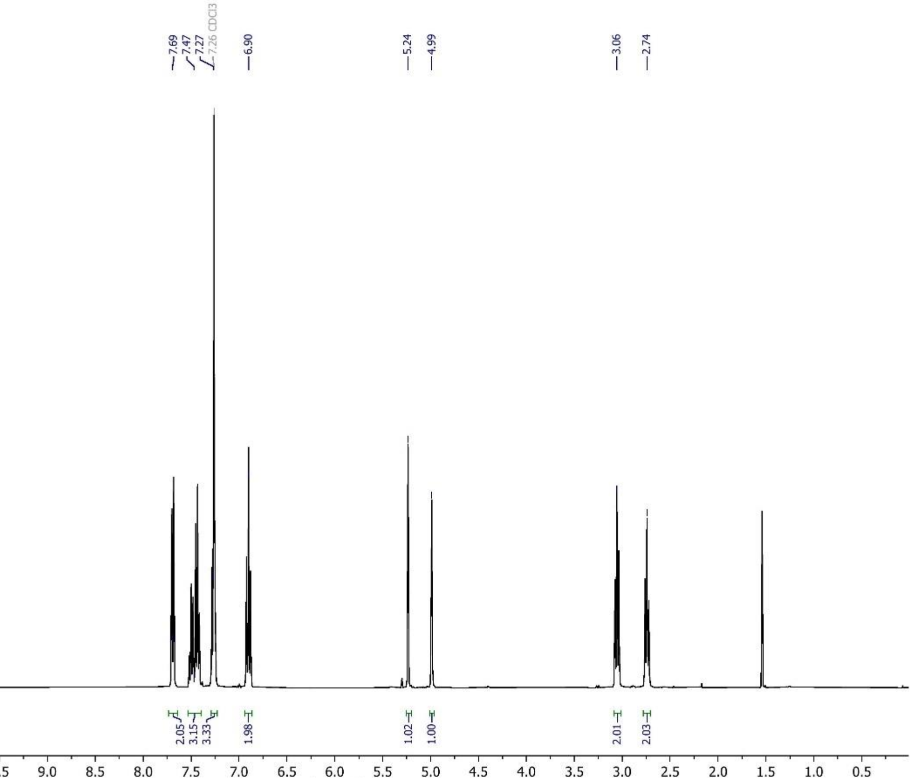

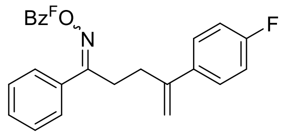

1w
^{13}C NMR
101 MHz, $CDCl_3$

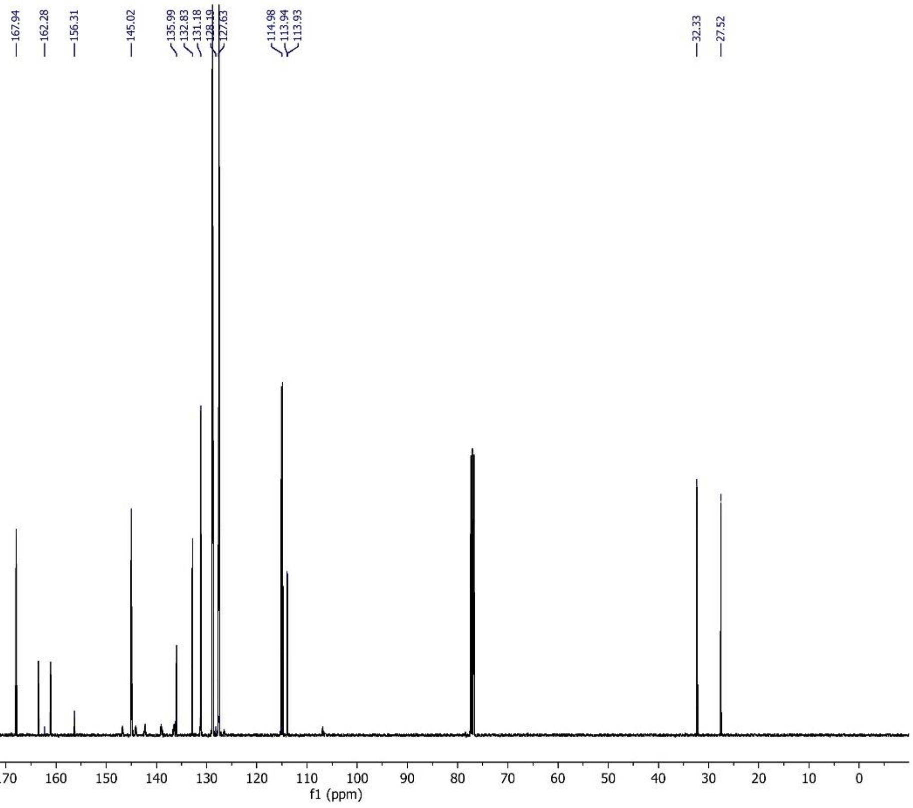

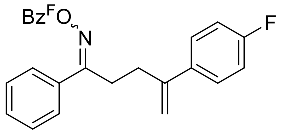

1w
^{19}F NMR
376 MHz, $CDCl_3$

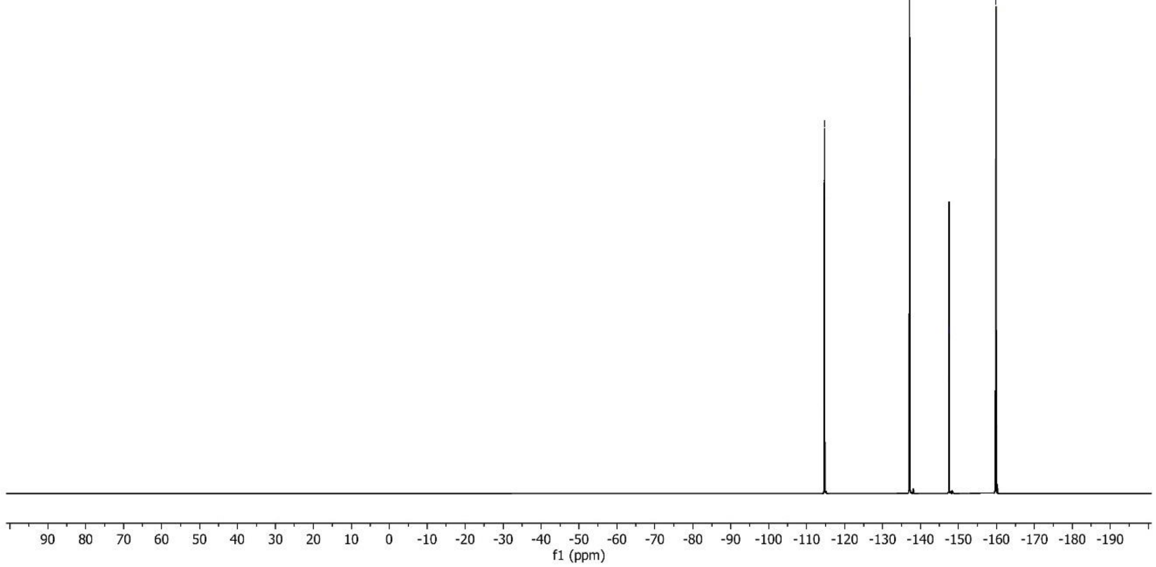

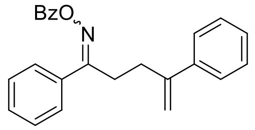

1x
^{1}H NMR
600 MHz, $CDCl_3$

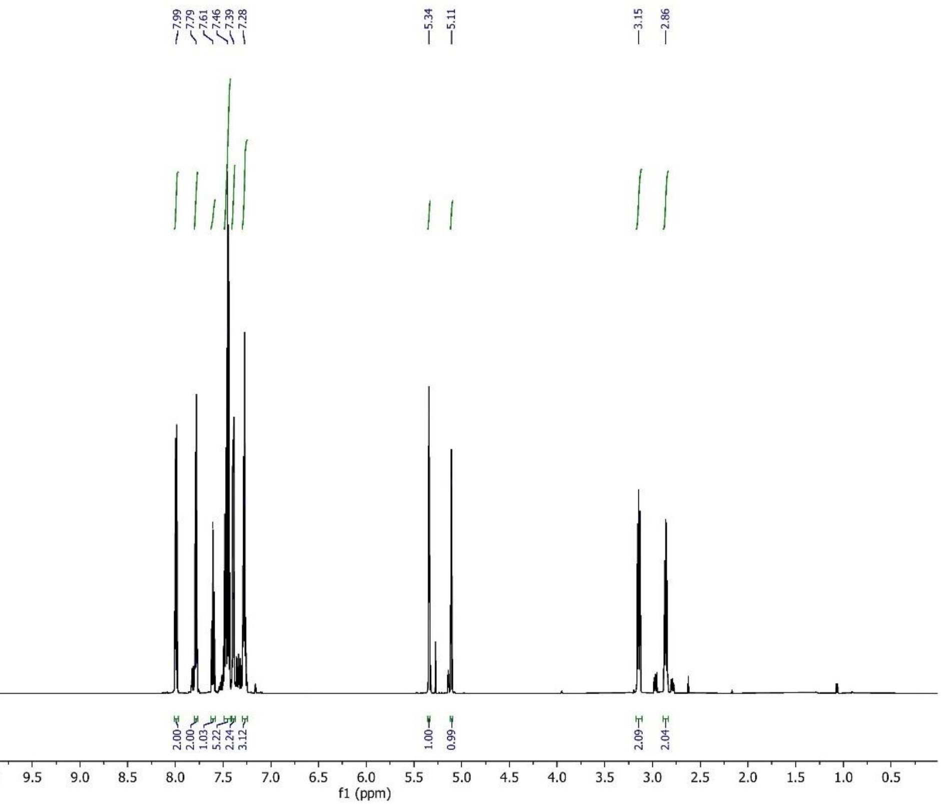

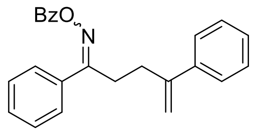

1x
^{13}C NMR
126 MHz, $CDCl_3$

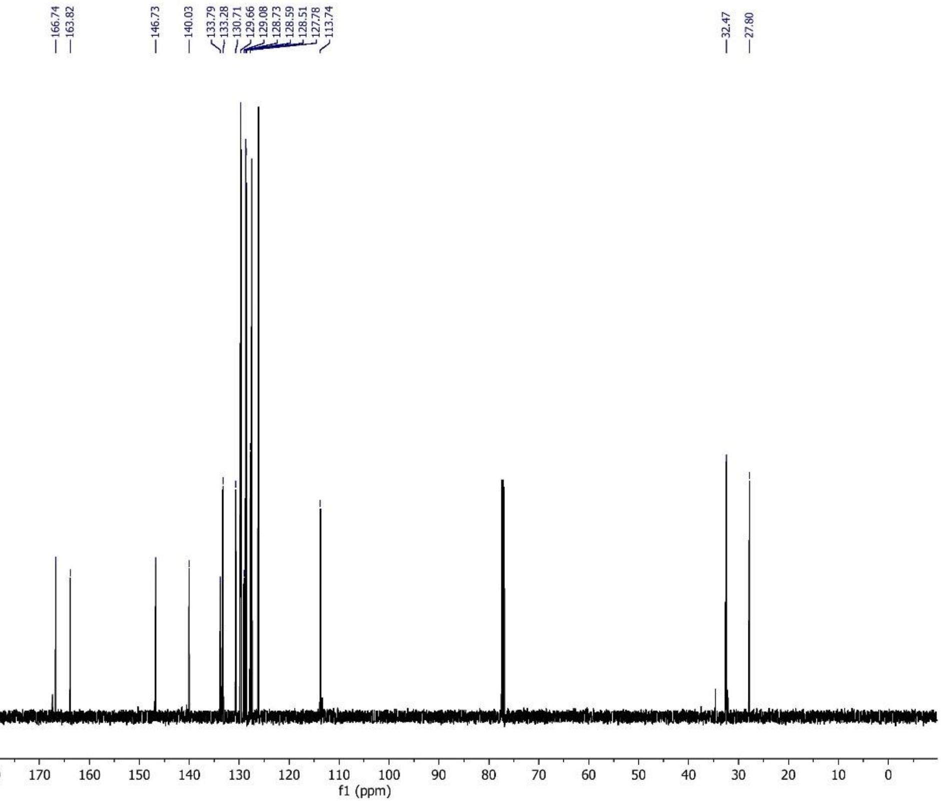

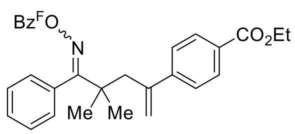

1k, ^{1}H NMR
$CDCl_3$, 400 MHz

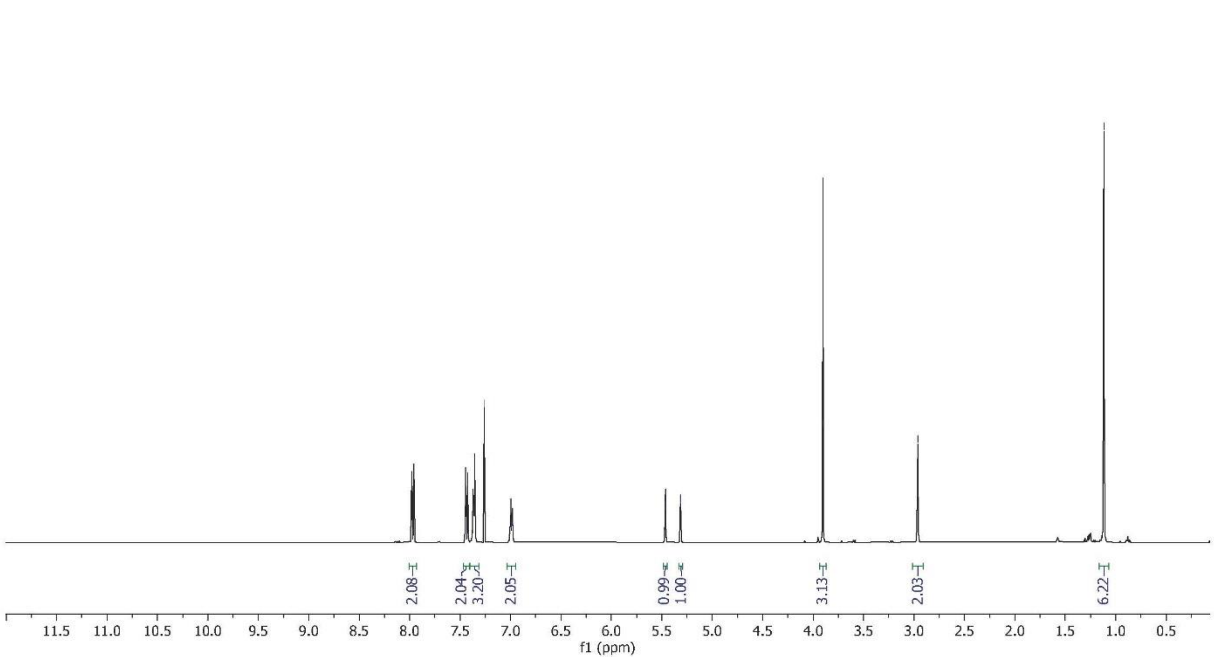

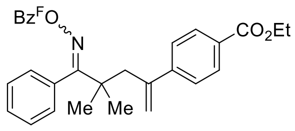

1k, ^{13}C NMR
$CDCl_3$, 101 MHz

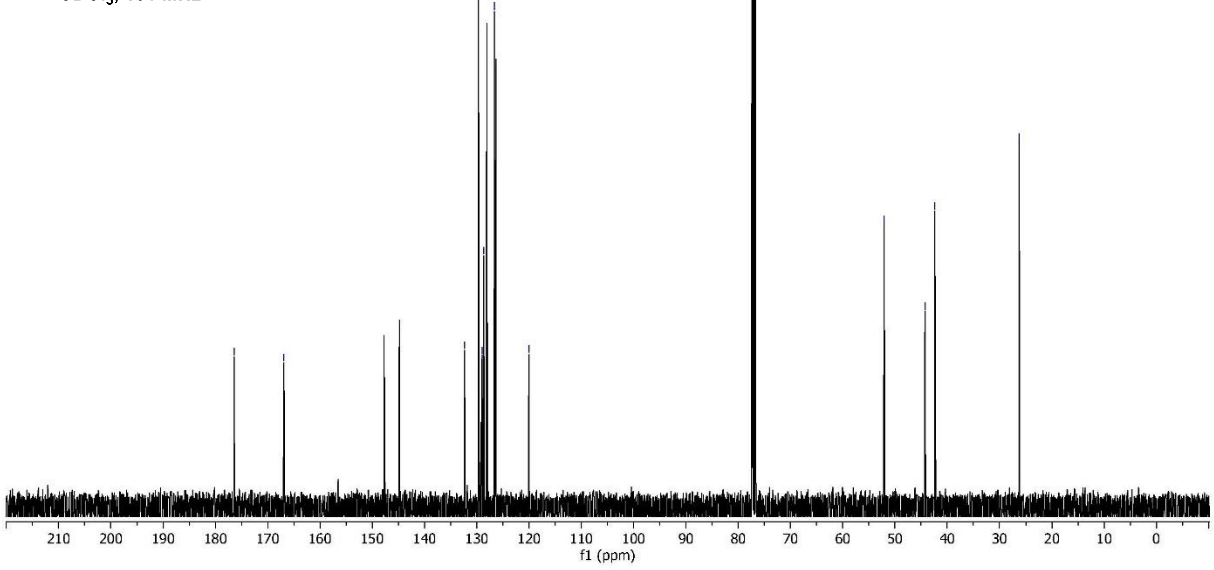

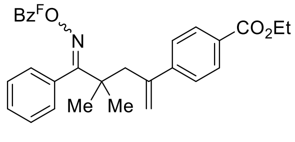

1k, ^{19}F NMR
$CDCl_3$, 470 MHz

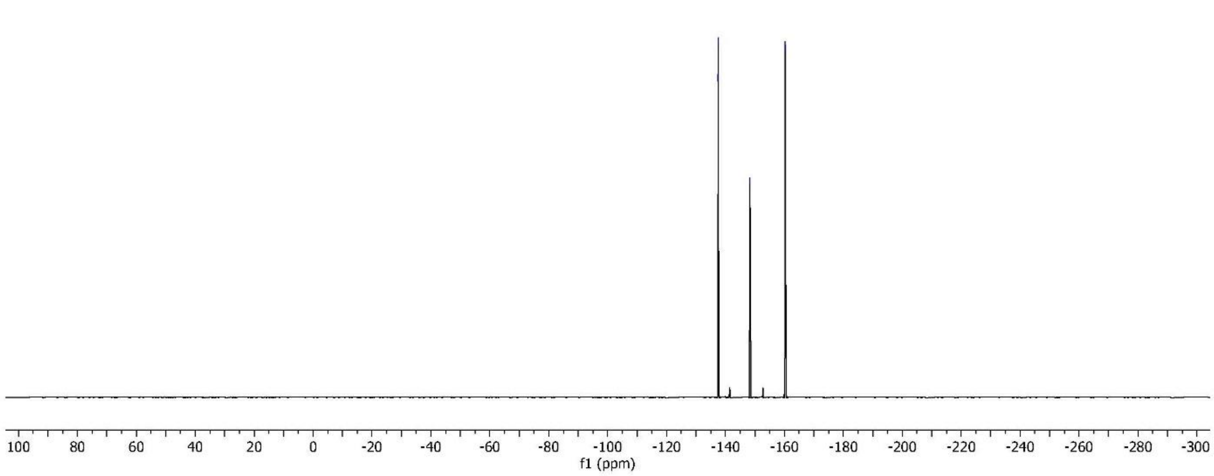

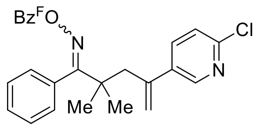

1t, ^{1}H NMR
$CDCl_3$, 500 MHz

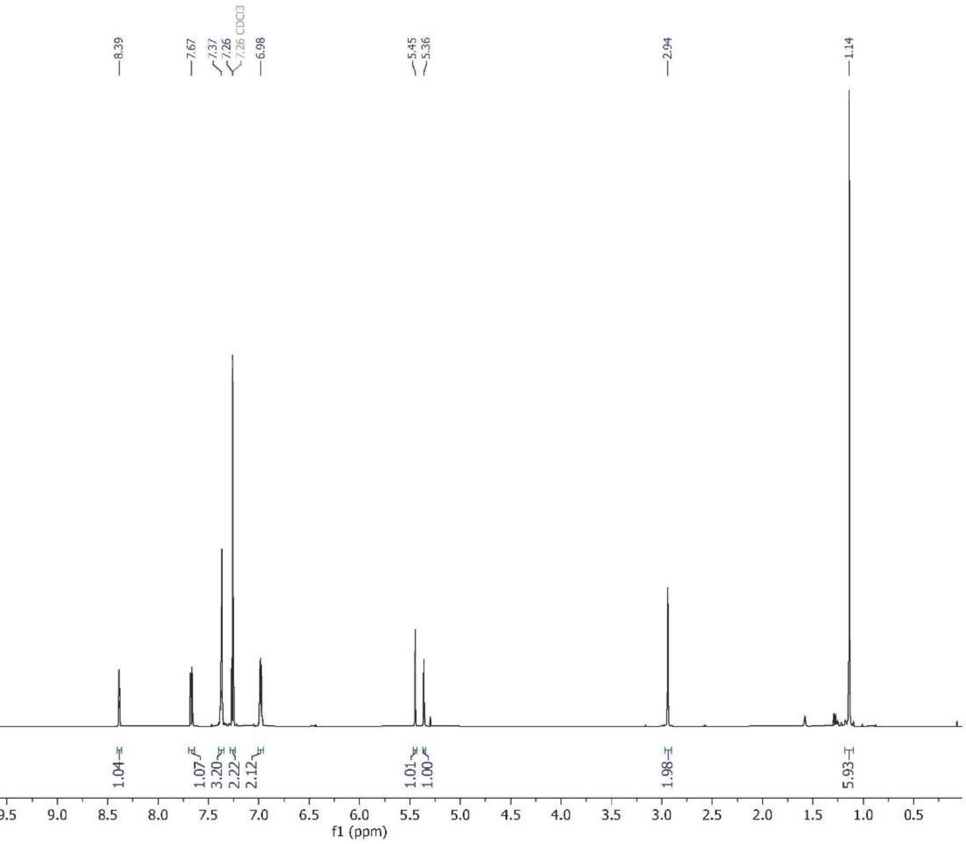

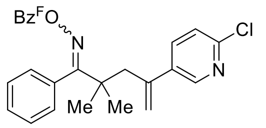

1t, ^{13}C NMR
$CDCl_3$, 126 MHz

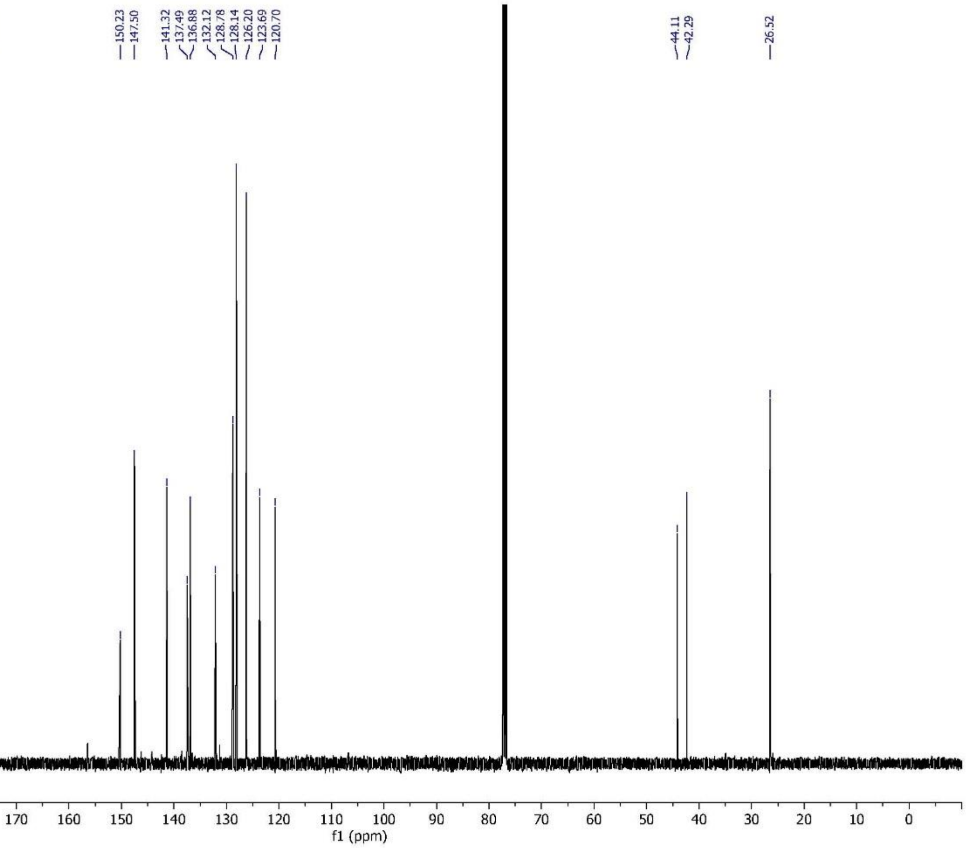

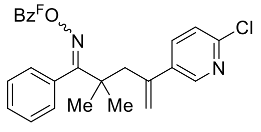

1t, ^{19}F NMR
$CDCl_3$, 470 MHz

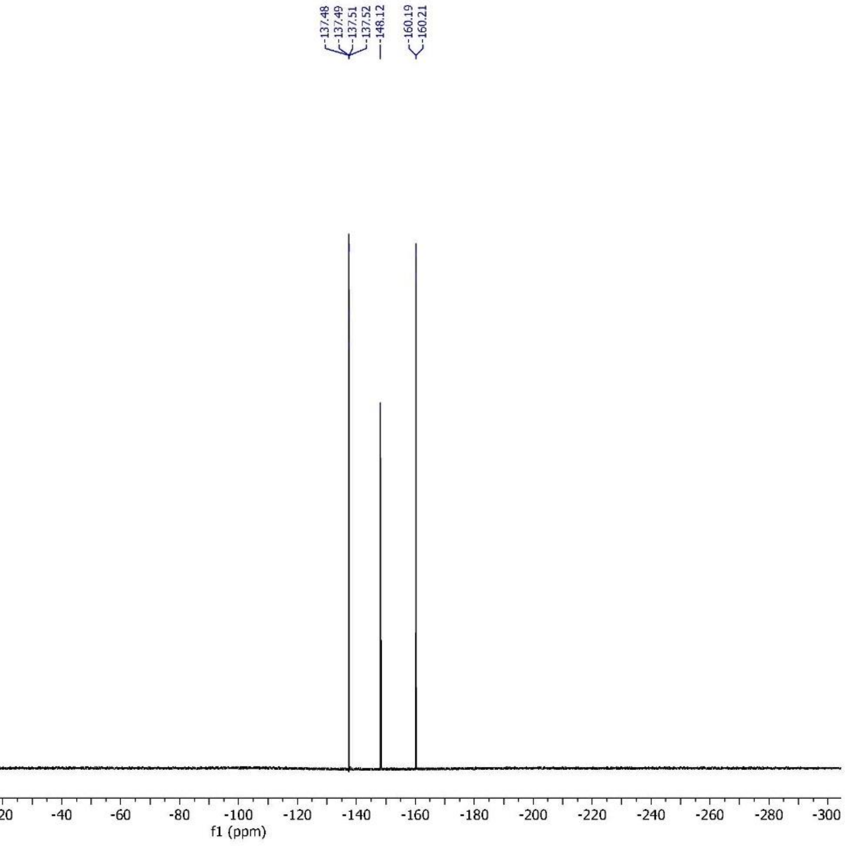

3p Crystallographic Data

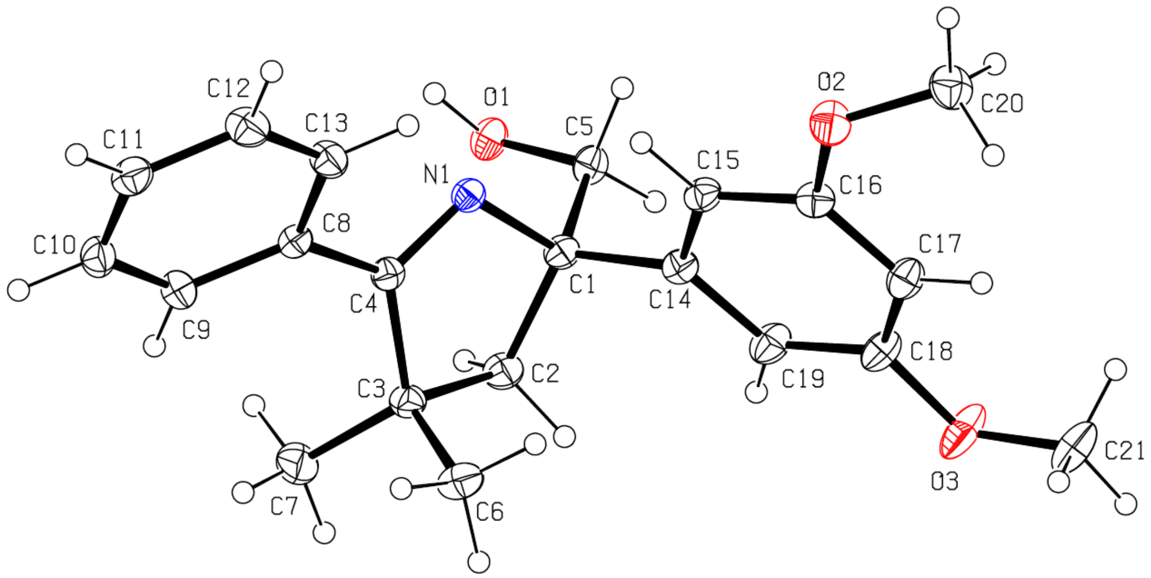

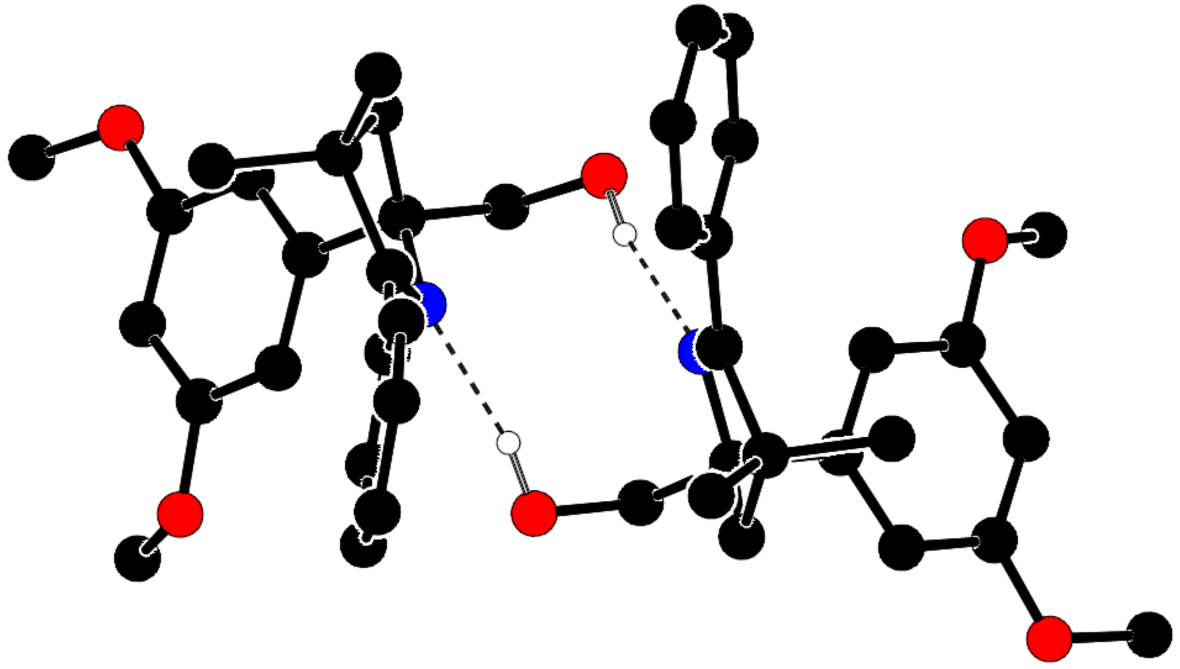

Table S5. Crystal data and structure refinement for d2374_a.

Identification code	d2374_a
Empirical formula	C21 H25 N O3
Formula weight	339.42
Temperature	150(2) K

Wavelength	1.54178 Å	
Crystal system	Monoclinic	
Space group	C2	
Unit cell dimensions	a = 18.9751(13) Å	α= 90°.
	b = 8.7281(6) Å	β= 103.475(3)°.
	c = 11.2702(8) Å	γ = 90°.
Volume	1815.1(2) Å^3	
Z	4	
Density (calculated)	1.242 Mg/m^3	
Absorption coefficient	0.659 mm^{-1}	
F(000)	728	
Crystal size	0.210 x 0.200 x 0.150 mm^3	
Theta range for data collection	4.033 to 66.264°.	
Index ranges	-22<=h<=22, -10<=k<=10, -13<=l<=13	
Reflections collected	20059	
Independent reflections	3086 [R(int) = 0.0316]	
Completeness to theta = 66.264°	98.2 %	
Absorption correction	Semi-empirical from equivalents	
Max. and min. transmission	0.7528 and 0.6744	
Refinement method	Full-matrix least-squares on F^2	
Data / restraints / parameters	3086 / 1 / 235	
Goodness-of-fit on F^2	1.120	
Final R indices [I>2sigma(I)]	R1 = 0.0262, wR2 = 0.0661	
R indices (all data)	R1 = 0.0266, wR2 = 0.0663	
Flack absolute structure parameter	0.04(5)	
Extinction coefficient	0.0040(5)	
Largest diff. peak and hole	0.184 and -0.132 e.Å^{-3}	

Table S6. Atomic coordinates (x 10^4) and equivalent isotropic displacement parameters (Å^{2}x 10^3) for d2374_a. U(eq) is defined as one third of the trace of the orthogonalized U^{ij} tensor.

	x	y	z	U(eq)
O(1)	4123(1)	3843(2)	828(1)	31(1)
O(2)	7572(1)	1378(2)	2618(1)	37(1)
O(3)	6246(1)	940(2)	5800(1)	52(1)
N(1)	5593(1)	5082(2)	1378(1)	24(1)
C(1)	5310(1)	4178(2)	2278(2)	25(1)
C(2)	5016(1)	5389(2)	3021(2)	30(1)
C(3)	5433(1)	6862(2)	2890(2)	27(1)
C(4)	5663(1)	6492(2)	1703(2)	23(1)
C(5)	4718(1)	3084(2)	1585(2)	29(1)
C(6)	6125(1)	7015(3)	3906(2)	37(1)
C(7)	4952(1)	8275(3)	2878(2)	41(1)
C(8)	6041(1)	7564(2)	1028(2)	24(1)
C(9)	5887(1)	9122(2)	900(2)	30(1)
C(10)	6268(1)	10057(2)	278(2)	33(1)
C(11)	6808(1)	9461(2)	-216(2)	34(1)
C(12)	6970(1)	7914(2)	-98(2)	36(1)
C(13)	6588(1)	6975(2)	519(2)	30(1)
C(14)	5918(1)	3161(2)	3014(2)	26(1)
C(15)	6514(1)	2775(2)	2549(2)	27(1)
C(16)	7017(1)	1727(2)	3163(2)	28(1)
C(17)	6959(1)	1086(2)	4267(2)	31(1)
C(18)	6367(1)	1498(3)	4726(2)	34(1)
C(19)	5846(1)	2506(2)	4100(2)	34(1)
C(20)	7927(1)	-60(2)	2895(2)	39(1)
C(21)	6785(1)	-19(3)	6514(2)	49(1)

Table S7. Bond lengths [Å] and angles [°] for d2374_a.

O(1)-C(5)	1.411(2)
O(1)-H(1O)	0.93(3)
O(2)-C(16)	1.372(2)
O(2)-C(20)	1.424(2)
O(3)-C(18)	1.372(2)
O(3)-C(21)	1.418(3)
N(1)-C(4)	1.282(2)
N(1)-C(1)	1.481(2)
C(1)-C(2)	1.533(3)
C(1)-C(14)	1.536(2)
C(1)-C(5)	1.541(2)
C(2)-C(3)	1.535(3)
C(2)-H(2A)	0.9900
C(2)-H(2B)	0.9900
C(3)-C(7)	1.531(3)
C(3)-C(6)	1.534(3)
C(3)-C(4)	1.536(2)
C(4)-C(8)	1.491(2)
C(5)-H(5A)	0.9900
C(5)-H(5B)	0.9900
C(6)-H(6A)	0.9800
C(6)-H(6B)	0.9800
C(6)-H(6C)	0.9800
C(7)-H(7A)	0.9800
C(7)-H(7B)	0.9800
C(7)-H(7C)	0.9800
C(8)-C(9)	1.391(3)
C(8)-C(13)	1.396(3)
C(9)-C(10)	1.384(3)
C(9)-H(9A)	0.9500
C(10)-C(11)	1.377(3)
C(10)-H(10A)	0.9500
C(11)-C(12)	1.384(3)
C(11)-H(11A)	0.9500

C(12)-C(13)	1.385(3)
C(12)-H(12A)	0.9500
C(13)-H(13A)	0.9500
C(14)-C(19)	1.387(3)
C(14)-C(15)	1.393(2)
C(15)-C(16)	1.386(3)
C(15)-H(15A)	0.9500
C(16)-C(17)	1.391(3)
C(17)-C(18)	1.390(3)
C(17)-H(17A)	0.9500
C(18)-C(19)	1.387(3)
C(19)-H(19A)	0.9500
C(20)-H(20A)	0.9800
C(20)-H(20B)	0.9800
C(20)-H(20C)	0.9800
C(21)-H(21A)	0.9800
C(21)-H(21B)	0.9800
C(21)-H(21C)	0.9800

C(5)-O(1)-H(1O)	108.7(17)
C(16)-O(2)-C(20)	118.08(15)
C(18)-O(3)-C(21)	117.62(16)
C(4)-N(1)-C(1)	110.22(15)
N(1)-C(1)-C(2)	104.11(14)
N(1)-C(1)-C(14)	109.33(13)
C(2)-C(1)-C(14)	115.24(15)
N(1)-C(1)-C(5)	108.73(14)
C(2)-C(1)-C(5)	112.89(15)
C(14)-C(1)-C(5)	106.40(14)
C(1)-C(2)-C(3)	105.30(14)
C(1)-C(2)-H(2A)	110.7
C(3)-C(2)-H(2A)	110.7
C(1)-C(2)-H(2B)	110.7
C(3)-C(2)-H(2B)	110.7
H(2A)-C(2)-H(2B)	108.8
C(7)-C(3)-C(6)	109.95(17)

C(7)-C(3)-C(2)	110.89(15)
C(6)-C(3)-C(2)	111.81(16)
C(7)-C(3)-C(4)	116.81(16)
C(6)-C(3)-C(4)	107.50(14)
C(2)-C(3)-C(4)	99.52(14)
N(1)-C(4)-C(8)	119.15(15)
N(1)-C(4)-C(3)	114.78(15)
C(8)-C(4)-C(3)	125.51(15)
O(1)-C(5)-C(1)	113.68(15)
O(1)-C(5)-H(5A)	108.8
C(1)-C(5)-H(5A)	108.8
O(1)-C(5)-H(5B)	108.8
C(1)-C(5)-H(5B)	108.8
H(5A)-C(5)-H(5B)	107.7
C(3)-C(6)-H(6A)	109.5
C(3)-C(6)-H(6B)	109.5
H(6A)-C(6)-H(6B)	109.5
C(3)-C(6)-H(6C)	109.5
H(6A)-C(6)-H(6C)	109.5
H(6B)-C(6)-H(6C)	109.5
C(3)-C(7)-H(7A)	109.5
C(3)-C(7)-H(7B)	109.5
H(7A)-C(7)-H(7B)	109.5
C(3)-C(7)-H(7C)	109.5
H(7A)-C(7)-H(7C)	109.5
H(7B)-C(7)-H(7C)	109.5
C(9)-C(8)-C(13)	118.46(17)
C(9)-C(8)-C(4)	123.49(16)
C(13)-C(8)-C(4)	118.04(16)
C(10)-C(9)-C(8)	120.41(18)
C(10)-C(9)-H(9A)	119.8
C(8)-C(9)-H(9A)	119.8
C(11)-C(10)-C(9)	120.55(18)
C(11)-C(10)-H(10A)	119.7
C(9)-C(10)-H(10A)	119.7
C(10)-C(11)-C(12)	119.93(18)

C(10)-C(11)-H(11A)	120.0
C(12)-C(11)-H(11A)	120.0
C(11)-C(12)-C(13)	119.69(19)
C(11)-C(12)-H(12A)	120.2
C(13)-C(12)-H(12A)	120.2
C(12)-C(13)-C(8)	120.97(18)
C(12)-C(13)-H(13A)	119.5
C(8)-C(13)-H(13A)	119.5
C(19)-C(14)-C(15)	119.17(17)
C(19)-C(14)-C(1)	119.95(16)
C(15)-C(14)-C(1)	120.64(15)
C(16)-C(15)-C(14)	119.82(16)
C(16)-C(15)-H(15A)	120.1
C(14)-C(15)-H(15A)	120.1
O(2)-C(16)-C(15)	115.77(15)
O(2)-C(16)-C(17)	122.71(17)
C(15)-C(16)-C(17)	121.51(16)
C(18)-C(17)-C(16)	117.97(17)
C(18)-C(17)-H(17A)	121.0
C(16)-C(17)-H(17A)	121.0
O(3)-C(18)-C(19)	115.39(17)
O(3)-C(18)-C(17)	123.54(18)
C(19)-C(18)-C(17)	121.07(17)
C(14)-C(19)-C(18)	120.40(17)
C(14)-C(19)-H(19A)	119.8
C(18)-C(19)-H(19A)	119.8
O(2)-C(20)-H(20A)	109.5
O(2)-C(20)-H(20B)	109.5
H(20A)-C(20)-H(20B)	109.5
O(2)-C(20)-H(20C)	109.5
H(20A)-C(20)-H(20C)	109.5
H(20B)-C(20)-H(20C)	109.5
O(3)-C(21)-H(21A)	109.5
O(3)-C(21)-H(21B)	109.5
H(21A)-C(21)-H(21B)	109.5
O(3)-C(21)-H(21C)	109.5

H(21A)-C(21)-H(21C)	109.5
H(21B)-C(21)-H(21C)	109.5

Symmetry transformations used to generate equivalent atoms:

Table S8. Anisotropic displacement parameters ($Å^2$x 10^3) for d2374_a. The anisotropic displacement factor exponent takes the form: $-2\pi^2[h^2 a^{*2}U^{11} + ... + 2 h k a^* b^* U^{12}]$

	U^{11}	U^{22}	U^{33}	U^{23}	U^{13}	U^{12}
O(1)	26(1)	35(1)	31(1)	7(1)	6(1)	-1(1)
O(2)	32(1)	40(1)	42(1)	6(1)	18(1)	7(1)
O(3)	47(1)	80(1)	34(1)	29(1)	16(1)	14(1)
N(1)	23(1)	26(1)	23(1)	1(1)	7(1)	0(1)
C(1)	26(1)	28(1)	24(1)	1(1)	11(1)	-1(1)
C(2)	29(1)	35(1)	29(1)	0(1)	13(1)	0(1)
C(3)	28(1)	29(1)	25(1)	-1(1)	9(1)	2(1)
C(4)	21(1)	26(1)	23(1)	0(1)	4(1)	2(1)
C(5)	28(1)	29(1)	28(1)	4(1)	6(1)	-2(1)
C(6)	39(1)	45(1)	26(1)	-4(1)	7(1)	-7(1)
C(7)	48(1)	39(1)	42(1)	1(1)	22(1)	13(1)
C(8)	23(1)	26(1)	21(1)	1(1)	4(1)	-2(1)
C(9)	31(1)	26(1)	33(1)	0(1)	8(1)	3(1)
C(10)	38(1)	25(1)	33(1)	2(1)	2(1)	-2(1)
C(11)	40(1)	34(1)	29(1)	1(1)	8(1)	-12(1)
C(12)	39(1)	36(1)	39(1)	-4(1)	21(1)	-5(1)
C(13)	33(1)	27(1)	34(1)	-1(1)	14(1)	-1(1)
C(14)	25(1)	28(1)	25(1)	-1(1)	5(1)	-3(1)
C(15)	28(1)	30(1)	24(1)	1(1)	9(1)	-4(1)
C(16)	25(1)	31(1)	28(1)	-2(1)	9(1)	-3(1)
C(17)	26(1)	36(1)	29(1)	5(1)	2(1)	-1(1)
C(18)	34(1)	44(1)	25(1)	8(1)	9(1)	-2(1)
C(19)	30(1)	45(1)	29(1)	6(1)	13(1)	2(1)
C(20)	31(1)	41(1)	44(1)	-1(1)	7(1)	8(1)
C(21)	45(1)	68(2)	31(1)	21(1)	1(1)	0(1)

Table S9. Hydrogen coordinates (x 10^4) and isotropic displacement parameters ($Å^2$x 10^3) for d2374_a.

	x	y	z	U(eq)
H(1O)	4277(15)	4300(30)	190(30)	60(8)
H(2A)	5103	5079	3888	36
H(2B)	4489	5537	2697	36
H(5A)	4935	2383	1078	34
H(5B)	4540	2453	2185	34
H(6A)	5995	7190	4687	55
H(6B)	6412	7881	3726	55
H(6C)	6410	6071	3953	55
H(7A)	4736	8245	3587	61
H(7B)	4567	8279	2128	61
H(7C)	5246	9204	2911	61
H(9A)	5518	9548	1243	36
H(10A)	6155	11118	191	40
H(11A)	7069	10112	-638	41
H(12A)	7341	7499	-440	43
H(13A)	6700	5913	598	36
H(15A)	6575	3230	1814	32
H(17A)	7313	389	4693	37
H(19A)	5438	2750	4418	40
H(20A)	8219	-276	2303	58
H(20B)	7564	-869	2855	58
H(20C)	8242	-26	3718	58
H(21A)	6622	-383	7228	74
H(21B)	7236	562	6784	74
H(21C)	6870	-897	6025	74

Table S10. Torsion angles [°] for d2374_a.

C(4)-N(1)-C(1)-C(2)	15.76(19)
C(4)-N(1)-C(1)-C(14)	-107.88(16)
C(4)-N(1)-C(1)-C(5)	136.34(15)
N(1)-C(1)-C(2)-C(3)	-24.12(18)
C(14)-C(1)-C(2)-C(3)	95.60(17)
C(5)-C(1)-C(2)-C(3)	-141.86(15)
C(1)-C(2)-C(3)-C(7)	146.09(16)
C(1)-C(2)-C(3)-C(6)	-90.81(18)
C(1)-C(2)-C(3)-C(4)	22.49(17)
C(1)-N(1)-C(4)-C(8)	171.11(14)
C(1)-N(1)-C(4)-C(3)	-0.7(2)
C(7)-C(3)-C(4)-N(1)	-133.68(18)
C(6)-C(3)-C(4)-N(1)	102.26(19)
C(2)-C(3)-C(4)-N(1)	-14.35(19)
C(7)-C(3)-C(4)-C(8)	55.1(2)
C(6)-C(3)-C(4)-C(8)	-69.0(2)
C(2)-C(3)-C(4)-C(8)	174.40(16)
N(1)-C(1)-C(5)-O(1)	-60.57(19)
C(2)-C(1)-C(5)-O(1)	54.4(2)
C(14)-C(1)-C(5)-O(1)	-178.22(14)
N(1)-C(4)-C(8)-C(9)	150.63(18)
C(3)-C(4)-C(8)-C(9)	-38.5(3)
N(1)-C(4)-C(8)-C(13)	-30.7(2)
C(3)-C(4)-C(8)-C(13)	140.23(18)
C(13)-C(8)-C(9)-C(10)	0.2(3)
C(4)-C(8)-C(9)-C(10)	178.94(17)
C(8)-C(9)-C(10)-C(11)	-0.5(3)
C(9)-C(10)-C(11)-C(12)	0.4(3)
C(10)-C(11)-C(12)-C(13)	-0.2(3)
C(11)-C(12)-C(13)-C(8)	-0.1(3)
C(9)-C(8)-C(13)-C(12)	0.0(3)
C(4)-C(8)-C(13)-C(12)	-178.74(17)
N(1)-C(1)-C(14)-C(19)	163.75(17)
C(2)-C(1)-C(14)-C(19)	46.9(2)

C(5)-C(1)-C(14)-C(19)	-79.0(2)
N(1)-C(1)-C(14)-C(15)	-22.0(2)
C(2)-C(1)-C(14)-C(15)	-138.77(17)
C(5)-C(1)-C(14)-C(15)	95.29(19)
C(19)-C(14)-C(15)-C(16)	1.2(3)
C(1)-C(14)-C(15)-C(16)	-173.10(16)
C(20)-O(2)-C(16)-C(15)	-155.39(17)
C(20)-O(2)-C(16)-C(17)	25.0(3)
C(14)-C(15)-C(16)-O(2)	177.86(16)
C(14)-C(15)-C(16)-C(17)	-2.6(3)
O(2)-C(16)-C(17)-C(18)	-178.78(18)
C(15)-C(16)-C(17)-C(18)	1.7(3)
C(21)-O(3)-C(18)-C(19)	-176.1(2)
C(21)-O(3)-C(18)-C(17)	4.8(3)
C(16)-C(17)-C(18)-O(3)	179.65(19)
C(16)-C(17)-C(18)-C(19)	0.5(3)
C(15)-C(14)-C(19)-C(18)	0.9(3)
C(1)-C(14)-C(19)-C(18)	175.30(18)
O(3)-C(18)-C(19)-C(14)	178.99(19)
C(17)-C(18)-C(19)-C(14)	-1.8(3)

Symmetry transformations used to generate equivalent atoms:

Table S11. Hydrogen bonds for d2374_a [Å and °].

D-H...A	d(D-H)	d(H...A)	d(D...A)	<(DHA)
O(1)-H(1O)...N(1)#1	0.93(3)	1.96(3)	2.874(2)	167(3)

Symmetry transformations used to generate equivalent atoms:

#1 -x+1,y,-z

3s-Ni Crystallographic Data

Note: Two co-crystallized methanol molecules omitted in the ORTEP diagram for clarity.

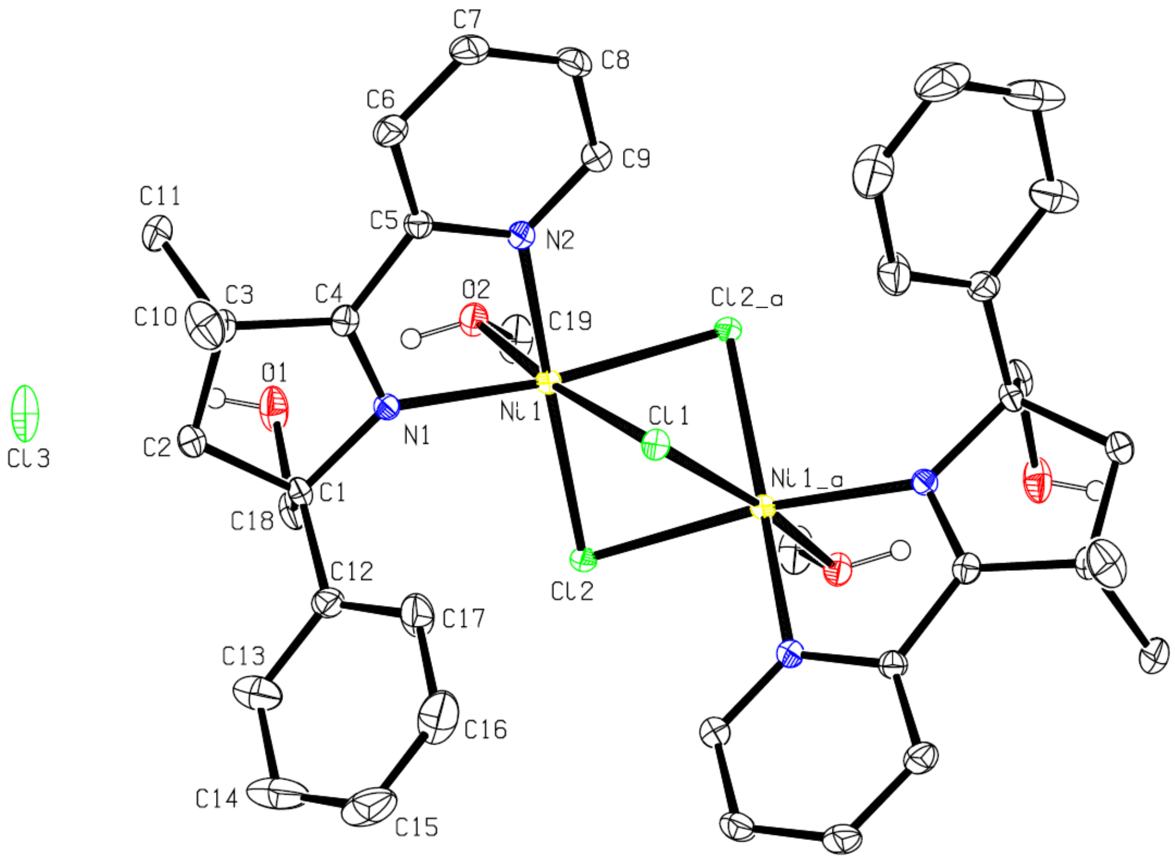

Table S12. Crystal data and structure refinement for d2421_a.

Identification code	d2421_a
Empirical formula	C40 H56 Cl4 N4 Ni2 O6
Formula weight	948.10

Temperature	150(2) K	
Wavelength	1.54178 Å	
Crystal system	Monoclinic	
Space group	C2	
Unit cell dimensions	a = 18.5704(9) Å	α= 90°.
	b = 7.9283(4) Å	β= 92.805(2)°.
	c = 15.2674(8) Å	γ = 90°.
Volume	2245.2(2) $Å^3$	
Z	2	
Density (calculated)	1.402 Mg/m^3	
Absorption coefficient	3.619 mm^{-1}	
F(000)	992	
Crystal size	0.220 x 0.180 x 0.040 mm^3	
Theta range for data collection	5.460 to 66.150°.	
Index ranges	-21<=h<=21, -9<=k<=9, -17<=l<=17	
Reflections collected	15717	
Independent reflections	3787 [R(int) = 0.0291]	
Completeness to theta = 66.150°	99.0 %	
Absorption correction	Semi-empirical from equivalents	
Max. and min. transmission	0.7528 and 0.5894	
Refinement method	Full-matrix least-squares on F^2	
Data / restraints / parameters	3787 / 1 / 266	
Goodness-of-fit on F^2	1.060	
Final R indices [I>2sigma(I)]	R1 = 0.0287, wR2 = 0.0798	
R indices (all data)	R1 = 0.0290, wR2 = 0.0800	
Flack absolute structure parameter	0.10(2)	
Extinction coefficient	n/a	
Largest diff. peak and hole	0.856 and -0.356 e.$Å^{-3}$	

Table S13. Atomic coordinates (x 10^4) and equivalent isotropic displacement parameters (Å^{2}x 10^3) for d2421_a. U(eq) is defined as one third of the trace of the orthogonalized U^{ij} tensor.

	x	y	z	U(eq)
Ni(1)	5373(1)	5316(1)	4148(1)	17(1)
Cl(1)	5000	7739(1)	5000	20(1)
Cl(2)	4230(1)	4148(1)	4428(1)	20(1)
O(1)	5109(2)	3161(4)	1973(2)	35(1)
O(2)	5754(1)	3112(3)	3557(2)	24(1)
N(1)	5160(2)	6231(4)	2867(2)	18(1)
N(2)	6317(2)	6539(4)	3933(2)	21(1)
C(1)	4576(2)	5933(5)	2181(2)	22(1)
C(2)	4874(2)	6642(5)	1323(2)	26(1)
C(3)	5631(2)	7359(5)	1569(2)	25(1)
C(4)	5707(2)	6922(4)	2534(2)	20(1)
C(5)	6356(2)	7222(4)	3120(2)	21(1)
C(6)	6954(2)	8160(5)	2898(3)	30(1)
C(7)	7507(2)	8411(6)	3523(3)	35(1)
C(8)	7459(2)	7736(6)	4349(3)	32(1)
C(9)	6854(2)	6808(5)	4531(3)	26(1)
C(10)	5630(3)	9273(6)	1426(3)	42(1)
C(11)	6214(2)	6492(7)	1048(3)	41(1)
C(12)	3867(2)	6789(5)	2402(3)	28(1)
C(13)	3256(2)	6516(7)	1844(4)	45(1)
C(14)	2610(3)	7260(8)	2005(5)	63(2)
C(15)	2552(3)	8279(9)	2728(4)	65(2)
C(16)	3157(3)	8604(9)	3275(4)	67(2)
C(17)	3813(3)	7840(7)	3109(3)	45(1)
C(18)	4454(2)	4019(5)	2122(2)	27(1)
C(19)	5641(3)	1444(5)	3887(3)	37(1)
Cl(3)	5000	2638(2)	0	43(1)
O(1S)	3306(4)	2268(12)	199(6)	140(3)
C(1S)	3216(5)	676(13)	584(8)	124(4)

Table S14. Bond lengths [Å] and angles [°] for d2421_a.

Ni(1)-N(2)	2.044(3)
Ni(1)-O(2)	2.105(3)
Ni(1)-N(1)	2.105(3)
Ni(1)-Cl(2)	2.3739(9)
Ni(1)-Cl(1)	2.4392(10)
Ni(1)-Cl(2)#1	2.4434(9)
O(1)-C(18)	1.421(5)
O(1)-H(1O)	0.73(6)
O(2)-C(19)	1.434(5)
O(2)-H(2O)	0.79(6)
N(1)-C(4)	1.282(5)
N(1)-C(1)	1.489(5)
N(2)-C(9)	1.337(5)
N(2)-C(5)	1.359(5)
C(1)-C(12)	1.534(5)
C(1)-C(18)	1.536(6)
C(1)-C(2)	1.552(5)
C(2)-C(3)	1.546(5)
C(2)-H(2A)	0.9900
C(2)-H(2B)	0.9900
C(3)-C(4)	1.512(5)
C(3)-C(10)	1.533(6)
C(3)-C(11)	1.536(6)
C(4)-C(5)	1.485(5)
C(5)-C(6)	1.393(5)
C(6)-C(7)	1.382(6)
C(6)-H(6A)	0.9500
C(7)-C(8)	1.378(6)
C(7)-H(7A)	0.9500
C(8)-C(9)	1.382(6)
C(8)-H(8A)	0.9500
C(9)-H(9A)	0.9500
C(10)-H(10A)	0.9800
C(10)-H(10B)	0.9800

C(10)-H(10C)	0.9800
C(11)-H(11A)	0.9800
C(11)-H(11B)	0.9800
C(11)-H(11C)	0.9800
C(12)-C(17)	1.370(7)
C(12)-C(13)	1.402(6)
C(13)-C(14)	1.370(7)
C(13)-H(13A)	0.9500
C(14)-C(15)	1.376(10)
C(14)-H(14A)	0.9500
C(15)-C(16)	1.390(10)
C(15)-H(15A)	0.9500
C(16)-C(17)	1.395(7)
C(16)-H(16A)	0.9500
C(17)-H(17A)	0.9500
C(18)-H(18A)	0.9900
C(18)-H(18B)	0.9900
C(19)-H(19A)	0.9800
C(19)-H(19B)	0.9800
C(19)-H(19C)	0.9800
O(1S)-C(1S)	1.406(12)
O(1S)-H(1S)	0.8400
C(1S)-H(1S1)	0.9800
C(1S)-H(1S2)	0.9800
C(1S)-H(1S3)	0.9800

N(2)-Ni(1)-O(2)	90.93(12)
N(2)-Ni(1)-N(1)	79.12(12)
O(2)-Ni(1)-N(1)	86.55(11)
N(2)-Ni(1)-Cl(2)	174.61(9)
O(2)-Ni(1)-Cl(2)	94.30(8)
N(1)-Ni(1)-Cl(2)	99.88(8)
N(2)-Ni(1)-Cl(1)	88.89(9)
O(2)-Ni(1)-Cl(1)	173.02(8)
N(1)-Ni(1)-Cl(1)	100.26(8)
Cl(2)-Ni(1)-Cl(1)	86.08(3)

N(2)-Ni(1)-Cl(2)#1	95.73(9)
O(2)-Ni(1)-Cl(2)#1	88.50(8)
N(1)-Ni(1)-Cl(2)#1	172.79(8)
Cl(2)-Ni(1)-Cl(2)#1	85.72(3)
Cl(1)-Ni(1)-Cl(2)#1	84.58(3)
Ni(1)-Cl(1)-Ni(1)#1	76.09(4)
Ni(1)-Cl(2)-Ni(1)#1	77.22(3)
C(18)-O(1)-H(1O)	114(5)
C(19)-O(2)-Ni(1)	123.8(2)
C(19)-O(2)-H(2O)	108(4)
Ni(1)-O(2)-H(2O)	107(4)
C(4)-N(1)-C(1)	110.6(3)
C(4)-N(1)-Ni(1)	113.5(2)
C(1)-N(1)-Ni(1)	134.2(2)
C(9)-N(2)-C(5)	119.3(3)
C(9)-N(2)-Ni(1)	125.6(2)
C(5)-N(2)-Ni(1)	114.8(2)
N(1)-C(1)-C(12)	112.2(3)
N(1)-C(1)-C(18)	107.3(3)
C(12)-C(1)-C(18)	108.9(3)
N(1)-C(1)-C(2)	105.0(3)
C(12)-C(1)-C(2)	111.8(3)
C(18)-C(1)-C(2)	111.5(3)
C(3)-C(2)-C(1)	106.6(3)
C(3)-C(2)-H(2A)	110.4
C(1)-C(2)-H(2A)	110.4
C(3)-C(2)-H(2B)	110.4
C(1)-C(2)-H(2B)	110.4
H(2A)-C(2)-H(2B)	108.6
C(4)-C(3)-C(10)	111.5(3)
C(4)-C(3)-C(11)	111.6(3)
C(10)-C(3)-C(11)	111.4(4)
C(4)-C(3)-C(2)	101.1(3)
C(10)-C(3)-C(2)	109.5(3)
C(11)-C(3)-C(2)	111.4(3)
N(1)-C(4)-C(5)	117.7(3)

N(1)-C(4)-C(3)	116.6(3)
C(5)-C(4)-C(3)	125.7(3)
N(2)-C(5)-C(6)	120.9(3)
N(2)-C(5)-C(4)	114.2(3)
C(6)-C(5)-C(4)	124.9(3)
C(7)-C(6)-C(5)	118.9(4)
C(7)-C(6)-H(6A)	120.5
C(5)-C(6)-H(6A)	120.5
C(8)-C(7)-C(6)	119.8(4)
C(8)-C(7)-H(7A)	120.1
C(6)-C(7)-H(7A)	120.1
C(7)-C(8)-C(9)	118.7(4)
C(7)-C(8)-H(8A)	120.6
C(9)-C(8)-H(8A)	120.6
N(2)-C(9)-C(8)	122.3(4)
N(2)-C(9)-H(9A)	118.9
C(8)-C(9)-H(9A)	118.9
C(3)-C(10)-H(10A)	109.5
C(3)-C(10)-H(10B)	109.5
H(10A)-C(10)-H(10B)	109.5
C(3)-C(10)-H(10C)	109.5
H(10A)-C(10)-H(10C)	109.5
H(10B)-C(10)-H(10C)	109.5
C(3)-C(11)-H(11A)	109.5
C(3)-C(11)-H(11B)	109.5
H(11A)-C(11)-H(11B)	109.5
C(3)-C(11)-H(11C)	109.5
H(11A)-C(11)-H(11C)	109.5
H(11B)-C(11)-H(11C)	109.5
C(17)-C(12)-C(13)	119.0(4)
C(17)-C(12)-C(1)	122.6(4)
C(13)-C(12)-C(1)	118.4(4)
C(14)-C(13)-C(12)	120.9(5)
C(14)-C(13)-H(13A)	119.5
C(12)-C(13)-H(13A)	119.5
C(13)-C(14)-C(15)	120.0(6)

C(13)-C(14)-H(14A)	120.0
C(15)-C(14)-H(14A)	120.0
C(14)-C(15)-C(16)	119.9(5)
C(14)-C(15)-H(15A)	120.1
C(16)-C(15)-H(15A)	120.1
C(15)-C(16)-C(17)	119.8(6)
C(15)-C(16)-H(16A)	120.1
C(17)-C(16)-H(16A)	120.1
C(12)-C(17)-C(16)	120.4(5)
C(12)-C(17)-H(17A)	119.8
C(16)-C(17)-H(17A)	119.8
O(1)-C(18)-C(1)	110.9(3)
O(1)-C(18)-H(18A)	109.4
C(1)-C(18)-H(18A)	109.4
O(1)-C(18)-H(18B)	109.4
C(1)-C(18)-H(18B)	109.4
H(18A)-C(18)-H(18B)	108.0
O(2)-C(19)-H(19A)	109.5
O(2)-C(19)-H(19B)	109.5
H(19A)-C(19)-H(19B)	109.5
O(2)-C(19)-H(19C)	109.5
H(19A)-C(19)-H(19C)	109.5
H(19B)-C(19)-H(19C)	109.5
C(1S)-O(1S)-H(1S)	109.5
O(1S)-C(1S)-H(1S1)	109.5
O(1S)-C(1S)-H(1S2)	109.5
H(1S1)-C(1S)-H(1S2)	109.5
O(1S)-C(1S)-H(1S3)	109.5
H(1S1)-C(1S)-H(1S3)	109.5
H(1S2)-C(1S)-H(1S3)	109.5

Symmetry transformations used to generate equivalent atoms:

#1 -x+1,y,-z+1

Table S15. Anisotropic displacement parameters ($Å^2 x 10^3$) for d2421_a. The anisotropic displacement factor exponent takes the form: $-2\pi^2[h^2 a^{*2}U^{11} + ... + 2 h k a^* b^* U^{12}]$

	U^{11}	U^{22}	U^{33}	U^{23}	U^{13}	U^{12}
Ni(1)	14(1)	20(1)	16(1)	0(1)	1(1)	-1(1)
Cl(1)	24(1)	18(1)	19(1)	0	2(1)	0
Cl(2)	15(1)	25(1)	18(1)	-2(1)	1(1)	-3(1)
O(1)	53(2)	30(2)	20(2)	-4(1)	-1(1)	10(1)
O(2)	28(1)	24(1)	20(2)	0(1)	2(1)	4(1)
N(1)	17(1)	20(2)	17(2)	-1(1)	0(1)	1(1)
N(2)	20(1)	23(2)	19(2)	1(1)	1(1)	-1(1)
C(1)	22(2)	28(2)	15(2)	1(1)	-4(1)	0(1)
C(2)	26(2)	30(2)	21(2)	5(2)	-1(2)	2(2)
C(3)	28(2)	28(2)	18(2)	3(2)	2(2)	1(2)
C(4)	24(2)	16(2)	20(2)	-2(1)	3(1)	3(1)
C(5)	20(2)	21(2)	22(2)	-1(1)	1(1)	0(1)
C(6)	26(2)	35(2)	30(2)	7(2)	6(2)	-4(2)
C(7)	22(2)	44(2)	40(3)	5(2)	3(2)	-10(2)
C(8)	21(2)	40(2)	33(2)	2(2)	-5(2)	-6(2)
C(9)	24(2)	33(2)	23(2)	1(2)	0(2)	-1(2)
C(10)	57(3)	30(2)	39(2)	15(2)	-11(2)	-7(2)
C(11)	33(2)	68(3)	22(2)	-4(2)	6(2)	9(2)
C(12)	23(2)	33(2)	27(2)	7(2)	3(2)	6(2)
C(13)	29(2)	45(3)	61(3)	8(2)	-5(2)	-3(2)
C(14)	24(2)	63(4)	100(5)	23(3)	-2(3)	4(2)
C(15)	40(3)	80(4)	78(4)	37(4)	22(3)	32(3)
C(16)	74(4)	87(5)	42(3)	15(3)	13(3)	55(4)
C(17)	48(3)	58(3)	28(2)	-2(2)	-2(2)	29(2)
C(18)	35(2)	27(2)	19(2)	-1(2)	-6(2)	-5(2)
C(19)	60(3)	22(2)	30(2)	0(2)	5(2)	7(2)
Cl(3)	80(1)	30(1)	18(1)	0	0(1)	0
O(1S)	112(5)	143(7)	164(7)	36(6)	-13(5)	19(5)
C(1S)	99(6)	97(7)	184(11)	44(7)	79(7)	26(5)

Table S16. Hydrogen coordinates (x 10^4) and isotropic displacement parameters ($Å^2$x 10^3) for d2421_a.

	x	y	z	U(eq)
H(1O)	5160(30)	2980(80)	1510(40)	46(17)
H(2O)	5610(30)	3140(70)	3060(40)	35(14)
H(2A)	4906	5736	880	31
H(2B)	4555	7542	1078	31
H(6A)	6981	8621	2326	36
H(7A)	7920	9047	3383	43
H(8A)	7835	7905	4786	38
H(9A)	6819	6346	5102	32
H(10A)	5293	9802	1817	64
H(10B)	5479	9521	815	64
H(10C)	6116	9719	1553	64
H(11A)	6684	6998	1204	61
H(11B)	6102	6638	419	61
H(11C)	6227	5286	1190	61
H(13A)	3291	5805	1347	54
H(14A)	2202	7073	1618	75
H(15A)	2100	8760	2853	78
H(16A)	3124	9343	3761	80
H(17A)	4225	8049	3487	54
H(18A)	4258	3610	2674	33
H(18B)	4097	3769	1637	33
H(19A)	5133	1133	3787	55
H(19B)	5769	1416	4517	55
H(19C)	5944	644	3583	55
H(1S)	3745	2423	112	210
H(1S1)	3665	32	560	186
H(1S2)	2827	67	264	186
H(1S3)	3094	819	1197	186

Table S17. Torsion angles [°] for d2421_a.

C(4)-N(1)-C(1)-C(12)	-123.0(3)
Ni(1)-N(1)-C(1)-C(12)	73.4(4)
C(4)-N(1)-C(1)-C(18)	117.4(3)
Ni(1)-N(1)-C(1)-C(18)	-46.3(4)
C(4)-N(1)-C(1)-C(2)	-1.4(4)
Ni(1)-N(1)-C(1)-C(2)	-165.0(3)
N(1)-C(1)-C(2)-C(3)	-0.9(4)
C(12)-C(1)-C(2)-C(3)	121.0(3)
C(18)-C(1)-C(2)-C(3)	-116.8(3)
C(1)-C(2)-C(3)-C(4)	2.4(4)
C(1)-C(2)-C(3)-C(10)	-115.3(4)
C(1)-C(2)-C(3)-C(11)	121.0(4)
C(1)-N(1)-C(4)-C(5)	-176.7(3)
Ni(1)-N(1)-C(4)-C(5)	-9.4(4)
C(1)-N(1)-C(4)-C(3)	3.3(4)
Ni(1)-N(1)-C(4)-C(3)	170.6(2)
C(10)-C(3)-C(4)-N(1)	112.6(4)
C(11)-C(3)-C(4)-N(1)	-122.1(4)
C(2)-C(3)-C(4)-N(1)	-3.7(4)
C(10)-C(3)-C(4)-C(5)	-67.4(5)
C(11)-C(3)-C(4)-C(5)	57.9(5)
C(2)-C(3)-C(4)-C(5)	176.3(3)
C(9)-N(2)-C(5)-C(6)	1.6(5)
Ni(1)-N(2)-C(5)-C(6)	176.9(3)
C(9)-N(2)-C(5)-C(4)	-176.4(3)
Ni(1)-N(2)-C(5)-C(4)	-1.1(4)
N(1)-C(4)-C(5)-N(2)	7.2(5)
C(3)-C(4)-C(5)-N(2)	-172.8(3)
N(1)-C(4)-C(5)-C(6)	-170.7(4)
C(3)-C(4)-C(5)-C(6)	9.3(6)
N(2)-C(5)-C(6)-C(7)	-0.9(6)
C(4)-C(5)-C(6)-C(7)	176.8(4)
C(5)-C(6)-C(7)-C(8)	-0.1(6)
C(6)-C(7)-C(8)-C(9)	0.4(6)

C(5)-N(2)-C(9)-C(8)	-1.3(6)
Ni(1)-N(2)-C(9)-C(8)	-176.0(3)
C(7)-C(8)-C(9)-N(2)	0.3(6)
N(1)-C(1)-C(12)-C(17)	6.2(6)
C(18)-C(1)-C(12)-C(17)	124.9(5)
C(2)-C(1)-C(12)-C(17)	-111.5(5)
N(1)-C(1)-C(12)-C(13)	-175.5(4)
C(18)-C(1)-C(12)-C(13)	-56.9(5)
C(2)-C(1)-C(12)-C(13)	66.8(5)
C(17)-C(12)-C(13)-C(14)	-1.1(7)
C(1)-C(12)-C(13)-C(14)	-179.4(4)
C(12)-C(13)-C(14)-C(15)	-0.6(8)
C(13)-C(14)-C(15)-C(16)	2.5(9)
C(14)-C(15)-C(16)-C(17)	-2.6(9)
C(13)-C(12)-C(17)-C(16)	0.9(8)
C(1)-C(12)-C(17)-C(16)	179.2(5)
C(15)-C(16)-C(17)-C(12)	0.9(9)
N(1)-C(1)-C(18)-O(1)	-54.6(4)
C(12)-C(1)-C(18)-O(1)	-176.2(3)
C(2)-C(1)-C(18)-O(1)	59.9(4)

Symmetry transformations used to generate equivalent atoms:

#1 -x+1,y,-z+1

Table S18. Hydrogen bonds for d2421_a [Å and °].

D-H...A	d(D-H)	d(H...A)	d(D...A)	<(DHA)
O(1)-H(1O)...Cl(3)	0.73(6)	2.33(6)	3.038(3)	164(6)
O(2)-H(2O)...O(1)	0.79(6)	1.87(6)	2.646(4)	169(5)
O(1S)-H(1S)...Cl(3)	0.84	2.35	3.188(8)	173.5

Symmetry transformations used to generate equivalent atoms:

#1 -x+1,y,-z+1

References

[1] Lebedev, Y., Polishchuk, I., Maity, B., Dinis Veloso Guerreiro, M., Cavallo, L., & Rueping, M. Asymmetric Hydroboration of Heteroaryl Ketones by Aluminum Catalysis. *J. Am. Chem. Soc.* **2019**, *141*, 19415–19423.

[2] Huang, R. Z.; Lau, K. K.; Li, Z. F.; Liu, T. L.; Zhao, Y., Rhodium-Catalyzed Enantioconvergent Isomerization of Homoallylic and Bishomoallylic Secondary Alcohols. *J. Am. Chem. Soc*. **2018**, *140*, 14647-14654.

[3] Whyte, A., Mirabi, B., Torelli, A., Prieto, L., Bajohr, J., & Lautens, M. Asymmetric Synthesis of Boryl-Functionalized Cyclobutanols. *ACS Catal.* **2019**, *9*, 9253–9258.

[4] Arora, R., Bajohr, J., & Lautens, M. Rapid Assembly of Unsymmetrically Linked Bis-heterocycles via Palladium Domino Catalysis. *Org. Lett.* **2023**, *25*, 9053–9057.

[5] Wei, W.-X., Li, Y., Wen, Y.-T., Li, M., Li, X.-S., Wang, C.-T., Liu, H.-C., Xia, Y., Zhang, B.-S., Jiao, R.-Q., & Liang, Y.-M. Experimental and Computational Studies of Palladium-Catalyzed Spirocyclization via a Narasaka–Heck/C(sp^3 or sp^2)–H Activation Cascade Reaction. *J. Am. Chem. Soc.* **2021**, *143*, 7868–7875.

[6] Bajohr, J., Dupeux, A., Schenk, D., Jans, C., & Lautens, M. Pd-Catalyzed Domino Narasaka–Heck/C–H Activation/Amination Reactions: Synthesis of Bis-heterocyclic Spirocycles. *Org. Lett.* **2023**, *25*, 5361–5365.

[7] J.-D. Chai, M. Head-Gordon, *Physical Chemistry Chemical Physics* **2008**, *10*, 6615.

[8] S. Grimme, *J. Comp. Chem.* **2006**, *27*, 1787–1799

[9] Li, X.; Frisch, M. J. Energy-Represented Direct Inversion in the Iterative Subspace within a Hybrid Geometry Optimization Method. *J. Chem. Theory Comput.*, **2006**, *2* (3), 835–839.

[10] G. A. Petersson, A. Bennett, T. G. Tensfeldt, M. A. Al-Laham, W. A. Shirley, J. Mantzaris, *J. Chem. Phys.* **1988**, *89*, 2193–2218.

[11] G. A. Petersson, M. A. Al-Laham, *J. Chem. Phys.* **1991**, *94*, 6081–6090.

[12] T. H. Dunning Jr and P. J. Hay in *Modern Theoretical Chemistry*, Plenum, New York, **1997**.

[13] P. J. Hay, W. R. Wadt, *J. Chem. Phys.* **1985**, *82*, 270–283.

[14] W. R. Wadt, P. J. Hay, *J. Chem. Phys.* **1985**, *82*, 284–298.

[15] P. J. Hay, W. R. Wadt, *J. Chem. Phys.* **1985**, *82*, 299–310.

[16] A. D. Becke, *J. Chem. Phys.* **1993**, *98*, 5648–5652.

[17] Grimme, S.; Ehrlich, A.S.; Krieg, H. *J. Chem. Phys.*, **2010**, 132, 154104.

[18] A. D. McLean, *J. Chem. Phys.*, **1980**, 72, 5639-5648.

[19] R. Krishnan, J. S. Binkley, R. Seeger, J. A. Pople, *J. Chem. Phys.*, **1980**, 72, 650-654.

[20] M. P. McGrath, L. Radom, *J. Chem. Phys.*, **1991**, 94, 511-516.

[21] L. A., Curtiss, *J. Chem. Phys.*, **1995**, 103, 6104-6113.

[22] R. C. Binning Jr., L. A. Curtiss, *J. Comp. Chem.*, **1990**, 11, 1206-1216.

[23] M. J. Frisch, J. A. Pople, *J. Chem. Phys.* **1984**, *80*, 3265–3269.

[24] T. Clark, J. Chandrasekhar, G. W. Spitznagel, P. v. R. Schleyer, *J. Comp. Chem.*, **1983**, 4, 294-301.

[25] P. Fuentealba, H. Stoll, L. von Szentpaly, P. Schwerdtfeger, H. Preuss, *Journal of Physics B: Atomic and Molecular Physics* **1983**, *16*, L323–L328.

[26] P. Fuentealba, H. Preuss, H. Stoll, L. Von Szentpály, *Chem. Phys. Lett.* **1982**, *89*, 418–422.

[27] H. Stoll, P. Fuentealba, P. Schwerdtfeger, J. Flad, L. v. Szentpály, H. Preuss, *J. Chem. Phys.* **1984**, *81*, 2732–2736.

[28] M. Dolg, U. Wedig, H. Stoll, H. Preuss, *J. Chem. Phys.* **1987**, *86*, 866–872.

[29] A. V. Marenich, C. J. Cramer, D. G. Truhlar, *J. Phys. Chem. B.* **2009**, *113*, 6378–6396.

[30] Hopmann, K. H. *Organometallics* **2016**, *35*, 3795–3807.

[31] These calculations were carried out with the Python script GoodVibes. I. Funes-Ardoiz and R. S. Paton GoodVibes.py, DOI: 10.5281/zeonodo.124756

[32] M. J. Frisch, G. W. Trucks, H. B. Schlegel, G. E. Scuseria, M. A. Robb, J. R. Cheeseman, G. Scalmani, V. Barone, G. A. Petersson, H. Nakatsuji, X. Li, M. Caricato, A. V. Marenich, J. Bloino, B. G. Janesko, R. Gomperts, B. Mennucci, H. P. Hratchian, J. V. Ortiz, A. F. Izmaylov, J. L. Sonnenberg, D. Williams-Young, F. Ding, F. Lipparini, F. Egidi, J. Goings, B. Peng, A. Petrone, T. Henderson, D. Ranasinghe, V. G. Zakrzewski, J. Gao, N. Rega, G. Zheng, W. Liang, M. Hada, M. Ehara, K. Toyota, R. Fukuda, J. Hasegawa, M. Ishida, T. Nakajima, Y. Honda, O. Kitao, H. Nakai, T. Vreven, K. Throssell, J. A. Montgomery, Jr., J. E. Peralta, F. Ogliaro, M. J. Bearpark, J. J. Heyd, E. N. Brothers, K. N. Kudin, V. N. Staroverov, T. A. Keith, R. Kobayashi, J. Normand, K. Raghavachari, A. P. Rendell, J. C. Burant, S. S. Iyengar, J. Tomasi, M. Cossi, J. M. Millam, M. Klene, C. Adamo, R. Cammi, J. W. Ochterski, R. L. Martin, K. Morokuma, O. Farkas, J. B. Foresman, and D. J. Fox, Gaussian, Inc., Wallingford CT, 2016.

[33] Chemcraft - graphical software for visualization of quantum chemistry computations. Version 1.8, build 682. https://www.chemcraftprog.com

[34] T. Lu, F. Chen, *J. Comput. Chem.* **2012**, *33*, 580–592.

[35] Wang, L., & Wang, C. *J. Org. Chem.* **2019**, *84*, 6547–6556.

[36] Kang, T., Erbay, T. G., Xu, K. L., Gallego, G. M., Burtea, A., Nair, S. K., Patman, R. L., Zhou, R., Sutton, S. C., McAlpine, I. J., Liu, P., & Engle, K. M. *ACS Catal.* **2020**, *10*, 13075–13083.

[37] Bursch, M., Mewes, J., Hansen, A., & Grimme, S. *Angew. Chem. Int. Ed.* **2022**, *61*.

[38] *Reviews in Computational Chemistry* (Vol. 13). (**1999**). Wiley. https://doi.org/10.1002/9780470125908
